# Metabolomic studies in tissues of mice treated with amifostine and exposed to gamma-radiation

**DOI:** 10.1038/s41598-019-52120-w

**Published:** 2019-10-30

**Authors:** Amrita K. Cheema, Yaoxiang Li, Michael Girgis, Meth Jayatilake, Madison Simas, Stephen Y. Wise, Ayodele O. Olabisi, Thomas M. Seed, Vijay K. Singh

**Affiliations:** 1 Department of Oncology, Lombardi Comprehensive Cancer Center, Georgetown University Medical Center, Washington, DC USA; 2 Department of Biochemistry, Molecular and Cellular Biology, Georgetown University Medical Center, Washington, DC USA; 3 Division of Radioprotectants, Department of Pharmacology and Molecular Therapeutics, F. Edward Hébert School of Medicine, Uniformed Serices University of the Health Sciences, Bethesda, MD USA; 4 Armed Forces Radiobiology Research Institute, Uniformed Serices University of the Health Sciences, Bethesda, MD USA; 5 Tech Micro Services, Bethesda, MD USA

**Keywords:** Predictive markers, Metabolomics, Lipids

## Abstract

Although multiple radioprotectors are currently being investigated preclinically for efficacy and safety, few studies have investigated concomitant metabolic changes. This study examines the effects of amifostine on the metabolic profiles in tissues of mice exposed to cobalt-60 total-body gamma-radiation. Global metabolomic and lipidomic changes were analyzed using ultra-performance liquid chromatography (UPLC) quadrupole time-of-flight mass spectrometry (QTOF-MS) in bone marrow, jejunum, and lung samples of amifostine-treated and saline-treated control mice. Results demonstrate that radiation exposure leads to tissue specific metabolic responses that were corrected in part by treatment with amifostine in a drug-dose dependent manner. Bone marrow exhibited robust responses to radiation and was also highly responsive to protective effects of amifostine, while jejunum and lung showed only modest changes. Treatment with amifostine at 200 mg/kg prior to irradiation seemed to impart maximum survival benefit, while the lower dose of 50 mg/kg offered only limited survival benefit. These findings show that the administration of amifostine causes metabolic shifts that would provide an overall benefit to radiation injury and underscore the utility of metabolomics and lipidomics to determine the underlying physiological mechanisms involved in the radioprotective efficacy of amifostine. This approach may be helpful in identifying biomarkers for radioprotective efficacy of amifostine and other countermeasures under development.

## Introduction

It is well-recognized that intense exposure to ionizing radiation produces injuries that can be expressed either soon after radiation exposure as acute syndrome(s) or can be displayed as late-arising pathologies (delayed or late effects) that are the direct result of the time-dependent progression of initial injuries^1^. Acute radiation syndrome (ARS) is representative of the early-occurring syndromes, while radiation-induced tissue fibrosis (pulmonary or myelofibrosis) or malignancy (solid tumors, leukemias or related myeloproliferative diseases) are representative of the late-arising diseases induced by prior, sufficiently intense, radiation exposure^2–6^. The induction and severity of these pathologic syndromes are largely governed by radiological and biological factors^7–9^. Many of these radiological and biological factors have been investigated with the use of both large and small animal models^10–12^.

Development of suitable radiation countermeasures to protect individuals, and the public at large, from serious, potentially lethal injuries following radiation exposures remains a substantial unmet medical need. Further, suitable, pharmacologic protection against less severe types of radiation injuries (i.e., sublethal radiation injuries) remain a major health concern as well. To date, no radioprotector for ARS has received approval for general use from the United States Food and Drug Administration (US FDA); however, three radiomitigative agents have been approved recently by the FDA for post-exposure treatment of evolving hematopoietic ARS^13–15^. Amifostine (WR2721, Ethyol), 2-(3-aminopropyl) aminoethylphosphorothioate, is a radioprotective agent which has been shown to (i) reduce or limit the extent of acute radiation injury through mainly free radical scavenging, although other cytoprotective mechanisms have also been documented as well (direct protection of DNA, enhancing molecular and cellular repair processes, induction of tissue hypoxia) and (ii) provide protection against late-arising, radiation-induced malignancies through the drug’s capacity to counter radiation-induced mutagenesis^16–21^. Amifostine has already been approved by the FDA for limited indications: (i) to reduce the renal toxicity with repeated use of cisplatin in ovarian cancer patients in 1996, (ii) to reduce the occurrence of xerostomia in head and neck cancer patients undergoing radiotherapy in 1999^22–26^. Because of its side effects, amifostine was not approved as a radioprotector for ARS for protection of high-risk individuals from lethal doses of radiation exposure^27^. However, it has been demonstrated that by using significantly low doses of amifostine prophylactically, its toxic side-effects can be minimized while still maintaining significant levels of radioprotection^21,28^. The radioprotective threshold for amifostine dosing appear to lie between 25 and 50 mg/kg for mice. Mature, lineage-restricted progenitors appear to be more responsive to the protective effects of low doses of amifostine than the more primitive, multipotential progenitors. Currently, this agent is gaining attention for possible regulatory approval by using a drug development strategy, namely a polypharmacy approach, of combining low doses of the drug with other radiation countermeasures in order to reduce its side effects^21,29,30^. Recently, we have demonstrated that amifostine at low doses (30 and 50 mg/kg) enhanced the efficacy of low doses of γ-tocotrienol (25 and 50 mg/kg) in mice. This study suggests that amifostine and γ-tocotrienol may be used in combination at lower doses and still achieve optimal protection against a lethal dose of radiation without producing adverse effects^29^.

**Figure 1:**
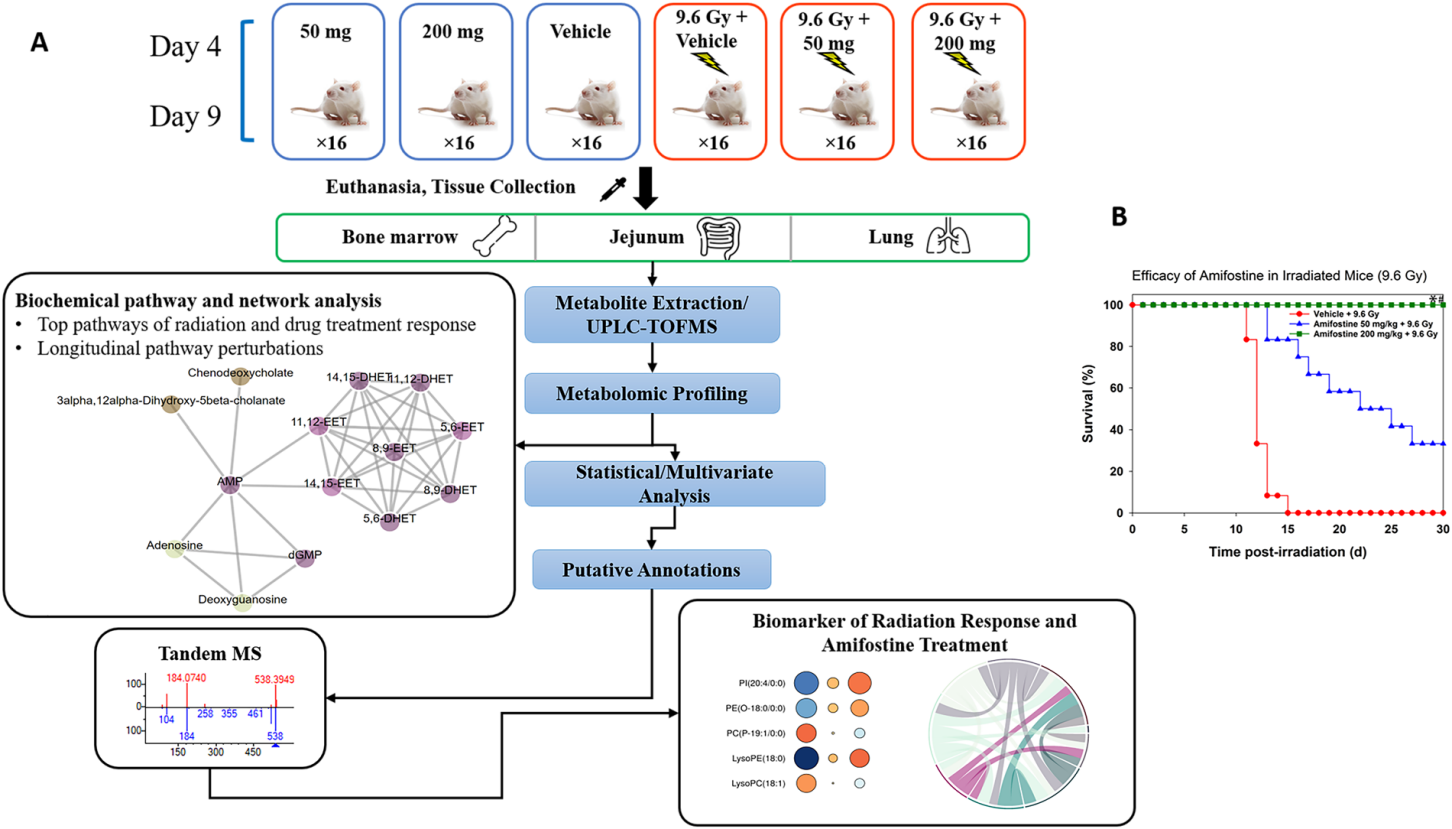
(**A)** Experimental and analytical design of the study. Bone marrow, jejunum and lung samples were collected from irradiated and/or amifostine treated mice at day 4 or 9 and processed for untargeted LC-MS profiling. Metabolomics pathway and network analysis were analyzed by Mummichog v2.0. Putative annotation was performed by an in-house CEU Mass Mediator RESTful API service, which search Kegg, HMDB, LipidMaps, Metlin,and PubChem and is consumed in the R package “cmmr”. Validation with tandem mass spectrometry was performed by the TandemQuery tool (Li *et al*., unpublished) and the NIST 2017 MS/MS spectra database. **(B)** Radioprotective efficacy of different doses of amifostine administration in irradiated CD2F1 male mice. Mice were administered vehicle or amifostine (50 or 200 mg/kg) 30 min prior to irradiation (^60^Co γ-radiation, 9.6 Gy, 0.6 Gy/min). Survival was monitored for 30 days post irradiation.

There were two major goals of this study: the first goal was to study metabolic responses in bone marrow, jejunum, and lung of mice exposed to LD_90/30_ dose of total-body ^60^Co γ-radiation to understand tissue specific differences in overall response after 4 and 9 days post-irradiation; the second goal was to gain a better understanding of both temporal and dose-dependent differences in metabolic profiles following administration of the radioprotective drug, amifostine. Additionally, we mined our data in order to identify metabolic corrections due to amifostine’s pre-treatments that served, at least in part, to attenuate radiation induced tissue damage (Fig. 1A). Our results demonstrate that exposure to ionizing radiation induced a robust response in bone marrow and to some extent in the jejunum, however, lung showed modest changes in metabolomic and lipidomic profiles. Furthermore, prior treatment with amifostine showed dose-dependent, maximal reversals of metabolic dysfunction in bone marrow and to some extent in jejunum.

## Results

### Treatment with amifostine prior to irradiation significantly increases survival

In order to assess the radioprotective efficacy of amifostine, two doses of amifostine were evaluated for survival benefit in mice exposed to comparable, whole-body doses of ionizing radiation (9.6 Gy). The two doses were chosen based not only on current clinical use and toxicity, but also prior experimental work (21). There were six groups of mice and each group had 16 mice. All mice were monitored for survival for 30 days. Consistent with prior experimental observations (21), we observed 100% survival in mice treated with 200 mg/kg amifostine, 33% survival in mice treated with 50 mg/kg amifostine and 0% survival in the vehicle-treated group (Fig. 1B). Treatment with 200 mg/kg amifostine clearly yielded significantly higher survival compared to the vehicle control (p < 0.0001). An apparent, but marginal survival benefit was observed with 50 mg/kg dose of amifostine compared with vehicle control, but proved statistically not to be significant (p = 0.093). Survival difference between 50 mg/kg and 200 mg/kg was significant (33% vs 100%, p = 0.001) suggesting that 200 mg/kg was optimal, and 50 mg/kg was suboptimal dose for survival benefit against lethal dose of radiation in mice.

### Metabolic changes correlate with radiation induced tissue injury in bone marrow, jejunum and lung

**Figure 2:**
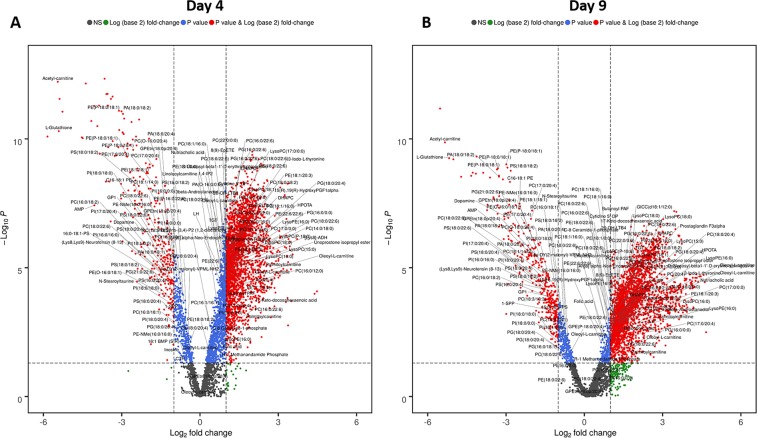
Volcano plot showing dysregulated metabolites for bone marrow at 4 and 9 days post-irradiation. All annotated metabolites have a FDR adjusted significant p-value (<0.05) comparing radiation versus vehicle and were validated by tandem mass spectrometry.

We used high resolution mass spectrometry based untargeted metabolomics and lipidomics approaches to delineate metabolic changes that accompany exposure to ionizing radiation in three tissue types, namely, bone marrow, jejunum and lung. LC-MS data was pre-processed using the XCMS R package and yielded 2675 (bone marrow), 3582 jejunum, 2761 (lung) and 3439 (bone marrow), 3710 (jejunum), and 3541 (lung), features in electrospray positive and negative modes, respectively. Initial examination of the total features, using partial least squares-discriminant analysis (PLS-DA), indicated reasonable separation between molecular profiles of irradiated mice compared to sham-irradiated mice for all three tissue types (Supplementary Figs 1–4). The metabolites with the greatest contribution to the group separations were annotated after level 2 validation by tandem mass spectrometry and were visualized as volcano plots for bone marrow (Fig. 2), jejunum and lung (Supplementary Figs 5 and 6, respectively). Overall, the number of significantly dysregulated metabolites across all comparative groups was 1,614 of which 186 metabolites were validated. An exhaustive list of validated metabolites with fragmentation information is tabulated in Supplementary Table 1. Metabolic and lipidomic profiles of bone marrow showed a large number of dysregulated metabolites at day 4 and 9 that were characteristic of an ARS (Fig. 2, panels A and B). We observed changes in the tissue across several classes of lipids including phosphatidylcholines, phosphatidylserines, phosphatidylinositols, and acylcarnitines indicative of dyslipidemia that has been reported by us and others previously^31,32^. We also observed decrease in glutathione, acetyl carnitine, dopamine, neurotensin, N-steaoryltaurine, and adenosine monophosphate with a concomitant increase in long chain acylcarnitines (LCACs), butanoyl-platelet activating factor (PAF), prostaglandins and other eicosanoids and nutriacholic acid. These metabolites have important functional and regulatory roles in biological systems and can help explain the mediation of acute response to radiation exposure and onset of radiation induced tissue injury^33–36^. We performed further pathway analysis using Mummichog v2.0 which indicated changes in carnitine shuttle, tyrosine and phosphatidylinositol metabolism with significant overlap size, pathway size, and p-value (Table 1)^37^. Similar analysis of jejunum showed changes in tissue levels of several metabolites of which we were able to validate a subset that predominantly included bile acids, glutathione, glycerophospholipids, and arachidonic acid metabolites (Supplementary Fig. 5 and Supplementary Table 2A). There were relatively few changes in lung metabolic profiles (Supplementary Fig. 6 and Supplementary Table 2B) showing that it was not responsive to acute irradiation effects, at least at the level of radiation exposure employed.

**Table 1:** Radiation induced pathway dysregulation in bone marrow at 4 and 9 days post-irradiation.

Pathway	Day 4		Day 9	
	*overlap size*	*p-value*	*overlap size*	*p-value*
Carnitine shuttle	22(22)^a^	2.27E-03	—	—
Tyrosine metabolism	6(6)	1.24E-02	10(10)	1.93E-02
Phosphatidylinositol phosphate metabolism	5(5)	2.21E-02	5(5)	3.68E-02
Histidine metabolism	4(4)	3.53E-02	—	—
Bile acid biosynthesis	4(4)	3.53E-02	—	—
Xenobiotics metabolism	—	—	7(7)	4.73E-02

*Note*. Pathway analysis result with positive mode and negative mode combined.

^a^Numbers in parenthesis indicates the pathway size.

### Treatment with amifostine does not elicit major metabolic perturbations in mice

Although amifostine has been cleared for clinical use for the treatment of xerostomia at lower doses (50 mg/kg) not much is known about how this drug impacts metabolism^38,39^. Additionally, metabolic changes that correlated with drug toxicity at higher doses (200 mg/kg) have not been elucidated. Hence, going into this study, we sought to understand dose dependent metabolic perturbations at 4 and 9 days in all three tissue types that would also provide insights into metabolic changes that correlate with observed drug toxicity at higher doses. A comprehensive list of all metabolites that changed with significant p-value (with and without FDR correction) is tabulated in Supplementary Table 3A, and is further broken down as metabolites showing changes exclusively to the response of administration of 200 mg/kg of amifostine at 4 days (Table 2), 9 days (Supplementary Table 3B), and for 50 mg/kg at 4 and 9 days (Supplementary Table 3C,D) across all the three tissue types. Surprisingly, while there were changes in glycerophospholipids and acylcarnitines in bone marrow and to some extent in jejunum and lung, none of these metabolites had either a significant p-value after correcting for FDR (false discovery rate) or fold change at either doses. In lung, however, we observed a >10-fold increase (with highly significant FDR corrected p-value), in the endogenous levels of hydroxyphenyl-2-hydroxyethyl oleamide (omdm-2) at both doses of amifostine tested in the study. Omdm-2 is a fatty acid amide hydrolase (FAAH) inhibitor^40^. FAAH is the principal catabolic enzyme for a class of bioactive lipids called the fatty acid amides. Inhibition of this enzyme may prevent the release of free fatty acids from membrane lipids leading to a slow or a complete shutdown of the inflammatory signals triggered by free fatty acids. Taken together, accumulation of this metabolite in lung could help impart resistance to radiation induced tissue injury via anti-inflammatory and by attenuating oxidative stress caused by exposure to ionizing radiation.

**Table 2:** Metabolic changes in bone marrow, jejunum, and lung in response to amifostine treatment at 200 mg/kg.

Name	Organ/Tissue	*p-value*	*FDR* ^*a*^	*Fold Change*	*Log2(FC)*
PG(18:0/20:4)	Bone marrow	2.61E-05	2.69E-02	1.4681	**↑** 0.5540
PC(O-16:0/20:4)	Bone marrow	3.77E-02	*NS*	1.1399	**↑** 0.1889
PA(18:0/20:4)	Bone marrow	3.33E-02	*NS*	1.1413	**↑** 0.1907
PE(P-16:0/22:6)	Bone marrow	1.20E-03	*NS*	1.2031	**↑** 0.2668
PG(18:0/22:6)	Bone marrow	1.53E-02	*NS*	1.2523	**↑** 0.3246
PE(18:0/22:6)	Bone marrow	2.07E-02	*NS*	2.0152	**↑** 1.0110
PC(p-18:0/18:1)	Bone marrow	3.72E-02	*NS*	1.2359	**↑** 0.3056
PC(18:0/22:6)	Bone marrow	3.40E-02	*NS*	1.2078	**↑** 0.2724
PG(16:0/18:1)	Bone marrow	1.60E-02	*NS*	1.3228	**↑** 0.4036
N-Stearoyl taurine	Bone marrow	2.95E-02	*NS*	1.1863	**↑** 0.2465
PC(18:0/20:4)	Bone marrow	2.64E-02	*NS*	1.2759	**↑** 0.3515
PE(16:0/20:4)	Bone marrow	2.06E-02	*NS*	1.1394	**↑** 0.1882
GPE(P-18:0/20:4)	Bone marrow	1.24E-02	*NS*	1.1534	**↑** 0.2059
Oleoyl-L-carnitine	Bone marrow	3.63E-02	*NS*	0.6242	**↓** −0.6799
PI(20:4/0:0)	Jejunum	4.33E-02	*NS*	0.6477	**↓** −0.6266
L-Cysteine-glutathione disulfide	Jejunum	2.42E-02	*NS*	0.4403	**↓** −1.1835
Docosahexaenoylglycine	Jejunum	1.50E-02	*NS*	0.7432	**↓** −0.4282
Tauroursodeoxycholic acid	Jejunum	1.58E-03	*NS*	1.5609	**↑** 0.6424
Hydroxyphenyl-2-hydroxyethyl oleamide	Lung	1.14E-13	3.91E-10	31.1710	**↑** 4.9621
Lyso-SM	Lung	1.74E-02	*NS*	0.7263	**↓** −0.4614

*Note*. All metabolite names are validated through tandem MS.

^a^Numbers are FDR adjusted *P* values. NS = not significant (FDR, *P* value > 0.05).

### Treatment with amifostine attenuates metabolic consequences of irradiation in bone marrow

**Figure 3:**
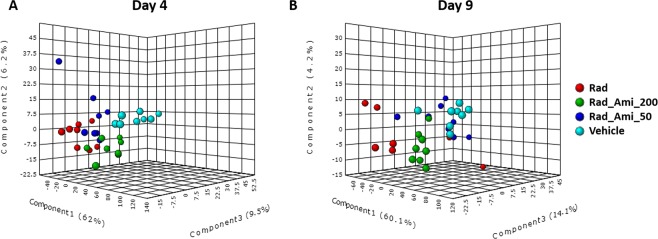
A three-dimensional PLS-DA plot showing separation for study groups vehicle only, radiation only, amifostine 50 mg + radiation and amifostine 200 mg + radiation based on metabolic profiles of bone marrow at day 4 (panel A) and day 9 (panel B) post-irradiation, negative mode.

**Figure 4:**
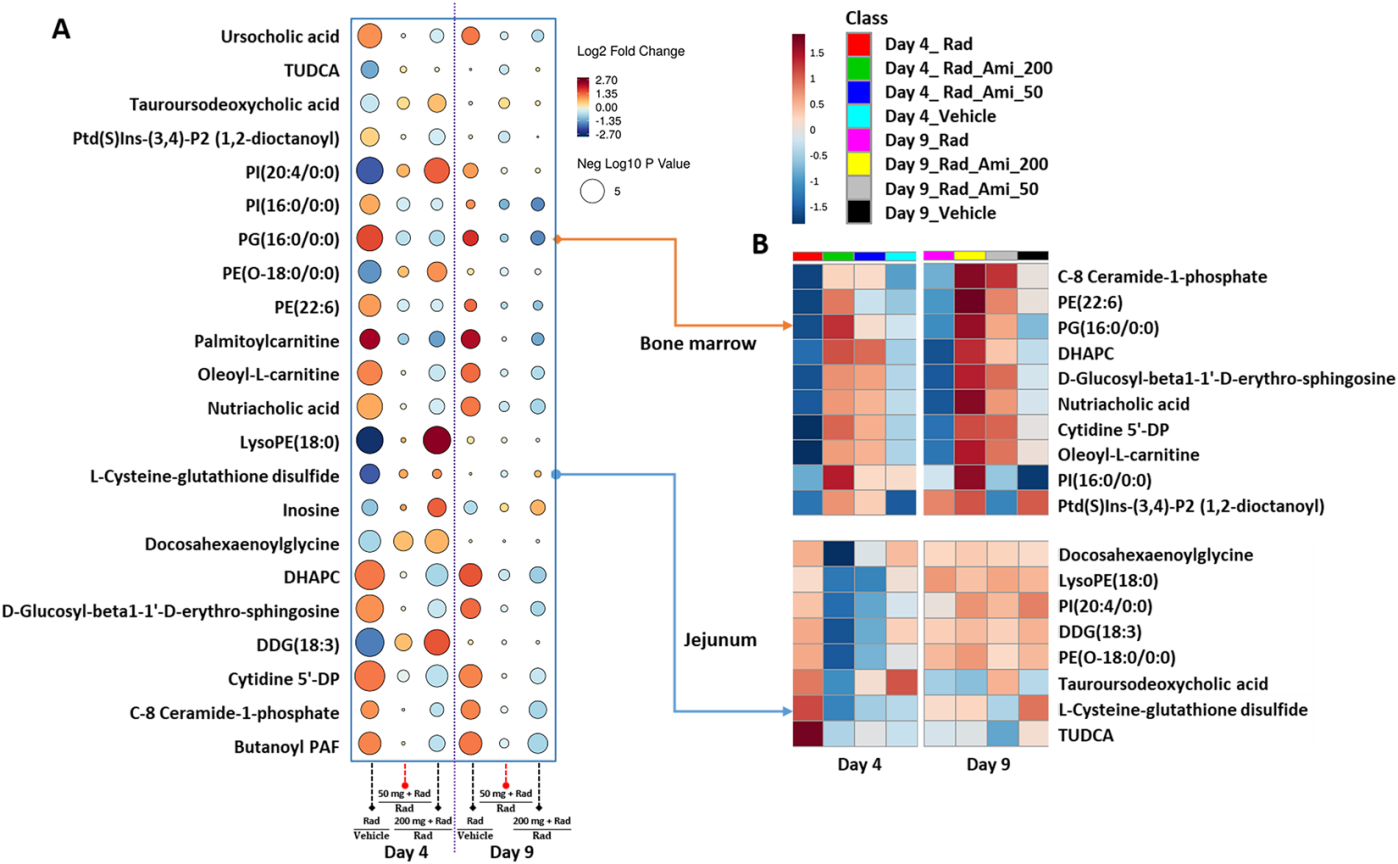
Correction of endogenous levels of metabolites by amifostine treatment in bone marrow is dose dependent. A subset of metabolites for bone marrow (panel A) and overlapping metabolite patterns in bone marrow and jejunum, respectively (panel B).

The findings so far indicate that the extent of metabolic changes within bone marrow during evolving ARS is greater than in other organ systems/tissues studied to date. Given that amifostine treatment resulted in increased survival, we wanted to investigate if this is mediated, at least in part, by attenuation of radiation induced biochemical pathway perturbations. We used PLS-DA to visualize group differences due to metabolic changes in bone marrow, jejunum, and lung (Fig. 3, Supplementary Figs 2–4). Group separation for changes in bone marrow at both day 4 an day 9 can be seen in Fig. 3, panels A and B accordingly. Examination of these plots showed maximal separation between the irradiated and sham groups while *a prior* treatment with the drug seemed to cause dose-dependent metabolic shifts towards the metabolic profiles noted in control animals. Comparative studies of the metabolomic and lipidomic profiles in bone marrow showed restoration of endogenous levels of a large number of metabolites at both doses of amifostine (Fig. 4, Supplementary Fig. 7). Figure 4 shows a raindrop plot illustration of endogenous levels of metabolites including N-acylamino acids, LCACs, phosphatidylethanolamines, and inositol that change in response to radiation and that are corrected by amifostine treatment in a dose dependent manner (Fig. 4, panel A). Interestingly, some of the metabolites including bile acids, ceramide-1-phosphate, oleolyl-L-carnitine and glutathione overlapped between bone marrow and jejunum (Fig. 4, panel B) at 50 and 200 mg/kg doses of amifostine. We performed pathway analysis and found correction in prostaglandin synthesis, linolenate metabolism, and fatty acid oxidation that seemed to impart maximum benefit from radiation injury in the bone marrow (Table 3). These results emphasize the efficacy of amifostine in treating hematopoietic subsyndrome, at least in part, via multiple pathway corrections.

**Table 3:** Mummichog v2.0 based pathway correction of radiation injury in bone marrow by treatment of amifostine 50 mg/kg.

Pathway	Day 4		Day 9	
	*overlap size*	*p-value*	*overlap size*	*p-value*
Prostaglandin formation from arachidonate	3(7)^a^	8.40E-05	0(7)	1.68E-02
Linoleate metabolism	3(7)	3.36E-04	0(3)	1.68E-02
Di-unsaturated fatty acid beta-oxidation	2(3)	9.24E-04	0(1)	1.68E-02
D4&E4-neuroprostanes formation	0(2)	3.70E-02	1(2)	3.02E-03
Carnitine shuttle	4(22)	5.29E-03	0(2)	1.68E-02
Fatty acid metabolism	2(5)	5.29E-03	0(1)	1.68E-02
Xenobiotics metabolism	1(1)	8.57E-03	0(1)	1.68E-02
Arachidonic acid metabolism	2(8)	8.57E-03	0(8)	1.68E-02
Ascorbate (vitamin C) and aldarate metabolism	1(1)	8.57E-03	0(1)	1.68E-02
Glutamate metabolism	1(1)	8.57E-03	0(1)	1.68E-02
Leukotriene metabolism	2(8)	8.57E-03	0(8)	1.68E-02
Pyruvate metabolism	1(1)	8.57E-03	0(1)	1.68E-02
Glycerophospholipid metabolism	3(16)	1.44E-02	0(10)	1.68E-02
Tryptophan metabolism	1(2)	1.60E-02	0(2)	1.68E-02
Glutathione metabolism	1(2)	1.60E-02	0(2)	1.68E-02
Aspartate and asparagine metabolism	1(2)	1.60E-02	0(2)	1.68E-02
Putative anti-Inflammatory metabolites formation from EPA	1(2)	1.60E-02	0(2)	1.68E-02
Fatty acid activation	2(8)	1.61E-02	0(3)	1.68E-02
Vitamin A (retinol) metabolism	0(7)	3.70E-02	0(7)	1.68E-02
De novo fatty acid biosynthesis	2(9)	1.97E-02	0(4)	1.68E-02
Glycine, serine, alanine and threonine metabolism	1(3)	2.48E-02	0(3)	1.68E-02
Androgen and estrogen biosynthesis and metabolism	1(3)	2.48E-02	0(3)	1.68E-02
Tyrosine metabolism	1(6)	3.28E-02	0(6)	1.68E-02
Methionine and cysteine metabolism	1(3)	2.48E-02	0(3)	1.68E-02
Histidine metabolism	1(4)	2.76E-02	0(4)	1.68E-02

*Note*. Pathway analysis result with positive mode and negative mode combined.

^a^Numbers in parenthesis indicates the pathway size.

**Figure 5:**
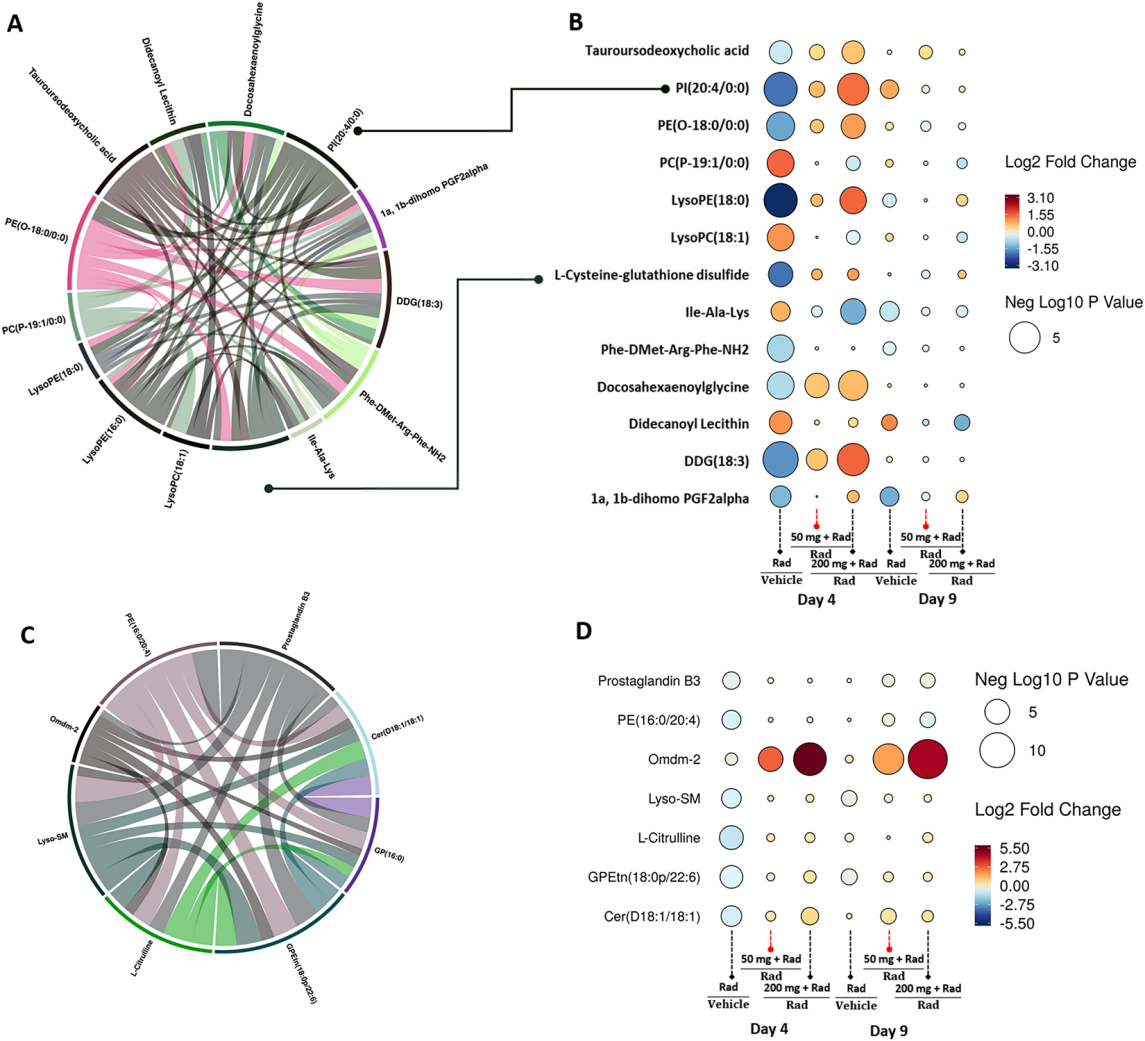
(**A)** Amifostine treatment results in modest changes in metabolite profiles of jejunum. (panel A) Circos plot showing most correlated metabolite patterns (panel B) Raindrop illustration of metabolite patterns that correlate with radioprotection in jejunum with 50 and 200 mg/kg of amifostine at 4 and 9 d post-irradiation. (**B**) Amifostine treatment results in modest changes in metabolite profiles of lung. (panel C) Circos plot showing most correlated metabolite patterns (panel D) Raindrop illustration of metabolite patterns (union set) that correlate with radioprotection in lung with 50 and 200 mg/kg of amifostine at 4 d and 9 d post-irradiation.

Furthermore, similar analysis of jejunum showed that bile acids and glycerophospholipids showed maximum correlation with amifostine mediated attenuation of metabolic disturbances caused by acute irradiation injury. The latter was visualized by circos plots (Fig. 5, panel A), suggesting a reversal to near normal levels with 50 mg/kg dose and to a higher extent with the higher dose (Fig. 5, panel B). Pathway analysis showed correction of folate, vitamin A, and amino acid metabolism with the 50 mg/kg dose of amifostine in jejunum (Supplementary Table 4A) while the higher dose resulted in a higher number of pathway corrections that included arachidonic acid metabolism, steroid hormone biosynthesis, glutathione, and amino acid metabolism (Supplementary Table 4B). These results also help explain why the higher dose leads to a better survival by minimizing radiation induced adverse metabolic outcomes.

Finally, we analyzed putative radioprotective effects of amifostine as manifested as a correction of endogenous metabolite abundance (Fig. 5C,D and Supplementary Table 5A,B). As discussed in the previous sections, metabolite profiles of lung did not show robust changes following acute irradiation. When visualized as a circos plot and a rain drop plot (Fig. 5, panels C and D, respectively), the metabolites that correlated maximally to the protective effects of amifostine included citrulline, ceramides, and prostaglandins. Recovery of the pathway profiles was dose dependent; at 50 mg/kg, we observed recovery of steroid hormone biosynthesis and glycerophospholipid metabolism while treatment with the higher amifostine dose resulted in a larger number of pathway corrections.

In summary, these results show that treatment with 200 mg/kg resulted in multiple metabolic pathway corrections while the lower dose also offered robust but partial alleviation of the metabolic disturbances resulting from irradiation. The drug was most effective in correcting hematopoietic injury and to some extent gastrointestinal injury at days 4 and 9 after irradiation. This is not surprising since amifostine is an agent that helps to ameliorate hematopoietic ARS (H-ARS).

## Discussion

Amifostine is a FDA-approved drug for radio/cytoprotection^18–21^. In spite of such approval, this agent has been allowed for limited indications; the dry mouth/xerostomia and the renal toxicity in patients of ovarian cancer^41,42^. Because of the potentially severe side-effects of upper and lower gastrointestinal tract and hypotension, amifostine was not approved as a radioprotector for general use for high risk personnel. However, we have demonstrated that by using sufficiently low doses of amifostine prophylactically, its toxic side-effects can be minimized while still maintaining significant levels of radioprotection^21^.

It has been reported earlier that prophylactic administration of amifostine at a dose of 50 mg/kg significantly regenerated bipotential progenitors, moderately regenerated multipotential progenitors, and had no effect on more primitive progenitors in irradiated mice^21^. A dose of 25 mg/kg of amifostine failed to produce radioprotective effects on the progenitor subtypes. Lineage-restricted mature progenitors were more responsive to the effects of low doses of amifostine compared with the more primitive, multipotential progenitors. In recent past, it has been demonstrated that combination of low doses of amifostine and γ-tocotrienol significantly enhanced mouse survival compared to single treatments of either amifostine or γ-tocotrienol in irradiated animals^29^. The development of such prophylactic radiation countermeasures using poly-pharmacy approach would be exceedingly useful for military personnel and first responders to nuclear/radiological contingencies. There are FDA-approved agents which can be repurposed for use with low doses of amifostine. Recently, such strategy is getting traction in the field of radiation countermeasure development for ARS and several groups are trying to exploit the benefits of poly-pharmacy and repurposing strategy.

Based on the above observations, we conducted a metabolomics/lipidomic study using an acute radiation rodent model. For this study, we used suboptimal doses of amifostine (50 mg/kg) and compared it with more optimal amifostine doses of 200 mg/kg in order to identify metabolomic product differences. These two doses are comparable to suboptimal and optimal dose of amifostine for human use and hence were used in this study. Optimal dose of amifostine used for radioprotection against ARS provides unparalleled protection in preclinical models but has shown adverse side effects in humans at doses needed for radioprotection of individuals with ARS. The dose conversion between animal model and human is central for any drug development program^43^. Allometric dose conversion from dose for body weight in animals to dose for human body surface area takes into account the variance in body surface. Such allometric method consideration differences of anatomical, physiological, and biochemical parameters between species. It also takes into consideration the drug pharmacokinetics. In a recent publication, different equations have been described for dose extrapolation^43^.

Here, we embarked upon a systematic investigation that involved a total of 288 tissue samples (bone marrow (N = 96), jejunum (N = 96) and lung (N = 96), obtained from mice (N = 16/group), that were either irradiated with 9.6 Gy γ-radiation or sham irradiated (N = 16). Mice were treated either with amifostine at 50 mg/kg (N = 16) or at 200 mg/kg (N = 16), or treated with saline (drug vehicle) alone that served as a control. We selected two time points for tissue collection for metabolomics/lipidomic analysis; 4 and 9 days post-irradiation. Day 4 post-irradiation for irradiated mice is comparable to the latent phase of human which follows the prodromal phase and exposed individuals will be relatively symptom free while day 9 is comparable to the illness phase presenting clinical symptoms associated with the manifestation of pathologies. Our goal was to compare metabolomic and lipidomic profiles between these two clinically relevant phases in irradiated and treated with two different doses of amifostine. In order to understand tissue specific impact of irradiation and consequent metabolic correction by amifostine treatment, we performed a combination of multivariate and univariate statistics using in house developed R scripts. Overall, we performed 96 comparisons for the positive and negative mode data for all three tissue type resulting in 1,614 dysregulated metabolites that were subjected to tandem MS, leading to validation of 186 metabolites across the different comparative groups (Supplementary Table 6). Initially, we investigated the acute effects of irradiation at two early, post-irradiation time points (4 d and 9 d) in each of the three tissue types (bone marrow, jejunum and lung) and found that the response to irradiation was tissue dependent. For example, bone marrow was most susceptible to acute effects of irradiation and showed a decrease in anti-oxidant metabolites like glutathione, N-acylamino acids and a concomitant increase in pro-inflammatory metabolites including prostaglandins and PAF. Jejunum also showed an increase in pro-inflammatory metabolites including bile acids, arachidonic acid, and a decrease in glutathione suggesting some acute effects^44,45^. Similar investigations of lung showed few changes in metabolomics and lipidomic profiles, suggesting that lung of irradiated mice were relatively resistant to acute responses elicited by the level of irradiation (employed at the level). This is expected since lung is an organ where we observe generally a prominent delayed pathologic effect of acute irradiation at these high, potentially lethal levels of exposure (so called delayed effect of acute radiation exposure or DEARE). The time points of sample harvest were not appropriate for studying DEARE, only for examining acute effects for which bone marrow was an optimal hematopoietic tissue. In general, these noted shifts in metaobolic profiles following acute irradiation support and are consistent with prior reported observations, based largely on older analytic technologies^46–51^. While we have focused on a subset of metabolites that help explain radiation response and possible pathway attenuation by amifostine treatment, we have included all data to facilitate meta-analysis by the scientific community as well as integrative analyses with other “omics” data sets as they become available.

A primary goal of this study was to develop a mechanistic picture with broadest of brush strokes, the metabolic basis and linkages of amifostine’s radioprotective nature, relative to its inherent, drug-dose-dependent toxicities. As such, we wanted to examine the drug’s capacity to minimize the extent of metabolic disturbances induced by acute irradiation and, in turn, to reduce potential lethal outcomes. As the latter (alleviation of lethality risk) is strongly dependent on the level of initial drug dosing and is also strongly associated with systemic drug toxicity, we first asked if the drug treatment results in tissue toxicity when evaluated from a metabolic standpoint. To our surprise, at both doses, we found few metabolic changes that had a significant fold change and p-value. Interestingly, hydroxyphenyl-2-hydroxyethyl oleamide was one of the few metabolites that was found to be upregulated (p = 1.14E-13) in the lung of the amifostine treated group, at both doses. This metabolite is an endocannabinoid analogue that inhibits fatty acid amide hydrolase (FAAH) within the neurons^52^. This action leads to mitigation of the release of pro-inflammatory fatty acids like arachidonic acid and may contribute to the anti-inflammatory activity of amifostine.

We then checked to see if there were metabolic correlations between the observed protective effects and amifostine treatment. We found that treatment with amifostine led to correction of endogenous levels of selected species of metabolites in bone marrow in a dose dependent manner. For example, C-8 ceramide-1-phosphate that was up-regulated after irradiation, reversed to near normal abundance with the administration of amifostine at both doses in the bone marrow at both time points^53^. A similar trend was observed with cytidine-5′-DP, a pyrimidine ribonucleoside 5′-diphosphate, elevation of this metabolite in response to radiation exposure is reported to be indicative of DNA damage and cell death^54^. Treatment with the lower dose of amifostine also helped reverse levels of LCACs in the bone marrow. These acyl carnitine species are known to be associated with fatty acid transport into the mitochondria as well as for glycerophospholipids that are crucial for maintaining cellular and membrane integrity. A similar low dose benefit was also observed in jejunum wherein the endogenous levels of long chain acyl carnitines, bile acids, glutathione, and phosphatidyl inositol seemed to revert to control levels, thus providing alleviation of radiation injury^55^.

Next, we performed metabolic pathway analysis (Mummichog v2.0 analysis) to understand the effects of the drug on mitigating biochemical pathway perturbations caused by radiation exposure. Mummichog is a free Python program for analyzing data from high throughput, untargeted metabolomics. The software leverages the organization of metabolic networks to predict functional activity directly from feature tables, bypassing metabolite identification^56^. Treatment with both doses of amifostine led to changes in carnitine shuttle, tyrosine metabolism, phosphatidyl inositol phosphate mechanism, histidine metabolism, and bile acid biosynthesis in bone marrow (Tables 3 and 4). Carnitine is involved in transporting fatty acids across the mitochondrial membrane by forming a LCAC ester. Carnitine also plays a role in stabilizing acetyl CoA and coenzyme A levels through the ability to receive or give an acetyl group^57^. Taken together, these changes suggest a dysregulated mitochondrial function in response to radiation that seems to be corrected at least in part by treatment with amifostine.

**Table 4:** Mummichog v2.0 based pathway correction of radiation injury in bone marrow by treatment of amifostine 200 mg/kg.

Pathway	Day 4		Day 9	
	*overlap size*	*p-value*	*overlap size*	*p-value*
Tyrosine metabolism	10(10)^a^	1.13E-02	9(10)	1.09E-03
Androgen and estrogen biosynthesis and metabolism	—	—	6(6)	1.60E-03
Phosphatidylinositol phosphate metabolism	5(5)	5.55E-03	5(5)	4.45E-03
Leukotriene metabolism	4(4)	4.36E-02	4(4)	1.08E-02
Histidine metabolism	4(4)	1.11E-02	—	—
Carnitine shuttle	—	—	15(22)	1.35E-02
Bile acid biosynthesis	—	—	4(4)	1.63E-02
Glycerophospholipid metabolism	—	—	8(10)	2.16E-02
Purine metabolism	—	—	5(6)	2.26E-02
Glycosphingolipid biosynthesis - ganglioseries	3(3)	2.53E-02	3(3)	2.37E-02
Glycine, serine, alanine and threonine metabolism	5(5)	2.51E-02	3(3)	2.37E-02
Methionine and cysteine metabolism	5(5)	2.51E-02	3(3)	2.37E-02
Linoleate metabolism	3(3)	2.53E-02	3(3)	2.37E-02
Vitamin A (retinol) metabolism	—	—	6(7)	2.41E-02
Vitamin E metabolism	3(3)	2.53E-02	—	—
Arachidonic acid metabolism	—	—	3(3)	2.59E-02
Selenoamino acid metabolism	—	—	3(3)	2.59E-02
Pentose phosphate pathway	—	—	3(3)	2.59E-02
Prostaglandin formation from arachidonate	—	—	6(8)	3.45E-02
Sialic acid metabolism	5(6)	3.66E-02	5(6)	3.48E-02
Xenobiotics metabolism	7(7)	3.50E-02	—	—
Aspartate and asparagine metabolism	4(4)	4.36E-02	—	—

*Note*. Pathway analysis result with positive mode and negative mode combined.

^a^Numbers in parenthesis indicates the pathway size.

In summary, while treatment with 200 mg/kg appeared to trigger a massive recovery response in bone marrow and to some extent in jejunum and lung that correlates with 100% survival in irradiated animals; treatment with the lower dose of 50 mg/kg also leads to the mitigation of dysfunction of multiple pathways, as seen at the higher dose as well (Supplementary Table 7). These results show that the lower dose of amifostine improves metabolic outcomes of radiation exposure in a tissue dependent manner and thus may provide benefit towards alleviation of radiation induced tissue injury.

One of the constraints of this study is that we have not been able to address what metabolic changes correlate with drug toxicity associated with higher doses in humans since control mice (saline treated mice) did not seem to show any adverse metabolic consequences at the 200 mg/kg dose. Nevertheless, our findings emphasize the metabolic benefit of this drug at lower doses as a prophylaxis for providing protection against radiation induced organ or tissue injury.

## Materials and Methods

### Mice

Male 6–8 week-old CD2F1 mice were purchased from a commercial vendor (Envigo, Indianapolis, IN, USA) and subsequently housed (four per cage) following arrival in an environmentally controlled facility accredited by the Association for Assessment and Accreditation of Laboratory Animal Care-International. All mice were kept in rooms with a 12-hour light and 12-hour dark cycle. The mouse holding room was maintained at 20–26 °C with 10–15 air exchange cycle/h and a relative humidity of 30–70%. Mice were held in quarantine for one week. A microbiological examination of representative samples ensured the absence of *Pseudomonas aeruginosa*. Mice were provided certified rodent rations (Teklad Rodent Diet, Envigo) and acidified water (pH = 2.5–2.8) *ad libitum*^29^. All animal procedures were performed according to a protocol approved by the Institutional Animal Care and Use Committee (Protocol number P-2017-08-009). Research was conducted according to the Guide for the Care and Use of Laboratory Animals prepared by the Institute of Laboratory Animal Resources, National Research Council, US National Academy of Sciences^58^.

### Experimental design

There were six groups of mice (16 mice per group) for this metabolomics study. Groups 1 and 2 received amifostine (50 or 200 mg/kg) and were not irradiated. Group 3 received vehicle (saline) and was also not irradiated. Group 4 received saline and was irradiated at the LD_90/30_ dose (9.6 Gy, 0.6 Gy/min). Groups 5 and 6 received a single dose of amifostine (50 or 200 mg/kg) and were irradiated at 9.6 Gy, 30 min (±10 min) after amifostine injection. Each animal was identified with 1, 2, 3, or 4 bands marked on their tails. Tissue samples were collected on days 4 and 9 post-irradiation (for each group, eight mice were sacrificed on day 4 and the other eight were sacrificed on day 9). There were an additional three groups of irradiated animals set aside for a survival study: these groups included one pretreated with vehicle alone, one pretreated with 50 mg/kg amifostine and one pretreated with 200 mg/kg amifostine. These irradiated groups of mice served to compare survival benefits of low versus high doses of amifostine. The treatment schedule for the survival study was exactly as mentioned above for metabolomics arm of the study.

### Drug preparation and administration for mice

Pharmaceutical grade amifostine (Ethyol) was purchased from Cumberland Pharmaceuticals (Nashville, TN, USA) as 500 mg sterile lyophilized powder vial, and reconstituted with normal saline (0.9% sodium chloride) prior to use. A dose of 50 or 200 mg/kg was injected subcutaneously at the nape of the neck in 0.1 ml volume, approximately 30 min (±10 min) prior to irradiation. Control groups received equivalent volume of normal saline

### Radiation exposure

Mice were placed in ventilated Plexiglas boxes compartmentalized to accommodate four mice per box and exposed to bilateral radiation in ^60^Co facility at a dose rate of 0.6 Gy/min. Animals were irradiated with a midline dose of 9.6 Gy (with an estimated LD_50/30_ dose of 8.6 Gy for CD2F1 mice). After irradiation, mice were returned to their cages and monitored for 30 days post-irradiation or until samples were collected. Radiation dosimetry was based primarily on the alanine/EPR (electron paramagnetic resonance) system^29,59^, currently accepted as one of the most accurate methods for relatively high radiation doses and used for inter-comparison between national metrology institutions. The calibration curves (spectrometer e-Scan, Burker Biospin, Inc., Madison, WI, USA) used in dose measurements are based on standard alanine calibration sets purchased from the US National Institute of Standards and Technology (NIST, Gaithersburg, MD, USA). The alanine dosimeters obtained from NIST were calibrated in terms of absorbed dose to water using the US national standard radiation sources. Identical alanine dosimeters were placed midline within mouse phantoms (Plexiglas 1″ diameter, 3″ length) and irradiated for predefined periods of time. Measurement of their EPR signals using the calibration curve constructed with alanine dosimeters from NIST provided dose rates to water in the core bodies of mice. A small correction was subsequently applied for the difference in mass energy absorption coefficients between water and soft tissue.

### Tissue sample collection

Tissue collections were performed on study days 4 and 9 post-irradiation. Mice were anesthetized with 1–5% isoflurane (Abbott Laboratories, Chicago, IL, USA) and then euthanized. The femur bones, jejunum, and lung were collected from each mouse and flash frozen in liquid nitrogen and then transferred to −80 °C. For bone marrow cell collection, both femur bones were excised using surgical scissors and forceps, and then cleaned of excess muscle tissue. The bones were immersed in 2 ml of cold Hank’s buffered salt solution (HBSS, Invitrogen, Carlsbad, CA, USA) and were kept on ice. Using a mortar and pestle, the femurs were crushed in 2 ml of cold HBSS. The cell suspension was then filtered into another sterile tube using a 100 μm nylon mesh (ELCO Filtering Company, Fort Lauderdale, FL, USA) and then centrifuged at 400 × g, 4 °C for 10 min. Red blood cells were lysed using 1 ml of lysing buffer (Invitrogen). After adding the lysing buffer, the cell suspension was allowed to sit for five minutes at room temperature, and washed twice with phosphate buffered saline (PBS, Invitrogen, Carlsbad, CA, USA) and then centrifuged at 400 × g at room temperature for five minutes. Tissue samples and bone marrow cells were stored at −70 °C until shipped on dry ice to Georgetown University Medical Center (Washington, DC).

### Tissue metabolomics using UPLC-ESI-QTOF-MS

A total of 150 μL of chilled 35% water, 25% methanol and 40% isopropanol containing internal standards (debrisoquine and 4-nitrobenzoic acid) was added to 5 mg of tissue. Samples were homogenized on ice and 150 μL of chilled acetonitrile was added to each. Next, the samples were vortexed and incubated at −20 °C for 30 min. Finally, the samples were centrifuged at 17,968 × g for 15 min at 4 °C. The supernatant of each sample was transferred to MS vials for data acquisition. The sample queue was randomized to avoid bias. Each sample (1 μL) was injected to a 1.7 μm, 2.1 × 50 mm Acquity BEH C18 column (Waters Corporation, Milford, MA) using an Acquity UPLC system connected to an electrospray ion source coupled with a quadrupole time-of-flight mass spectrometer (ESI-Q-TOF, Xevo-G2S, Waters Corporation, Milford, MA) operating in positive and negative ionization mode. The data were acquired in centroid TOF-MS mode over a mass range from 50 to 1,200 m/z. Positive mode had a capillary voltage of 3.0 kV, a sampling cone voltage of 30 V, and a source offset of 80 V. Negative mode had a capillary voltage of 2.75 kV, a sampling cone voltage of 20 V, and a source offset of 80 V. The desolvation gas flow was 600 L/h. and the temperature was set to 500 °C. The cone gas flow was 25 L/h, and the source temperature was 100 °C. The data was acquired in the sensitivity MS Mode with a scan time of 0.1 seconds, and inter-scan delay at 0.08 seconds. Real-time mass correction was applied using a solution of Leucine-Enkephalin (0.1 ng/ml) [M+H]^+^ (m/z 556.2771), [M−H] (m/z 554.2615) in 500 ml 50:50 acetonitrile/water and 250 μL formic acid at an infusion rate of 10 μL/min utilizing the Waters Lockspray® interface. Before and after samples run, a mixture of six standards (acetaminophen: m/z 152.0712 [M+H]^+^/150.0555 [M−H]^−^, sulfaguanidine: m/z 215.0603 [M+ H]^+^/213.0446 [M−H]^−^, sulfadimethoxine: m/z 311.0814 [M+H]^+^/309.0658 [M−H]^−^, Val-Tyr-Val: m/z 380.2185 [M+H]^+^/378.2029 [M−H]^−^, terfenadine: m/z 472.3216 [M+H]^+^ and leucine-enkephalin: m/z 556.2771 [M+H]^+^/554.2615 [M−H]^−^) were run to ensure mass accuracy during batch acquisition. The quality control (QC) samples for each tissue type consisted of a pooled aliquot of all samples in that study set, thus represents all metabolites in each matrix. The column was conditioned using the pooled QC sample and was injected periodically (after every 10 sample injections) to monitor mass accuracy, shifts in retention time and signal intensities for reproducibility and data quality of the LC-MS data^60^. The overlap of QC sample chromatograms (base peak intensity) shows minimal shifts in retention time and consistency in peak intensities throughout the acquisition (detailed in Supplementary Fig. 1).

### Statistical analysis of metabolomics data

The abundance measurements for metabolites (with a specific mass/charge ratio, and retention time) in both positive and negative modes were expressed as intensity units that were initially normalized to internal standards and total protein concentration. Mass search to assign putative metabolite identifications was performed using an in-house CEU Mass Mediator RESTful API service, which searched Kegg, HMDB, LipidMaps, Metlin, and PubChem which is consumed in the “cmmr” R package. Normalized LC-MS data was log transformed and Pareto scaled. For the 96 samples in the study set, the level of differential expression for each metabolite was calculated using an unpaired t-test, comparing vehicle versus amifostine 50 mg/kg (side effect of drug), vehicle versus amifostine 200 mg/kg (side effect of drug), vehicle and radiation (effect of radiation), radiation vs radiation + amifostine 50 mg/kg or 200 mg/kg (effect of drug), constrained by FDR adjusted p-value < 0.05. The effect of the higher dose of the drug was done by ANOVA among sham only, radiation only, radiation with amifostine 50 mg/kg and 200 mg/kg groups constrained by FDR <0.05.

The identities of all significantly dysregulated metabolites were confirmed using tandem mass spectrometry. Validation results were produced using the TandemQuery tool (Li *et al*., unpublished), an in-house developed R package “msmsr” (Li *et al*., unpublished) and the NIST 2017 MS/MS spectra database. The fragmentation information of the validated metabolites that were significantly dysregulated for different comparisons was included in Supplementary Table 1.

To evaluate the metabolic pathways for radiation and the effect of amifostine, we used Mummichog v2.0, a Python package specifically designed for untargeted metabolomics^37^ which has gained increasing popularity^61–63^. Mummichog v2.0 tests pathway enrichment patterns using permutations and computes the probability for involvement in each pathway.

## Supplementary information

Supplementary information

## Data Availability

All data generated or analyzed during this study are included in this published article (and its Supplementary Information Files).
