# Supplementary material for: Metabolomic studies in tissues of mice treated with amifostine and exposed to gamma-radiation
Source: Sci Rep. 2019 Oct 30;9:15701. doi: 10.1038/s41598-019-52120-w (PMC6821891; doi:10.1038/s41598-019-52120-w)
Supplement: Supplementary file 1 — Supplementary information [file 41598_2019_52120_MOESM1_ESM.pdf]

Metabolomic studies in tissues of mice treated with amifostine and exposed to gamma-radiation

Amrita K. Cheema, Yaoxiang Li, Michael Girgis, Meth Jayatilake, Madison Simas, Stephen Y. Wise, Ayodele O. Olabisi, Thomas M. Seed, Vijay K. Singh

Supplementary Figure 1. Data quality monitoring with blank injections and standard compound mixture that shows mass accuracy within 2 ppm. QC overlays show minimum shifts in retention time and changing intensities.

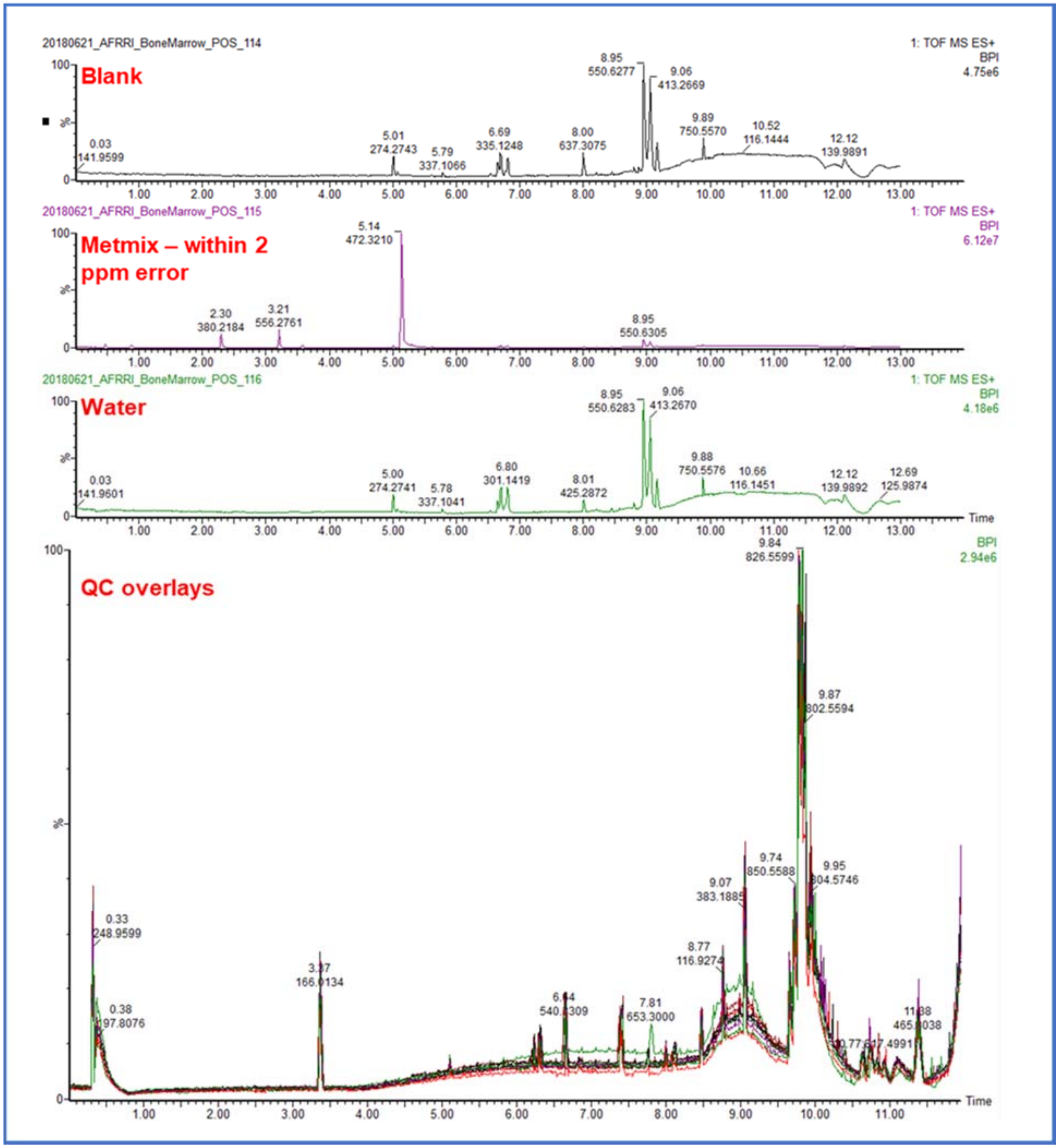
20180621_AFRRI_BoneMarrow_POS_114
1: TOF MS ES+
BPI
4.75e6
Blank
20180621_AFRRI_BoneMarrow_POS_115
1: TOF MS ES+
BPI
6.12e7
Metmix – within 2 ppm error
20180621_AFRRI_BoneMarrow_POS_116
1: TOF MS ES+
BPI
4.18e6
Water
Time
BPI
2.94e6
QC overlays
Time

Supplementary Figure 2. PLS-DA plots showing metabolomics-based separation of different study groups in bone marrow positive mode at day 4 (panel a – ESI positive and panel c – ESI negative) and day 9 (panels b and d) respectively.

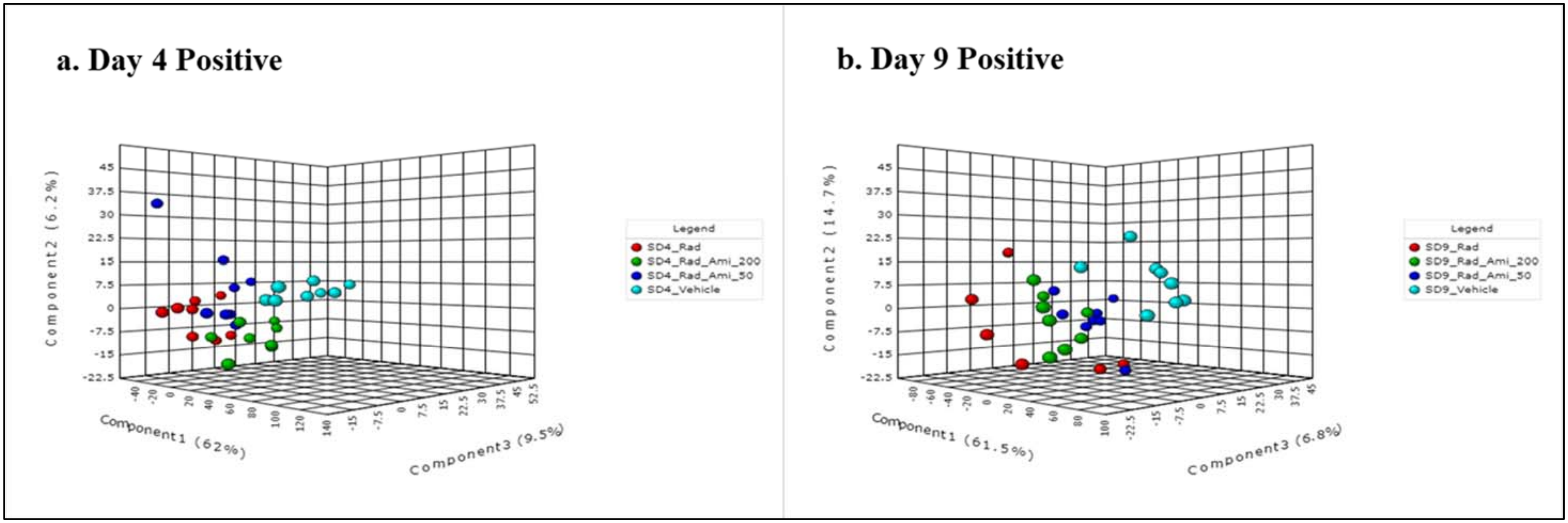
a. Day 4 Positive
Component2 (6.2%)
Component1 (62%)
Component3 (9.5%)
Legend
SD4_Rad
SD4_Rad_Ami_200
SD4_Rad_Ami_50
SD4_Vehicle
b. Day 9 Positive
Component2 (14.7%)
Component1 (61.5%)
Component3 (6.8%)
Legend
SD9_Rad
SD9_Rad_Ami_200
SD9_Rad_Ami_50
SD9_Vehicle

Supplementary Figure 3. PLS-DA plots showing metabolomics-based separation of different study groups in jejunum (radiation only, sham only, radiation + amifostine 50 mg/kg and 200 mg/kg) at day 4 (panel a – ESI positive and panel c- ESI negative) and day 9 (panels b and d), respectively.

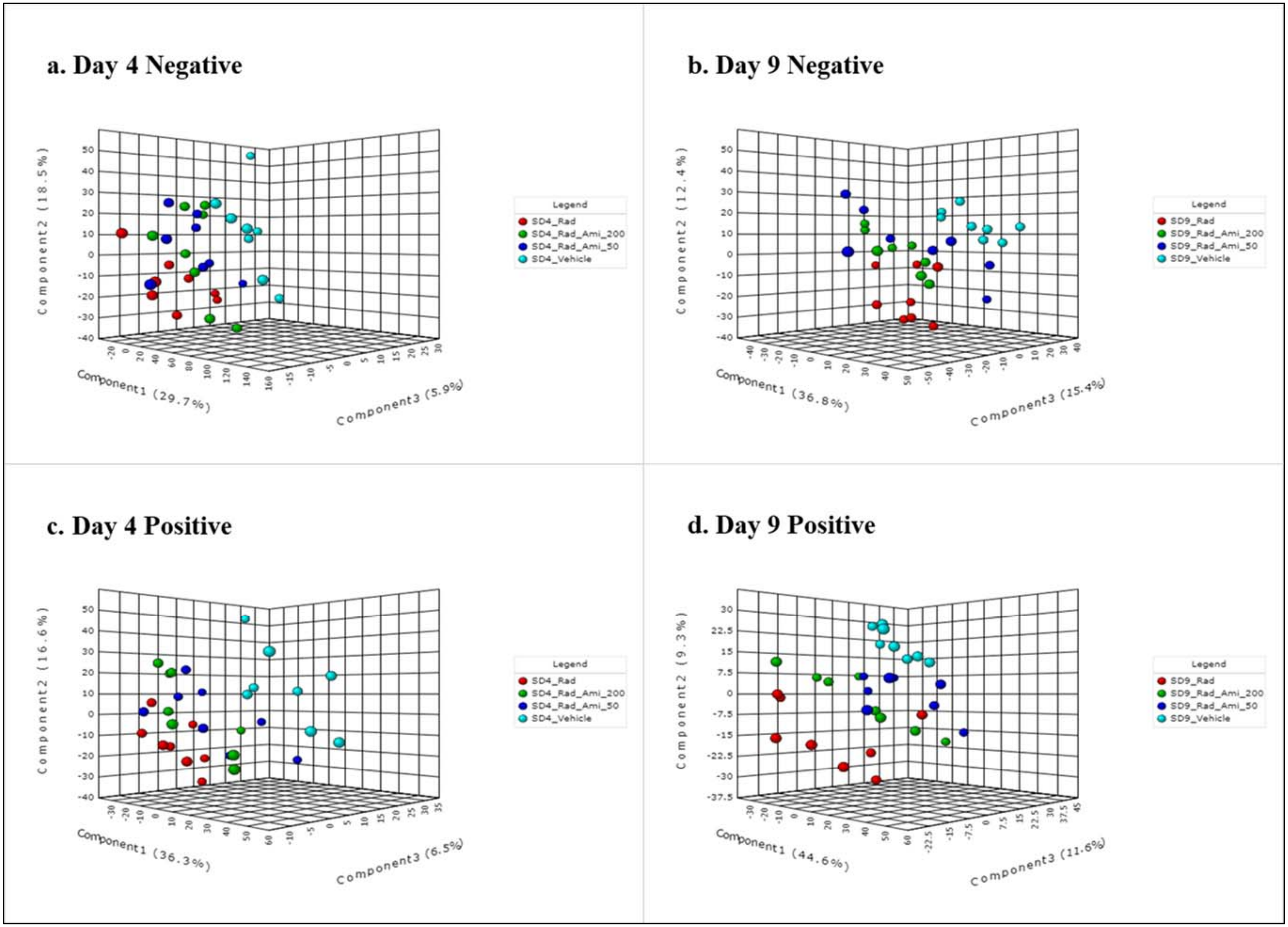
a. Day 4 Negative
Component2 (18.5%)
Component1 (29.7%)
Component3 (5.9%)
Legend
SD4_Rad
SD4_Rad_Ami_200
SD4_Rad_Ami_50
SD4_Vehicle
b. Day 9 Negative
Component2 (12.4%)
Component1 (36.8%)
Component3 (15.4%)
Legend
SD9_Rad
SD9_Rad_Ami_200
SD9_Rad_Ami_50
SD9_Vehicle
c. Day 4 Positive
Component2 (16.6%)
Component1 (36.3%)
Component3 (6.5%)
Legend
SD4_Rad
SD4_Rad_Ami_200
SD4_Rad_Ami_50
SD4_Vehicle
d. Day 9 Positive
Component2 (9.3%)
Component1 (44.6%)
Component3 (11.6%)
Legend
SD9_Rad
SD9_Rad_Ami_200
SD9_Rad_Ami_50
SD9_Vehicle

Supplementary Figure 4. PLS-DA plots showing metabolomics-based separation in lung of different study groups (radiation only, sham only, radiation + amifostine 50 mg/kg and 200 mg/kg) at day 4 (panel a – ESI positive and panel c- ESI negative) and day 9 (panels b and d), respectively.

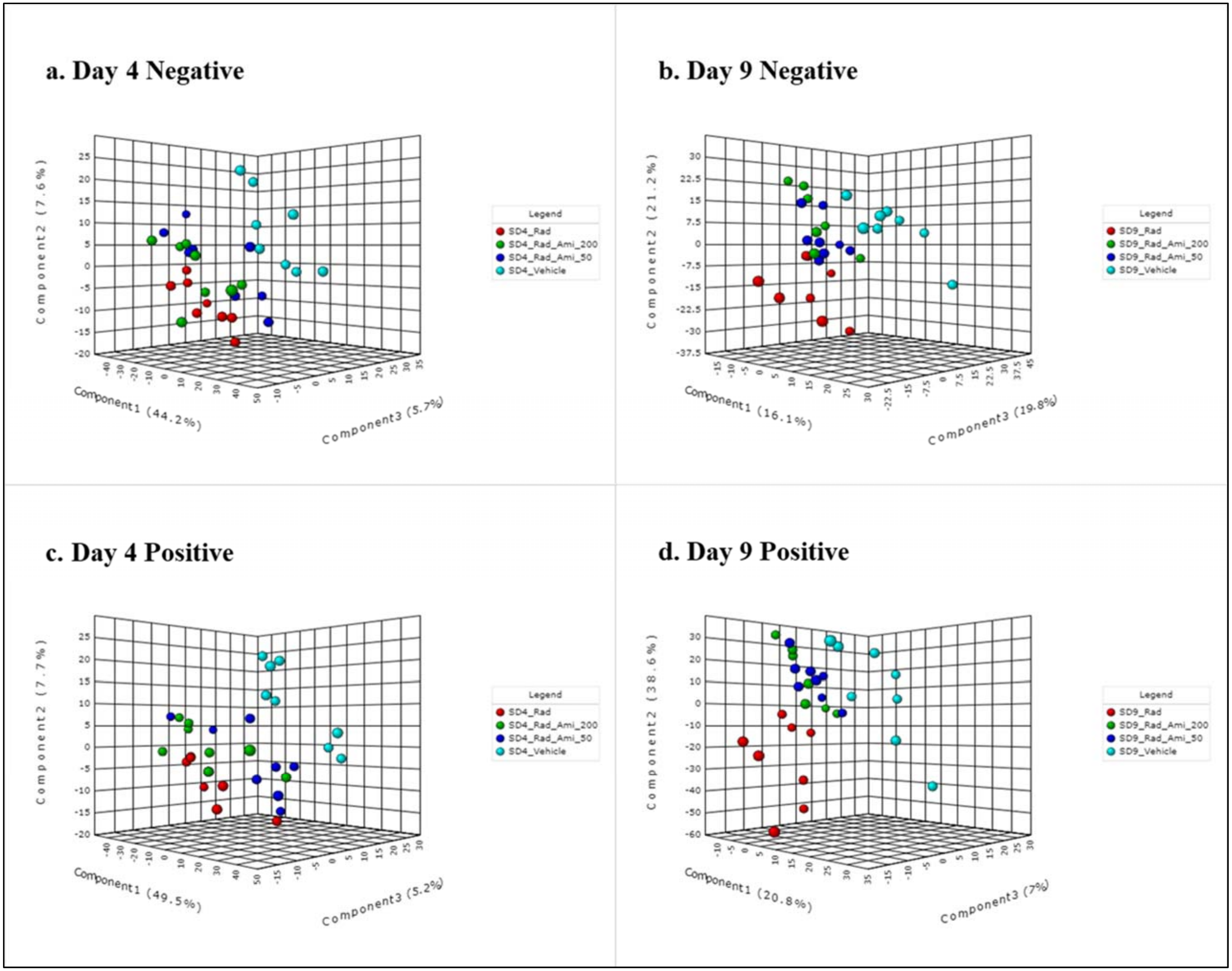
a. Day 4 Negative
Component2 (7.6%)
Component1 (44.2%)
Component3 (5.7%)
Legend
SD4_Rad
SD4_Rad_Ami_200
SD4_Rad_Ami_50
SD4_Vehicle
b. Day 9 Negative
Component2 (21.2%)
Component1 (16.1%)
Component3 (19.8%)
Legend
SD9_Rad
SD9_Rad_Ami_200
SD9_Rad_Ami_50
SD9_Vehicle
c. Day 4 Positive
Component2 (7.7%)
Component1 (49.5%)
Component3 (5.2%)
Legend
SD4_Rad
SD4_Rad_Ami_200
SD4_Rad_Ami_50
SD4_Vehicle
d. Day 9 Positive
Component2 (38.6%)
Component1 (20.8%)
Component3 (7%)
Legend
SD9_Rad
SD9_Rad_Ami_200
SD9_Rad_Ami_50
SD9_Vehicle

Supplementary Figure 5. Volcano plot of radiation dysregulated metabolites for jejunum at day 4 and day 9 (positive and negative). All marked names are tandem mass spectrometry validated metabolites which FDR significant (FDR adjusted p-value < 0.05) comparing radiation versus vehicle.

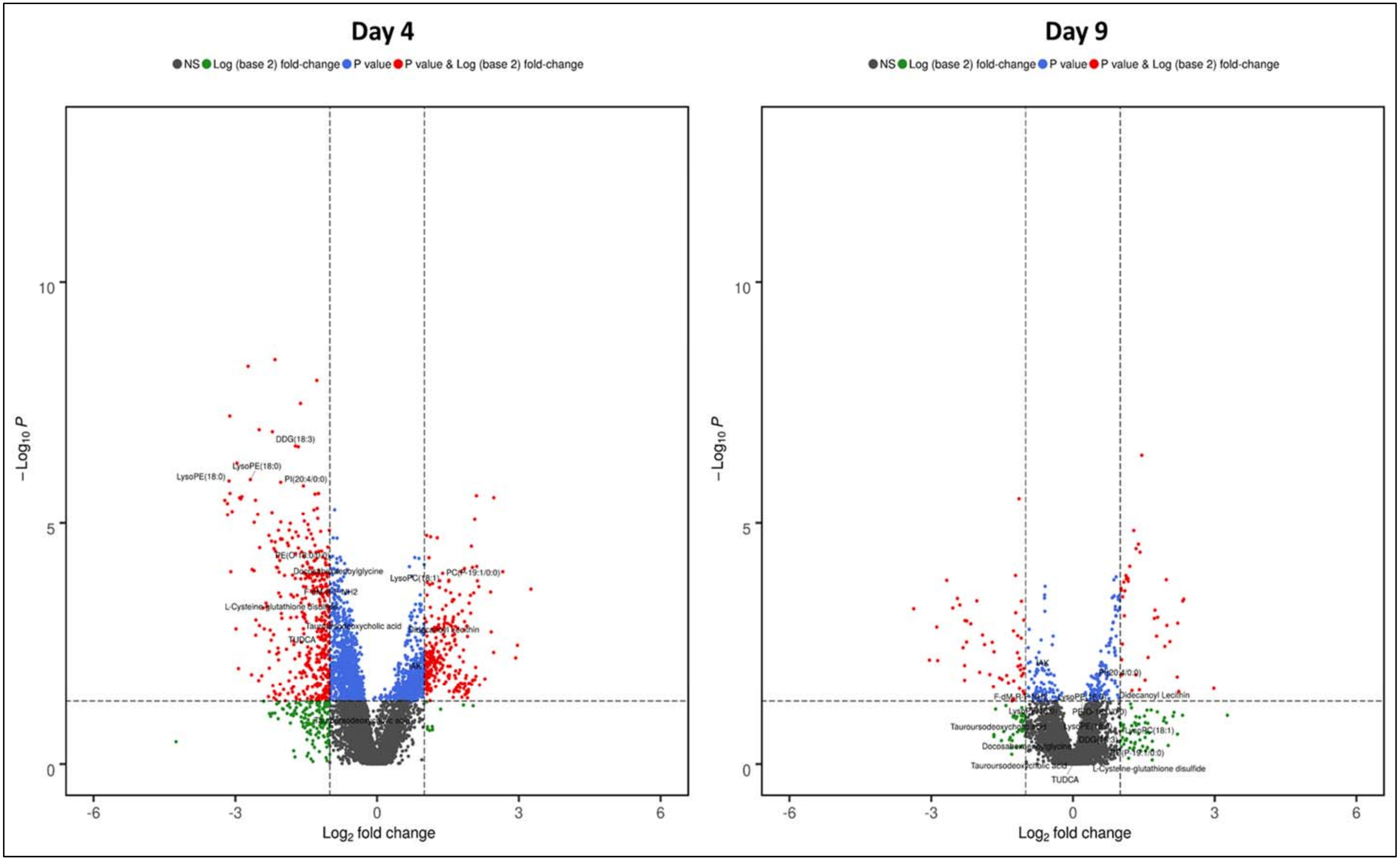

Day 4
Day 9
NS
Log (base 2) fold-change
P value
P value & Log (base 2) fold-change
−Log₁₀ P
Log₂ fold change
DDG(18:3)
LysoPE(18:0)
PI(20:4/0:0)
L-Cysteine-glutathione disulfide
TUDCA
Tauroursodeoxycholic acid
PC(P-19:1/0:0)
LysoPC(18:1)
Didecanoyl Lecithin
AK
Docosahexaenoylglycine

Supplementary Figure 6. Volcano plot of radiation dysregulated metabolites for lung at day 4 and day 9 (positive and negative). All labeled names are tandem mass spectrometry validated metabolites which FDR significant (FDR adjusted p-value < 0.05) comparing radiation versus vehicle.

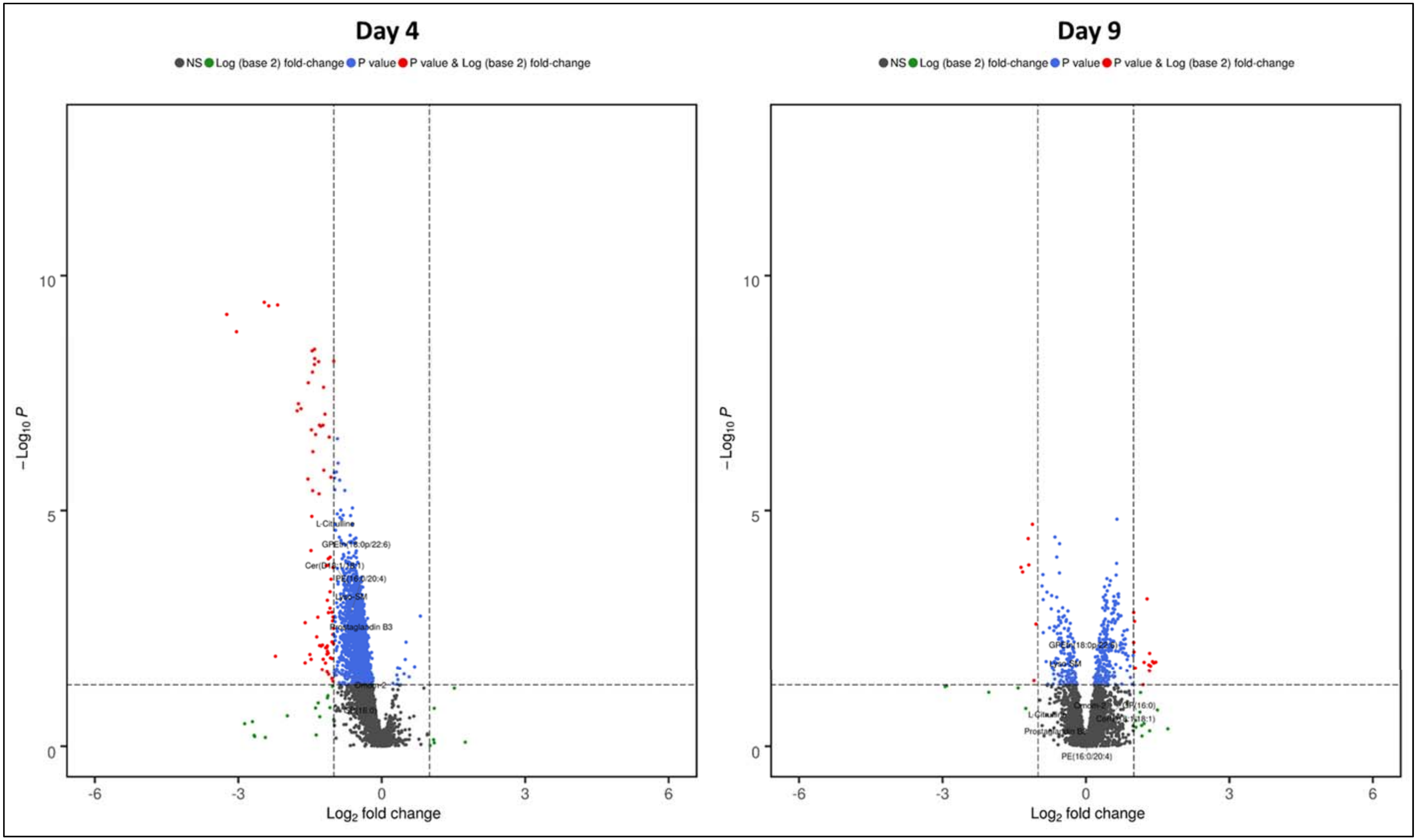

Day 4
NS Log (base 2) fold-change P value P value & Log (base 2) fold-change
−Log10 P
Log2 fold change
L-Citrulline
GPEtn(18:0p/22:6)
PE(16:0/20:4)
Lyso-SM
Prostaglandin B3
Omdm-2
Day 9
NS Log (base 2) fold-change P value P value & Log (base 2) fold-change
−Log10 P
Log2 fold change
PE(16:0/20:4)

Supplementary Figure 7. Rain plot for all validated recovered metabolites in bone marrow day 4/day 9, 50 mg/200 mg.

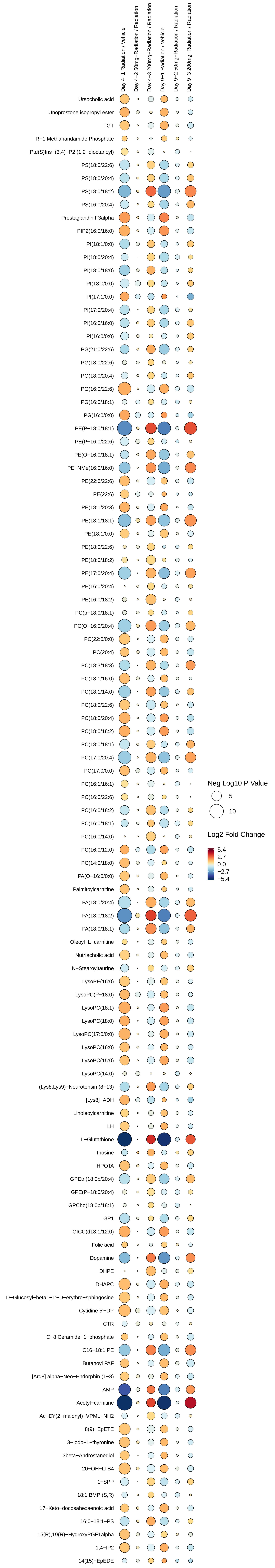

Day 4−1 Radiation / Vehicle
Day 4−2 50mg+Radiation / Radiation
Day 4−3 200mg+Radiation / Radiation
Day 9−1 Radiation / Vehicle
Day 9−2 50mg+Radiation / Radiation
Day 9−3 200mg+Radiation / Radiation
Ursocholic acid
Unoprostone isopropyl ester
TGT
R−1 Methanandamide Phosphate
Ptd(S)Ins−(3,4)−P2 (1,2−dioctanoyl)
PS(18:0/22:6)
PS(18:0/20:4)
PS(18:0/18:2)
PS(16:0/20:4)
Prostaglandin F3alpha
PIP2(16:0/16:0)
PI(18:1/0:0)
PI(18:0/20:4)
PI(18:0/18:0)
PI(18:0/0:0)
PI(17:1/0:0)
PI(17:0/20:4)
PI(16:0/16:0)
PI(16:0/0:0)
PG(21:0/22:6)
PG(18:0/22:6)
PG(18:0/20:4)
PG(16:0/22:6)
PG(16:0/18:1)
PG(16:0/0:0)
PE(P−18:0/18:1)
PE(P−16:0/22:6)
PE(O−16:0/18:1)
PE−NMe(16:0/16:0)
PE(22:6/22:6)
PE(22:6)
PE(18:1/20:3)
PE(18:1/18:1)
PE(18:1/0:0)
PE(18:0/22:6)
PE(18:0/18:2)
PE(17:0/20:4)
PE(16:0/20:4)
PE(16:0/18:2)
PC(p−18:0/18:1)
PC(O−16:0/20:4)
PC(22:0/0:0)
PC(20:4)
PC(18:3/18:3)
PC(18:1/16:0)
PC(18:1/14:0)
PC(18:0/22:6)
PC(18:0/20:4)
PC(18:0/18:2)
PC(18:0/18:1)
PC(17:0/20:4)
PC(17:0/0:0)
PC(16:1/16:1)
PC(16:0/22:6)
PC(16:0/18:2)
PC(16:0/18:1)
PC(16:0/14:0)
PC(16:0/12:0)
PC(14:0/18:0)
PA(O−16:0/0:0)
Palmitoylcarnitine
PA(18:0/20:4)
PA(18:0/18:2)
PA(18:0/18:1)
Oleoyl−L−carnitine
Nutriacholic acid
N−Stearoyltaurine
LysoPE(16:0)
LysoPC(P−18:0)
LysoPC(18:1)
LysoPC(18:0)
LysoPC(17:0/0:0)
LysoPC(16:0)
LysoPC(15:0)
LysoPC(14:0)
(Lys8,Lys9)−Neurotensin (8−13)
[Lys8]−ADH
Linoleoylcarnitine
LH
L−Glutathione
Inosine
HPOTA
GPEtn(18:0p/20:4)
GPE(P−18:0/20:4)
GPCho(18:0p/18:1)
GP1
GlCC(d18:1/12:0)
Folic acid
Dopamine
DHPE
DHAPC
D−Glucosyl−beta1−1'−D−erythro−sphingosine
Cytidine 5'−DP
CTR
C−8 Ceramide−1−phosphate
C16−18:1 PE
Butanoyl PAF
[Arg8] alpha−Neo−Endorphin (1−8)
AMP
Acetyl−carnitine
Ac−DY(2−malonyl)−VPML−NH2
8(9)−EpETE
3−Iodo−L−thyronine
3beta−Androstanediol
20−OH−LTB4
1−SPP
18:1 BMP (S,R)
17−Keto−docosahexaenoic acid
16:0−18:1−PS
15(R),19(R)−HydroxyPGF1alpha
1,4−IP2
14(15)−EpEDE
Neg Log10 P Value
5
10
Log2 Fold Change
5.4
2.7
0.0
−2.7
−5.4

Supplementary Table 1
Full list of tandem MS validated metabolites and details

Name	Synonyms	Level	Confidence[a]	CID
1-Stearoyl-2-linoleoyl-sn-glycero-3-phosphate	PA(18:0/18:2)	2.1	B	698.51, 436.28, 418.28
1-(1Z-Octadecenyl)-2-(9Z-octadecenoyl)-sn-glycero-3-phosphoethanolamine	PE(P-18:0/18:1)	2.1	A	728.58, 279.23
1-Hexadecyl-2-(5Z,8Z,11Z,14Z-eicosatetraenoyl)-sn-glycero-3-phosphoethanolamine	PC(O-16:0/20:4)	2.1	B	725.51, 303.23
1-(1Z-Octadecenyl)-2-(9Z-octadecenoyl)-sn-glycero-3-phosphoethanolamine	PE(P-18:0/18:1)	2.1	A	727.57, 279.23
1,2-Di-(9Z,12Z,15Z-octadecatrienoyl)-sn-glycero-3-phosphocholine	PC(18:3/18:3)	2.1	B	813.51, 279.23
1,2-Dihexadecanoyl-sn-glycero-3-phospho-N-methylethanolamine	PE-NMe(16:0/16:0)	2.1	B	704.57, 466.32, 255.23
1-(1Z-Octadecenyl)-2-(9Z-octadecenoyl)-sn-glycero-3-phosphoethanolamine	PE(P-18:0/18:1)	2.1	B	729.57, 466.32, 279.23
1-Heptadecanoyl, 2-(5Z,8Z,11Z,14Z-eicosatetraenoyl)-sn-glycero-3-phosphoethanolamine	PC(17:0/20:4)	2.1	B	753.54, 466.37, 279.23
1,2-Dioleoyl-sn-glycero-3-phosphoethanolamine-N-methyl	PE(18:1/18:1)	2.1	B	755.54, 279.23
1,2-Dipalmitoyl-sn-glycero-3-phosphoethanolamine	DHPE	2.1	B	689.56, 452.21, 255.23
1-Stearoyl-2-linoleoyl-sn-glycero-3-phospho-L-serine	PS(18:0/18:2)	2.1	B	699.51, 279.23
1-Stearoyl-2-docosahexaenoyl-sn-glycero-3-phosphocholine	PC(18:0/22:6)	2.1	C	869.57, 327.23, 303.28
1,2-Dihexadecanoyl-sn-glycero-3-phospho-N-methylethanolamine1,2-Dihexadecanoyl-sn-glycero-3-phospho-N-methylethanolamine	PE-NMe(16:0/16:0)	2.1	A	703.45, 281.24
1-Octadecanoyl-sn-glycero-3-phospho-(1'-myo-inositol)	PI(18:0/0:0)	2.1	A	599.32, 241.04, 152.99
1-Hexadecyl-2-(9Z-octadecenoyl)-sn-glycero-3-phosphoethanolamine	C16-18:1 PE	2.1	B	702.54, 281.17
1,2-Dioctadecanoyl-sn-glycero-3-phospho-(1'-myo-inositol)	PI(18:0/18:0)	2.1	C	865.5
1-Octadecanoyl-2-(5Z,8Z,11Z,14Z-eicosatetraenoyl)-sn-glycero-3-phosphate	PA(18:0/20:4)	2.1	B	724.52, 303.23, 279.28
1-Palmitoyl-2-linoleoyl-sn-glycero-3-phosphocholine	PC(16:0/18:2)	2.1	A	801.54, 742.59, 279.23
1,2-Didocosahexaenoyl-sn-glycero-3-phosphocholine	DHAPC	2.1	B	922.56, 862.54, 327.23
1-(1Z-Octadecenyl)-2-(9Z-octadecenoyl)-sn-glycero-3-phosphocholine	PC(18:0/18:1)	2.1	B	814.53, 754.57
1-(9Z-Octadecenoyl)-sn-glycero-3-phospho-(1'-myo-inositol)	PI(18:1/0:0)	2.1	B	597.32, 281.24
1-Stearoyl-2-linoleoyl-sn-glycero-3-phosphocholine	PC(18:0/18:2)	2.1	C	819.52, 281.24
1-Stearoyl-2-arachidonyl-sn-glycero-3-phosphocholine	PC(18:0/20:4)	2.1	B	844.48, 788.52, 303.23
(Lys8,Lys9)-Neurotensin (8-13)	(Lys8,Lys9)-Neurotensin (8-13)	2.1	C	758.5
1-Stearoyl-2-docosahexaenoyl-sn-glycero-3-phosphocholine	PC(18:0/22:6)	2.1	C	868.54, 327.23, 303.23
1-Stearoyl-2-docosahexaenoyl-sn-glycero-3-phosphocholine	PC(18:0/22:6)	2.1	B	867.54, 808.54, 283.24
1,2-Dioctadecanoyl-sn-glycero-3-phospho-(1'-myo-inositol)	PI(18:0/18:0)	2.1	C	864.55, 806.5
1-Hexadecyl-2-(9Z-octadecenoyl)-sn-glycero-3-phosphoethanolamine	PE(O-16:0/18:1)	2.1	B	701.56, 279.23
1-Heptadecanoyl, 2-(5Z,8Z,11Z,14Z-eicosatetraenoyl)-sn-glycero-3-phosphoethanolamine	PE(17:0/20:4)	2.1	B	752.55, 466.31, 303.23
1,2-Didocosahexaenoyl-sn-glycero-3-phosphocholine	DHAPC	2.1	B	923.55, 864.5, 327.23, 283.3
2-Oleoyl-1-palmitoyl-sn-glycero-3-phosphoserine	16:0-18:1-PS	2.1	B	759.55, 673.48, 391.22
1-Oleoyl-2-myristoyl-sn-glycero-3-phosphocholine	PC(18:1/14:0)	2.1	C	775.55
2-Linoleoyl-1-palmitoyl-sn-glycero-3-phosphoethanolamine	PE(16:0/18:2)	2.1	A	714.5, 279.23
1-Heptadecanoyl-2-(5Z,8Z,11Z,14Z-eicosatetraenoyl)-sn-glycero-3-phosphocholine	PC(17:0/20:4)	2.1	C	829.52
1-Stearoyl-2-linoleoyl-sn-glycero-3-phosphoethanolamine	PE(18:0/18:2)	2.1	B	742.53, 279.23
1-Palmitoyl-2-lauroyl-sn-glycero-3-phosphorylcholine	PC(16:0/12:0)	2.1	B	711.48, 647.48
1,2-Di(4Z,7Z,10Z,13Z,16Z,19Z-docosahexaenoyl)-sn-glycero-3-phosphoethanolamine	PE(22:6/22:6)	2.1	B	835.53, 327.23
1-O-(1Z-Hexadecenyl)-2-(4Z,7Z,10Z,13Z,16Z,19Z-docosahexaenoyl)-sn-glycero-3-phosphoethanolamine	PE(P-16:0/22:6)	2.1	B	746552, 436.2
2-Oleoyl-1-palmitoyl-sn-glycero-3-phosphocholine	PC(16:0/18:1)	2.1	B	794.55
1-Stearoyl-2-docosahexaenoyl-sn-glycero-3-phospho-(1'-sn-glycerol)	PG(18:0/22:6)	2.1	B	820.58, 152.98
1-Heneicosanoyl-2-(4Z,7Z,10Z,13Z,16Z,19Z-docosahexenoyl)-sn-glycero-3-phospho-(1'-rac-glycerol)	PG(21:0/22:6)	2.1	B	863.59
1-Palmitoyl-2-myristoyl-sn-glycero-3-phosphocholine	PC(16:0/14:0)	2.1	C	740.56, 255.55
1-Octadecanoyl-2-(9Z-octadecenoyl)-sn-glycero-3-phosphate	PA(18:0/18:1)	2.1	B	700.54, 631.13, 418.27, 279.23
D-myo-Inositol-1,4-diphosphate	1,4-IP2	2.1	B	677.56, 418.94, 339.32
Prostaglandin F3α	Prostaglandin F3alpha	2.1	B	351.28, , 289.16
N-(Dodecanoyl)-1-β-glucosyl-sphing-4-enine	GlCC(d18:1/12:0)	2.1	B	643.49, 380.04
1-(1,2R-Dihexadecanoylphosphatidyl)inositol-3,4-bisphosphate	PIP2(16:0/16:0)	2.1	B	483.28,
9-Hydroperoxy-10E,12Z,15Z-octadecatrienoic acid	HPOTA	2.1	A	310.17, 171.08, 96.98
1-Stearoyl-2-docosahexaenoyl-sn-glycero-3-phosphocholine	PC(18:0/22:6)	2.1	B	878.58, 818.58, 327.26, 283.25
1-Oleoyl-sn-glycero-3-phosphoethanolamine	PE(18:1/0:0)	2.1	B	477.28, 283.01, 196.07
1-Palmitoyl-2-docosahexaenoyl-sn-glycero-3-phospho-(1'-rac-glycerol)	PG(16:0/22:6)	2.1	B	795.51, 255.22
1-Behenoyl-2-hydroxy-sn-glycero-3-phosphocholine	PC(22:0/0:0)	2.1	C	813.39
1-Stearoyl-2-docosahexaenoyl-sn-glycero-3-phospho-(1'-sn-glycerol)	PG(18:0/22:6)	2.1	C	822.56, 766.35, 303.23
1-Stearoyl-2-arachidonoyl-sn-glycero-3-phospho-(1'-sn-glycerol)	PG(18:0/20:4)	2.1	B	798.52, 279.23
8(9)-Epoxy-5Z,11Z,14Z,17Z-eicosatetraenoic acid	8(9)-EpETE	2.1	B	298.98, 255.32
1-(1Z-Octadecenyl)-2-(4Z,7Z,10Z,13Z,16Z,19Z-docosahexaenoyl)-sn-glycero-3-phosphocholine	PC(18:0/22:6)	2.1	B	861.58, 802.53, 327.24
1-Stearoyl-2-arachidonoyl-sn-glycero-3-phospho-(1'-sn-glycerol)	PG(18:0/20:4)	2.1	B	797.52, 311.36, 303.22
1-(1Z-Octadecenyl)-2-(4Z,7Z,10Z,13Z,16Z,19Z-docosahexaenoyl)-sn-glycero-3-phosphoethanolamine	PE(18:0/22:6)	2.1	A	774.54, 464.3, 327.32, 281.24
1-(1Z-Octadecenyl)-2-(9Z-octadecenoyl)-sn-glycero-3-phosphocholine	PC(p-18:0/18:1)	2.1	B	817.21, 756.58, 281.28
1-Stearoyl-2-docosahexaenoyl-sn-glycero-3-phosphocholine	PC(18:0/22:6)	2.1	B	879.56, 322.27, 283.23
1-Stearoyl-2-linoleoyl-sn-glycero-3-phospho-L-serine	PS(18:0/18:2)	2.1	A	786.53, 699.49, 419.25, 283.25, 152.95
1,2-Diarachidonoyl-sn-glycero-3-phosphocholine	PC(20:4)	2.1	B	873.55, 814.56
D-Glucosamine 1-phosphate	GP1	2.1	C	518.38, 258.23
1-Stearoyl-2-hydroxy-sn-glycero-3-phosphocholine	LysoPC(18:0)	2.1	A	567.44, 508.38, 283.22
2-Arachidonoyl-1-palmitoyl-sn-glycero-3-phosphoethanolamine	PE(16:0/20:4)	2.1	A	737.5, 303.23, 140.01
1-Palmitoyl-2-hydroxy-sn-glycero-3-phosphoethanolamine	LysoPE(16:0)	2.1	A	451.28, 255.28, 196.04
8,11-Tridecadienoic acid, 13-(3-pentyl-2-oxiranyl)-, (8Z,11Z)-	14(15)-EpEDE	2.1	B	303.22, 126.96
3-Iodo-L-thyronine	3-Iodo-L-thyronine	2.1	C	380.28, 338.52, 25.27
1-Stearoyl-2-docosahexaenoyl-sn-glycero-3-phospho-(1'-sn-glycerol)	PG(18:0/22:6)	2.1	B	621.57, 281.25
1-Hexadecyl lysophosphatidic acid	PA(O-16:0/0:0)	2.1	C	377.24, 78.9
1-Palmitoyl-2-hydroxy-sn-glycero-3-phosphoethanolamine	LysoPE(16:0)	2.1	A	452.37, 255.22, 196.4
1-Stearoyl-2-linoleoyl-sn-glycero-3-phospho-L-serine	PS(18:0/18:2)	2.1	A	786.55, 699.28, 419.22, 283.23
1-(1Z-Octadecenyl)-2-(5Z,8Z,11Z,14Z-eicosatetraenoyl)-sn-glycero-3-phosphoethanolamine	GPEtn(18:0p/20:4)	2.1	A	751.56, 464.11303.22
1-Oleoyl-sn-glycero-3-phosphocholine	LysoPC(18:1)	2.1	A	565.34, 508.3, 283.24
1-(1Z-Octadecenyl)-2-(5Z,8Z,11Z,14Z-eicosatetraenoyl)-sn-glycero-3-phosphoethanolamine	GPEtn(18:0p/20:4)	2.1	A	750.51, 674, 464.2.303.23
1-Octadecanoyl-2-(5Z,8Z,11Z,14Z-eicosatetraenoyl)-sn-glycero-3-phosphate	PA(18:0/20:4)	2.1	B	723.52, 303.22
1-Palmitoyl-2-docosahexaenoyl-sn-glycero-3-phosphocholine	PC(16:0/22:6)	2.1	B	841.58, 814.51, 480.32, 279.32
1-Stearoyl-2-docosahexaenoyl-sn-glycero-3-phosphocholine	PC(18:0/22:6)	2.1	C	869.56, 818.57
17-Keto-4(Z),7(Z),10(Z),13(Z),15(E),19(Z)-docosahexaenoic acid	17-Keto-docosahexaenoic acid	2.1	B	341.32, 295.22
1-Pentadecanoyl-sn-glycero-3-phosphocholine	LysoPC(15:0)	2.1	B	525.37, 466.32
1-Heptadecanoyl-2-(5Z,8Z,11Z,14Z-eicosatetraenoyl)-sn-glycero-3-phospho-(1'-myo-inositol)	PI(17:0/20:4)	2.1	B	871.58, 403.7, 269.4
1-(1Z-Octadecenyl)-2-(9Z-octadecenoyl)-sn-glycero-3-phosphocholine	GPCho(18:0p/18:1)	2.1	A	815.25, 756.51, 279.23
Ac-Asp-Tyr(2-malonyl)-Val-Pro-Met-Leu-NH2	Ac-DY(2-malonyl)-VPML-NH2	2.1	B	878.61, 881.55, 832.5
1-Palmitoyl-2-docosahexaenoyl-sn-glycero-3-phosphocholine	PC(16:0/22:6)	2.1	A	851.57, 790.52, 480.4
1-Palmitoyl-2-oleoyl-phosphatidylglycerol	PG(16:0/18:1)	2.1	C	747.57
1-Palmitoyl-2-docosahexaenoyl-sn-glycero-3-phosphocholine	PC(16:0/22:6)	2.1	A	850.65, 790.5
1-Palmitoyl-2-docosahexaenoyl-sn-glycero-3-phospho-(1'-rac-glycerol)	PG(16:0/22:6)	2.1	C	792.55
N-Stearoyltaurine	N-Stearoyltaurine	2.1	B	390.21, 325.11, 79.96
1-(1Z-Octadecenyl)-2-(5Z,8Z,11Z,14Z-eicosatetraenoyl)-sn-glycero-3-phosphoethanolamine	GPEtn(18:0p/20:4)	2.1	B	751.56, 464.3, 303.23
1,2-Dipalmitoyl-sn-glycero-3-phospho-(1'-myo-inositol)	PI(16:0/16:0)	2.1	B	808.58, 152.99
1-Stearoyl-2-arachidonyl-sn-glycero-3-phosphocholine	PC(18:0/20:4)	2.1	B	843.54, 303.23
2-Docosahexaenoyl-1-stearoyl-sn-glycero-3-phosphoserine	PS(18:0/22:6)	2.1	B	833.51, 747.48, 419.29
[Lys8]-Vasopressin	[Lys8]-ADH	2.1	C	526.21
1-Stearoyl-2-arachidonoyl-sn-glycero-3-phospho-(1'-sn-glycerol)	PG(18:0/20:4)	2.1	B	796.56, 303.23
1-Stearoyl-2-arachidonoyl-sn-glycero-3-phosphoserine	PS(18:0/20:4)	2.1	A	809.58, 723.48, 419.25, 283.23, 152.98
1-Stearoyl-2-arachidonoyl-sn-glycero-3-phospho-(1'-myo-inositol)	PI(18:0/20:4)	2.1	A	885.54, 581.38, 241.01
2-Docosahexaenoyl-1-stearoyl-sn-glycero-3-phosphoethanolamine	PE(18:0/22:6)	2.1	B	791.56, 480.3, 283.23
1-(1Z-Octadecenyl)-2-(5Z,8Z,11Z,14Z-eicosatetraenoyl)-sn-glycero-3-phosphoethanolamine	GPE(P-18:0/20:4)	2.1	A	748.45, 463.08, 303.27
2-Arachidonoyl-1-palmitoyl-sn-glycero-3-phosphoethanolamine	PE(16:0/20:4)	2.1	B	738.59, 45.22, 303.24, 255.34
2-Arachidonoyl-1-palmitoyl-sn-glycero-3-phosphoethanolamine	PE(16:0/20:4)	2.1	C	739.55
2-Docosahexaenoyl-1-stearoyl-sn-glycero-3-phosphoethanolamine	PE(18:0/22:6)	2.1	A	790.56, 480.32
1-(10Z-Heptadecenoyl)-sn-glycero-3-phospho-(1'-myo-inositol)	PI(17:1/0:0)	2.1	C	582.34
1-Oleoyl-2-palmitoyl-sn-glycero-3-phosphocholine	PC(18:1/16:0)	2.1	B	805.58, 744.54
15(R),19(R)-Hydroxyprostaglandin F1α	15(R),19(R)-HydroxyPGF1alpha	2.1	B	370.1, 357.8, 327.21
Tyr-Leu-Pro-Leu-Arg-Phe-NH2	PC(18:1/16:0)	2.1	C	804.58
1,2-Dipalmitoleoyl-sn-glycero-3-phosphocholine	PC(16:1/16:1)	2.1	C	763.57
1-Myristoyl-2-stearoyl-sn-glycero-3-phosphocholine	PC(14:0/18:0)	2.1	C	779.51, 718.58
1-Palmitoyl-2-arachidonoyl-sn-glycero-3-phosphoserine	PS(16:0/20:4)	2.1	B	782.48, 699.49, 283.25, 152.98
1-Stearoyl-2-arachidonoyl-sn-glycero-3-phosphoserine	PS(18:0/20:4)	2.1	A	810.58, 723.49, 419.23, 483.32, 152.99
1-(1-2R-Phosphothiotidyl)inositol-3,4-bisphosphate	Ptd(S)Ins-(3,4)-P2 (1,2-dioctanoyl)	2.1	C	762.58, 511.23, 78.99
Nis(monooleoylglycero)phosphate (S,R Isomer)	18:1 BMP (S,R)	2.1	C	774.56, 281.23
Thr-Gly-Thr	Thr-Gly-Thr	2.1	C	275.18, 231.88
N-(2-Phosphate-1R-methylethyl)-5Z,8Z,11Z,14Z-eicosatetraenamide	R-1 Methanandamide Phosphate	2.1	B	440.3, 239.3, 78.92
Folic acid	Folic acid	2.1	B	441.3, 311.07
1,2-Dioleoyl-sn-glycero-3-phospho-(1'-myo-inositol-3'-phosphate)	PIP(18:1)	2.1	C	470.18, 281.28

Thyroxine 4'-O-β-D-glucuronide	Thyroxine 4'-O-beta-D-glucuronide	2.1	C	474.28
1-Palmitoyl-2-hydroxy-sn-glycero-3-phospho-(1'-rac-glycerol)	PG(16:0/0:0)	2.1	A	482.27, 227.2, 152.99
N-Octanoylsphingosine-1-phosphate	C-8 Ceramide-1-phosphate	2.1	B	486.28, 78.05
1-Hexadecanoyl-sn-glycero-3-phosphoserine	PS(16:0/18:0)	2.1	B	495.23, 409.27, 153
1,2-Di(4Z,7Z,10Z,13Z,16Z,19Z-docosahexaenoyl)-sn-glycero-3-phosphoethanolamine	PE(22:6)	2.1	B	524.27, 327.12, 283.1, 196.03
1-Heptadecanoyl-sn-glycero-3-phosphocholine	LysoPC(17:0/0:0)	2.1	B	555.9, 494.36, 405.29
1-Stearoyl-2-hydroxy-sn-glycero-3-phosphocholine	LysoPC(18:0)	2.1	A	568.15, 508.35, 283.25
1-Hexadecanoyl-sn-glycero-3-phospho-(1'-myo-inositol)	PI(16:0/0:0)	2.1	A	570.45, 255.26, 214.91
1-Hexadecanoyl-sn-glycero-3-phospho-(1'-myo-inositol)	PI(16:0/0:0)	2.1	A	571.28, 315.07, 255.23, 152.98
Dopamine	Dopamine	2.1	A	137.04, 119.07
D-erythro-Sphingosine-1-phosphate	1-SPP	2.1	B	361.24, 281.32
20-Hydroxyleukotriene B4	20-OH-LTB4	2.1	B	375.31, 337.91
Acetyl-DL-carnitine	Acetyl-carnitine	2.1	A	204.12, 145.05, 85.05
Luteinizing hormone releasing hormone, human	Luteinizing hormone	2.1	C	393.24
Palmitoylcarnitine	Palmitoylcarnitine	2.1	A	399.31, 341.23, 85.08
Palmitoylcarnitine	Palmitoylcarnitine	2.1	A	401.34, 341.28, 85.08
Linoleoylcarnitine	Linoleoylcarnitine	2.1	B	423.3, 324.23, 144.1, 85.04
Cytidine 5'-diphosphate	Cytidine 5'-DP	2.1	C	425.16
Oleoyl-L-carnitine	Oleoyl-L-carnitine	2.1	A	425.26, 367.28, 144.1, 85.02
Oleoyl-L-carnitine	Oleoyl-L-carnitine	2.1	A	425.26, 144.1, 85.04
Oleoyl-L-carnitine	Oleoyl-L-carnitine	2.1	A	425.38, 144.1, 85.04
Oleoyl-L-carnitine	Oleoyl-L-carnitine	2.1	A	426.36, 367.23, 144.1, 85.04
Oleoyl-L-carnitine	Oleoyl-L-carnitine	2.1	B	426.32, 144.1, 85.04
Cys-Tyr-Arg	Cys-Tyr-Arg	2.1	B	440.35, 266.59
Unoprostone isopropyl ester	Unoprostone isopropyl ester	2.1	B	448.28, 405.23
1-Palmitoyl-2-hydroxy-sn-glycero-3-phosphoethanolamine	LysoPE(16:0)	2.1	B	454.32, 313.31, 216.09, 155.05,
D-Glucosyl-β1-1'-D-erythro-sphingosine	-Glucosyl-beta1-1'-D-erythro-sphingos	2.1	C	462.31
1-Myristoyl-sn-glycero-3-phosphocholine	LysoPC(14:0)	2.1	A	467.28, 184.07
1-Palmitoyl-sn-glycero-3-phosphocholine	LysoPC(16:0)	2.1	A	495.28, 185.42, 104.24
1-Eicosatrienoyl-sn-glycero-3-phosphoethanolamine	PE(18:1/20:3)	2.1	B	503.47, 461.26,
1-Eicosatrienoyl-sn-glycero-3-phosphoethanolamine	PE(18:1/20:3)	2.1	B	504.38, 461.28, 341.34, 184.07
1-(1Z-Octadecenyl)-sn-glycero-3-phosphocholine	LysoPC(P-18:0)	2.1	B	508.38, 184.26, 104.23
1-Heptadecanoyl-sn-glycero-3-phosphocholine	PC(17:0/0:0)	2.1	A	511.37, 256.21, 184.07, 104.23
1-Oleoyl-sn-glycero-3-phosphocholine	PC(18:1/16:0)	2.1	B	521.33, 184.07, 104.23
1-O-Hexadecyl-2-O-butanoyl-sn-glyceryl-3-phosphocholine	Butanoyl PAF	2.1	B	574.48, 184.07
Androstane-3,17-diol, (3β,5α,17β)-	3beta-Androstanediol	2.1	B	276.04, 257.23
Inosine	Inosine	2.1	B	291.03, 158.9
L-Glutathione, reduced	L-Glutathione	2.1	A	308.06, 233.03, 162.02, 76.06
[Arg8] α-Neo-Endorphin (1-8)	[Arg8] alpha-Neo-Endorphin (1-8)	2.1	B	331.28, 175.01, 119.02
Arg-Tyr	Arg-Tyr	2.1	C	337.1, 277.05
Adenosine 3'-monophosphate	AMP	2.1	B	348.08, 136.06
3α-Hydroxy-7-oxo-5β-cholanic acid	Nutriacholic acid	2.1	B	354.2, 161.21
Ursocholic acid	Ursocholic acid	2.1	B	355.2, 215.13
1a,1b-Dihomoprostaglandin F2α	1a,1b-dihomo PGF2alpha	2.1	B	363.12, 318.2
L-Cysteine-glutathione disulfide	L-Cysteine-glutathione disulfide	2.1	B	425.03, 304.03, 175.02
1-Palmitoyl-2-hydroxy-sn-glycero-3-phosphoethanolamine	LysoPE(16:0)	2.1	B	451.18, 255.23, 140.02
1-Octadecyl-sn-glycero-3-phosphoethanolamine	PE(O-18:0/0:0)	2.1	B	466.37, 405.07
1-Stearoyl-2-hydroxy-sn-glycero-3-phosphoethanolamine	LysoPE(18:0)	2.1	B	479.3, 283.25, 196.06
1-Stearoyl-2-hydroxy-sn-glycero-3-phosphoethanolamine	LysoPE(18:0)	2.1	B	479.36, 283.47
Tauroursodeoxycholic acid	Tauroursodeoxycholic acid	2.1	B	498.28
1-(5Z,8Z,11Z,14Z-Eicosatetraenoyl)-sn-glycero-3-phosphocholine	PI(20:4/0:0)	2.1	B	579.41, 303.27,
Ile-Ala-Lys	Ile-Ala-Lys	2.1	B	332.06, 185.13, 130.08, 104.11
Docosahexaenoylglycine	Docosahexaenoylglycine	2.1	B	385.23, 131.09
Tauroursodeoxycholic acid	Tauroursodeoxycholic acid	2.1	A	464.33, 339.24
Tauroursodeoxycholic acid	Tauroursodeoxycholic acid	2.1	B	483.27, 464.23, 337.25, 171.14, 148.02, 128.08, 107.08
1-(1Z,12Z-Nonadecadienyl)-sn-glycero-3-phosphocholine	PC(P-19:1/0:0)	2.1	C	520.23, 184.07
1-Oleoyl-sn-glycero-3-phosphocholine	LysoPC(18:1)	2.1	A	521.28, 184.07
Dilinolenin (9c,12c,15c)	DDG(18:3)	2.1	C	540.31, 503.4
1,2-Bis(O-decanoyl)-sn-glyceryl-3-phosphorylcholine	Didecanoyl Lecithin	2.1	B	566.38, 184.07
Phe-DMet-Arg-Phe-NH2	Phe-DMet-Arg-Phe-NH2	2.1	C	599.42
L-Citrulline	L-Citrulline	2.1	B	131.08
Prostaglandin B3	Prostaglandin B3	2.1	B	313.26, 269.19
1-Hexadecanoyl-2-sn-glycero-3-phosphate	GP(16:0)	2.1	B	410.27, 152.99
1-(1Z-Octadecenyl)-2-(4Z,7Z,10Z,13Z,16Z,19Z-docosahexaenoyl)-sn-glycero-3-phosphoethanolamine	GPEtn(18:0p/22:6)	2.1	A	463.28, 267.23, 196.08, 140.01
N-Oleoyl-4-sphingenine	Cer(D18:1/18:1)	2.1	B	562.38, 307.04
(R)-N-(1-(4-Hydroxyphenyl)-2-hydroxyethyl)oleamide	Omdm-2	2.1	C	413.32
Lyso-sphingomyelin	Lyso-SM	2.1	B	464.36, 184.52, 104.1
2-Arachidonoyl-1-palmitoyl-sn-glycero-3-phosphoethanolamine	PE(16:0/20:4)	2.1	A	599.5, 513.27

[a.] Confidence of MS/MS matching. A represents high confidence, B for good match and C for moderate match.

Supplementary Table 2A

Radiation dysregulation pathways in jejunum

**Pathway**	**Day 4**		**Day 9**	
	overlap size	*p-value*	*overlap size*	*p-value*
Arachidonic acid metabolism	8(14)[a]	2.15E-02	7(14)	1.68E-04
Squalene and cholesterol biosynthesis	5(5)	6.72E-04	2(5)	2.76E-02
Purine metabolism	-	-	5(17)	4.79E-03
Ascorbate (vitamin C) and aldarate metabolism	-	-	2(3)	6.81E-03
Leukotriene metabolism	-	-	4(9)	7.06E-03
Bile acid biosynthesis	-	-	6(27)	7.39E-03
Glycine, serine, alanine and threonine metabolism	-	-	3(8)	7.73E-03
Glycosphingolipid biosynthesis - globoseries	3(3)	8.99E-03	-	-
Limonene and pinene degradation	3(3)	8.99E-03	-	-
Vitamin A (retinol) metabolism	-	-	4(10)	9.41E-03
Starch and sucrose metabolism	4(5)	1.05E-02	2(5)	2.76E-02
Vitamin D3 (cholecalciferol) metabolism	3(4)	3.40E-02	2(4)	1.38E-02
Prostaglandin formation from arachidonate	-	-	3(7)	1.58E-02
Tryptophan metabolism	6(10)	1.95E-02	-	-
Histidine metabolism	-	-	2(5)	2.76E-02
C21-steroid hormone biosynthesis and metabolism	-	-	3(11)	2.84E-02
N-Glycan biosynthesis	4(6)	2.97E-02	-	-
Glycolysis and gluconeogenesis	-	-	2(4)	3.12E-02
Putative anti-inflammatory metabolites formation from EPA	-	-	2(4)	3.12E-02
Pentose phosphate pathway	-	-	2(4)	3.12E-02
Vitamin B2 (riboflavin) metabolism	2(2)	3.56E-02	-	-
Blood group biosynthesis	2(2)	3.56E-02	-	-
Glycosphingolipid biosynthesis - lactoseries	2(2)	3.56E-02	-	-
Glycosphingolipid biosynthesis - neolactoseries	2(2)	3.56E-02	-	-
Keratan sulfate biosynthesis	2(2)	3.56E-02	-	-
O-Glycan biosynthesis	2(2)	3.56E-02	-	-
Propanoate metabolism	-	-	1(1)	3.87E-02
Mono-unsaturated fatty acid beta-oxidation	-	-	1	3.87E-02

Note. Pathway analysis result with positive mode and negative mode combined.

[a] Numbers in parathesis indicates the pathway size.

Supplementary Table 2B

Radiation dysregulation pathways in lung

**Pathway**	**Day 4**		**Day 9**	
	overlap size	*p-value*	*overlap size*	*p-value*
N-Glycan biosynthesis	7(7)[a]	2.52E-04	-	-
Tyrosine metabolism	9(10)	2.52E-04	-	-
Glycosphingolipid biosynthesis - ganglioseries	5(5)	4.20E-04	2(7)	4.02E-02
Galactose metabolism	5(5)	2.27E-03	2(5)	2.16E-02
Glycosphingolipid biosynthesis - globoseries	5(5)	2.27E-03	-	-
Phosphatidylinositol phosphate metabolism	6(8)	2.35E-03	2(6)	2.92E-02
Hexose phosphorylation	-	-	2(2)	3.02E-03
N-Glycan degradation	-	-	2(2)	3.02E-03
Purine metabolism	6(7)	5.38E-03	-	-
Glycosphingolipid biosynthesis - lactoseries	3(3)	6.30E-03	-	-
Blood group biosynthesis	3(3)	6.30E-03	-	-
Glycosphingolipid biosynthesis - neolactoseries	3(3)	6.30E-03	-	-
O-Glycan biosynthesis	3(3)	6.30E-03	-	-
Keratan sulfate biosynthesis	3(3)	6.30E-03	-	-
Sialic acid metabolism	4(5)	6.39E-03	3(10)	1.55E-02
Fructose and mannose metabolism	2(2)	2.61E-02	2(3)	7.98E-03
Glycolysis and gluconeogenesis	4(4)	8.32E-03	-	-
Histidine metabolism	4(4)	8.32E-03	-	-
Ascorbate (vitamin C) and aldarate metabolism	4(4)	8.32E-03	1(2)	2.76E-02
Methionine and cysteine metabolism	4(4)	8.32E-03	-	-
Arachidonic acid metabolism	-	-	2(9)	1.15E-02
Pyruvate metabolism	3(3)	2.73E-02	1(1)	1.24E-02
Putative anti-Inflammatory metabolites formation from EPA	-	-	1(1)	1.24E-02
Glycosphingolipid metabolism	8(17)	1.80E-02	3(10)	1.55E-02
Glutathione metabolism	3(4)	2.29E-02	-	-
Keratan sulfate degradation	2(2)	2.61E-02	-	-
Pentose phosphate pathway	3(3)	2.73E-02	-	-
Chondroitin sulfate degradation	-	-	1(1)	3.27E-02
Caffeine metabolism	-	-	1(1)	3.27E-02
Heparan sulfate degradation	-	-	1(1)	3.27E-02

Glycine, serine, alanine and threonine metabolism	5(7)	4.50E-02	-	-
Aspartate and asparagine metabolism	5(7)	4.50E-02	-	-
Aminosugars metabolism	3(5)	4.82E-02	-	-

Note. Pathway analysis result with positive mode and negative mode combined.

[a] Numbers in parathesis indicates the pathway size.

Supplementary Table 3A

Metabolic changes due to Amifostine Treatment (Amifostine side effect in SD4 and SD9 for validated metabolites across all tissues)

			Day 4					Day 9			
			Amifostine 50 mg		Amifostine 200 mg			Amifostine 50 mg		Amifostine 200 mg	
**Name**	**Organ/Tissue**	**Ionization Mode**	***p-value***	***FDR*** [a]	***p-value2***	***FDR***	***Column3***	***p-value4***	***FDR5***	***p-value6***	***FDR7***
Hydroxyphenyl-2-hydroxyethyl oleamide	Lung	Positive	3.14E-08	5.56E-05	1.14E-13	3.91E-10		8.49E-05	NS	1.74E-05	2.66E-02
PG(18:0/20:4)	Bone marrow	Negative	NS	NS	2.61E-05	2.69E-02		NS	NS	NS	NS
PE(P-16:0/22:6)	Bone marrow	Negative	NS	NS	1.20E-03	NS		NS	NS	NS	NS
GPE(P-18:0/20:4)	Bone marrow	Negative	NS	NS	1.24E-02	NS		NS	NS	NS	NS
Docosahexaenoylglycine	Jejunum	Positive	3.27E-03	NS	1.50E-02	NS		1.53E-03	NS	9.12E-03	NS
PG(18:0/22:6)	Bone marrow	Negative	NS	NS	1.53E-02	NS		4.19E-02	NS	NS	NS
PG(16:0/18:1)	Bone marrow	Negative	2.92E-02	NS	1.60E-02	NS		NS	NS	NS	NS
Lyso-SM	Lung	Positive	NS	NS	1.74E-02	NS		NS	NS	NS	NS
PE(18:0/22:6)	Bone marrow	Negative	2.86E-02	NS	2.07E-02	NS		NS	NS	NS	NS
L-Cysteine-glutathione disulfide	Jejumun	Negative	2.90E-03	NS	2.42E-02	NS		NS	NS	NS	NS
N-Stearoyltaurine	Bone marrow	Negative	NS	NS	2.95E-02	NS		1.40E-02	NS	NS	NS
PA(18:0/20:4)	Bone marrow	Negative	NS	NS	3.33E-02	NS		NS	NS	NS	NS
PC(p-18:0/18:1)	Bone marrow	Negative	NS	NS	3.72E-02	NS		NS	NS	NS	NS
PC(O-16:0/20:4)	Bone marrow	Negative	NS	NS	3.77E-02	NS		NS	NS	NS	NS
PI(20:4/0:0)	Jejumun	Negative	1.38E-02	NS	4.33E-02	NS		1.37E-02	NS	1.54E-02	NS
PA(18:0/18:2)	Bone marrow	Negative	NS	NS	NS	NS		NS	NS	NS	NS
PE(P-18:0/18:1)	Bone marrow	Negative	NS	NS	NS	NS		NS	NS	NS	NS
PC(18:3/18:3)	Bone marrow	Negative	NS	NS	NS	NS		NS	NS	NS	NS
PE-NMe(16:0/16:0)	Bone marrow	Negative	NS	NS	NS	NS		NS	NS	NS	NS
PC(17:0/20:4)	Bone marrow	Negative	NS	NS	NS	NS		NS	NS	NS	NS
PE(18:1/18:1)	Bone marrow	Negative	NS	NS	NS	NS		NS	NS	NS	NS
DHPE	Bone marrow	Negative	NS	NS	NS	NS		2.91E-02	NS	NS	NS
PS(18:0/18:2)	Bone marrow	Negative	NS	NS	NS	NS		NS	NS	NS	NS
PC(18:0/22:6)	Bone marrow	Negative	NS	NS	NS	NS		NS	NS	NS	NS
PI(18:0/0:0)	Bone marrow	Negative	NS	NS	NS	NS		NS	NS	NS	NS
C16-18:1 PE	Bone marrow	Negative	NS	NS	NS	NS		2.65E-02	NS	NS	NS
PI(18:0/18:0)	Bone marrow	Negative	NS	NS	NS	NS		3.04E-02	NS	NS	NS
PC(16:0/18:2)	Bone marrow	Negative	NS	NS	NS	NS		NS	NS	NS	NS
DHAPC	Bone marrow	Negative	2.54E-02	NS	NS	NS		3.04E-02	NS	NS	NS
PC(18:0/18:1)	Bone marrow	Negative	NS	NS	NS	NS		NS	NS	NS	NS
PI(18:1/0:0)	Bone marrow	Negative	NS	NS	NS	NS		7.90E-03	NS	NS	NS
PC(18:0/18:2)	Bone marrow	Negative	NS	NS	NS	NS		NS	NS	NS	NS
PC(18:0/20:4)	Bone marrow	Negative	NS	NS	NS	NS		NS	NS	NS	NS
(Lys8,Lys9)-Neurotensin (8-13)	Bone marrow	Negative	NS	NS	NS	NS		NS	NS	NS	NS
PE(O-16:0/18:1)	Bone marrow	Negative	NS	NS	NS	NS		3.41E-02	NS	NS	NS
PE(17:0/20:4)	Bone marrow	Negative	NS	NS	NS	NS		NS	NS	NS	NS
16:0-18:1-PS	Bone marrow	Negative	NS	NS	NS	NS		NS	NS	NS	NS
PC(18:1/14:0)	Bone marrow	Negative	NS	NS	NS	NS		NS	NS	NS	NS
PE(16:0/18:2)	Bone marrow	Negative	NS	NS	NS	NS		NS	NS	NS	NS
PE(18:0/18:2)	Bone marrow	Negative	NS	NS	NS	NS		NS	NS	NS	NS
PC(16:0/12:0)	Bone marrow	Negative	NS	NS	NS	NS		NS	NS	NS	NS
PE(22:6/22:6)	Bone marrow	Negative	NS	NS	NS	NS		NS	NS	NS	NS
PC(16:0/18:1)	Bone marrow	Negative	NS	NS	NS	NS		NS	NS	NS	NS
PG(21:0/22:6)	Bone marrow	Negative	NS	NS	NS	NS		2.33E-02	NS	NS	NS
PC(16:0/14:0)	Bone marrow	Negative	NS	NS	NS	NS		NS	NS	NS	NS
PA(18:0/18:1)	Bone marrow	Negative	NS	NS	NS	NS		4.68E-02	NS	NS	NS
1,4-IP2	Bone marrow	Negative	NS	NS	NS	NS		NS	NS	NS	NS
Prostaglandin F3alpha	Bone marrow	Negative	NS	NS	NS	NS		NS	NS	NS	NS
GICC(d18:1/12:0)	Bone marrow	Negative	NS	NS	NS	NS		NS	NS	NS	NS
PIP2(16:0/16:0)	Bone marrow	Negative	NS	NS	NS	NS		NS	NS	NS	NS
HPOTA	Bone marrow	Negative	NS	NS	NS	NS		NS	NS	NS	NS
PE(18:1/0:0)	Bone marrow	Negative	NS	NS	NS	NS		NS	NS	NS	NS
PG(16:0/22:6)	Bone marrow	Negative	NS	NS	NS	NS		NS	NS	NS	NS
PC(22:0/0:0)	Bone marrow	Negative	NS	NS	NS	NS		NS	NS	NS	NS
8(9)-EpETE	Bone marrow	Negative	NS	NS	NS	NS		2.98E-02	NS	NS	NS
PC(20:4)	Bone marrow	Negative	NS	NS	NS	NS		NS	NS	NS	NS
GP1	Bone marrow	Negative	NS	NS	NS	NS		NS	NS	NS	NS
LysoPC(18:0)	Bone marrow	Negative	NS	NS	NS	NS		NS	NS	NS	NS
PE(16:0/20:4)	Bone marrow	Negative	NS	NS	NS	NS		NS	NS	NS	NS
LysoPE(16:0)	Bone marrow	Negative	NS	NS	NS	NS		NS	NS	NS	NS
14(15)-EpEDE	Bone marrow	Negative	NS	NS	NS	NS		1.79E-02	NS	NS	NS
3-Iodo-L-thyronine	Bone marrow	Negative	NS	NS	NS	NS		NS	NS	NS	NS
PA(O-16:0/0:0)	Bone marrow	Negative	NS	NS	NS	NS		NS	NS	NS	NS
GPEtn(18:0p/20:4)	Bone marrow	Negative	NS	NS	NS	NS		4.52E-02	NS	NS	NS
LysoPC(18:1)	Bone marrow	Negative	NS	NS	NS	NS		NS	NS	NS	NS
PC(16:0/22:6)	Bone marrow	Negative	NS	NS	NS	NS		NS	NS	NS	NS
17-Keto-docosahexaenoic acid	Bone marrow	Negative	NS	NS	NS	NS		NS	NS	NS	NS
LysoPC(15:0)	Bone marrow	Negative	NS	NS	NS	NS		NS	NS	NS	NS
PI(17:0/20:4)	Bone marrow	Negative	NS	NS	NS	NS		NS	NS	NS	NS
GPCho(18:0p/18:1)	Bone marrow	Negative	NS	NS	NS	NS		NS	NS	NS	NS
Ac-DY(2-malonyl)-VPML-NH2	Bone marrow	Negative	NS	NS	NS	NS		2.23E-02	NS	NS	NS
PI(16:0/16:0)	Bone marrow	Negative	NS	NS	NS	NS		NS	NS	NS	NS
PS(18:0/22:6)	Bone marrow	Negative	NS	NS	NS	NS		NS	NS	NS	NS
[Lys8]-ADH	Bone marrow	Negative	NS	NS	NS	NS		NS	NS	NS	NS
PS(18:0/20:4)	Bone marrow	Negative	NS	NS	NS	NS		NS	NS	NS	NS
LysoPC(17:0/0:0)	Bone marrow	Negative	NS	NS	NS	NS		NS	NS	NS	NS
PC(18:1/16:0)	Bone marrow	Negative	NS	NS	NS	NS		NS	NS	NS	NS
15(R),19(R)-HydroxyPGF1alpha	Bone marrow	Negative	NS	NS	NS	NS		NS	NS	NS	NS
PG(16:0/0:0)	Bone marrow	Negative	NS	NS	NS	NS		NS	NS	NS	NS
PC(14:0/18:0)	Bone marrow	Negative	NS	NS	NS	NS		NS	NS	NS	NS
TGT	Bone marrow	Negative	NS	NS	NS	NS		NS	NS	NS	NS
PI(17:1/0:0)	Bone marrow	Negative	NS	NS	NS	NS		NS	NS	NS	NS
PE(22:6)	Bone marrow	Negative	NS	NS	NS	NS		NS	NS	NS	NS
PS(16:0/20:4)	Bone marrow	Negative	NS	NS	NS	NS		NS	NS	NS	NS

PI(16:0/0:0)	Bone marrow	Negative	*NS*	*NS*	*NS*	*NS*	4.11E-02	*NS*	*NS*	*NS*
Ptd(S)Ins-(3,4)-P2 (1,2-dioctanoyl)	Bone marrow	Negative	*NS*	*NS*	*NS*	*NS*	*NS*	*NS*	*NS*	*NS*
PC(16:1/16:1)	Bone marrow	Negative	*NS*	*NS*	*NS*	*NS*	*NS*	*NS*	*NS*	*NS*
PI(18:0/20:4)	Bone marrow	Negative	*NS*	*NS*	*NS*	*NS*	*NS*	*NS*	*NS*	*NS*
C-8 Ceramide-1-phosphate	Bone marrow	Negative	*NS*	*NS*	*NS*	*NS*	*NS*	*NS*	*NS*	*NS*
Folic acid	Bone marrow	Negative	*NS*	*NS*	*NS*	*NS*	*NS*	*NS*	*NS*	*NS*
18:1 BMP (S,R)	Bone marrow	Negative	*NS*	*NS*	*NS*	*NS*	*NS*	*NS*	*NS*	*NS*
R-1 Methanandamide Phosphate	Bone marrow	Negative	*NS*	*NS*	*NS*	*NS*	*NS*	*NS*	*NS*	*NS*
Dopamine	Bone marrow	Positive	*NS*	*NS*	*NS*	*NS*	*NS*	*NS*	4.92E-03	*NS*
L-Glutathione	Bone marrow	Positive	*NS*	*NS*	*NS*	*NS*	*NS*	*NS*	4.04E-02	*NS*
AMP	Bone marrow	Positive	*NS*	*NS*	*NS*	*NS*	*NS*	*NS*	*NS*	*NS*
Acetyl-carnitine	Bone marrow	Positive	*NS*	*NS*	*NS*	*NS*	*NS*	*NS*	*NS*	*NS*
PC(17:0/0:0)	Bone marrow	Positive	*NS*	*NS*	*NS*	*NS*	*NS*	*NS*	*NS*	*NS*
1-SPP	Bone marrow	Positive	*NS*	*NS*	*NS*	*NS*	*NS*	*NS*	*NS*	*NS*
D-Glucosyl-beta1-1'-D-erythro-sphingosine	Bone marrow	Positive	*NS*	*NS*	*NS*	*NS*	*NS*	*NS*	*NS*	*NS*
Cytidine 5'-DP	Bone marrow	Positive	*NS*	*NS*	*NS*	*NS*	4.62E-02	*NS*	*NS*	*NS*
20-OH-LTB4	Bone marrow	Positive	*NS*	*NS*	*NS*	*NS*	*NS*	*NS*	*NS*	*NS*
3beta-Androstanediol	Bone marrow	Positive	*NS*	*NS*	*NS*	*NS*	*NS*	*NS*	*NS*	*NS*
PE(18:1/20:3)	Bone marrow	Positive	*NS*	*NS*	*NS*	*NS*	*NS*	*NS*	*NS*	*NS*
LysoPC(16:0)	Bone marrow	Positive	*NS*	*NS*	*NS*	*NS*	*NS*	*NS*	*NS*	*NS*
Oleoyl-L-carnitine	Bone marrow	Positive	*NS*	*NS*	*NS*	*NS*	*NS*	*NS*	*NS*	*NS*
Nutriacholic acid	Bone marrow	Positive	*NS*	*NS*	*NS*	*NS*	*NS*	*NS*	*NS*	*NS*
LysoPC(P-18:0)	Bone marrow	Positive	*NS*	*NS*	*NS*	*NS*	*NS*	*NS*	*NS*	*NS*
Unoprostone isopropyl ester	Bone marrow	Positive	*NS*	*NS*	*NS*	*NS*	*NS*	*NS*	*NS*	*NS*
Palmitoylcarnitine	Bone marrow	Positive	*NS*	*NS*	*NS*	*NS*	*NS*	*NS*	*NS*	*NS*
Ursocholic acid	Bone marrow	Positive	*NS*	*NS*	*NS*	*NS*	*NS*	*NS*	*NS*	*NS*
LH	Bone marrow	Positive	*NS*	*NS*	*NS*	*NS*	*NS*	*NS*	*NS*	*NS*
Butanoyl PAF	Bone marrow	Positive	*NS*	*NS*	*NS*	*NS*	*NS*	*NS*	*NS*	*NS*
[Arg8] alpha-Neo-Endorphin (1-8)	Bone marrow	Positive	*NS*	*NS*	*NS*	*NS*	*NS*	*NS*	*NS*	*NS*
Linoleoylcarnitine	Bone marrow	Positive	*NS*	*NS*	*NS*	*NS*	*NS*	*NS*	*NS*	*NS*
Inosine	Bone marrow	Positive	*NS*	*NS*	*NS*	*NS*	*NS*	*NS*	*NS*	*NS*
CTR	Bone marrow	Positive	*NS*	*NS*	*NS*	*NS*	3.74E-02	*NS*	*NS*	*NS*
LysoPC(14:0)	Bone marrow	Positive	*NS*	*NS*	*NS*	*NS*	*NS*	*NS*	*NS*	*NS*
LysoPE(18:0)	Jejumun	Negative	*NS*	*NS*	*NS*	*NS*	*NS*	*NS*	2.75E-04	*NS*
PE(O-18:0/0:0)	Jejumun	Negative	*NS*	*NS*	*NS*	*NS*	*NS*	*NS*	*NS*	*NS*
TUDCA	Jejumun	Negative	*NS*	*NS*	*NS*	*NS*	*NS*	*NS*	*NS*	*NS*
1a,1b-dihomo PGF2alpha	Jejumun	Negative	*NS*	*NS*	*NS*	*NS*	*NS*	*NS*	*NS*	*NS*
DDG(18:3)	Jejunum	Positive	*NS*	*NS*	*NS*	*NS*	3.86E-02	*NS*	*NS*	*NS*
PC(P-19:1/0:0)	Jejunum	Positive	*NS*	*NS*	*NS*	*NS*	*NS*	*NS*	*NS*	*NS*
F-dM-R-F-NH2	Jejunum	Positive	*NS*	*NS*	*NS*	*NS*	*NS*	*NS*	*NS*	*NS*
Didecanoyl Lecithin	Jejunum	Positive	*NS*	*NS*	*NS*	*NS*	*NS*	*NS*	*NS*	*NS*
Tauroursodeoxycholic acid	Jejunum	Positive	*NS*	*NS*	*NS*	*NS*	*NS*	*NS*	*NS*	*NS*
IAK	Jejunum	Positive	2.46E-02	*NS*	*NS*	*NS*	4.10E-02	*NS*	*NS*	*NS*
L-Citrulline	Lung	Negative	*NS*	*NS*	*NS*	*NS*	5.06E-03	*NS*	*NS*	*NS*
GPEtn(18:0p/22:6)	Lung	Negative	*NS*	*NS*	*NS*	*NS*	2.17E-02	*NS*	*NS*	*NS*
Cer(D18:1/18:1)	Lung	Negative	*NS*	*NS*	*NS*	*NS*	*NS*	*NS*	*NS*	*NS*
Prostaglandin B3	Lung	Negative	1.48E-02	*NS*	*NS*	*NS*	*NS*	*NS*	*NS*	*NS*

Note. All metabolite names are validated through tandem MS.

[a] Numbers are FDR adjusted *P* values. NS = not significant (FDR, $P > 0.05$).

Supplementary Table 3B

Metabolic changes due to 200 mg/kg Amifostine Treatment (SD9 for validated metabolites across all tissues)

Name	Organ/Tissue	*p-value*	*FDR* [a]	*Fold Change*		*Log2(FC)*
Dopamine	Bone marrow	4.92E-03	*NS*	0.6713	↓	-0.5750
L-Glutathione	Bone marrow	4.04E-02	*NS*	0.6455	↓	-0.6314
LysoPE(18:0)	Jejumun	2.75E-04	*NS*	0.4292	↓	-1.2201
PI(20:4/0:0)	Jejumun	1.54E-02	*NS*	0.6576	↓	-0.6048
Docosahexaenoylglycine	Jejunum	9.12E-03	*NS*	0.7047	↓	-0.5050
Hydroxyphenyl-2-hydroxyethyl oleamide	Lung	1.74E-05	2.66E-02	13.5970	↑	3.7652

Note. All metabolite names are validated through tandem MS.

[a] Numbers are FDR adjusted *P* values. NS = not significant (FDR, *P* value > 0.05).

Supplementary Table 3C

Metabolic changes due to 50 mg/kg Amifostine Treatment (SD4 for validated metabolites across all tissues)

**Name**	**Organ/Tissue**	***p-value***	***FDR*** [a]	***Fold Change***	***Log2(FC)***
DHAPC	Bone marrow	2.54E-02	*NS*	1.1208	↑ 0.1646
PE(18:0/22:6)	Bone marrow	2.86E-02	*NS*	2.1102	↑ 1.0774
PG(16:0/18:1)	Bone marrow	2.92E-02	*NS*	1.0330	↑ 0.0469
GPEtn(18:0p/20:4)	Bone marrow	1.31E-02	*NS*	1.0782	↑ 0.1087
Oleoyl-L-carnitine	Bone marrow	2.56E-02	*NS*	0.9334	↓ -0.0993
PI(20:4/0:0)	Jejumun	1.38E-02	*NS*	0.4380	↓ -1.1908
L-Cysteine-glutathione disulfide	Jejumun	2.90E-03	*NS*	1.2271	↑ 0.2953
Docosahexaenoylglycine	Jejunum	2.92E-02	*NS*	1.5238	↑ 0.6077
IAK	Jejunum	3.27E-03	*NS*	1.1605	↑ 0.2148
Prostaglandin B3	Lung	2.46E-02	*NS*	1.5358	↑ 0.6190
Hydroxyphenyl-2-hydroxyethyl oleamide	Lung	1.48E-02	*NS*	1.0132	↑ 0.0189

Note. All metabolite names are validated through tandem MS.

[a] Numbers are FDR adjusted *P* values. NS = not significant (FDR, *P* value > 0.05).

Supplementary Table 3D

Metabolic changes due to 50 mg/kg Amifostine Treatment (SD9 for validated metabolites across all tissues)

Name	Organ/Tissue	*p-value*	*FDR* [a]	*Fold Change*	*Log2(FC)*
PE(P-18:0/18:1)	Bone marrow	3.89E-02	*NS*	0.7590	↓-0.3979
DHPE	Bone marrow	2.91E-02	*NS*	0.7472	↓-0.4205
PE-NMe(16:0/16:0)	Bone marrow	2.74E-02	*NS*	0.7725	↓-0.3724
C16-18:1 PE	Bone marrow	2.65E-02	*NS*	0.7748	↓-0.3681
PI(18:0/18:0)	Bone marrow	3.04E-02	*NS*	1.2369	↑0.3068
DHAPC	Bone marrow	3.04E-02	*NS*	1.1653	↑0.2207
PI(18:1/0:0)	Bone marrow	7.90E-03	*NS*	1.3935	↑0.4787
PC(18:0/22:6)	Bone marrow	4.08E-02	*NS*	1.2022	↑0.2657
PE(O-16:0/18:1)	Bone marrow	3.41E-02	*NS*	0.7573	↓-0.4010
PG(18:0/22:6)	Bone marrow	4.19E-02	*NS*	1.1973	↑0.2598
PG(21:0/22:6)	Bone marrow	2.33E-02	*NS*	0.6918	↓-0.5316
PA(18:0/18:1)	Bone marrow	4.68E-02	*NS*	0.7850	↓-0.3492
8(9)-EpETE	Bone marrow	2.98E-02	*NS*	1.1155	↑0.1577
PS(18:0/18:2)	Bone marrow	2.80E-02	*NS*	0.7695	↓-0.3779
14(15)-EpEDE	Bone marrow	1.79E-02	*NS*	1.4835	↑0.5690
GPEtn(18:0p/20:4)	Bone marrow	4.52E-02	*NS*	0.8094	↓-0.3051
Ac-DY(2-malonyl)-VPML-NH2	Bone marrow	2.23E-02	*NS*	0.7580	↓-0.3997
N-Stearoyltaurine	Bone marrow	1.40E-02	*NS*	1.2383	↑0.3084
PI(16:0/0:0)	Bone marrow	4.11E-02	*NS*	1.2217	↑0.2889
Cytidine 5'-DP	Bone marrow	4.62E-02	*NS*	0.8195	↓-0.2871
CTR	Bone marrow	3.74E-02	*NS*	1.2448	↑0.3159
PI(20:4/0:0)	Jejumun	1.37E-02	*NS*	0.6441	↓-0.6347
DDG(18:3)	Jejunum	3.86E-02	*NS*	0.6767	↓-0.5633
Docosahexaenoylglycine	Jejunum	1.53E-03	*NS*	0.6190	↓-0.6919
IAK	Jejunum	4.10E-02	*NS*	0.6337	↓-0.6581
L-Citrulline	Lung	5.06E-03	*NS*	1.3089	↑0.3884
GPEtn(18:0p/22:6)	Lung	2.17E-02	*NS*	1.1554	↑0.2084
Hydroxyphenyl-2-hydroxyethyl oleamide	Lung	8.49E-05	*NS*	2.4912	↑1.3169

Note. All metabolite names are validated through tandem MS.

[a] Numbers are FDR adjusted *P* values. NS = not significant (FDR, *P* value > 0.05).

Supplementary Table 4A

Pathways Analysis of Amifostine 50 mg/kg benefit to radiation jejunum injury

Pathway	Day 4		Day 9	
	overlap size	*p-value*	*overlap size*	*p-value*
Urea cycle/amino group metabolism	3(7)[a]	1.41E-02	-	-
Alanine and aspartate metabolism	-	-	1(6)	2.54E-02
Drug metabolism - other enzymes	1(1)	2.69E-02	-	-
Vitamin B9 (folate) metabolism	2(4)	2.97E-02	-	-
D4&E4-neuroprostanes formation	-	-	2(4)	3.03E-02
Sialic acid metabolism	3(10)	3.87E-02	-	-
Squalene and cholesterol biosynthesis	2(5)	4.56E-02	-	-
Aspartate and asparagine metabolism	-	-	1(10)	4.59E-02
Vitamin A (retinol) metabolism	-	-	3(10)	4.67E-02

Note. Pathway analysis result with positive mode and negative mode combined

[a] Numbers in parathesis indicates the pathway size.

Supplementary Table 4B

Pathways Analysis of Amifostine 200 mg/kg benefit to radiation jejunum injury

**Pathway**	**Day 4**		**Day 9**	
	overlap size	*p-value*	*overlap size*	*p-value*
Arachidonic acid metabolism	2(14)[a]	2.31E-02	5(14)	2.52E-04
C21-steroid hormone biosynthesis and metabolism	5(11)	7.56E-04	-	-
Glutathione metabolism	4(4)	1.01E-03	-	-
Vitamin A (retinol) metabolism	6(12)	1.03E-02	3(12)	1.43E-03
Aspartate and asparagine metabolism	4(7)	3.78E-03	-	-
Glutamate metabolism	3(3)	3.95E-03	-	-
Porphyrin metabolism	2(2)	4.71E-03	-	-
Glycine, serine, alanine and threonine metabolism	2(7)	6.47E-03	-	-
Ascorbate (vitamin C) and aldarate metabolism	2(2)	8.65E-03	-	-
Androgen and estrogen biosynthesis and metabolism	2(3)	9.33E-03	-	-
Purine metabolism	2(9)	9.50E-03	-	-
R group synthesis	1(1)	1.08E-02	-	-
Pyruvate metabolism	1(1)	1.08E-02	-	-
Fatty acid oxidation	1(1)	1.08E-02	-	-
Dynorphin metabolism	1(1)	1.08E-02	1(1)	3.31E-02
TCA cycle	1(1)	1.08E-02	-	-
Dimethyl-branched-chain fatty acid mitochondrial beta-oxidation	1(1)	1.08E-02	-	-
Tryptophan metabolism	2(11)	1.35E-02	-	-
Prostaglandin formation from arachidonate	3(7)	3.61E-02	2(11)	1.42E-02
Putative anti-inflammatory metabolites formation from EPA	2(2)	2.16E-02	-	-
Propanoate metabolism	1(2)	2.34E-02	-	-
Saturated fatty acids beta-oxidation	1(2)	2.34E-02	-	-
Mono-unsaturated fatty acid beta-oxidation	1(2)	2.34E-02	-	-
CoA catabolism	-	-	1(2)	2.42E-02
Phytanic acid peroxisomal oxidation	-	-	1(4)	2.67E-02
Histidine metabolism	2(5)	2.92E-02	-	-
Leukotriene metabolism	-	-	2(9)	2.93E-02
Vitamin E metabolism	2(3)	3.02E-02	-	-
Methionine and cysteine metabolism	3(11)	3.18E-02	-	-
Squalene and cholesterol biosynthesis	3(5)	3.63E-02	-	-
Omega-6 fatty acid metabolism	-	-	1(3)	3.69E-02
Xenobiotics metabolism	1(3)	4.13E-02	1(6)	4.05E-02
Di-unsaturated fatty acid beta-oxidation	1(3)	4.13E-02	-	-
Vitamin B2 (riboflavin) metabolism	1(3)	4.13E-02	-	-
Valine, leucine and isoleucine degradation	1(3)	4.13E-02	-	-
Arginine and proline metabolism	1(3)	4.13E-02	-	-
Lysine metabolism	1(3)	4.13E-02	-	-
Vitamin B5 - CoA biosynthesis from pantothenate	-	-	1(4)	4.80E-02

Note. Pathway analysis result with positive mode and negative mode combined

[a] Numbers in parathesis indicates the pathway size.

Supplementary Table 5A

Pathways Analysis of Amifostine 50 mg/kg benefit to radiation lung injury

Pathway	Day 4		Day 9	
	overlap size	*p-value*	*overlap size*	*p-value*
C21-steroid hormone biosynthesis and metabolism	-	-	2(5)	2.71E-02
Glycosylphosphatidylinositol(GPI)-anchor biosynthesis	-	-	1(1)	3.55E-02
Di-unsaturated fatty acid beta-oxidation	1(5)[a]	3.76E-02	-	-
Linoleate metabolism	1(7)	4.44E-02	-	-
Fatty acid metabolism	1(7)	4.44E-02	-	-

Note. Pathway analysis result with positive mode and negative mode combined

[a] Numbers in parathesis indicates the pathway size.

Supplementary Table 5B

Pathways Analysis of Amifostine 200 mg/kg benefit to radiation lung injury

**Pathway**	**Day 4**		**Day 9**	
	overlap size	*p-value*	*overlap size*	*p-value*
Fatty acid activation	-	-	7(11)	8.40E-05
De novo fatty acid biosynthesis	2(4)[a]	1.52E-02	8(15)	8.40E-05
Glycerophospholipid metabolism	-	-	7(19)	2.52E-04
Urea cycle/amino group metabolism	3(3)	5.04E-04	-	-
TCA cycle	3(3)	5.04E-04	-	-
Carnitine shuttle	4(24)	1.26E-03	-	-
Vitamin A (retinol) metabolism	2(14)	3.39E-02	5(14)	2.77E-03
Putative anti-Inflammatory metabolites formation from EPA	-	-	2(3)	4.29E-03
Linoleate metabolism	2(7)	6.30E-03	6(7)	1.75E-02
Fatty acid metabolism	-	-	3(7)	7.98E-03
Arachidonic acid metabolism	2(9)	1.52E-02	-	-
Xenobiotics metabolism	-	-	1(1)	2.22E-02
Leukotriene metabolism	3(8)	2.23E-02	-	-
Ascorbate (vitamin C) and aldarate metabolism	2(4)	2.81E-02	-	-
Prostaglandin formation from arachidonate	3(9)	2.98E-02	-	-
Vitamin B3 (nicotinate and nicotinamide) metabolism	-	-	2(5)	4.13E-02
Di-unsaturated fatty acid beta-oxidation	-	-	2(5)	4.13E-02
Porphyrin metabolism	2(5)	4.13E-02	-	-
R group synthesis	1(1)	4.70E-02	-	-
Saturated fatty acids beta-oxidation	1(1)	4.70E-02	-	-
Fatty acid oxidation	1(1)	4.70E-02	-	-
Dimethyl-branched-chain fatty acid mitochondrial beta-oxidation	1(1)	4.70E-02	-	-

Note. Pathway analysis result with positive mode and negative mode combined

[a] Numbers in parathesis indicates the pathway size.

Supplementary Table 6. Statistical analysis results for all tandem MS validated metabolites including all comparisons

To visualize the data on table 6, please zoom to 600%.

													1. Metabolites affected by drug only<br>Day 4<br>ami_50/Vehicle		
Type	RT	erimental.i	Adduct	PPM.Error	lolecular.Weig	Name	Formula	Kegg	HMDB	LipidMaps	Metlin	PubChem	p.value	FDR	old.Ch
Bonemar	11.02	797.5311	M-H	3	798.5410853	PG(18:1(11Z)/20:3(	C44H79O10P		HMDB0010624		61895	53480623	0.14195	0.5203	[illegible]
Bonemar	10.99	823.547	M-H	3	824.5567353	PG(18:1(11Z)/22:4(	C46H81O10P		HMDB0010626		61897	53480625	0.050638	0.45321	[illegible]
Bonemar	11	772.521	M-H	10	773.5206846	PS(20:2(11Z,14Z)/1	C41H76NO10P		HMDB011	LMGP0301	78226	52925672	0.23018	0.62638	[illegible]
Bonemar	10.99	771.5163	M-H	2	772.5254352	PG(18:3(6Z,9Z,12Z)	C42H77O10P		HMDB001	LMGP0401	61933	52926642	0.14998	0.53408	[illegible]
Bonemar	11.04	798.5369	M-H	10	799.536338	PS(20:1(11Z)/17:2(	C43H78NO10P			LMGP0301	78202		0.22971	0.62575	[illegible]
Bonemar	6.86	466.3273	M-H	6	467.3375748	PC(O-14:0/O-1:0)	C23H50NO6P				40194		0.57735	0.85518	[illegible]
Bonemar	10.06	698.5118	M-H	2	699.5202902	PE(18:2(9Z,12Z)/P-1	C39H74NO7P		HMDB0009114		60521	53479669	0.66346	0.89134	[illegible]
Bonemar	9.9	712.5046	M+Cl	1	677.53594	PE(O-16:0/16:0)[U]	C37H76NO7P				40748		0.94609	0.98322	[illegible]
Bonemar	10.08	731.5759	M-H	7	732.578141	PE-Cer(d14:1(4E)/2	C40H81N2O7P			LMSP0302	103103		0.60484	0.86781	[illegible]
Bonemar	10.11	728.5563	M-H	5	729.5672404	PE(18:1(11Z)/P-18:	C41H80NO7P		HMDB0009049		60466	53479647	0.98427	0.99302	[illegible]
Bonemar	10.03	725.5295	M+Cl	5	690.556344	PA(O-20:0/16:0)	C39H79O7P			LMGP1002	82218		0.51745	0.83872	[illegible]
Bonemar	10.09	730.5712	M-H	6	731.5828905	PC(P-18:0/15:0)	C41H82NO7P		HMDB001	LMGP0102	62064	52923930	0.43752	0.80327	[illegible]
Bonemar	10.12	727.546	M-H	9	728.546841	PE-Cer(d14:2(4E,6E	C40H77N2O7P			LMSP0302	103112		0.82378	0.95024	[illegible]
Bonemar	9.81	993.56	M-H	4	994.563008	PIM1(18:1(9Z)/16:1	C49H87O18P			LMGP15010023			0.54128	0.84675	[illegible]
Bonemar	6.25	476.2772	M-H	7	477.2812	Arg Phe Arg	C21H35N9O4				19179		0.54192	0.84675	[illegible]
Bonemar	9.9	813.577	M+Cl	2	778.608774	PG(O-16:0/21:0)	C43H87O9P			LMGP0402	79825		0.37545	0.7622	[illegible]
Bonemar	10.09	704.5568	M-H	4	705.56724	PE(O-18:0/16:0)[U]	C39H80NO7P				40749		0.83422	0.95257	[illegible]
Bonemar	10.11	729.5609	M-H	8	730.562491	PE-Cer(d14:1(4E)/2	C40H79N2O7P			LMSP0302	103104		0.97824	0.9928	[illegible]
Bonemar	10.12	726.5424	M-H	3	727.5515904	PE(18:1(11Z)/P-18:	C41H78NO7P		HMDB0009051		60468	53479649	0.73489	0.92076	[illegible]
Bonemar	10.08	753.5585	M-H	8	754.559501	MGDG(16:0/18:2(9	C43H78O10			LMGL0501	46662		0.6502	0.88722	[illegible]
Bonemar	9.75	935.5395	M+Cl	3	900.572784	PI(18:4(6Z,9Z,12Z,1	C48H85O13P			LMGP0601	80440		0.085515	0.46987	[illegible]
Bonemar	10.01	870.6207	M-H	3	871.6302351	PS(24:1(15Z)/18:1(	C48H90NO10P		HMDB0112916			131820035	0.64655	0.88558	[illegible]
[illegible]	[illegible]	[illegible]	[illegible]	[illegible]	[illegible]	[illegible]	[illegible]	[illegible]	[illegible]	[illegible]	[illegible]	[illegible]	[illegible]	[illegible]	[illegible]

1. Metabolites affected by drug only
Day 4
ami_50/Vehicle

Type	RT	erimental.r	Adduct	PPM.Error	lolecular.Weig	Name	Formula	Kegg	HMDB	LipidMaps	Metlin	PubChem	p.value
Bonemar	12.2	786.5275	M-H	2	787.53633	PS(18:1(9Z)/18:1(9	C42H78NO10P				40798		0.73591
Bonemar	9.93	881.6042	M+Cl	0	846.634989	PG(21:0/20:1(11Z))	C47H91O10P			LMGP0401	79518		0.2986
Bonemar	10.11	759.6024	M-H	0	760.609441	PE-Cer(d16:1(4E)/2	C42H85N2O7P			LMSP0302	103125		0.90331
Bonemar	9.64	397.3357	M-H	8	398.339611	Tetracosanedioic aci	C24H46O4			LMFA0117	35991		0.76853
Bonemar	10.08	751.5466	M+Cl	7	716.571994	PA(O-20:0/18:1(9Z	C41H81O7P			LMGP1002	82199		0.31994
Bonemar	8.48	405.1806	M-H	6	406.18524	Tyr Gln Pro	C19H26N4O6				17234		0.37394
Bonemar	6.98	386.2338	M-H	8	387.244332	N-linoleoyl taurine	C20H37NO4S			LMFA0802	75520		0.64173
Bonemar	9.88	828.5711	M+Cl	4	793.5985406	PC(P-16:0/22:4(7Z	C46H84NO7P		HMDB0011226		62056	53480693	0.72013
Bonemar	9.86	565.5223	M-H	4	566.5274107	FAHFA(18:0/5-O-18	C36H70O4		HMDB011	LMFA07011042		101527487	0.40524
Bonemar	8.51	614.3982	M+Cl	4	579.4263899	PC(22:0/0:0)	C30H62NO7P		HMDB001	LMGP0105	61707	24779479	0.20611
Bonemar	10.17	860.6462	M-H	9	861.645888	PS(21:0/20:0)	C47H92NO10P			LMGP0301	78356		0.37615
Bonemar	12.16	235.0839	M-H	1	236.0909	Purine deoxyribonu	C10H12N4	C20463					0.3209
Bonemar	10.08	896.6533	M+Cl	2	861.682273	PS(O-20:0/22:0)	C48H96NO9P			LMGP0302	78716		0.97431
Bonemar	10.07	750.5428	M+Cl	3	715.5753998	SM C16:1	C40H80N2O6P		HMDB0029216		86096	124202091	0.30534
Bonemar	6.34	267.1452	M-H	4	268.15354	Histidylleucine;N-L-H[illegible]	[illegible]	C05010			66283	440549	0.0059007
Bonemar	9.99	723.5166	M+Cl	9	688.540694	PA(O-20:0/16:1(9Z	C39H77O7P			LMGP1002	82194		0.30718
Bonemar	11.49	1029.7648	M+Cl	4	994.7989411	TG(22:4(7Z,10Z,13Z	C66H106O6		HMDB0054781			131765344	0.38786
Bonemar	9.86	841.5725	M+Cl	1	806.603686	PG(i-13:0/a-25:0)	C44H87O10P		HMDB0116677			131823310	0.58834
Bonemar	9.96	583.5192	M+Cl	6	548.553231	Heneicosanyl palmit	C37H72O2			LMFA0701	75404		0.7033
Bonemar	9.74	786.5082	M-H	0	787.5152049	PE(20:3(5Z,8Z,11Z)	C45H74NO8P		HMDB0009335		60731	53479761	0.06430
Bonemar	12	869.5525	M-H	3	870.562219	PI(O-18:0/20:5(5Z,8	C47H83O12P			LMGP0602	81070		0.5507
Bonemar	9.05	341.2486	M-H	0	342.25588	Docosahexaenoic Ac	C23H34O2				44958		0.9234
Bonemar	9.92	880.6	M-H	8	881.614588	PS(22:4(7Z,10Z,13Z	C49H88NO10P			LMGP0301	78476		0.2341
Bonemar	10.14	782.5746	M-H	5	783.5778051	PC(20:3(5Z,8Z,11Z)	C44H82NO8P		HMDB000	LMGP0101	59810	53478949	0.7697
Bonemar	9.77	525.4521	M-H	1	526.4597251	DG(14:0/15:0/0:0)	C32H62O5		HMDB0007010		58645	53477949	0.3969
Bonemar	10.33	989.6517	M-H	1	990.660339	NeuAcalpha2-3Galb	C51H94N2O16			LMSP0601	56047		0.7217
Bonemar	10.13	823.5759	M-H	6	824.577869	PI(O-20:0/14:0)	C43H85O12P			LMGP0602	81028		0.4527
Bonemar	10.66	431.2667	M+Cl	4	396.298808	N-oleoyl asparagine	C22H40N2O4			LMFA0802	75519		0.3603
Bonemar	12.04	879.514	M-H	2	880.5230657	PGP(18:0/20:3(5Z,8	C44H82O13P2		HMDB0013510			53481828	0.8475
Bonemar	10.27	491.2714	M+Cl	7	456.298808	N-docosahexaenoyl	C27H40N2O4			LMFA0802	75511		0.0909
Bonemar	12.77	330.8377	M+Cl	0	295.86837	Lanosol;2,3-Dibrom	C7H6Br2O	C17098			71557	78659	0.8459
Bonemar	8.33	586.3651	M+Cl	1	551.3950688	PC(2:0/O-18:0)[U]	C28H58NO7P				40164		0.2052
Bonemar	7	624.3463	M+Cl	4	589.3743543	PC(11:1(10E)/11:1(	C30H56NO8P				39167		0.8615
Bonemar	7.62	464.3136	M-H	2	465.321927	PC(P-15:0/0:0)	C23H48NO6P			LMGP0107	40399		0.7239
Bonemar	9.74	787.5113	M+Cl	8	752.5356064	PA(22:2(13Z,16Z)/1	C43H77O8P		HMDB011	LMGP1001	81934	52929361	0.07233
Bonemar	11.98	871.5555	M-H	10	872.5543668	PGP(i-13:0/i-24:0)	C43H86O13P2		HMDB0116565			131823220	0.7007
Bonemar	11.85	815.5322	M+Cl	5	780.5669066	PA(20:3(5Z,8Z,11Z)	C45H81O8P		HMDB0115137			131822004	0.3669
Bonemar	11.94	956.5366	M+Cl	6	921.5731137	PS(DiMe(13,5)/DiM	C50H84NO12P		HMDB0061570			131770260	0.6374
Bonemar	12.05	878.5107	M-H	9	879.5261635	PS(DiMe(11,5)/Mor	C47H78NO12P		HMDB0061569			131770258	0.9645
Bonemar	8.77	456.2383	M-H	4	457.24375	Pro Trp Arg	C22H31N7O4				15873		0.08554
Bonemar	9.78	851.5626	M-H	3	852.572784	PI(19:0/16:0)	C44H85O13P			LMGP0601	80947		0.3340
Bonemar	10.86	845.5388	M-H	6	846.5410857	PG(22:6(4Z,7Z,10Z,	C48H79O10P		HMDB011	LMGP0401	79662	52927102	0.6311
Bonemar	11.94	1082.4793	M-H	5	1083.492181	Cyclolinopeptide F	C55H73N9O10S2		HMDB0036479		91603	6398538	0.9667
Bonemar	10.18	859.6777	M-H	5	860.6893907	TG(15:0/20:5(5Z,8Z	C56H92O6		HMDB0043729			131754848	0.7493
Bonemar	12	888.5605	M+Cl	9	853.583288	PS(22:4(7Z,10Z,13Z	C47H84NO10P			LMGP0301	78468		0.7380
Bonemar	6.83	438.295	M-H	9	439.3062747	PC(O-12:0/O-1:0)	C21H46NO6P				40186		0.6738
Bonemar	9.88	953.5928	M+Cl	4	918.619734	PI(22:0/18:2(9Z,12Z	C49H91O13P			LMGP0601	80701		0.8533
Bonemar	6.52	550.3478	M-H	7	551.3587	PE(11:0/11:0)[U]	C27H54NO8P				40416		0.1610
Bonemar	11.19	747.518	M-H	0	748.5254352	PG(18:0/16:1(9Z))	C40H77O10P		HMDB001	LMGP0401	61876	52927152	0.0292
Bonemar	9.78	850.5594	M-H	1	851.5676344	PE(MonoMe(11,5)/	C47H82NO10P		HMDB0061531			131770203	0.3133
Bonemar	8.48	403.1683	M+Cl	7	368.1963904	LysoPA(a-13:0/0:0)	C16H33O7P		HMDB0114760			131821852	0.3154
Bonemar	9.95	792.5488	M-H	8	793.5621551	PE(20:3(5Z,8Z,11Z)	C45H80NO8P		HMDB0009330		60726	53479756	0.6718
Bonemar	10.73	841.5034	M-H	1	842.5097856	PG(22:6(4Z,7Z,10Z,	C48H75O10P		HMDB0116604			131823247	0.5026
Bonemar	12	913.5755	M-H	6	914.5884294	PI(20:0/20:3(8Z,11Z	C49H87O13P		HMDB0009868		61253		0.6861
Bonemar	11.42	231.0866	M-H	3	232.0946882	Glycerol 1-propanoa	C10H16O6		HMDB0031640		87831	20111767	0.7860
Bonemar	7.33	349.2716	M-H	9	350.2820951	Furanoid acid - F6	C22H38O3		HMDB003	LMFA0115	45904	13963867	0.9259
Bonemar	9.98	390.2652	M-H	1	391.272259	N-arachidonoyl L-se	C23H37NO4			LMFA0802	75463		0.7316
Bonemar	6.74	555.2734	M-H	1	556.280123	PG(22:6(4Z,7Z,10Z,	C28H45O9P			LMGP0405	80004		0.09348
Bonemar	11.94	1023.5272	M+Cl	8	988.5653254	CL(8:0/8:0/8:0/i-14	C47H90O17P2		HMDB0116806			131823415	0.8146
Bonemar	11.4	466.307	M+Cl	5	431.33994	OMDM-1	C27H45NO3				45394		0.03322
Bonemar	12.19	813.5453	M+Cl	5	778.5723859	PG(i-12:0/i-24:0)	C42H83O10P		HMDB0116671			131823304	0.7655
Bonemar	9.93	834.5908	M-H	5	835.602095	LacCer(d18:0/14:0)	C44H85NO13			LMSP0501	83817		0.3054
Bonemar	11.76	751.5637	M+Cl	2	716.5954903	TG(14:1(9Z)/14:1(9	C45H80O6		HMDB004	LMGL0301	98479	14122758	0.04268
Bonemar	12.14	808.5097	M-H	5	809.5206842	PE(MonoMe(9,5)/D	C44H76NO10P		HMDB0061547			131770219	0.5152
Bonemar	9.79	902.5763	M+Cl	9	867.598935	PS(22:4(7Z,10Z,13Z	C48H86NO10P		HMDB011	LMGP0301	78470	52925916	0.3105
Bonemar	11.01	824.5614	M+Cl	5	789.588373	PS(P-16:0/21:0)	C43H84NO9P			LMGP0303	78763		0.7896
Bonemar	10.79	843.5176	M-H	2	844.5230667	PGP(i-13:0/i-22:0)	C41H82O13P2		HMDB0116564			131823219	0.7576
Bonemar	12.06	833.5157	M-H	3	834.5258291	PI(16:1(9Z)/18:1(11	C43H79O13P		HMDB0009800		61185		0.4389
Bonemar	9.9	788.5509	M-H	8	789.55198	GPSer(18:0/18:1(9Z	C42H80NO10P				40791		0.2689
Bonemar	6.34	233.1543	M-H	2	234.1619799	Cyclodehydroisolubi	C15H22O2		HMDB0037038		92017	14258974	0.9323
Bonemar	9.97	696.5017	M-H	6	697.5046402	PE(18:3(6Z,9Z,12Z)	C39H72NO7P		HMDB0009147		60554	53479681	0.5639
Bonemar	6.4	526.2922	M-H	3	527.3011893	LysoPE(0:0/22:5(4Z	C27H46NO7P		HMDB001	LMGP0205	62282	53480943	0.5900
Bonemar	8.73	337.2043	M-H	7	338.20932	Idebenone	C19H30O5				757		0.8126
Bonemar	10.07	796.5894	M-H	4	797.5934552	PC(18:0/19:3(9Z,12	C45H84NO8P				39519		0.7120
Bonemar	10.12	433.3279	M+Cl	9	398.354866	Fecosterol	C28H46O	C04525		LMST0103	83958	440371	0.02597
Bonemar	11.4	465.304	M-H	6	466.30831	Pristimerol	C30H42O4				44017		0.02831
Bonemar	11.51	479.2947	M+Cl	3	444.3239599	MG(0:0/24:6(6Z,9Z,	C28H44O4		HMDB0011560			131801685	0.3018
Bonemar	11.86	923.6429	M+Cl	9	888.681939	PG(22:0/22:1(11Z))	C50H97O10P			LMGP0401	79547		0.4454
Bonemar	12.14	809.5135	M-H	6	810.5258291	PI(16:0/16:0)	C41H79O13P		HMDB000	LMGP0601	40890		0.7716
Bonemar	9.98	457.2523	M-H	2	458.260316	N-(4-benzenesulfon	C26H38N2O3S			LMFA0802	36718		0.02888
Bonemar	10.44	1098.7493	M-H	5	1099.751143	MIPC(d18:0/26:0(2	C56H110NO17P			LMSP0303	103188		0.8428
Bonemar	12.19	812.5401	M-H	6	813.5519843	PS(20:3(8Z,11Z,14Z	C44H80NO10P		HMDB001	LMGP0301	78264	52925710	0.5865
Bonemar	10.49	1099.7607	M-H	2	1100.769889	NeuAcalpha2-3Galb	C59H108N2O16			LMSP0601	56053		0.7693
Bonemar	9.82	852.5717	M+Cl	5	817.5985406	PC(22:5(4Z,7Z,10Z,	C48H84NO7P		HMDB0008687		60133	53479337	0.5311
Bonemar	9.66	925.5571	M+Cl	1	890.5884294	PI(20:0/18:2(9Z,12Z	C47H87O13P		HMDB000	LMGP0601	61252		0.04225
Bonemar	11.4	1001.7386	M+Cl	2	966.767642	TG(17:0/22:5(7Z,10	C64H102O6			LMGL0301	38693		0.9091
Bonemar	9.77	685.5261	M-H	4	686.536276	SM(d18:2/15:0)	C38H75N2O6P			LMSP0301	83738		0.3953
Bonemar	7.38	583.3238	M-H	3	584.332554	PI(P-18:0/0:0)	C27H53O11P			LMGP0607	81202		0.9032
Bonemar	9.81	909.5575	M-H	8	910.5571293	PI(18:3(9Z,12Z,15Z)	C49H83O13P		HMDB0009865		61250		0.5496
Bonemar	9.86	555.4863	M+Cl	10	520.521931	Nonadecyl palmitole	C35H68O2			LMFA0701	75402		0.3790
Bonemar	10.03	718.5372	M-H	3	719.546505	PC(12:0/19:0)[U]	C39H78NO8P				39184		0.6778
Bonemar	10.02	719.5409	M+Cl	9	684.565254	Lysine-containing sic	C39H76N2O7			LMFA0000	45745		0.8301
Bonemar	0.33	957.798	M-H	7	958.7989411	TG(22:4(7Z,10Z,13Z	C63H106O6		HMDB0054658			131765230	0.1010
Bonemar	10	882.6153	M+Cl	0	847.645493	PC(O-20:0/22:6(4Z,	C50H90NO7P			LMGP0102	76475		0.4977
Bonemar	0.33	923.805	M-H	3	924.8145912	TG(15:0/20:4(8Z,11	C60H108O6		HMDB0043689			131754809	0.01024
Bonemar	9.88	568.4948	M-H	7	569.506251	DG(16:1(9Z)/0:0/16	C35H59D5O5			LMGL0201	4692		0.7569
Bonemar	9.94	791.5637	M+Cl	5	756.5904049	DG(11D5/11D5/0:0)	C47H80O7		HMDB0116379			131823034	0.6555
Bonemar	9.94	854.5887	M-H	3	855.598938	PS(20:3(8Z,11Z,14Z	C47H86NO10P			LMGP0301	78278		0.7018
Bonemar	10.13	822.5716	M-H	7	823.572723	PS(O-20:0/20:5(5Z,	C46H82NO9P			LMGP0302	78711		0.8840
Bonemar	9.83	853.5767	M+Cl	4	818.603689	PG(21:0/18:1(9Z))	C45H87O10P			LMGP0401	79510		0.6075
Bonemar	9.95	790.5616	M-H	2	791.5676348	PS(16:0/20:0)	C42H82NO10P		HMDB011	LMGP0301	40820	9547095	0.7071
Bonemar	7.41	480.3088	M-H	2	481.3168394	PC(15:0/0:0)[S]	C23H48NO7P				40283		0.1899
Bonemar	10.01	883.6197	M+Cl	0	848.650639	PG(21:0/20:0)	C47H93O10P			LMGP0401	79517		0.5194
Bonemar	10	804.574	M-H	2	805.58328	PS(18:0/19:0)[U]	C43H84NO10P				40822		0.6978
Bonemar	10.01	805.5778	M-H	3	806.5825566	PA(22:5(4Z,7Z,10Z,	C47H83O8P		HMDB0115367			131822124	0.7241
Bonemar	0.39	682.5219	M-H	7	683.53362	GlcCer(d15:2(4E,6E	C39H73NO8			LMSP0501	103298		0.06886
Bonemar	9.78	686.516	M-H	4	687.5202902	PE(15:0/P-18:1(11Z	C38H74NO7P		HMDB0008918		60361	53479585	0.3813
Bonemar	9.8	735.5221	M-H	5	736.5254357	PG(i-12:0/a-21:0)	C39H77O10P		HMDB0116658			131823291	0.5428
Bonemar	8.78	370.1908	M+Cl	4	335.2227831	Prostaglandin C1(1-	C20H31O4		HMDB0062756			45266617	0.1507
Bonemar	7.41	482.3132	M-H	2	483.3196	Tumonoic Acid H	C26H45NO7				65436		0.1355
Bonemar	9.91	802.5593	M-H	1	803.5676348	PS(15:0/22:1(13Z))	C43H82NO10P		HMDB0112335			131819718	0.4086
Bonemar	9.91	803.5629	M-H	4	804.5669066	PA(22:2(13Z,16Z)/2	C47H81O8P		HMDB011	LMGP1001	81950	52929377	0.3403
Bonemar	8.79	369.1885	M-H	2	370.19647	Ser His Lys	C15H26N6O5				19816		0.0957
Bonemar	10.05	778.5623	M+Cl	2	743.591135	GlcCer(d14:1(4E)/2	C42H81NO9			LMSP0501	103306		0.8079
Bonemar	6.58	144.1029	M-H	1	145.11028	N-Methylisoleucine	C7H15NO2				44542		0.008484
Bonemar	6.56	445.3219	M+Cl	6	410.354866	Corbisterol	C29H46O			LMST0104	84139		0.04999
Bonemar	7.4	548.296	M-H	6	549.306672	PS(20:2(11Z,14Z)/0	C26H48NO9P			LMGP0305	78848		0.2137
Bonemar	0.33	1162.7571	M+Cl	5	1127.782443	MIPC(d20:0/26:0(2	C58H114NO17P			LMSP0303	103218		0.5201
Bonemar	10.05	779.5657	M+Cl	9	744.603294	PA(O-18:0/22:1(11	C43H85O7P			LMGP1002	82185		0.7929
Bonemar	8.76	641.4277	M+Cl	7	606.462444	PA(O-16:0/14:0)	C33H67O7P			LMGP1002	82150		0.02169
Bonemar	0.33	1169.7585	M-H	3	1170.768776	CL(a-13:0/a-13:0/a-	C60H116O17P2		HMDB0090576			131797123	0.02018
Bonemar	6.03	275.1638	M-H	5	276.1725446	Panaquinquecol 2	C17H24O3		HMDB003	LMFA0500	93549	14827985	0.2613
Bonemar	7.08	249.113	M-H	1	250.1205091	Ubiquinone-1	C14H18O4		HMDB0002012		45118	4462	0.126
Bonemar	7.43	640.2923	M-H	2	641.2982154	S-(PGA2)-glutathion	C30H47N3O10S		HMDB0013062			53481602	0.6463
Bonemar	0.33	1101.7696	M-H	8	1102.785539	NeuAcalpha2-3Galb	C59H110N2O16			LMSP0601	56051		0.03721
Bonemar	10.85	767.5703	M+Cl	3	732.603294	PA(O-18:0/21:0)	C42H85O7P			LMGP1002	82184		0.2749
Bonemar	8.77	455.2387	M-H	6	456.24882	PG(14:0/0:0)[U]	C20H41O9P				40873		0.02529
Bonemar	0.33	931.7929	M+Cl	4	896.8196766	TG(18:2(9Z,12Z)/a-	C59H108O5		HMDB0052833			131763484	0.1067
Bonemar	10	745.5555	M+Cl	2	710.584926	DG(21:0/22:6(4Z,7Z	C46H78O5			LMGL0201	4665		0.7948
Bonemar	10.06	806.5839	M+Cl	0	771.6141906	PC(P-16:0/20:1(11Z	C44H86NO7P		HMDB001	LMGP0103	62046	52923904	0.8943
Bonemar	6.85	536.2276	M-H	5	537.23761	Trp Phe Trp	C31H31N5O4				20466		0.9689
Bonemar	10.03	744.5522	M-H	4	745.5621551	PE(22:1(13Z)/14:0)	C41H80NO8P		HMDB0009514		60903	53479852	0.6369
Bonemar	8.87	655.4409	M-H	10	656.4417061	PA(18:3(6Z,9Z,12Z)	C36H65O8P		HMDB011	LMGP1001	81534	52928961	0.01353
Bonemar	10.07	832.6024	M-H	6	833.614585	PS(15:0/24:0)	C45H88NO10P		HMDB0112340			131819721	0.5826
Bonemar	0.33	971.8089	M-H	2	972.814592	TG(17:1(9Z)/22:3(1	C64H108O6			LMGL0301	38442		0.212
Bonemar	9.77	877.5759	M-H	6	878.588434	PI(15:1(9Z)/22:0)	C46H87O13P			LMGP0601	80177		0.6072
Bonemar	0.34	897.7988	M-H	8	898.7989411	TG(15:0/18:2(9Z,12	C58H106O6		HMDB0043407			131754528	0.05483
Bonemar	9.99	697.4977	M+Cl	5	662.5250418	PA(O-16:0/18:0)	C37H75O7P		HMDB001	LMGP1002	82228	52929644	0.09473
Bonemar	12	883.5326	M-H	2	884.5414792	PI(22:3(10Z,13Z,16	C47H81O13P		HMDB0009908		61293		0.2674
Bonemar	7.76	582.3721	M+Cl	5	547.4001751	PC(O-3:1(1E)/O-18:	C29H58NO6P				40245		0.8499
Bonemar	0.33	970.8121	M-H	9	971.8282061	PC(23:0/26:0)[U]	C57H114NO8P				39880		0.1943
Bonemar	12.03	857.5171	M-H	2	858.5258291	PI(18:2(9Z,12Z)/18:	C45H79O13P		HMDB000	LMGP0601	61235		0.2094
Bonemar	6.25	525.2799	M+Cl	9	490.3059409	LysoPA(22:2(13Z,16	C25H47O7P		HMDB0114751			131821851	0.4461
Bonemar	10.08	833.607	M-H	1	834.6138568	PA(24:0/22:5(7Z,10	C49H87O8P		HMDB0115451			131822189	0.6505
Bonemar	6.78	478.2929	M-H	2	479.3011893	PE(18:1(9Z)/0:0)	C23H46NO7P		HMDB001	LMGP0205	40778	58177709	0.5259
Bonemar	9.97	1009.8035	M+Cl	4	974.8302413	TG(15:0/22:6(4Z,7Z	C64H110O6		HMDB0043785			131754903	0.3764
Bonemar	9.83	827.5625	M+Cl	6	792.5880359	PG(a-13:0/i-24:0)	C43H85O10P		HMDB0116653			131823286	0.7563
Bonemar	6.25	524.2773	M-H	2	525.2855393	PE(22:6(4Z,7Z,10Z,	C27H44NO7P		HMDB001	LMGP0205	77679	52925132	0.6348
Bonemar	8.49	733.3225	M+Cl	2	698.3513564	(15a,20R)-Dihydrox	C35H54O14		HMDB0033168		89045	85220526	0.7780
Bonemar	10	830.5896	M-H	2	831.598935	PS(15:0/24:1(15Z))	C45H86NO10P		HMDB0112341			131819722	0.9531
Bonemar	0.33	1004.8059	M-H	1	1005.812556	PC(26:2(5Z,9Z)/26:	C60H112NO8P				39946		0.1545
Bonemar	9.96	771.5573	M-H	4	772.561824	PG(P-20:0/17:2(9Z	C43H81O9P			LMGP0403	79970		0.2258
Bonemar	9	670.46	M+Cl	3	635.4889901	lysoPC(26:0)	C34H70NO7P		HMDB0029205		86087	131750810	0.002263
Bonemar	7.82	578.3453	M-H	2	579.3536	PC(16:0/4:0(CHO))1-	C44H81NO8P						0.5839
Bonemar	12.01	881.5173	M-H	1	882.525834	PI(18:4(6Z,9Z,12Z,1	C47H79O13P			LMGP0601	80436		0.9411
Bonemar	7.77	524.2983	M-H	2	525.30667	PS(18:0/0:0)[U]	C24H48NO9P				40831		0.1456
Bonemar	9.76	876.5722	M-H	4	877.583288	PS(21:0/22:6(4Z,7Z	C49H84NO10P			LMGP0301	3872		0.4594
Bonemar	9.83	826.5591	M-H	2	827.567638	PS(17:1(9Z)/22:2(1	C45H82NO10P			LMGP0301	77939		0.8434
Bonemar	5.99	416.2961	M+Cl	6	381.324294	N-stearoyl proline	C23H43NO3			LMFA0802	75501		0.01648
Bonemar	0.39	692.4875	M-H	0	693.494473	PS(O-16:0/14:0)	C36H72NO9P			LMGP0302	78650		0.09826
Bonemar	12.18	782.496	M-H	2	783.50503	PS(18:2(9Z,12Z)/18	C42H74NO10P				40801		0.3723
Bonemar	9	669.4572	M+Cl	9	634.493744	PA(O-20:0/12:0)	C35H71O7P			LMGP1002	82188		0.002323
Bonemar	8.77	459.2342	M-H	4	460.24342	Trp Gln Lys	C22H32N6O5				16113		0.5593
Bonemar	10.01	831.5932	M-H	3	832.5982067	PA(24:1(15Z)/22:5(	C49H85O8P		HMDB0115477			131822214	0.9291
Bonemar	11.4	533.2836	M-H	9	534.295773	PG(20:3(8Z,11Z,14Z	C26H47O9P			LMGP0405	80013		0.8586
Bonemar	9.92	708.5228	M+Cl	6	673.54927	GlcCer(d18:0/14:0)	C38H75NO8			LMSP0501	53987		0.4419
Bonemar	10.05	716.5223	M-H	2	717.5308549	PC(15:0/16:1(7Z))[	C39H76NO8P				39276		0.6102
Bonemar	9	671.4613	M-H	7	672.4730057	PA(16:0/18:2(9Z,12	C37H69O8P		HMDB000	LMGP1001	40921	9547167	0.01180
Bonemar	8.68	371.2784	M-H	5	372.2875744	DG(10:0/0:0/8:0)	C21H40O5		HMDB0092960			73416316	0.1842
Bonemar	8.98	691.4387	M-H	6	692.4417061	PA(18:3(9Z,12Z,15Z	C39H65O8P		HMDB011	LMGP1001	40914	9547161	0.001622
Bonemar	12.18	806.4959	M-H	2	807.5050341	PS(16:0/22:6(4Z,7Z	C44H74NO10P		HMDB001	LMGP0301	77707	46891805	0.9012
Bonemar	9.83	863.5835	M-H	3	864.588039	PG(21:0/22:6(4Z,7Z	C49H85O10P			LMGP0401	3875		0.1643
Bonemar	12.12	832.5101	M-H	4	833.5206842	PS(18:1(9Z)/22:6(4	C46H76NO10P		HMDB001	LMGP0301	77709	52925155	0.3498
Bonemar	6.63	452.2775	M-H	2	453.2855393	PC(13:0/0:0)[U]	C21H44NO7P				40277		0.5127
Bonemar	7.82	666.3963	M-H	5	667.4003723	Gluten exorphin C	C29H57N5O12		HMDB0059796			131769784	0.8883
Bonemar	9.64	1080.5033	M-H	2	1081.512916	Cyclolinopeptide H	C56H75N9O9S2		HMDB0036553		91634	6398540	0.8977
Bonemar	6.75	546.2807	M-H	6	547.291022	PS(20:3(8Z,11Z,14Z	C26H46NO9P			LMGP0305	78849		0.7902
Bonemar	8.77	457.2268	M-H	8	458.2304534	Hydroxystrobilurin [	C26H34O7		HMDB0034760		90186	131751621	0.3637
Bonemar	9.71	810.5322	M-H	4	811.5363342	PS(18:0/20:4(5Z,8Z	C44H78NO10P		HMDB001	LMGP0301	40824	91746195	0.6853
Bonemar	8.55	429.1742	M-H	9	430.18524	Pro Trp Glu	C21H26N4O6				16156		0.7539
Bonemar	12.07	830.4945	M-H	4	831.5050341	PS(22:6(4Z,7Z,10Z,1	C46H74NO10P		HMDB001	LMGP0301	78493	52925939	0.3177
Bonemar	12.17	807.5	M-H	4	808.5101791	PI(16:1(9Z)/16:0)	C41H77O13P		HMDB000	LMGP0601	61182		0.8651
Bonemar	6.83	516.3055	M-H	8	517.3168394	PC(18:3(6Z,9Z,12Z)	C26H48NO7P				40303		0.5115
Bonemar	6.82	571.2877	M-H	2	572.296168	PI(16:0/0:0)	C25H49O12P			LMGP0605	46744		0.9145
Bonemar	8.77	642.431	M+Cl	6	607.45769	PC(24:0/0:0)	C32H66NO7P		HMDB001	LMGP0105	61714	24779481	0.02671
Bonemar	8.03	427.1616	M-H	1	428.16825	Furcatin	C20H28O11	C10458			68339	442789	0.4955

1. Metabolites affected by drug only

Day 4

ami_50/Vehicle

Type	RT	erimental.r	Adduct	PPM.Error	Molecular.Weig	Name	Formula	Kegg	HMDB	LipidMaps	Metlin	PubChem	p.value	FDR
Bonemar	12.19	789.5446	M-H	1	790.551259	PA(21:0/22:6(4Z,7Z	C46H79O8P			LMGP1001	3883		0.84574	0.95257
Bonemar	0.33	1005.8029	M+Cl	2	970.8353266	TG(22:5(4Z,7Z,10Z	C65H110O5		HMDB0055084			131765628	0.19839	0.58988
Bonemar	0.33	1183.7697	M-H	6	1184.784426	CL(8:0/10:0/11:0/2	C61H118O17P2		HMDB0117489			131824098	0.15033	0.53408
Bonemar	11.94	1103.4733	M-H	8	1104.471771	PIP3(18:0/18:1(11Z	C45H88O22P4		HMDB0010152		61530		0.34025	0.74003
Bonemar	7.43	558.3296	M+Cl	7	523.3637704	enantio-PAF C-16	C26H54NO7P				43413		0.82098	0.94987
Bonemar	8.59	634.4416	M-H	6	635.4526046	PC(10:0/15:0)[U]	C33H66NO8P				39129		0.54928	0.84984
Bonemar	11.37	1027.7487	M+Cl	4	992.783292	TG(19:0/22:6(4Z,7Z	C66H104O6			LMGL0301	38979		0.79158	0.93487
Bonemar	8.08	380.1863	M-H	9	381.18999	Ser Tyr Ile	C18H27N3O6				18072		0.32398	0.73011
Bonemar	9.96	954.5936	M+Cl	7	919.6302351	PS(22:5(7Z,10Z,13Z	C52H90NO10P		HMDB0112863			131819994	0.74505	0.92368
Bonemar	7.43	570.3665	M+Cl	6	535.4001751	PC(O-18:0/O-2:1(1E	C28H58NO6P				40228		0.84607	0.95257
Bonemar	5.9	391.2829	M-H	6	392.2926598	Hyodeoxycholic acid	C24H40O4		HMDB0000733		5701	9963687	0.033265	0.45321
Bonemar	8.56	665.4141	M-H	7	666.426056	PA(14:1(9Z)/20:4(8	C37H63O8P		HMDB0114802			131821871	0.70042	0.90772
Bonemar	8.76	965.4064	M-H	10	966.42292	Cys-Asn-Pro-Gly-Tyr	C40H62N12O14S				85178		0.074403	0.45878
Bonemar	7.43	508.3397	M-H	2	509.3481395	PC(O-15:0/2:0)[U]	C25H52NO7P				40054		0.95894	0.98585
Bonemar	10.88	770.5076	M+Cl	4	735.541423	PS(O-16:0/17:0)	C39H78NO9P			LMGP0302	78728		0.42434	0.79323
Bonemar	12.01	887.5569	M+Cl	1	852.588039	PG(20:4(5Z,8Z,11Z	C48H85O10P			LMGP0401	79470		0.55834	0.85326
Bonemar	12.28	262.0947	M-H	7	263.1037306	N(2)-phenylacetyl-L-	C13H15N2O4		HMDB0062645			131770469	0.29371	0.69774
Bonemar	8.77	780.5148	M-H	5	781.525772	PE(18:0/20:4(5Z,8Z	C43H76NO9P			LMGP2001	82377		0.49281	0.82754
Bonemar	8.53	472.2211	M+Cl	6	437.254242	PC(O-10:1(9E)/2:0)	C20H40NO7P			LMGP0102	40006		0.02393	0.45321
Bonemar	10.89	769.5034	M-H	1	770.5097851	PG(18:1(9Z)/18:3(9	C42H75O10P		HMDB001	LMGP0401	61908	52927145	0.44752	0.80327
Bonemar	9.68	1014.537	M-H	9	1015.535194	delta5-Demissine	C50H81NO20		HMDB0033029		88921	131751363	0.59969	0.86572
Bonemar	9.64	848.5432	M-H	2	849.551988	PS(22:6(4Z,7Z,10Z	C47H80NO10P			LMGP0301	78497		0.58266	0.85858
Bonemar	8.88	656.4417	M+Cl	2	621.473342	PE(O-16:0/12:0)	C33H68NO7P			LMGP0202	77500		0.53788	0.84675
Bonemar	0.34	939.7803	M-H	1	940.7883764	TG(20:4(5Z,8Z,11Z	C63H104O5		HMDB0054340			131764937	0.040924	0.45321
Bonemar	11.93	967.5156	M+Cl	5	932.541484	PI(22:4(7Z,10Z,13Z	C51H81O13P			LMGP0601	80802		0.9481	0.98351
Bonemar	9.87	762.5078	M-H	0	763.5152049	PE(16:1(9Z)/22:5(7Z	C43H74NO8P		HMDB0008978		60407	53479602	0.94037	0.98032
Bonemar	9.87	763.5109	M+Cl	8	728.5356064	PA(14:1(9Z)/24:1(1	C41H77O8P		HMDB0114811			131821876	0.94027	0.98032
Bonemar	9.63	849.5467	M-H	4	850.557134	PI(16:0/19:1(9Z))	C44H83O13P			LMGP0601	80186		0.49801	0.82847
Bonemar	9.96	906.5852	M+Cl	8	871.6091057	PE-NMe2(22:2(13Z	C51H86NO8P		HMDB0114492			131821599	0.78178	0.93257
Bonemar	8	372.2372	M-H	2	373.24375	Arg Lys Ala	C15H31N7O4				16523		0.17986	0.57117
Bonemar	11.89	965.5843	M+Cl	5	930.619734	PI(19:1(9Z)/22:2(13	C50H91O13P			LMGP0601	80498		0.88512	0.96365
Bonemar	12.01	885.5488	M-H	1	886.5571293	PI(16:0/22:4(7Z,10Z	C47H83O13P		HMDB000	LMGP0601	61179		0.53255	0.84594
Bonemar	8.57	381.1765	M-H	4	382.18524	Lys-Lys-OH	C17H26N4O6				65269		0.89778	0.96636
Bonemar	0.46	124.9646	M+Cl	1	89.99530855	Oxalic acid	C2H2O4	C00209	HMDB0002329		113	18676629	0.27976	0.67848
Bonemar	11.16	477.2903	M+Cl	3	442.319543	N-palmitoyl tryptop	C27H42N2O3			LMFA0802	75477		0.61629	0.87364
Bonemar	6.83	522.2817	M-H	4	523.29102	PS(18:1(9Z)/0:0)[U]	C24H46NO9P				40832		0.58938	0.85882
Bonemar	10.14	899.6919	M+Cl	2	864.7206908	TG(15:0/20:2n6/18	C56H96O6		HMDB0043476			131754596	0.48999	0.82737
Bonemar	6.66	482.3092	M+Cl	4	447.338232	N-docosanoyl taurin	C24H49NO4S			LMFA0802	75465		0.48887	0.82737
Bonemar	9.77	485.2799	M-H	9	486.291616	N-(2'-(4-benzenesul	C28H42N2O3S			LMFA0802	36721		0.77888	0.93155
Bonemar	8.57	382.1775	M-H	1	383.18451	Phe Ala Phe	C21H25N3O4				17644		0.77653	0.93155
Bonemar	9.95	955.6117	M+Cl	8	920.635384	PI(18:1(9Z)/22:0)	C49H93O13P			LMGP0601	80950		0.55799	0.85326
Bonemar	6.66	542.3343	M+Cl	8	507.368875	PC(O-18:1(9E)/0:0)	C26H54NO6P				40373		0.79136	0.93487
Bonemar	10.09	807.5893	M-H	2	808.5982067	PA(22:2(13Z,16Z)/2	C47H85O8P		HMDB011	LMGP1001	81949	52929376	0.68529	0.90264
Bonemar	8.59	663.4183	M-H	3	664.42336	Prostaglandin F2a-b	C35H60N4O6S				44963		0.68349	0.90243
Bonemar	11.94	1178.4546	M+Cl	0	1143.485725	(17Z)-hexacosenoyl-	C47H84N7O17P3S			LMFA07050099			0.32172	0.72747
Bonemar	8.57	442.1886	M+Cl	6	407.21687	Phe Asn Lys	C19H29N5O5				16043		0.027841	0.45321
Bonemar	6.27	569.2687	M-H	5	570.272987	Pipercyclobutanami	C34H38N2O6		HMDB0036360		91504	131751971	0.83375	0.95257
Bonemar	10.68	685.4796	M-H	3	686.4886562	PA(20:2(11Z,14Z)/1	C38H71O8P		HMDB011	LMGP1001	81726	52929153	0.08843	0.47457
Bonemar	9.94	765.5448	M-H	1	766.551259	PA(22:4(7Z,10Z,13Z	C44H79O8P			LMGP1001	81969		0.79954	0.93889
Bonemar	11.96	953.5357	M+Cl	0	918.566439	Megalomicin B	C46H82N2	C11986		LMPK0400	40991		0.38388	0.76914
Bonemar	9.67	875.5619	M-H	4	876.572784	PI(15:1(9Z)/22:1(11	C46H85O13P			LMGP0601	80178		0.31019	0.71235
Bonemar	9.97	715.5118	M+Cl	6	680.5379754	DG(18:2n6/0:0/22:	C44H72O5		HMDB0056294			131801994	0.61817	0.87451
Bonemar	11.95	949.5096	M-H	10	950.5074166	PGP(22:5(4Z,7Z,10Z	C50H80O13P2		HMDB0116494			131823149	0.78231	0.93257
Bonemar	10.8	794.5172	M+Cl	8	759.541423	PS(P-16:0/19:1(9Z))	C41H78NO9P			LMGP0303	78757		0.4858	0.82663
Bonemar	11.98	1163.4483	M+Cl	6	1128.471771	PIP3(18:2(9Z,12Z)/2	C47H88O22P4		HMDB0010162		61540		0.35481	0.7491
Bonemar	11.94	1085.4719	M-H	6	1086.485943	LPIM3(18:0/0:0)	C45H83O27P			LMGP15040019			0.34495	0.74175
Bonemar	12.14	835.5307	M-H	4	836.5414792	PI(18:1(9Z)/16:0)	C43H81O13P		HMDB000	LMGP0601	61219		0.74232	0.92186
Bonemar	12.17	836.5393	M-H	6	837.5519843	PE(DiMe(9,5)/Mono	C46H80NO10P		HMDB0061513			131770185	0.86404	0.95487
Bonemar	6.67	530.3009	M+Cl	2	495.3324895	PC(O-14:0/2:0)[U]	C24H50NO7P				40049		0.7275	0.91811
Bonemar	7.47	422.2287	M-H	6	423.2385891	PE(14:1(9Z)/0:0)	C19H38NO7P		HMDB001	LMGP0205	77698	52925151	0.95618	0.98585
Bonemar	8.08	379.1874	M-H	5	380.1963904	PA(14:1(9Z)/0:0)	C17H33O7P		HMDB006	LMGP1005	82357	52929768	0.22764	0.62456
Bonemar	12.18	837.5442	M-H	7	838.5571293	PI(18:0/16:0)	C43H83O13P		HMDB000	LMGP0601	61190		0.97888	0.9928
Bonemar	7.19	450.2994	M-H	1	451.306277	PC(O-14:1(1E)/0:0)	C22H46NO6P			LMGP0107	40397		0.26588	0.66347
Bonemar	8.75	393.2795	M+Cl	5	358.3083098	MG(0:0/18:0/0:0)	C21H42O4		HMDB0011535		62319	79075	0.78794	0.93462
Bonemar	11.95	1194.4608	M+Cl	9	1159.48064	CoA(26:0(3Ke))	C47H84N7O18P3S				97426		0.4064	0.78541
Bonemar	6.84	436.2823	M-H	2	437.2906247	PE(P-16:0/0:0)	C21H44NO6P		HMDB001	LMGP0207	46719	42607469	0.9788	0.9928
Bonemar	12.19	839.5613	M+Cl	5	804.588039	PG(21:0/17:1(9Z))	C44H85O10P			LMGP0401	79508		0.65836	0.88811
Bonemar	12.19	838.5572	M-H	4	839.5676348	PS(20:3(5Z,8Z,11Z)	C46H82NO10P		HMDB0112606			131819835	0.71989	0.91723
Bonemar	12.17	834.5262	M-H	3	835.5363342	PS(18:0/22:6(4Z,7Z	C46H78NO10P		HMDB001	LMGP0301	40825	91746196	0.60606	0.86882
Bonemar	7.61	532.3015	M+Cl	6	497.33525	Tumonoic Acid I	C27H47NO7				65437		0.44402	0.80327
Bonemar	10.83	793.5047	M-H	3	794.5097851	PG(18:3(6Z,9Z,12Z)	C44H75O10P		HMDB001	LMGP0401	61940	52926653	0.59954	0.86572
Bonemar	9.96	776.5502	M-H	7	777.5519848	PS(15:0/20:0)	C41H80NO10P		HMDB011	LMGP0301	77814	52925260	0.97354	0.99095
Bonemar	7.69	694.4246	M+Cl	4	659.4526046	PE-NMe2(14:1(9Z)/	C35H66NO8P		HMDB0116710			131823339	0.2223	0.61793
Bonemar	7.86	395.222	M-H	4	396.2276905	PA(15:0/0:0)	C18H37O7P		HMDB006	LMGP1005	82356	52929767	0.058441	0.45321
Bonemar	10.16	785.6791	M+Cl	1	750.7101261	DG(i-24:0/0:0/a-21:	C48H94O5		HMDB0094571			131800692	0.046625	0.45321
Bonemar	12.1	855.5057	M-H	3	856.510184	PI(22:4(7Z,10Z,13Z	C45H77O13P			LMGP0601	80781		0.40682	0.78541
Bonemar	9.89	721.5049	M-H	3	722.5097856	PG(16:0/16:0)	C38H75O10P		HMDB001	LMGP0401	61847	446440	0.44405	0.80327
Bonemar	12.03	916.4885	M+Cl	2	881.5206846	PS(22:6(4Z,7Z,10Z	C50H76NO10P		HMDB0112878			131820000	0.068659	0.45864
Bonemar	10.06	820.5648	M+Cl	2	785.5934552	PC(18:2(9Z,12Z)/18	C44H84NO8P		HMDB000	LMGP0101	59581	15378085	0.014142	0.45321
Bonemar	8.77	441.23	M-H	9	442.233173	PG(13:0/0:0)	C19H39O9P			LMGP0405	46732		0.35406	0.7491
Bonemar	9.97	777.5537	M-H	2	778.559501	MGDG(18:2(9Z,12Z	C45H78O10			LMGL0501	46659		0.86985	0.95892
Bonemar	9.78	926.565	M+Cl	4	891.598935	PS(22:2(13Z,16Z)/2	C50H86NO10P		HMDB011	LMGP0301	78449	52925895	0.19846	0.58988
Bonemar	12	943.5047	M+Cl	7	908.541484	PI(22:4(7Z,10Z,13Z	C49H81O13P			LMGP0601	80792		0.70492	0.90821
Bonemar	9.6	823.5297	M-H	5	824.541484	PI(17:0/16:0)	C42H81O13P			LMGP0601	80219		0.40224	0.78349
Bonemar	11.07	1152.7348	M-H	7	1153.733566	Fucalpha1-2Galalph	C58H107NO21			LMSP0506	55921		0.56037	0.85326
Bonemar	9.88	929.5866	M+Cl	3	894.619734	PI(20:0/18:0)	C47H91O13P			LMGP0601	80946		0.51777	0.83872
Bonemar	12.16	774.5667	M-H	2	775.572723	PS(P-16:0/20:0)	C42H82NO9P			LMGP0303	78758		0.43528	0.80192
Bonemar	11.98	914.5506	M+Cl	4	879.5778051	PC(22:6(4Z,7Z,10Z	C52H82NO8P		HMDB0008747		60193	53479417	0.47966	0.82376
Bonemar	11.64	474.3726	M+Cl	1	439.4025446	Pentacosanoylglycin	C27H53NO3		HMDB0013297			53481662	0.8474	0.95257
Bonemar	9.6	822.5276	M-H	2	823.5363342	PE(DiMe(9,5)/DiMe(	C45H78NO10P		HMDB0061511			131770183	0.58867	0.85861
Bonemar	6.28	589.3297	M+Cl	3	554.358374	PG(21:0/0:0)	C27H55O9P			LMGP0405	80017		0.0045061	0.45321
Bonemar	9.76	659.5118	M-H	2	660.520626	PE-Cer(d14:1(4E)/2	C36H73N2O6P			LMSP0302	103044		0.30338	0.70722
Bonemar	9.92	738.5079	M-H	0	739.5152049	PE(18:2(9Z,12Z)/18	C41H74NO8P		HMDB000	LMGP0201	40509	91746202	0.10547	0.48078
Bonemar	7	592.3596	M-H	4	593.3692689	POV-PC	C29H56NO9P				45370		0.13328	0.50716
Bonemar	8.77	440.2221	M-H	7	441.22637	Tyr Ile Phe	C24H31N3O5				15705		0.29538	0.69872
Bonemar	6.1	538.3121	M-H	5	539.322322	PS(19:0/0:0)	C25H50NO9P			LMGP0305	78855		0.24334	0.63881
Bonemar	9.97	749.5334	M+Cl	5	714.567576	PE-Cer(d14:1(4E)/2	C40H79N2O6P			LMSP0302	103051		0.44723	0.80327
Bonemar	9.93	739.512	M+Cl	10	704.5356064	PA(i-12:0/i-24:0)	C39H77O8P		HMDB0115792			131822448	0.24507	0.64082
Bonemar	10.17	857.6687	M-H	3	858.6737406	TG(15:0/20:5(5Z,8Z	C56H90O6		HMDB0043737			131754856	0.73889	0.92092
Bonemar	10.19	671.4756	M-H	2	672.484241	PE-Cer(d14:2(4E,6E	C36H69N2O7P			LMSP0302	103108		0.35295	0.7491
Bonemar	12.05	987.4829	M-H	2	988.4879095	Medicagenic acid 3-(	C48H76O21		HMDB0039487			4483634	0.45917	0.80834
Bonemar	0.33	995.7751	M+Cl	9	960.8145912	TG(20:3n6/20:1(11Z	C63H108O6		HMDB0053785			131764423	0.16666	0.55633
Bonemar	11.9	873.5583	M-H	10	874.557134	PI(15:1(9Z)/22:2(13	C46H83O13P			LMGP0601	80179		0.78183	0.93257
Bonemar	10.2	799.6607	M+Cl	2	764.6893907	TG(15:0/15:0/15:0)	C48H92O6		HMDB004	LMGL0301	98480	4290702	0.98143	0.99302
Bonemar	9.95	747.5481	M-H	9	748.561824	PG(P-20:0/15:0)	C41H81O9P			LMGP0403	79964		0.57945	0.85684
Bonemar	11.94	903.5159	M-H	0	904.5230657	PGP(18:0/22:5(7Z,1	C46H82O13P2		HMDB0013515			53481833	0.84576	0.95257
Bonemar	7.63	622.3683	M-H	7	623.3798	PC(16:0/6:0(COOH))	C30H58NO10P						0.45653	0.80824
Bonemar	12.2	732.484	M-H	3	733.489384	PS(18:0/14:1(9Z))	C38H72NO10P		HMDB001	LMGP0301	77973	52925419	0.64123	0.88077
Bonemar	11.92	1087.4915	M-H	5	1088.503953	Medicagenic acid 3-(	C52H80O24		HMDB0039292		93879	14779968	0.63008	0.87876
Bonemar	0.33	885.7954	M-H	4	886.7989411	TG(16:1(9Z)/22:0/1	C57H106O6		HMDB0048523			131759350	0.26899	0.66703
Bonemar	10.62	710.4811	M-H	6	711.4839047	PE(20:3(8Z,11Z,14Z	C39H70NO8P		HMDB000	LMGP0201	60746	52924606	0.14748	0.53034
Bonemar	7.11	347.2562	M-H	9	348.266445	Dinorlithocholic acid	C22H36O3			LMST04050010			0.4533	0.80677
Bonemar	6.61	343.2264	M-H	4	344.2351449	Testosterone propio	C22H32O3	C08158	HMDB001	LMST0202	2783	5995	0.047381	0.45321
Bonemar	6.68	319.2266	M-H	4	320.2351449	(+/-)5,6-EpETrE	C20H32O3	C14768	HMDB000	LMFA0308	3851	5283202	0.21823	0.61369
Bonemar	6.61	344.2286	M-H	5	345.23761	Lys Lys Ala	C15H31N5O4				16558		0.033368	0.45321
Bonemar	9.72	760.4984	M-H	8	761.4995548	PE(20:4(8Z,11Z,14Z	C43H72NO8P		HMDB0009424		60820	53479808	0.24201	0.63827
Bonemar	10.92	795.5186	M-H	1	796.5254352	PG(18:1(9Z)/20:4(5	C44H77O10P		HMDB001	LMGP0401	61911	52927144	0.25163	0.64973
Bonemar	10.93	796.5244	M+Cl	3	761.557073	PS(P-16:0/19:0)	C41H80NO9P			LMGP0303	78756		0.33816	0.73783
Bonemar	9.88	838.5814	M+Cl	10	803.604023	PS(P-16:0/22:0)	C44H86NO9P			LMGP0303	78764		0.3144	0.71885
Bonemar	10.1	780.5718	M+Cl	5	745.5985084	PC(O-18:1(9Z)/16:0	C42H84NO7P				40014		0.88638	0.96396
Bonemar	9.73	824.5435	M-H	1	825.551988	PS(22:4(7Z,10Z,13Z	C45H80NO10P			LMGP0301	78459		0.97006	0.98929
Bonemar	9.73	825.547	M+Cl	7	790.572389	PG(15:0/22:1(11Z))	C43H83O10P			LMGP0401	78982		0.92639	0.97448
Bonemar	6.74	496.2674	M-H	1	497.275372	PS(16:0/0:0)	C22H44NO9P			LMGP0305	40830		0.54497	0.84805
Bonemar	9.71	799.5338	M-H	7	800.5356064	PA(22:2(13Z,16Z)/2	C47H77O8P		HMDB011	LMGP1001	81951	52929378	0.42973	0.79884
Bonemar	10.01	766.5383	M-H	1	767.546505	PE(20:0/18:4(6Z,9Z	C43H78NO8P		HMDB000	LMGP0201	40512	9546815	0.36188	0.75569
Bonemar	10.01	767.5414	M+Cl	7	732.5669066	PA(a-25:0/13:0)	C41H81O8P		HMDB0115747			131822403	0.41792	0.78813
Bonemar	10.72	842.508	M+Cl	3	807.541423	PE(18:0/22:6(4Z,7Z	C45H78NO9P			LMGP2002	82385		0.45739	0.80824
Bonemar	10.65	815.4884	M-H	5	816.4917666	PGP(i-12:0/i-21:0)	C39H78O13P2		HMDB0116545			131823200	0.40349	0.78362
Bonemar	12.11	828.486	M-H	5	829.489384	PS(22:6(4Z,7Z,10Z	C46H72NO10P		HMDB001	LMGP0301	78495	52925941	0.78282	0.93276
Bonemar	9.79	837.5654	M-H	0	838.572389	PG(20:5(5Z,8Z,11Z,	C47H83O10P			LMGP0401	79497		0.047689	0.45321
Bonemar	10.12	808.5954	M+Cl	5	773.6298407	PC(P-16:0/20:0)	C44H88NO7P		HMDB001	LMGP0103	62045	52923902	0.77372	0.93155
Bonemar	9.51	784.496	M-H	5	785.4995548	PE(18:4(6Z,9Z,12Z	C45H72NO8P		HMDB0009209		60615	53479702	0.10391	0.47974
Bonemar	12.1	856.5116	M-H	2	857.5206842	PS(22:6(4Z,7Z,10Z	C48H76NO10P		HMDB001	LMGP0301	78502	52925948	0.98324	0.99302
Bonemar	9.85	928.5753	M+Cl	10	893.6145846	PE(MonoMe(13,5)/	C50H88NO10P		HMDB0061535			131770207	0.41218	0.7869
Bonemar	6.75	531.2784	M+Cl	6	496.3121664	Postin	C22H40N8O5		HMDB0005772		58307	128439	0.48635	0.82663
Bonemar	11.94	1005.5194	M+Cl	5	970.5547598	PIP(20:1(11Z)/18:1(	C47H88O16P2		HMDB0010000		61381	131750506	0.96355	0.98685
Bonemar	12	911.5604	M-H	6	912.5727793	PI(22:3(10Z,13Z,16	C49H85O13P		HMDB0009909		61294		0.78481	0.93388
Bonemar	8.17	536.3689	M-H	6	537.3794397	PC(O-16:0/3:0)[U]	C27H56NO7P				40096		0.87085	0.95892
Bonemar	11.96	905.514	M-H	5	906.525834	PI(18:4(6Z,9Z,12Z,1	C49H79O13P			LMGP0601	80444		0.64833	0.88665
Bonemar	9.79	836.5626	M+Cl	6	801.588373	PS(P-16:0/22:1(11Z	C44H84NO9P			LMGP0303	78765		0.039444	0.45321
Bonemar	9.92	713.512	M-H	1	714.5199564	PA(15:0/22:2(13Z,1	C40H75O8P		HMDB011	LMGP1001	81322	52928749	0.27181	0.67016
Bonemar	12.08	804.4961	M+Cl	1	769.525773	PS(O-16:0/20:4(5Z,	C42H76NO9P			LMGP0302	78737		0.79197	0.93492
Bonemar	12	882.5141	M+Cl	10	847.536338	PS(22:6(4Z,7Z,10Z,1	C47H78NO10P			LMGP0301	78498		0.26194	0.65978
Bonemar	11.95	896.5278	M+Cl	7	861.5519848	PS(22:4(7Z,10Z,13Z	C48H80NO10P		HMDB011	LMGP0301	78473	52925919	0.72401	0.91723
Bonemar	11.83	816.5196	M+Cl	4	781.546902	PI-Cer(d20:0/14:0)	C40H80NO11P			LMSP03030152			0.26508	0.66331
Bonemar	10.19	797.6499	M-H	5	798.661476	SM(d17:1/24:1)	C46H91N2O6P			LMSP0301	83774		0.85508	0.95417
Bonemar	11.96	897.5492	M-H	1	898.557134	PI(19:0/20:5(5Z,8Z	C48H83O13P			LMGP0601	80463		0.87217	0.95892
Bonemar	0.33	1097.7634	M-H	3	1098.767127	GlcNalpha1-6Ins-1-F	C56H111N2O16P			LMSP0604	41634		0.21272	0.60989
Bonemar	12	858.5205	M-H	10	859.5363347	PS(22:4(7Z,10Z,13Z	C48H78NO10P		HMDB011	LMGP0301	78474	52925920	0.4284	0.79747
Bonemar	8.51	446.1675	M-H	1	447.1754	Trp Glu Asn	C20H25N5O7				16147		0.019518	0.45321
Bonemar	10.2	673.488	M-H	10	674.4886558	PA(18:0/16:1(9Z))	C37H71O8P		HMDB011	LMGP1001	82053	52929479	0.3904	0.77759
Bonemar	6.85	482.3608	M+H	1	481.3532062	Lyso-PAF C-16	C24H52NO6P				24070		0.34679	0.99994
Bonemar	11.87	727.562	M+H	5	726.558711	Coenzyme Q8	C49H74O4	C17569		LMPR0201	4247	5283546	0.050979	0.99994
Bonemar	10.01	772.6164	M+H	7	771.6141906	PC(P-16:0/20:1(11Z	C44H86NO7P		HMDB001	LMGP0103	62046	52923904	0.96981	0.99994
Bonemar	9.94	770.602	M+H	5	769.5985406	PC(P-16:0/20:2(11Z	C44H84NO7P		HMDB001	LMGP0103	62047	52923906	0.69907	0.99994
Bonemar	10.09	728.5572	M+H	2	727.5515904	PE(18:1(11Z)/P-18:	C41H78NO7P		HMDB0009051		60468	53479649	0.96845	0.99994
Bonemar	11.87	744.4929	M+Na	1	721.5046402	PE(P-18:1(11Z)/18:	C41H72NO7P		HMDB0011412		62211	53480872	0.072385	0.99994
Bonemar	10	798.6272	M+Na	10	775.6454908	PC(O-16:0/20:0)	C44H90NO7P		HMDB001	LMGP0102	40078	24779297	0.78367	0.99994
Bonemar	9.96	744.5883	M+H	3	743.5828905	PC(P-18:0/16:1(9Z))	C42H82NO7P		HMDB001	LMGP0103	62066	52923934	0.5205	0.99994
Bonemar	11.85	750.5423	M+H	1	749.5359403	PE(P-16:0/22:5(4Z,7	C43H76NO7P		HMDB0011359		62170	53480849	0.69619	0.99994
Bonemar	11.91	524.2751	M+Na	1	501.2855393	PE(20:4(5Z,8Z,11Z,1	C25H44NO7P		HMDB001	LMGP0205	46715	42607465	0.093873	0.99994
Bonemar	9.95	745.5914	M+H	8	744.578141	PE-Cer(d15:2(4E,6E	C41H81N2O7P			LMSP0302	103118		0.48542	0.99994
Bonemar	11.86	833.651	M+Na	0	810.661476	SM(d18:2/24:1)	C47H91N2O6P			LMSP0301	83780		0.13525	0.99994
Bonemar	9.94	771.6063	M+H	9	770.606055	DG(11M5/13D5/0:0	C48H82O7		HMDB0116400			131823055	0.683	0.99994
Bonemar	11.85	767.5594	M+H	1	766.551259	PA(22:4(7Z,10Z,13Z	C44H79O8P			LMGP1001	81969		0.15097	0.99994
Bonemar	8.01	640.3229	M+H	5	639.31895	Leukotriene C4 meth	C31H49N3O9S				45048		0.26407	0.99994
Bonemar	9.79	730.5418	M+H	5	729.5308549	PC(18:1(11Z)/14:1(	C40H76NO8P		HMDB0008065		59512	53478713	0.93606	0.99994
Bonemar	9.88	784.5823	M+H	4	783.5778051	PC(20:3(5Z,8Z,11Z)	C44H82NO8P		HMDB000	LMGP0101	59810	53478949	0.65951	0.99994
Bonemar	11.85	722.509	M+Na	1	699.5202902	PE(18:2(9Z,12Z)/P-1	C39H74NO7P		HMDB0009114		60521	53479669	0.20375	0.99994
Bonemar	10.02	337.2743	M+H	2	336.266445	Isolinderanolide	C21H36O3		HMDB0038105		92862	24991154	0.013082	0.7711
Bonemar	11.86	738.5087	M+H	3	737.4995548	PE(22:5(7Z,10Z,13Z	C41H72NO8P		HMDB0009646		61035	53479942	0.11583	0.99994
Bonemar	10.02	746.6031	M+H	4	745.5985084	PC(O-18:1(9Z)/16:0	C42H84NO7P				40014		0.43174	0.99994
Bonemar	10.66	955.6259	M+Na	1	932.635384	PI(19:0/22:2(13Z,16	C50H93O13P			LMGP0601	80467		0.16288	0.99994
Bonemar	11.85	742.5688	M+H	8	741.5672404	PC(18:2(9Z,12Z)/P-	C42H80NO7P		HMDB0008159		59605	53478799	0.075882	0.99994
Bonemar	10.02	700.5286	M+Na	5	677.53594	PE(O-16:0/16:0)[U]	C37H76NO7P				40748		0.94157	0.99994
Bonemar	10.1	801.6505	M+H	3	800.6407397	SM(d18:0/22:1(13Z	C45H89N2O7P		HMDB0013466			53481785	0.71847	0.99994
Bonemar	0.43	137.0466	M+H	6	136.0385108	Hypoxanthine	C5H4N4O	C00262	HMDB0000157		83	790	0.18675	0.99994
Bonemar	9.14	467.3348	M+H	4	466.329441	Varanic acid	C27H46O6			LMST0403	84812		0.57827	0.99994
Bonemar	8.03	621.3024	M+H	2	620.296168	PI(20:4(5Z,8Z,11Z,1	C29H49O12P			LMGP0605	46748		0.18606	0.99994
Bonemar	9.97	796.6152	M+H	8	795.6141906	PC(P-18:0/20:3(8Z,	C46H86NO7P		HMDB001	LMGP0103	62077	52923962	0.9162	0.99994
Bonemar	9.9	743.5645	M+H	8	742.551259	PA(22:2(13Z,16Z)/1	C42H79O8P			LMGP1001	81929		0.62686	0.99994
Bonemar	11.86	723.5074	M+Na	4	700.515541	PE-Cer(d14:2(4E,6E	C38H73N2O7P			LMSP0302	103110		0.11878	0.99994
Bonemar	11.91	745.4864	M+H	8	744.4730062	PA(18:3(9Z,12Z,15Z	C43H69O8P		HMDB0115022			131821956	0.15054	0.99994
Bonemar	9.92	768.5831	M+H	9	767.5828905	PC(18:2(9Z,12Z)/P-	C44H82NO7P		HMDB0008161		59607	53478803	0.7417	0.99994
Bonemar	0.43	613.1599	M+H	1	612.1519619	Oxidized glutathione	C20H32N6O12S2		HMDB0003337		45	5255796	0.41854	0.99994
Bonemar	6.55	506.3192	M+H	10	505.3168394	PE(20:2(11Z,14Z)/0	C25H48NO7P		HMDB001	LMGP0205	77687	52925140	0.90664	0.99994

1. Metabolites affected by drug only

Day 4

ami_50/Vehicle

Type	RT	erimental.r	Adduct	PPM.Error	olecular.Weig	Name	Formula	Kegg	HMDB	LipidMaps	Metlin
Bonemar	9.81	860.5935	M+Na	6	837.609503	PI-Cer(d18:0/20:0)	C44H88NO11P			LMSP0303	103137
Bonemar	9.84	649.5171	M+H	1	648.509394	PA(O-20:0/13:0)	C36H73O7P			LMGP100	82189
Bonemar	9.9	906.6944	M+H	0	905.6873561	PE-NMe2(22:5(4Z,7Z	C53H96NO8P		HMDB0114547		
Bonemar	6.68	426.3579	M+H	0	425.350509	Elaidic carnitine	C25H47NO4		HMDB000	LMFA0707	58415
Bonemar	6.23	479.2907	M+Na	6	456.298808	N-docosahexaenoyl	C27H40N2O4			LMFA0802	75511
Bonemar	0.42	308.0908	M+H	1	307.083806	Glutathione	C10H17N3	C00051	HMDB0000125		44
Bonemar	11.85	749.5257	M+H	9	748.5254352	PG(18:0/16:1(9Z))	C40H77O10P		HMDB001	LMGP0401	61876
Bonemar	11.86	748.5236	M+H	5	747.5202902	PE(20:5(5Z,8Z,11Z	C43H74NO7P		HMDB0009480		60875
Bonemar	0.42	348.0717	M+Na	8	325.0797665	Dihydroxy-1H-indole	C14H15NO8		HMDB0059997		
Bonemar	0.39	204.1233	M+H	1	203.115758	N-lactoyl-Leucine	C9H17NO4		HMDB0062176		
Bonemar	10.13	843.6842	M+H	1	842.676457	PA(24:0/22:1(13Z))	C49H95O8P		HMDB0115447		
Bonemar	11.89	790.5447	M+H	8	789.5308549	PE(20:4(8Z,11Z,14Z	C45H76NO8P		HMDB0009431		60827
Bonemar	10.02	846.6605	M+H	3	845.650973	PS(P-20:0/21:0)	C47H92NO9P			LMGP0303	78821
Bonemar	11.87	747.5085	M+H	6	746.496901	MGDG(18:3(9Z,12Z	C43H70O10			LMGL0501	46655
Bonemar	10.66	969.6415	M+H	1	968.635384	PI(22:1(11Z)/22:4(	C53H93O13P			LMGP0601	80745
Bonemar	0.43	560.0773	M+H	3	559.07167	Phosphoribosyl-AM	C15H23N5	C02741			3596
Bonemar	5.58	453.1719	M+Na	6	430.18524	Pro Trp Glu	C21H26N4O6				16156
Bonemar	10.1	816.642	M+H	7	815.6404054	PC(24:1(15Z)/14:0)	C46H90NO8P		HMDB0008788		60234
Bonemar	9.91	794.6014	M+H	6	793.5985406	PC(P-16:0/22:4(7Z,	C46H84NO7P		HMDB0011226		62056
Bonemar	11.91	522.2588	M+Na	1	499.2698892	PE(20:5(5Z,8Z,11Z	C25H42NO7P		HMDB001	LMGP0205	77693
Bonemar	6.24	478.293	M+H	9	477.2812	Arg Phe Arg	C21H35N9O4				19179
Bonemar	11.86	746.5082	M+Na	2	723.5202902	PE(P-16:0/20:4(8Z	C41H74NO7P		HMDB0011353		62164
Bonemar	9.74	755.5532	M+H	7	754.5512565	PA(18:1(11Z)/22:2(	C43H79O8P		HMDB0114914		
Bonemar	11.84	776.5576	M+H	2	775.5515904	PE(22:5(7Z,10Z,13Z	C45H78NO7P		HMDB0009678		61067
Bonemar	10.09	729.5654	M+H	1	728.5591048	DG(9D3/13D5/0:0)	C45H76O7		HMDB0116426		
Bonemar	10.72	957.6384	M+H	4	956.635384	PI(21:0/22:4(7Z,10Z	C52H93O13P			LMGP0601	80692
Bonemar	6.01	372.3109	M+H	0	371.303559	N-stearoyl serine	C21H41NO4			LMFA0802	75484
Bonemar	9.79	756.5545	M+H	1	755.546505	PC(18:3(9Z,12Z,15Z	C42H78NO8P		HMDB000	LMGP0101	59645
Bonemar	0.38	160.1338	M+H	4	159.1259288	DL-2-amino-octanoi	C8H17NO2		HMDB000	LMFA0110	74884
Bonemar	9.96	692.5568	M+H	3	691.5515904	PC(O-14:0/16:0)[U]	C38H78NO7P				40042
Bonemar	11.85	797.5275	M+H	10	796.512551	MGDG(20:5(5Z,8Z,1	C47H72O10			LMGL0501	46652
Bonemar	11.84	774.542	M+H	2	773.5359403	PE(P-18:1(11Z)/22:	C45H76NO7P		HMDB0011427		62226
Bonemar	10.06	702.5484	M+H	7	701.5359403	PE(16:0/P-18:1(9Z))	C39H76NO7P		HMDB0008952		60382
Bonemar	10.08	603.534	M+Na	3	580.543061	DG(O-16:0/18:1(9Z	C37H72O4	C13862		LMGL0202	4696
Bonemar	11.89	769.4909	M+H	6	768.478884	PI(16:0/13:0)	C38H73O13P			LMGP0601	80182
Bonemar	9.96	718.5719	M+H	4	717.5672404	PC(P-18:0/14:0)	C40H80NO7P		HMDB001	LMGP0103	62062
Bonemar	10	726.5425	M+Na	2	703.5515904	PE(18:0/P-16:0)	C39H78NO7P		HMDB0009015		60433
Bonemar	11.89	849.5425	M+H	7	848.541484	PI(16:1(9Z)/19:1(9Z	C44H81O13P			LMGP0601	80205
Bonemar	11.13	890.6796	M+H	5	889.677188	PS(21:0/22:0)	C49H96NO10P			LMGP0301	78362
Bonemar	12.02	885.5447	M+H	5	884.5414792	PI(22:3(10Z,13Z,16Z	C47H81O13P		HMDB0009908		61293
Bonemar	10.08	826.6609	M+H	9	825.6611408	PC(22:2(13Z,16Z)/P	C48H92NO7P		HMDB0008620		60066
Bonemar	11.87	751.5332	M+H	8	750.5199564	PA(22:2(13Z,16Z)/1	C43H75O8P		HMDB011	LMGP1001	81935
Bonemar	11.84	819.5135	M+H	4	818.5097851	PG(18:3(6Z,9Z,12Z)	C46H75O10P		HMDB0010673		61944
Bonemar	10.12	605.5451	M+Na	5	582.5587109	DG(16:0e/18:0/0:0)	C37H74O4		HMDB0011146		62000
Bonemar	9.89	758.5701	M+H	1	757.5621551	PC(18:2(9Z,12Z)/16	C42H80NO8P		HMDB000	LMGP0101	59579
Bonemar	9.79	701.5516	M+H	5	700.540694	PA(O-20:0/17:2(9Z	C40H77O7P			LMGP100	82197
Bonemar	10.77	921.6404	M+H	2	920.635384	PI(18:1(9Z)/22:0)	C49H93O13P			LMGP0601	80950
Bonemar	12.01	886.5493	M+Na	9	863.5676348	PS(22:5(7Z,10Z,13Z	C48H82NO10P		HMDB0112851		
Bonemar	6.33	424.3423	M+H	0	423.3348589	Linoelaidyl carnitine	C25H45NO4		HMDB000	LMFA0707	58412
Bonemar	11.01	888.6648	M+H	5	887.661538	PS(21:0/22:1(11Z))	C49H94NO10P			LMGP0301	78363
Bonemar	11.9	762.5102	M+H	4	761.4995548	PE(20:4(8Z,11Z,14Z	C43H72NO8P		HMDB0009424		60820
Bonemar	10.77	920.6352	M+H	3	919.6302351	PS(22:5(7Z,10Z,13Z	C52H90NO10P		HMDB0112863		
Bonemar	11.12	876.6679	M+H	1	875.6615352	PS(18:0/24:0)	C48H94NO10P		HMDB0112384		
Bonemar	11.84	802.5726	M+Na	1	779.5828905	PE(P-18:0/22:4(7Z	C45H82NO7P		HMDB001	LMGP0203	62195
Bonemar	11.11	862.6517	M+H	2	861.645888	PS(21:0/20:0)	C47H92NO10P			LMGP0301	78356
Bonemar	6.31	448.3405	M+H	4	447.3348589	N-stearoyl tyrosine	C27H45NO4		HMDB006	LMFA0802	75482
Bonemar	10.77	958.6496	M+Na	1	935.6615347	PE(DiMe(13,5)/DiM	C53H94NO10P		HMDB0061491		
Bonemar	10.84	908.6362	M+H	1	907.6302346	PE(DiMe(11,5)/DiM	C51H90NO10P		HMDB0061482		
Bonemar	5.63	437.194	M+H	4	436.188591	Isochanelieric acid m	C26H28O6			LMPK1210	48440
Bonemar	6.16	398.3257	M+H	2	397.3192089	trans-Hexadec-2-en	C23H43NO4		HMDB000	LMFA0707	58388
Bonemar	11.88	726.5563	M+H	7	725.544185	Phalluside-1	C41H75NO9			LMSP0501	83804
Bonemar	9.79	858.6003	M+H	1	857.5934552	PC(20:3(8Z,11Z,14Z	C50H84NO8P		HMDB0008417		59863
Bonemar	12.2	575.5028	M+H	1	574.496111	Mayolene-19	C37H66O4			LMFA07010690	
Bonemar	8.8	511.3359	M+Na	7	488.3501746	Pitheduloside I	C30H48O5		HMDB0034036		89673
Bonemar	9.85	819.608	M+H	4	818.603689	PG(21:0/18:1(9Z))	C45H87O10P			LMGP0401	79510
Bonemar	9.72	647.5008	M+H	0	646.493744	PA(P-16:0/17:0)	C36H71O7P			LMGP100	82249
Bonemar	11.84	799.5431	M+H	10	798.528201	MGDG(20:5(5Z,8Z,1	C47H74O10			LMGL0501	46649
Bonemar	5.63	438.197	M+H	3	437.19105	Tyr Gln Gln	C19H27N5O7				20796
Bonemar	11.85	773.5255	M+H	7	772.512551	MGDG(18:3(9Z,12Z	C45H72O10			LMGL0501	46650
Bonemar	9.9	857.6348	M+H	10	856.619339	PG(22:0/20:3(8Z,11	C48H89O10P			LMGP0401	79543
Bonemar	10.78	959.6535	M+Na	3	936.666684	PI(22:0/19:0)	C50H97O13P			LMGP0601	80705
Bonemar	12.02	855.4936	M+H	10	854.494534	PI(18:3(9Z,12Z,15Z)	C45H75O13P			LMGP0601	80976
Bonemar	12.03	785.5039	M+Na	7	762.519959	PA(22:6(4Z,7Z,10Z	C44H75O8P			LMGP1001	81998
Bonemar	0.41	386.0448	M+Na	7	363.057999	Cyclic pyranopterin	C10H14N5	C18239	HMDB0059639		72085
Bonemar	11.98	923.4858	M+H	3	922.4761165	PGP(22:6(4Z,7Z,10Z	C48H76O13P2		HMDB0116746		
Bonemar	11.86	793.5181	M+H	6	792.505754	SQDG(16:0/16:1(9Z	C41H76O12S			LMGL0501	46642
Bonemar	12.01	883.5258	M+H	8	882.525834	PI(18:4(6Z,9Z,12Z,1	C47H79O13P			LMGP0601	80436
Bonemar	10.84	922.65	M+H	3	921.6458847	PE(DiMe(13,5)/Mor	C52H92NO10P		HMDB0061496		
Bonemar	11.96	907.5282	M+H	5	906.525834	PI(18:4(6Z,9Z,12Z,1	C49H79O13P			LMGP0601	80444
Bonemar	11.99	875.49	M+H	8	874.4761155	PGP(16:0/22:6(4Z,7	C44H76O13P2		HMDB0013486		
Bonemar	10.02	824.6465	M+H	8	823.6454908	PC(P-18:1(9Z)/22:2	C48H90NO7P		HMDB001	LMGP0103	62140
Bonemar	11.82	771.5113	M+H	9	770.496901	MGDG(20:5(5Z,8Z,1	C45H70O10			LMGL0501	46653
Bonemar	6.68	427.3606	M+H	8	426.349781	zymosterol intermed	C29H46O2			LMST0101	57615
Bonemar	10.85	923.653	M+H	6	922.651034	PI(19:0/21:0)	C49H95O13P			LMGP0601	80464
Bonemar	10	748.5897	M+H	6	747.5778051	PE(16:0/20:0)	C41H82NO8P		HMDB000	LMGP0201	40667
Bonemar	10.05	775.6167	M+H	6	774.6138568	PA(20:0/21:0)	C44H87O8P		HMDB011	LMGP1001	81688
Bonemar	8.86	439.2786	M+H	8	438.27464	LPA(0:0/18:0)	C21H43O7P			LMGP10050043	
Bonemar	6.57	401.3425	M+Na	9	378.34978	Farnesic acid	C25H46O2			LMFA01020400	
Bonemar	8.77	267.2677	M+H	2	266.26097	octadecan-3Z,13Z-d	C18H34O				75270
Bonemar	11.17	851.6547	M+Na	8	828.6720399	SM(d18:0/24:1(15Z	C47H93N2O7P		HMDB0013469		
Bonemar	5.62	439.2005	M+H	7	438.19032	Phe Ser Trp	C23H26N4O5				16810
Bonemar	11.9	848.5362	M+H	9	847.536338	PS(22:6(4Z,7Z,10Z,1	C47H78NO10P			LMGP0301	78498
Bonemar	9.78	961.7196	M+H	9	960.720692	TG(17:2(9Z,12Z)/22	C64H96O6			LMGL0301	38826
Bonemar	8.1	239.2363	M+H	3	238.22967	hexadeca-7,11-dien	C16H30O				75249
Bonemar	9.48	953.6834	M+Na	7	930.700747	DAT(16:0/22:0(2Me	C52H98O13			LMSL0300	82433
Bonemar	10.09	841.6622	M+H	7	840.660807	PA(22:1(13Z)/24:1(	C49H93O8P		HMDB0115295		
Bonemar	12.21	577.5179	M+H	2	576.5117607	Cohibin D	C37H68O4		HMDB0035398		90692
Bonemar	12.02	854.4902	M+H	8	853.4893845	PS(22:6(4Z,7Z,10Z,1	C48H72NO10P		HMDB011	LMGP0301	78504
Bonemar	7.69	650.4339	M+H	8	649.431872	PC(16:0/9:0(CHO))	C33H64NO9P			LMGP2001	82381
Bonemar	8.67	310.3108	M+H	1	309.30317	Oleoyl Ethyl Amide	C20H39NO				44905
Bonemar	10.77	907.6238	M+H	4	906.619734	PI(22:0/17:1(9Z))	C48H91O13P			LMGP0601	80699
Bonemar	9.27	598.3851	M+Na	1	575.3950897	PC(22:2(13Z,16Z)/0	C30H58NO7P		HMDB001	LMGP0105	76577
Bonemar	10.85	909.641	M+H	2	908.635384	PI(22:0/17:0)	C48H93O13P			LMGP0601	80698
Bonemar	12.07	811.5261	M+H	9	810.5258291	PI(16:0/16:0)	C41H79O13P		HMDB000	LMGP0601	40890
Bonemar	9.93	662.5123	M+H	1	661.5046402	PE(15:0/P-16:0)	C36H72NO7P		HMDB0008916		60359
Bonemar	8.75	343.2853	M+H	3	342.277011	Eicosanedioic acid	C20H38O4			LMFA0117	35987
Bonemar	12.19	788.5398	M+H	5	787.53633	PS(18:1(9Z)/18:1(9Z	C42H78NO10P				40798
Bonemar	11.81	709.5327	M+H	7	708.530524	Mannosyl-1beta-ph	C38H77O9P			LMPK0100	46757
Bonemar	9.97	786.6	M+H	1	785.5934552	PC(18:2(9Z,12Z)/18	C44H84NO8P		HMDB000	LMGP0101	59581
Bonemar	6.62	434.2543	M+H	0	433.24712	Lys Arg Met	C17H35N7O4S				16029
Bonemar	7.61	466.3273	M+H	4	465.321927	PC(P-15:0/0:0)	C23H48NO6P			LMGP0107	40399
Bonemar	8.88	448.3755	M+Na	1	425.3868945	Tetracosanoylglycine	C26H51NO3		HMDB0013310		
Bonemar	10.09	957.8025	M+H	10	956.8044204	Sorbitan trioleate	C60H108O8		HMDB0029889		86568
Bonemar	8.76	341.3044	M+H	2	340.29775	Glycidyl stearate	C21H40O3	C19427			73128
Bonemar	10.11	896.7095	M+H	1	895.7030056	PC(22:2(13Z,16Z)/2	C52H98NO8P		HMDB0008611		60057
Bonemar	8.96	431.2304	M+Na	10	408.23727	Tyr Val Lys	C20H32N4O5				15678
Bonemar	8.91	681.442	M+Na	7	658.4573561	PA(18:2(9Z,12Z)/15	C36H67O8P		HMDB011	LMGP1001	81508
Bonemar	9.24	642.4139	M+Na	5	619.421307	PE(12:0/15:1(9Z))	C32H62NO8P			LMGP0201	76596
Bonemar	11.87	781.5247	M+H	3	780.515269	PI(O-16:0/15:1(9Z))	C40H77O12P			LMGP0602	80991
Bonemar	12.04	832.5153	M+Na	7	809.5206842	PE(MonoMe(9,5)/D	C44H76NO10P		HMDB0061547		
Bonemar	10.05	752.5593	M+Na	4	729.5672404	PE(18:1(11Z)/P-18:	C41H80NO7P		HMDB0009049		60466
Bonemar	5.13	410.1696	M+H	10	409.1584107	dermatan L-iduronat	C16H27NO11		HMDB0060470		
Bonemar	9.85	818.6029	M+H	4	817.5985406	PC(22:5(4Z,7Z,10Z,	C48H84NO7P		HMDB0008687		60133
Bonemar	12.12	887.5591	M+H	6	886.5571293	PI(16:0/22:4(7Z,10Z	C47H83O13P		HMDB000	LMGP0601	61179
Bonemar	0.42	630.0712	M+Na	1	607.0815695	Uridine diphosphate	C17H27N3O17P2		HMDB0000304		3675
Bonemar	9.92	707.5538	M+Na	1	684.565254	Lysine-containing sic	C39H76N2O7			LMFA0000	45745
Bonemar	10.66	983.6541	M+Na	2	960.666684	PI(21:0/22:2(13Z,16	C52H97O13P			LMGP0601	80691
Bonemar	11.92	843.5229	M+H	7	842.5097856	PG(22:6(4Z,7Z,10Z,	C48H75O10P		HMDB0116604		
Bonemar	7.09	428.3737	M+H	1	427.3661591	Stearoylcarnitine	C25H49NO4		HMDB000	LMFA0707	5811
Bonemar	11.95	905.5097	M+H	9	904.510184	PI(22:6(4Z,7Z,10Z,1	C49H77O13P			LMGP0601	80821
Bonemar	9.14	687.4559	M+Na	2	664.467924	PG(P-16:0/13:0)	C35H69O9P			LMGP0403	79906
Bonemar	10.05	577.4836	M+H	2	576.4753752	DG(15:0/18:3(6Z,9Z	C36H64O5		HMDB0007075		58710
Bonemar	11.88	725.5544	M+Na	3	702.5675748	SM(d18:1/16:0)	C39H79N2O6P		HMDB006	LMSP0301	41586
Bonemar	10.02	1001.8429	M+Na	8	978.8615414	TG(24:1(15Z)/15:0/	C64H114O6		HMDB0051940		
Bonemar	9.68	945.6701	M+Na	7	922.687419	PI(O-20:0/21:0)	C50H99O12P			LMGP0602	81050
Bonemar	9.74	981.7135	M+Na	8	958.732047	DAT(16:0/24:0(2Me	C54H102O13			LMSL0300	82435
Bonemar	12	891.4986	M+Na	1	868.510184	PI(17:1(9Z)/20:5(5Z	C46H77O13P			LMGP0601	80261
Bonemar	11.94	1182.46	M+Na	9	1159.48064	CoA(26:0(3Ke))	C47H84N7O18P3S				97426
Bonemar	8.05	433.1926	M+H	5	432.18313	Pro Met Trp	C21H28N4O4S				16393
Bonemar	10.05	847.6742	M+H	5	846.671374	PG(P-20:0/22:0)	C48H95O9P			LMGP0403	79986
Bonemar	11.95	1045.4765	M+H	5	1044.47414	PIP2(18:1(9Z)/20:4	C47H83O19P3		HMDB0010096		61474
Bonemar	10.9	924.6624	M+H	7	923.6615352	PS(24:0/22:4(7Z,10	C52H94NO10P		HMDB0112904		
Bonemar	9.7	340.3606	M+H	9	339.3501151	Docosanamide	C22H45NO		HMDB000	LMFA0801	5565
Bonemar	12.02	884.5338	M+Na	9	861.5519848	PS(22:4(7Z,10Z,13Z	C48H80NO10P		HMDB011	LMGP0301	78473
Bonemar	0.33	969.7956	M+H	5	968.783292	TG(17:2(9Z,12Z)/22	C64H104O6			LMGL0301	38695
Bonemar	8.27	408.3098	M+Na	4	385.3192089	Pentadecanoylcarnit	C22H43NO4		HMDB0062517		
Bonemar	8.51	499.3416	M+H	4	498.332158	PG(O-18:0/0:0)	C24H51O8P			LMGP0406	80026
Bonemar	9.63	672.5016	M+H	8	671.4889901	PE(14:1(9Z)/P-18:1(	C37H70NO7P		HMDB0008886		60330
Bonemar	0.41	310.0864	M+H	8	309.0817	Met Cys Gly	C10H19N3O4S2				16638
Bonemar	10.06	956.8065	M+H	2	955.796906	PC(24:0/24:1(15Z))	C56H110NO8P		HMDB0008783		60229
Bonemar	5.13	409.1657	M+H	7	408.16135	Met Arg Cys	C14H28N6O4S2				18334
Bonemar	8.71	386.2661	M+H	7	385.26169	Docosahexaenoyl Gl	C24H35NO3				62993
Bonemar	11.94	1130.4521	M+H	10	1129.433689	(15Z)-3-oxotetracos	C45H78N7O18P3S			LMFA07050097	
Bonemar	12.02	893.5295	M+Na	1	870.5410857	PG(22:5(4Z,7Z,10Z,	C50H79O10P		HMDB0116618		
Bonemar	7.64	510.3914	M+H	1	509.384525	PC(O-10:0/O-8:0)[U	C26H56NO6P				40183
Bonemar	9.13	254.2472	M+H	3	253.240564	Palmitoleamide	C16H31NO			LMFA0801	97431
Bonemar	11.93	1085.4709	M+H	6	1084.470293	LPIM3(18:1(9Z)/0:0)	C45H81O27P			LMGP15040020	
Bonemar	11.95	1127.4558	M+H	7	1126.45612	PIP3(18:1(11Z)/20:	C47H86O22P4		HMDB0010157		61535
Bonemar	8.11	235.1329	M+H	0	234.1255944	Pterosin N	C14H18O3		HMDB0038218		92947
Bonemar	9.56	645.4881	M+H	4	644.478094	PA(P-16:0/17:1(9Z))	C36H69O7P			LMGP100	82250
Bonemar	7.16	376.2544	M+H	3	375.24817	Lys Lys Thr	C16H33N5O5				18691
Bonemar	11.17	837.641	M+H	5	836.6295068	PA(22:4(7Z,10Z,13Z	C49H89O8P		HMDB0115347		
Bonemar	12.03	857.5121	M+H	6	856.510184	PI(22:4(7Z,10Z,13Z	C45H77O13P			LMGP0601	80781
Bonemar	11.99	911.5547	M+Na	8	888.5727793	PI(18:0/20:3(5Z,8Z	C47H85O13P		HMDB0009813		61198
Bonemar	7.07	594.3722	M+H	7	593.3692689	POV-PC	C29H56NO9P				45370
Bonemar	9.71	621.4829	M+H	4	620.478094	PA(O-16:0/15:0)	C34H69O7P			LMGP100	82152
Bonemar	11.92	889.5258	M+Na	7	866.530919	PI(P-16:0/22:6(4Z,7	C47H79O12P			LMGP0603	81170
Bonemar	0.34	1022.7473	M+H	3	1021.742946	FMC-5(d18:1/24:0)	C58H103NO13			LMSP0501	83800
Bonemar	12.03	856.5075	M+H	6	855.5050341	PS(22:6(4Z,7Z,10Z,1	C48H74NO10P		HMDB001	LMGP0301	78503
Bonemar	0.33	873.8318	M+H	6	872.8196766	TG(18:1(11Z)/o-18:	C57H108O5		HMDB0049681		
Bonemar	11.87	780.5332	M+H	7	779.531252	PI-Cer(d20:1/14:0)	C40H78NO11P			LMSP03030151	
Bonemar	11.92	949.4957	M+H	4	948.4917666	PGP(22:5(4Z,7Z,10Z	C50H78O13P2		HMDB0116495		
Bonemar	11.93	1068.5035	M+H	1	1067.497266	Cyclolinopeptide I	C55H73N9O9S2		HMDB0036552		91633
Bonemar	10.02	799.6374	M+H	6	798.6250897	SM(d18:0/22:2(13Z	C45H87N2O7P		HMDB0013467		
Bonemar	0.33	1144.7817	M+H	3	1143.777358	MIPC(t20:0/26:0(2	C58H114NO18P			LMSP0303	103224
Bonemar	8.9	573.457	M+H	10	572.444076	DG(13:0/20:5(5Z,8Z	C36H60O5			LMGL0201	98387
Bonemar	0.33	1043.8029	M+Na	1	1020.814592	TG(21:0/22:6(4Z,7Z	C68H108O6			LMGL0301	39083
Bonemar	10.02	361.2745	M+H	2	360.26645	O-Arachidonoyl Glyc	C23H36O3				44872
Bonemar	9.99	591.4367	M+H	3	590.431144	PA(P-16:0/13:0)	C32H63O7P			LMGP100	82242
Bonemar	9.87	950.7398	M+H	2	949.734703	PI-Cer(d20:0/26:0)	C52H104NO11P			LMSP0303	103158
Bonemar	11.95	917.5226	M+H	8	916.5078096	PIP[3'](16:0/18:1(9	C43H82O16P2		HMDB000	LMGP0701	61309
Bonemar	8.1	313.2736	M+H	0	312.26645	Ricinoleic Acid meth	C19H36O3				45190
Bonemar	6.33	346.3132	M+H	8	345.303164	N-propyl arachidonoy	C23H39NO			LMFA0802	36681
Bonemar	11.86	795.5115	M+H	7	794.5097851	PG(18:3(6Z,9Z,12Z)	C44H75O10P		HMDB001	LMGP0401	61940
Bonemar	6.32	290.1624	M+H	9	289.1525375	O-adipoylcarnitine	C13H23NO6		HMDB006	LMFA07070087	
Bonemar	11.17	836.637	M+Na	1	813.648254	DGTA(18:1/22:4(10	C50H87NO7			LMGL0000	46619

Type	RT	erimental.r	Adduct	PPM.Error	olecular.Weig	Name	Formula	Kegg	HMDB	LipidMaps	Metlin	PubChem
Bonemar	11.99	879.4958	M+H	7	878.494534	PI(18:4(6Z,9Z,12Z,1	C47H75O13P			LMGP0601	80438	
Bonemar	5.66	416.267	M+Na	1	393.27801	Undecylprodigiosin	C25H35N3	C12023			69260	
Bonemar	0.41	179.0495	M+H	6	178.04121	Cysteinyl-Glycine	C5H10N2O3S				85689	
Bonemar	10.9	911.6555	M+H	3	910.6452	trans,octacis-Deca	C55H91O8	C20370				
Bonemar	9.85	664.4803	M+Na	7	641.48667	GlcCer(d14:2(4E,6E)	C36H67NO8			LMSP0501	103292	
Bonemar	9.85	765.5478	M+H	6	764.535609	PA(19:1(9Z)/22:4(7	C44H77O8P			LMGP1001	81673	
Bonemar	3.5	233.0774	M+Na	0	210.08818	Phosphoagmatine;Ag[illegible]	[illegible]	[illegible]			65821	439793
Bonemar	8.77	462.2558	M+Na	2	439.26555	Arg Lys His	C18H33N9O4				16758	
Bonemar	2.86	271.1197	M+H	8	270.11034	Idebenone Metaboli	C13H18O6				773	
Bonemar	11.96	1059.4693	M+H	7	1058.454643	LPIM3(16:0/0:0)	C43H79O27P			LMGP15040017		
Bonemar	6.65	713.4445	M+H	8	712.431539	PG(14:1(9Z)/18:4(6	C38H65O10P			LMGP0401	78949	
Bonemar	8.18	552.4033	M+H	2	551.3950688	PC(2:0/O-18:0)[U]	C28H58NO7P				40164	
[illegible]	[illegible]	[illegible]	[illegible]	[illegible]	[illegible]	[illegible]	[illegible]	[illegible]	[illegible]	[illegible]	[illegible]	[illegible]

Name	1. Metabolites affected by drug only Day 4 ami_50/Vehicle p.value	FDR	old.Chang	log2.FC	1. Metabolites affected by drug only Day 4 ami_200/Vehicle p.value	FDR	old.Chang	log2.FC	2. Dose response Day 4 Rad + ami_200/Rad + ami_50 p.value	FDR	old.Chang	log2.FC	Metabolites dysregulated by radiation o Day 4 Rad/Vehicle p.value	FDR	old.Chang	log2.FC	4. Metabolites that return to normal leve Day 4 Rad + ami_50/Rad p.value	FDR	old.Chang	log2.FC	4. Metabolites that return to normal level Day 4 Rad + ami_200/Rad p.value	FDR	old.Chang	log2.FC	5. Does the dose make a difference? Day 4 ami_200/ami_50 p.value	FDR	old.Chang	log2.FC	Does the dose make a differen Day 4 ad + ami_200/Rad + ami_50/Ra p.value	FDR	f.value
PI(18:4(6Z,9Z,12Z,1	0.42455	0.99994	1.0619	0.0867	0.76774	0.99946	1.0152	0.0218	0.0066394	0.034966	1.599	0.6771	1.185E-05	3.319E-05	0.50749	-0.9786	0.76756	0.9998	1.0739	0.1028	0.0006079	0.0076856	1.7171	0.7799	0.51108	0.99932	0.95604	-0.0649	0.0017063	0.011473	8.7665
Undecylprodigiosin	0.87809	0.99994	1.0293	0.0416	0.21695	0.99946	1.1253	0.1703	0.0066497	0.034967	0.7146	-0.4848	0.86039	0.87775	1.0102	0.0146	0.82521	0.9998	0.99154	-0.0123	0.0017674	0.013043	0.70855	-0.4971	0.37856	0.99932	1.0932	0.1286	0.0014335	0.010694	9.0888
Cysteinyl-Glycine	0.16043	0.99994	1.3596	0.4432	0.30587	0.99946	1.1908	0.2519	0.0066635	0.034986	1.7143	0.7776	1.11E-05	3.19E-05	0.21514	-2.2167	0.69195	0.9998	0.93726	-0.0935	0.01839	0.035833	1.6068	0.6842	0.55256	0.99932	0.87583	-0.1913	0.0062611	0.021361	6.5229
trans,octacis-Deca	0.67412	0.99994	1.0399	0.0565	0.0040635	0.50404	1.5714	0.652	0.0068367	0.035465	1.4633	0.5493	0.17317	0.19937	1.2844	0.3611	0.24884	0.9998	0.81014	-0.3038	0.17271	0.21598	1.1855	0.2455	0.00321	0.28305	1.5111	0.5956	0.038254	0.064525	3.8276
GlcCer(d14:2(4E,6E)	0.93497	0.99994	1.0212	0.0302	0.54677	0.99946	0.89784	-0.1555	0.0069643	0.03564	0.45997	-1.1204	4.223E-07	4.283E-06	4.139	2.0493	0.87473	0.9998	1.0349	0.0494	0.0015382	0.012387	0.476	-1.071	0.51335	0.99932	0.87922	-0.1857	0.0016398	0.011287	8.8396
PA(19:1(9Z)/22:4(7	0.54932	0.99994	0.92681	-0.1097	0.27747	0.99946	0.86406	-0.2108	0.0070818	0.035974	0.69918	-0.5163	7.808E-06	2.47E-05	2.0258	1.0185	0.82786	0.9998	0.98255	-0.0254	0.005208	0.019297	0.68698	-0.5417	0.66242	0.99932	0.93229	-0.1011	0.0018646	0.012031	8.6044
Phosphoagmatine;Ag[illegible]	0.94318	0.99994	1.0042	0.006	0.97549	0.99946	1.0099	0.0142	0.0071376	0.03615	0.71792	-0.4781	6.393E-06	2.153E-05	2.0223	1.016	0.88355	0.9998	0.99221	-0.0113	0.0023529	0.014339	0.71232	-0.4894	0.97066	0.99932	1.0057	0.0082	0.0035569	0.016074	7.4647
Arg Lys His	0.60302	0.99994	0.96085	-0.0576	0.42323	0.99946	0.93288	-0.1002	0.0071708	0.036159	0.73826	-0.4378	6.53E-07	5.115E-06	1.9815	0.9866	0.74858	0.9998	0.97485	-0.0368	0.0023037	0.014339	0.71969	-0.4746	0.8212	0.99932	0.97089	-0.0426	0.0025298	0.013898	8.0573
Idebenone Metaboli	0.9996	0.99994	1.0131	0.0188	0.99081	0.99946	1.0271	0.0386	0.0072558	0.036236	0.71937	-0.4752	6.115E-06	2.101E-05	2.0532	1.0379	0.86739	0.9998	0.99184	-0.0118	0.0018601	0.013376	0.7135	-0.487	0.99129	0.99932	1.0138	0.0198	0.0031922	0.015419	7.6508
LPIM3(16:0/0:0)	0.62136	0.99994	1.0408	0.0576	0.50313	0.99946	0.92352	-0.1148	0.0074968	0.036831	1.4885	0.5739	0.077125	0.092998	0.80523	-0.3125	0.79108	0.9998	0.97868	-0.0311	0.0037005	0.016507	1.4568	0.5428	0.15179	0.99932	0.88736	-0.1724	0.0058886	0.02077	6.6226
PG(14:1(9Z)/18:4(6	0.43002	0.99994	1.1387	0.1874	0.32765	0.99946	1.1565	0.2098	0.0076946	0.037061	0.60771	-0.7186	2.677E-06	1.188E-05	2.3365	1.2243	0.3301	0.9998	1.2023	0.2658	0.017734	0.035211	0.73066	-0.4527	0.89801	0.99932	1.0157	0.0224	0.0098446	0.02754	5.8047
PC(2:0/O-18:0)[U]	0.52858	0.99994	0.91545	-0.1274	0.50664	0.99946	1.0723	0.1008	0.0079794	0.037386	1.5357	0.6189	1.04E-02	1.40E-02	0.68516	-0.5455	0.12058	0.9998	0.81116	-0.3019	0.12353	0.16043	1.2457	0.317	0.14657	0.99932	1.1714	0.2282	0.014779	0.03541	5.1858
[illegible]	[illegible]																														

Name	1. Metabolites affected by drug only Day 9 ami_50/Vehicle p.value	FDR	old.Chang	log2.FC	1. Metabolites affected by drug only Day 9 ami_200/Vehicle p.value	FDR	old.Chang	log2.FC	2. Dose response Day 9 Rad + ami_200/Rad + ami_50 p.value	FDR	old.Chang	log2.FC	Metabolites dysregulated by radiation o Day 9 Rad/Vehicle p.value	FDR	old.Chang	log2.FC	4. Metabolites that return to normal leve Day 9 Rad + ami_50/Rad p.value	FDR	old.Chang	log2.FC	4. Metabolites that return to normal level Day 9 Rad + ami_200/Rad p.value	FDR	old.Chang	log2.FC	5. Does the dose make a difference? Day 9 ami_200/ami_50 p.value	FDR	old.Chang	log2.FC	Does the dose make a differen Day 9 ad + ami_200/Rad + ami_50/Ra p.value	FDR	f.value
PI(18:4(6Z,9Z,12Z,1	0.14627	0.86174	0.86416	-0.2106	0.69624	0.9083	0.97214	-0.0408	0.0003881	0.0043587	2.1473	1.1025	0.0002447	0.0012244	0.45114	-1.1484	0.10687	0.72814	0.66971	-0.5784	0.063852	0.13349	1.4381	0.5242	0.25569	0.99929	1.125	0.1699	0.0022538	0.028083	8.3953
Undecylprodigiosin	0.14402	0.86174	1.2243	0.2919	0.7547	0.91953	1.0096	0.0137	0.39154	0.44366	0.83298	-0.2637	0.83392	0.86459	0.93097	-0.1032	0.78236	0.85386	1.0616	0.0862	0.59104	0.70846	0.88429	-0.1774	0.12598	0.99929	0.82462	-0.2782	0.68147	0.70845	0.39096
Cysteinyl-Glycine	0.57917	0.99919	0.89543	-0.1594	0.023967	0.90043	0.63321	-0.6592	0.025364	0.052009	1.6615	0.7325	1.993E-05	0.0004337	0.24713	-2.0166	0.093005	0.72814	0.64451	-0.6337	0.7397	0.82272	1.0708	0.0987	0.052338	0.81444	0.70716	-0.4999	0.093219	0.16021	2.678
trans,octacis-Deca	0.18604	0.88676	1.1727	0.2298	0.48612	0.90043	1.1042	0.143	3.83E-05	7.21E-04	1.9307	0.9491	0.69199	0.73585	0.97586	-0.0353	0.28234	0.72814	0.68097	-0.5544	0.12878	0.21709	1.3147	0.3948	0.51086	0.99929	0.94159	-0.0868	0.011802	0.061657	5.5885
GlcCer(d14:2(4E,6E)	0.64904	0.99919	0.87997	-0.1845	0.63985	0.90043	0.89984	-0.1523	0.0011624	0.0096018	0.48348	-1.0485	7.44E-05	0.000775	5.1976	2.3778	0.97384	0.98083	0.87817	-0.1874	0.015349	0.096534	0.42458	-1.2359	0.95379	0.99929	1.0226	0.0322	0.0038138	0.04078	7.4527
PA(19:1(9Z)/22:4(7	0.65587	0.99919	0.91683	-0.1253	0.84435	0.95055	0.95219	-0.0707	0.1378	0.18039	0.84061	-0.2505	0.0028396	0.005073	2.2974	1.2	0.39797	0.72814	0.78253	-0.3538	0.094982	0.17183	0.65781	-0.6043	0.78164	0.99929	1.0386	0.0546	0.15157	0.22097	2.0764
Phosphoagmatine;Ag[illegible]	0.47306	0.99919	0.88683	-0.1733	0.6405	0.90043	0.94231	-0.0857	0.034553	0.062772	0.73818	-0.438	9.59E-04	0.0025657	2.5061	1.3254	0.40981	0.72814	0.79034	-0.3395	0.032485	0.10116	0.58342	-0.7774	0.88743	0.99929	1.0626	0.0875	0.039051	0.097914	3.8305
Arg Lys His	0.36752	0.99919	0.86574	-0.208	0.96211	0.98473	0.98247	-0.0255	0.010497	0.029884	0.72523	-0.4635	0.0006663	0.0021119	2.4983	1.3209	0.45327	0.72814	0.81079	-0.3026	0.024332	0.096735	0.58801	-0.7661	0.36484	0.99929	1.1348	0.1825	0.022655	0.074987	4.6044
Idebenone Metaboli	0.27814	0.96071	0.84436	-0.2441	0.25631	0.90043	0.84042	-0.2508	0.036315	0.064615	0.75398	-0.4074	0.0015513	0.0033426	2.3557	1.2361	0.40771	0.72814	0.78947	-0.3411	0.035947	0.10426	0.59525	-0.7484	0.87761	0.99929	0.99533	-0.0068	0.047197	0.1085	3.5709
LPIM3(16:0/0:0)	0.036871	0.72287	0.75549	-0.4045	0.4187	0.90043	0.90758	-0.1399	1.93E-02	0.043639	1.4555	0.5415	0.044678	0.061657	0.70016	-0.5142	0.19882	0.72814	0.72971	-0.4546	0.59364	0.71133	1.0621	0.0869	0.097295	0.99287	1.2013	0.2646	0.11464	0.18209	2.4185
PG(14:1(9Z)/18:4(6	0.70011	0.99919	1.09	0.1244	0.33605	0.90043	0.87951	-0.1852	0.026958	0.053775	0.74665	-0.4215	0.0009737	0.0025798	2.777	1.4735	0.37096	0.72814	0.73753	-0.4392	0.036788	0.10525	0.55068	-0.8607	0.24578	0.99929	0.80685	-0.3096	0.051266	0.11253	3.4591
PC(2:0/O-18:0)[U]	0.45941	0.99919	1.0791	0.1099	0.23759	0.90043	1.116	0.1583	0.017618	0.041235	0.7084	-0.4974	0.66352	0.70931	1.0375	0.0531	0.89392	0.93384	0.85967	-0.2182	0.15787	0.25609	0.60899	-0.7155	0.71486	0.99929	1.0342	0.0485	0.18597	0.25834	1.8319
[illegible]	[illegible]																														

Type	RT	erimental.	Adduct	PPM.Error	olecular.Weig	Name	Formula	Kegg	HMDB	LipidMaps	Metlin
Bonemar	5.7	302.3049	M+H	2	301.298079	Safingol (L-threo-sp	C18H39NO2			LMSP0108	53955
Bonemar	5.62	460.2674	M+H	2	459.2594	Arg Trp Val	C22H33N7O4				15690
Bonemar	0.33	1017.7892	M+H	8	1016.773912	DAT(18:0/24:0(2M	C57H108O14			LMSL0300	82438
Bonemar	8.19	545.4011	M+Na	9	522.4072956	Panaxydol linoleate	C35H54O3		HMDB0041205		95626
Bonemar	9.66	449.3596	M+H	7	448.35526	Teasterone	C28H48O4	C15791			64149
Bonemar	11.79	180.1033	M+H	8	179.0946287	N-methylphenylalan	C10H13NO2		HMDB0029224		86103
Bonemar	8.95	671.4561	M+Na	9	648.4730062	PA(i-24:0/8:0)	C35H69O8P		HMDB0115932		
Bonemar	8.19	399.216	M+H	2	398.2093241	Galbanic acid	C24H30O5		HMDB0030163		86766
Bonemar	9.04	391.2846	M+Na	7	368.2926598	Octadecyl fumarate	C22H40O4		HMDB003	LMFA0701	92842
Bonemar	8.9	663.5047	M+H	10	662.4910252	DG(20:4(8Z,11Z,14	C43H66O5		HMDB0007549		59037
Bonemar	6.36	235.1676	M+H	7	234.1619799	Cyclodehydroisolubi	C15H22O2		HMDB0037038		92017
Bonemar	9.98	737.5759	M+H	9	736.561824	Mannosyl-1beta-ph	C40H81O9P			LMPK0100	82404
Bonemar	7.27	309.2021	M+Na	5	286.2144094	Thapsic acid	C16H30O4	C19615	HMDB000	LMFA0117	5642
Bonemar	0.36	206.065	M+H	9	205.0595055	lipoamide	C8H15NOS	C00248	HMDB000	LMFA0801	3372
Bonemar	7.41	504.3062	M+H	5	503.3011893	PE(20:3(8Z,11Z,14Z	C25H46NO7P		HMDB001	LMGP0205	77688
Bonemar	8.98	688.4954	M+H	6	687.4839047	PE(18:1(9Z)/14:1(9	C37H70NO8P		HMDB000	LMGP0201	60470
Bonemar	5.1	291.2683	M+H	0	290.261	ent-13-epi-Manoyl	C20H34O	C21714			
Bonemar	7.56	355.2826	M+H	5	354.2770097	MG(18:2(9Z,12Z)/0	C21H38O4		HMDB0011568		
Bonemar	6.76	205.087	M+H	5	204.07864	Levulinic Acid, 3-Ben	C12H12O3				44382
Bonemar	6.72	397.0978	M+Na	8	374.1114	Tyr-Ala-OH	C18H18N2O7				64984
Bonemar	9.43	441.2976	M+H	5	440.2926598	Suillin	C28H40O4		HMDB0034349		89868
Bonemar	5.04	245.0818	M+H	4	244.0735589	Oxyresveratrol	C14H12O4	C10273	HMDB012	LMPK1309	53239
Bonemar	9.67	469.3383	M+H	9	468.335193	N-oleoyl tryptophan	C29H44N2O3			LMFA0807	75478
Bonemar	7.1	639.4067	M+H	7	638.3947559	PA(14:1(9Z)/18:4(6	C35H59O8P		HMDB011	LMGP1001	81288
Bonemar	6.59	258.2407	M+H	8	257.23548	D-erythro-Sphingosi	C15H31NO2				64626
Bonemar	9.99	736.5752	M+Na	8	713.58057	GlcCer(d15:1/20:0)	C41H79NO8			LMSP0501	103287
Bonemar	10.17	969.8302	M+H	3	968.8196766	TG(22:5(4Z,7Z,10Z,	C65H108O5		HMDB0055141		
Bonemar	9.43	442.301	M+Na	7	419.314792	N-oleoyl histidine	C24H41N3O3			LMFA0807	75515
Bonemar	6.55	377.2068	M+H	10	376.1958	Lys Glu Thr	C15H28N4O7				17285
Bonemar	7.34	426.2181	M+H	6	425.21352	Asn His Arg	C16H27N9O5				18941
Bonemar	7.11	391.123	M+Na	2	368.1332	Asn-Lys-OH	C15H20N4O7				65267
Bonemar	9.05	396.2925	M+H	7	395.28243	N-(3-hydroxypheny	C26H37NO2				45072
Bonemar	5.67	383.1689	M+Na	0	360.17976	Gly Val Trp	C18H24N4O4				15975
Bonemar	9.03	420.2278	M+H	9	419.21687	Lys Ser Trp	C20H29N5O5				17515
Bonemar	7.15	331.2235	M+H	10	330.2194948	Testosterone acetate	C21H30O3	C03027	HMDB006	LMST0202	41854
Bonemar	9.84	791.5588	M+Na	3	768.5669066	PA(22:1(13Z)/19:0	C44H81O8P		HMDB0115591		
Bonemar	9.45	421.3281	M+H	7	420.32396	Dihomoursodeoxych	C26H44O4			LMST04020033	
Bonemar	6.38	249.1847	M+Na	10	226.19328	Physeteric acid	C14H26O2				34738
Bonemar	9.81	937.7035	M+H	7	936.703069	PI(O-20:0/22:0)	C51H101O12P			LMGP0602	81054
Bonemar	6.3	330.3334	M+H	9	329.32319	Oleoyl Ethanolamide	C20H35D4NO2				96544
Bonemar	8.91	619.474	M+H	7	618.462444	PA(P-16:0/15:0)	C34H67O7P			LMGP1003	82245
Bonemar	8.51	397.2157	M+H	9	396.21212	Ile His Gln	C17H28N6O5				16176
Bonemar	8.52	395.2203	M+H	2	394.212043	PA(15:1(9Z)/0:0)	C18H35O7P			LMGP1005	82340
Bonemar	9.09	393.2976	M+H	6	392.2926598	Hyodeoxycholic acid	C24H40O4		HMDB0000733		5701
Bonemar	8.78	464.2335	M+H	2	463.22533	Trp Lys Met	C22H33N5O4S				15642
Bonemar	6.74	336.1295	M+Na	5	313.13862	Gly His Thr	C12H19N5O5				17161
Bonemar	8.21	457.3105	M+Na	4	434.319624	(10Z)-19-fluoro-1alp	C27H43FO3			LMST0302	42150
Bonemar	8.04	425.2876	M+Na	7	402.29545	Lys Lys Lys	C18H38N6O4				18451
Bonemar	6.78	381.0872	M+H	2	380.08071	O-Acetylserine	C13H21N2O7PS				6809
Bonemar	11.97	903.5049	M+H	3	902.494534	PI(18:4(6Z,9Z,12Z,1	C49H75O13P			LMGP0601	80445
Bonemar	9.96	719.5745	M+H	7	718.562491	PE-Cer(d15:1(4E)/2	C39H79N2O7P			LMSP0302	103114
Bonemar	11.86	481.257	M+Na	2	458.2668389	Lucidenic acid A	C27H38O6		HMDB0037611		92454
Bonemar	8.99	693.4552	M+H	9	692.4417061	PA(18:3(9Z,12Z,15Z	C39H65O8P		HMDB011	LMGP1001	40914
Bonemar	6.8	579.293	M+Na	5	556.301253	PI(P-16:0/0:0)	C25H49O11P			LMGP0607	81203
Bonemar	0.33	1051.7822	M+Na	9	1028.783291	TG(22:5(4Z,7Z,10Z,1	C69H104O6		HMDB0055125		
Bonemar	0.33	1187.7599	M+H	8	1186.742567	CL(1'-[18:2(9Z,12Z),	C63H112O16P2			LMGP1202	82365
Bonemar	6.26	328.0926	M+Na	7	305.1011706	Entacapone	C14H15N3	C07943	HMDB0012226		62873
Bonemar	9.82	923.706	M+H	7	922.7050407	TG(20:4(8Z,11Z,14Z	C61H94O6		HMDB0055656		
Bonemar	9.92	925.7248	M+Na	1	902.7363409	TG(20:4(8Z,11Z,14Z	C59H98O6		HMDB0055546		
Bonemar	6.78	480.3117	M+H	7	479.3011893	PE(18:1(9Z)/0:0)	C23H46NO7P		HMDB001	LMGP0205	40778
Bonemar	0.38	368.0352	M+Na	4	345.0474343	Guanosine 2',3'-cycl	C10H12N5	C06194	HMDB0011629		3487
Bonemar	9.03	805.5385	M+Na	4	782.546174	PG(P-20:0/18:4(6Z,	C44H79O9P			LMGP0403	79976
Bonemar	9.65	827.549	M+H	2	826.5397067	Pyropheophytin b	C53H70N4O4		HMDB0034248		89802
Bonemar	6.24	526.2931	M+H	1	525.2855393	PE(22:6(4Z,7Z,10Z,1	C27H44NO7P		HMDB001	LMGP0205	77679
Bonemar	6.68	399.251	M+Na	1	376.2613596	MG(0:0/20:5(5Z,8Z,	C23H36O4		HMDB001	LMGL0101	62333
Bonemar	6.26	568.3395	M+H	0	567.3324895	PC(22:6(4Z,7Z,10Z,1	C30H50NO7P		HMDB001	LMGP0105	61713
Bonemar	9.75	574.4195	M+Na	2	551.4314752	PC(O-16:0/O-5:0)[U	C29H62NO6P				40217
Bonemar	8.09	331.2653	M+H	6	330.25588	Clupadonic acid	C22H34O2			LMFA04000082	
Bonemar	6.26	327.0886	M+Na	8	304.1019	Asp Asn Gly	C10H16N4O7				15760
Bonemar	6.69	299.1639	M+Na	8	276.1725446	Panaquinquecol 2	C17H24O3		HMDB003	LMFA0500	93549
Bonemar	6.26	350.0593	M+Na	1	327.07026	Asn-Ser-OH	C12H13N3O8				65147
Bonemar	7.08	353.2672	M+Na	3	330.2770097	MG(16:0/0:0/0:0)	C19H38O4		HMDB001	LMGL0101	3855
Bonemar	9.09	384.2243	M+Na	7	361.23252	Lys Ser Lys	C15H31N5O5				19739
Bonemar	9.83	731.5702	M+H	1	730.562491	PE-Cer(d14:1(4E)/2	C40H79N2O7P			LMSP0302	103104
Bonemar	9.03	219.9994	M+Na	1	197.0100211	O-phosphonato-L-h	C4H8NO6P		HMDB0062692		
Bonemar	7.4	484.3222	M+H	10	483.3196	Tumonoic Acid H	C26H45NO7				65436
Bonemar	9.46	422.3286	M+H	5	421.3192089	Gamma-linolenyl car	C25H43NO4		HMDB000	LMFA0701	58389
Bonemar	9.03	332.1194	M+Na	7	309.13247	Ser Gly Phe	C14H19N3O5				16478
Bonemar	12	645.3084	M+H	8	644.296168	PI(22:6(4Z,7Z,10Z,1	C31H49O12P			LMGP0605	81177
Bonemar	8.86	622.5218	M+H	8	621.50973	PE-NMe(O-14:0/O-1	C34H72NO6P				40770
Bonemar	6.58	341.1043	M+Na	8	318.11755	Asn Asp Ala	C11H18N4O7				16102
Bonemar	12.07	834.5239	M+Na	2	811.5363342	PS(18:0/20:4(5Z,8Z	C44H78NO10P		HMDB001	LMGP0301	40824
Bonemar	12.07	859.5287	M+Na	2	836.5414792	PI(18:1(9Z)/16:0)	C43H81O13P		HMDB000	LMGP0601	61219
Bonemar	7.41	546.3556	M+Na	5	523.3637704	enantio-PAF C-16	C26H54NO7P				43413
Bonemar	9.98	838.6289	M+H	4	837.6247553	PC(20:3(5Z,8Z,11Z)	C48H88NO8P		HMDB000	LMGP0101	59820
Bonemar	9.68	713.5097	M+H	3	712.504309	PA(17:1(9Z)/20:2(1	C40H73O8P			LMGP1001	81432
Bonemar	8.08	337.178	M+Na	2	314.1882	Estradiol acetate	C20H26O3				43499
Bonemar	9.91	873.6967	M+H	0	872.6893907	TG(18:4(6Z,9Z,12Z,1	C57H92O6		HMDB0055411		
Bonemar	12.07	858.5247	M+Na	1	835.5363342	PS(18:0/22:6(4Z,7Z	C46H78NO10P		HMDB001	LMGP0301	40825
Bonemar	9.1	423.2494	M+H	3	422.243343	PA(17:1(9Z)/0:0)	C20H39O7P			LMGP1005	3887
Bonemar	0.58	223.9643	M+Na	7	200.9765637	Cysteine-S-sulfate	C3H7NO5S	C05824	HMDB0000731		58010
Bonemar	8.12	263.2364	M+Na	8	240.2453156	Palmitaldehyde	C16H32O	C00517	HMDB000	LMFA0600	6317
Bonemar	9.97	911.7185	M+H	7	910.7050407	TG(15:0/22:6(4Z,7Z	C60H94O6		HMDB0043797		
Bonemar	6.23	385.2746	M+Na	9	362.282095	Norlithocholic acid	C23H38O3			LMST04060023	
Bonemar	0.33	1053.7826	M+Na	4	1030.789562	DAT(18:0/25:0(2M	C58H110O14			LMSL0300	82439
Bonemar	6.65	708.4865	M+H	8	707.4737345	PS(16:0/14:0)	C36H70NO10P		HMDB001	LMGP0301	78634
Bonemar	8.6	677.6063	M+H	2	676.6005757	DG(22:0/18:2(9Z,12	C43H80O5		HMDB0007596		59077
Bonemar	9	401.303	M+H	5	400.2977451	Testosterone enanth	C26H40O3	C08157	HMDB000	LMST0202	2785
Bonemar	5.8	338.1135	M+Na	3	315.12528	Cys Pro Pro	C13H21N3O4S				18760
Bonemar	8.77	390.3004	M+H	0	389.292994	N-arachidonoyl GAB	C24H39NO3			LMFA0807	46556
Bonemar	0.33	1100.7602	M+H	2	1099.751143	MIPC(d18:0/26:0(2	C56H110NO17P			LMSP0303	103188
Bonemar	9.99	864.6554	M+H	9	863.6404054	PC(22:2(13Z,16Z)/2	C50H90NO8P		HMDB0008605		60051
Bonemar	11.89	805.5245	M+H	2	804.515269	PI(P-16:0/17:2(9Z,1	C42H77O12P			LMGP0607	81089
Bonemar	6.75	303.1753	M+H	7	302.17025	Gamma-glutamyl-Ar	C11H22N6O4				86035
Bonemar	6.66	518.3211	M+H	6	517.3168394	PC(18:3(6Z,9Z,12Z)	C26H48NO7P				40303
Bonemar	11.88	816.5541	M+H	0	815.546505	PE(22:5(7Z,10Z,13Z	C47H78NO8P		HMDB0009661		61050
Bonemar	8.59	657.4412	M+Na	8	634.4573561	PA(i-14:0/a-17:0)	C34H67O8P		HMDB0115813		
Bonemar	9.63	826.54	M+H	2	825.5308549	PC(20:5(5Z,8Z,11Z,	C48H76NO8P		HMDB000	LMGP0101	59957
Bonemar	5.8	321.1327	M+Na	6	298.14164	Idebenone Metaboli	C15H22O6				772
Bonemar	6.63	454.2923	M+H	1	453.2855393	PC(13:0/0:0)[U]	C21H44NO7P				40277
Bonemar	11.4	711.5969	M+H	7	710.584926	DG(21:0/22:6(4Z,7Z	C46H78O5			LMGL0201	4665
Bonemar	11.98	829.4981	M+H	1	828.4917656	PGP(18:1(11Z)/16:0	C40H78O13P2		HMDB0013517		
Bonemar	11.39	478.142	M+Na	6	455.1553314	N5,N10-Methenyltet	C20H21N7O6				712
Bonemar	8.29	307.2642	M+H	3	306.25588	Linolenic Acid ethyl e	C20H34O2				45006
Bonemar	11.39	699.5885	M+H	5	698.5849256	DG(20:0/22:5(4Z,7Z	C45H78O5		HMDB0007380		58908
Bonemar	6.42	528.3082	M+H	1	527.3011893	LysoPE(0:0/22:5(4Z	C27H46NO7P		HMDB001	LMGP0205	62282
Bonemar	12.07	860.5362	M+H	9	859.5363347	PS(22:4(7Z,10Z,13Z	C48H78NO10P		HMDB011	LMGP0301	78474
Bonemar	10.05	588.5381	M+Na	10	565.5433952	N-Stearoylsphingosi	C36H71NO3		HMDB0000829		
Bonemar	11.85	817.5134	M+Na	3	794.521404	SQDG(16:0/16:0)	C41H78O12S			LMGL0501	46640
Bonemar	8.7	661.5751	M+H	2	660.5692755	DG(20:1n9/0:0/18:	C42H76O5		HMDB0056220		
Bonemar	12.06	830.4935	M+H	4	829.489384	PS(22:6(4Z,7Z,10Z,1	C46H72NO10P		HMDB001	LMGP0301	78495
Bonemar	9.71	830.5682	M+H	1	829.5621551	PC(18:2(9Z,12Z)/22	C48H80NO8P		HMDB000	LMGP0101	59602
Bonemar	9.87	779.5676	M+H	1	778.559501	MGDG(18:2(9Z,12Z	C45H78O10			LMGL0501	46659
Bonemar	9.04	333.128	M+H	4	332.1219656	Mytilin A	C13H20N2O8		HMDB0039002		93608
Bonemar	11.87	571.2808	M+H	1	570.272987	Pipercyclobutanami	C34H38N2O6		HMDB0036360		91504
Bonemar	10.98	861.641	M+Na	8	838.6451569	PA(24:1(15Z)/22:2(	C49H91O8P		HMDB0115475		
Bonemar	6.82	383.182	M+Na	5	360.1909887	Tryptophyl-Arginine	C17H24N6O3		HMDB0029077		85970
Bonemar	10.01	866.6646	M+H	1	865.6560554	PC(20:3(5Z,8Z,11Z)	C50H92NO8P		HMDB000	LMGP0101	59828
Bonemar	6.66	496.3398	M+Na	0	473.350509	Clupanodonyl carnit	C29H47NO4		HMDB000	LMFA0707	58428
Bonemar	10.88	872.638	M+H	1	871.6302351	PS(24:1(15Z)/18:1(	C48H90NO10P		HMDB0112916		
Bonemar	10.88	858.6224	M+H	1	857.614588	PS(20:2(11Z,14Z)/2	C47H88NO10P			LMGP0301	78247
Bonemar	8.61	591.4509	M+Na	5	568.464416	Spheroidene	C41H60O	C15900		LMPR0107	41376
Bonemar	10.87	963.6823	M+H	8	962.682334	PI(21:0/22:1(11Z))	C52H99O13P			LMGP0601	80690
Bonemar	10.97	860.6364	M+H	1	859.630238	PS(20:1(11Z)/21:0)	C47H90NO10P			LMGP0301	78216
Bonemar	9.96	901.7228	M+Na	3	878.7363409	TG(20:3n6/14:0/20	C57H98O6		HMDB0053653		
Bonemar	8.45	333.2783	M+H	2	332.2715304	Ethyl Arachidonate	C22H36O2		HMDB0062725		404
Bonemar	10.1	867.6864	M+Na	6	844.6921071	PA(24:0/22:0)	C49H97O8P		HMDB0115446		
Bonemar	11.89	574.2818	M+H	9	573.27986	Z-Gly-Pro-Leu-Gly-P	C28H39N5	C03183			65925
Bonemar	9.1	615.4431	M+Na	8	592.4491604	Ubiquinol-6	C39H60O4		HMDB0012299		62918
Bonemar	9.96	891.6849	M+Na	4	868.6921071	PA(24:1(15Z)/24:1(	C51H97O8P		HMDB0115481		
Bonemar	10.02	850.662	M+H	8	849.6611408	PC(o-22:1(13Z)/20:	C50H92NO7P		HMDB0013451		
Bonemar	10.82	882.6243	M+H	3	881.614588	PS(22:4(7Z,10Z,13Z	C49H88NO10P			LMGP0301	78476
Bonemar	10.97	874.6508	M+H	3	873.6458852	PS(20:1(11Z)/22:0)	C48H92NO10P		HMDB011	LMGP0301	78217
Bonemar	9.79	832.5841	M+H	1	831.5778051	PC(18:2(9Z,12Z)/22	C48H82NO8P		HMDB000	LMGP0101	59600
Bonemar	9.75	1199.8003	M+H	6	1198.800076	CL(8:0/a-13:0/15:0,	C62H120O17P2		HMDB0120189		
Bonemar	9.98	892.6852	M+H	7	891.6717055	PC(20:5(5Z,8Z,11Z,	C52H94NO8P		HMDB0008519		59965
Bonemar	9.87	778.5629	M+H	5	777.5519848	PS(15:0/20:0)	C41H80NO10P		HMDB011	LMGP0301	77814
Bonemar	0.41	291.0701	M+Na	0	268.0807695	Inosine	C10H12N4	C00294	HMDB0000195		84
Bonemar	12.06	831.4975	M+H	5	830.494534	PI(16:1(9Z)/18:3(6Z	C43H75O13P			LMGP0601	80202
Bonemar	10.83	896.6396	M+H	2	895.6302351	PS(22:2(13Z,16Z)/2	C50H90NO10P		HMDB011	LMGP0301	78448
Bonemar	9.68	853.5871	M+H	8	852.572784	PI(19:0/16:0)	C44H85O13P			LMGP0601	80947
Bonemar	7.9	352.1934	M+H	8	351.1834437	Marmeline	C22H25NO3		HMDB0030192		86785
Bonemar	0.33	1168.7467	M+H	8	1167.749216	KDNalpha2-3Galbet	C59H109NO21			LMSP0601	56788
Bonemar	9.87	882.6577	M+Na	2	859.666623	PS(O-20:0/22:1(11Z	C48H94NO9P			LMGP0302	78713
Bonemar	6.28	544.3392	M+H	1	543.3324895	PC(20:4(5Z,8Z,11Z,1	C28H50NO7P		HMDB001	LMGP0105	61705
Bonemar	7.53	550.3893	M+H	5	549.3794196	PC(O-18:1(9E)/2:0)[	C28H56NO7P				40012
Bonemar	9.88	899.7023	M+Na	9	876.7206908	TG(16:1(9Z)/22:5(7	C57H96O6		HMDB0049007		
Bonemar	11.95	979.4828	M+Na	5	956.4980803	Phytolaccoside F	C48H76O19		HMDB0040880		95334
Bonemar	11.89	547.2809	M+H	6	546.2703	Trp Trp Arg	C28H34N8O4				17291
Bonemar	10.02	788.6108	M+H	7	787.6091052	PC(22:1(13Z)/14:0)	C44H86NO8P		HMDB000	LMGP0101	60004
Bonemar	9.71	698.5166	M+H	7	697.5046402	PE(18:3(6Z,9Z,12Z)/	C39H72NO7P		HMDB0009147		60554
Bonemar	9.77	678.5116	M+H	7	677.4995548	PC(16:0/12:0)[U]	C36H72NO8P				39300
Bonemar	11.96	899.4981	M+H	2	898.4926009	Pitheduloside D	C46H74O17		HMDB0031358		87611
Bonemar	0.33	965.7845	M+Na	9	942.8040265	TG(20:4(5Z,8Z,11Z,1	C63H106O5		HMDB0054334		
Bonemar	6.1	494.3212	M+H	6	493.3168394	PC(16:1(9Z)/0:0)[U]	C24H48NO7P				40289
Bonemar	11.89	820.5257	M+Na	7	797.541817	PI-Cer(t20:0/14:0)	C40H80NO12P			LMSP03030153	
Bonemar	9.09	454.3326	M+H	7	453.3219248	PC(O-12:0/O-2:0)	C22H48NO6P				40190
Bonemar	6.83	440.3175	M+H	9	439.3062747	PC(O-12:0/O-1:0)	C21H46NO6P				40186
Bonemar	11.96	1012.4859	M+Na	7	989.4897	Cobinamide:Cob(I)i	C48H72Co	C05774			
Bonemar	7.01	548.3726	M+H	3	547.3637704	PC(O-18:2(9Z,12Z)/	C28H54NO7P				40020
Bonemar	12.13	943.4868	M+H	3	942.4824302	Phytolaccoside I	C47H74O19		HMDB0040810		95273
Bonemar	9.04	425.2264	M+H	8	424.2226051	PA(8:0/8:0)	C19H37O8P		HMDB011	LMGP1001	40919
Bonemar	10.08	839.6559	M+Na	7	816.660807	PA(i-19:0/a-25:0)	C47H93O8P		HMDB0115824		
Bonemar	9.96	849.6499	M+H	9	848.650639	PG(21:0/20:0)	C47H93O10P			LMGP0401	79517
Bonemar	6.68	531.3043	M+Na	3	508.3165055	PA(10:0/i-12:0)	C25H49O8P		HMDB0115611		
Bonemar	11.84	800.552	M+Na	6	777.56724	PE(O-18:0/22:6(4Z	C45H80NO7P				76406
Bonemar	10.06	878.6645	M+H	1	877.6560554	PE(22:5(4Z,7Z,10Z,1	C51H92NO8P		HMDB0009640		61029
Bonemar	10.07	840.6537	M+H	7	839.6404054	PC(20:3(5Z,8Z,11Z)	C48H90NO8P		HMDB000	LMGP0101	59819
Bonemar	9.72	802.5381	M+H	0	801.5308549	PC(20:4(8Z,11Z,14Z	C46H76NO8P		HMDB0008470		59916
Bonemar	11.87	822.5342	M+H	8	821.520688	PS(22:4(7Z,10Z,13Z	C45H76NO10P			LMGP0301	78461
Bonemar	9.9	732.5558	M+H	3	731.546505	PC(14:0/18:1(9E))[U	C40H78NO8P				39233
Bonemar	10.71	931.6258	M+H	1	930.619734	PI(19:1(9Z)/22:2(13	C50H91O13P			LMGP0601	80498
Bonemar	11.87	823.5306	M+Na	7	800.5356064	PA(22:2(13Z,16Z)/2	C47H77O8P		HMDB011	LMGP1001	81951
Bonemar	11.99	853.4813	M+H	6	852.478884	PI(22:6(4Z,7Z,10Z,1	C45H73O13P			LMGP0601	80811
Bonemar	10.09	855.6537	M+Na	8	832.6580905	TG(18:4(6Z,9Z,12Z,1	C54H88O6		HMDB0055363		
Bonemar	9.85	690.5442	M+H	1	689.5359403	PC(O-14:0/16:1(9Z)	C38H76NO7P		HMDB001	LMGP0102	40043

													1. Metabolites affected by drug only				1. Metabolites affected by drug only				2. Dose response				. Metabolites dysregulated by radiation o				4. Metabolites that return to normal leve				4. Metabolites that return to normal level				5. Does the dose make a difference?				Does the dose make a differen			1. Metabolites affected by drug only				1. Metabolites affected by drug only				2. Dose response				. Metabolites dysregulated by radiation o				4. Metabolites that return to normal leve				4. Metabolites that return to normal level				5. Does the dose make a difference?				Does the dose make a differen		
													Day 4				Day 4				Day 4				Day 4				Day 4				Day 4				Day 4				Day 4			Day 9				Day 9				Day 9				Day 9				Day 9				Day 9				Day 9				Day 9		
													ami_50/Vehicle				ami_200/Vehicle				Rad + ami_200/Rad + ami_50				Rad/Vehicle				Rad + ami_50/Rad				Rad + ami_200/Rad				ami_200/ami_50				ad + ami_200/Rad + ami_50/R			ami_50/Vehicle				ami_200/Vehicle				Rad + ami_200/Rad + ami_50				Rad/Vehicle				Rad + ami_50/Rad				Rad + ami_200/Rad				ami_200/ami_50				ad + ami_200/Rad + ami_50/Ra		
Type	RT	erimental.i	Adduct	PPM.Error	olecular.Weig	Name	Formula	Kegg	HMDB	LipidMaps	Metlin	PubChem	p.value	FDR	old.Chang	log2.FC	p.value	FDR	old.Chang	log2.FC	p.value	FDR	old.Chang	log2.FC	p.value	FDR	old.Chang	log2.FC	p.value	FDR	old.Chang	log2.FC	p.value	FDR	old.Chang	log2.FC	p.value	FDR	old.Chang	log2.FC	p.value	FDR	f.value	p.value	FDR	old.Chang	log2.FC	p.value	FDR	old.Chang	log2.FC	p.value	FDR	old.Chang	log2.FC	p.value	FDR	old.Chang	log2.FC	p.value	FDR	old.Chang	log2.FC	p.value	FDR	old.Chang	log2.FC	p.value	FDR	old.Chang	log2.FC	p.value	FDR	f.value
Bonemar	7.6	467.3112	M+H	4	466.3059409	LysoPA(i-20:0/0:0)	C23H47O7P		HMDB0114767			17786813	0.36266	0.99994	0.89232	-0.1644	0.4271	0.99946	1.0805	0.1118	0.13967	0.17836	1.1515	0.2035	0.18161	0.20867	0.84168	-0.2487	0.12194	0.9998	0.83471	-0.2607	0.77209	0.81051	0.96118	-0.0571	0.056874	0.99932	1.2109	0.2761	0.20348	0.25136	1.7192	0.31276	0.98129	1.1068	0.1464	0.44207	0.90043	1.09	0.1243	0.0017675	0.012245	1.633	0.7075	0.55759	0.61401	0.99082	-0.0133	0.12739	0.72814	0.60022	-0.7364	0.72187	0.80916	0.98014	-0.0289	0.84882	0.99929	0.98482	-0.0221	0.070571	0.13483	3.0358
Bonemar	12.17	838.5516	M+Na	6	815.5676348	PS(20:1(11Z)/18:1	C44H82NO10P		HMDB011	LMGP0301	78204	52925650	0.81432	0.99994	1.0006	0.0008	0.82967	0.99946	0.96032	-0.0584	0.26708	0.31476	1.1623	0.2169	0.089481	0.10692	0.78694	-0.3457	0.82453	0.9998	1.1274	0.173	0.042956	0.066008	1.3103	0.3899	0.49214	0.99932	0.95979	-0.0592	0.28662	0.33595	1.327	0.10095	0.82631	0.82588	-0.276	0.93964	0.9816	1.0065	0.0094	0.0054449	0.021115	1.6735	0.7428	0.0054322	0.0088077	0.61933	-0.6912	0.10467	0.72814	0.6856	-0.5446	0.37719	0.5079	1.1473	0.1983	0.095332	0.98452	1.2187	0.2854	0.026041	0.080534	4.4024
Bonemar	10.71	945.6397	M+H	3	944.635384	PI(22:0/20:3(8Z,11	C51H93O13P			LMGP0601	80709		0.54842	0.99994	0.91072	-0.1349	0.40742	0.99946	1.1136	0.1552	0.072066	0.10457	1.2082	0.2729	0.45467	0.48909	1.1121	0.1532	0.28039	0.9998	0.85592	-0.2245	0.66745	0.71551	1.0341	0.0484	0.10904	0.99932	1.2227	0.2901	0.24476	0.29349	1.5062	0.13503	0.85101	1.1917	0.2531	0.94623	0.98222	1.005	0.0073	0.0056679	0.02149	1.4241	0.51	0.64755	0.69461	1.1832	0.2427	0.1551	0.72814	0.68571	-0.5443	0.76298	0.83883	0.97651	-0.0343	0.14156	0.99929	0.84335	-0.2458	0.094807	0.16149	2.6566
Bonemar	9.79	739.5246	M+H	4	738.519959	PA(22:2(13Z,16Z)/1	C42H75O8P			LMGP1001	81931		0.60695	0.99994	0.93208	-0.1015	0.74303	0.99946	0.95546	-0.0657	0.70215	0.74003	1.0293	0.0417	0.56005	0.59481	1.0631	0.0882	0.39861	0.9998	0.90984	-0.1363	0.55998	0.61174	0.93651	-0.0946	0.84683	0.99932	1.0251	0.0358	0.64872	0.67831	0.44179	0.53394	0.99919	0.90842	-0.1386	0.15757	0.90043	0.82554	-0.2766	0.0067759	0.023609	1.5088	0.5934	0.80064	0.83436	0.98656	-0.0195	0.23097	0.72814	0.76542	-0.3857	0.27649	0.39885	1.1549	0.2077	0.31429	0.99929	0.90876	-0.138	0.047147	0.1085	3.5723
Bonemar	8.6	309.2812	M+H	8	308.27153	Hormelic acid	C20H36O2			LMFA01140034			0.20519	0.99994	0.59531	-0.7483	0.80366	0.99946	1.1907	0.2519	0.17898	0.22078	0.52623	-0.9262	0.55468	0.58966	0.96608	-0.0498	0.25891	0.9998	2.0554	1.0394	0.57738	0.62835	1.0816	0.1132	0.16326	0.99932	2.0002	1.0002	0.25957	0.30796	1.4392	0.8549	0.99919	1.0423	0.0598	0.96169	0.98459	0.84393	-0.2448	0.0070055	0.024068	0.35521	-1.4933	0.034455	0.048344	3.1317	1.6469	0.95811	0.97311	0.80938	-0.3051	0.037109	0.10564	0.2875	-1.7984	0.87707	0.99929	0.80964	-0.3046	0.03653	0.095171	3.9231
Bonemar	9.87	792.5803	M+H	7	791.5676348	PS(16:0/20:0)	C42H82NO10P		HMDB011	LMGP0301	40820	9547095	0.26589	0.99994	0.91079	-0.1348	0.77656	0.99946	0.97862	-0.0312	0.25066	0.29725	1.1206	0.1643	0.07849	0.094545	0.85677	-0.223	0.24329	0.9998	0.88755	-0.1721	0.95181	0.95965	0.99459	-0.0078	0.51276	0.99932	1.0745	0.1036	0.30456	0.35368	1.2588	0.76486	0.99919	0.96464	-0.0519	0.22516	0.90043	0.87297	-0.196	0.013656	0.035231	1.3432	0.4257	0.092705	0.12168	0.77093	-0.3753	0.15433	0.72814	0.73227	-0.4496	0.80509	0.86746	0.98357	-0.0239	0.36794	0.99929	0.90497	-0.1441	0.10198	0.16897	2.5647
Bonemar	7.37	329.2674	M+H	4	328.2613596	MG(0:0/16:1(9Z)/0	C19H36O4		HMDB0011534		62318	53480960	0.080413	0.99994	0.53846	-0.8931	0.42368	0.99946	1.2139	0.2796	0.035251	0.065071	1.7271	0.7884	0.083084	0.099591	1.5348	0.6181	0.03756	0.9998	0.62365	-0.6812	0.87112	0.894	1.0771	0.1072	0.0033699	0.28323	2.2543	1.1727	0.064651	0.096083	3.1291	0.049546	0.75393	1.7572	0.8133	0.65326	0.90043	1.2404	0.3108	0.016031	0.038743	1.7543	0.8109	0.61686	0.6671	0.82281	-0.2814	0.10497	0.72814	0.59028	-0.7605	0.7333	0.81877	1.0355	0.0504	0.16379	0.99929	0.70588	-0.5025	0.092087	0.15882	2.6935
Bonemar	7.99	361.1811	M+H	5	360.17573	Arg Asp Ala	C13H24N6O6				18542		0.26067	0.99994	0.84843	-0.2371	0.40184	0.99946	1.1268	0.1723	0.67837	0.71805	1.0337	0.0478	0.068195	0.082753	1.2597	0.333	0.55703	0.9998	0.93893	-0.0909	0.8091	0.84242	0.97056	-0.0431	0.040573	0.99932	1.3281	0.4094	0.81077	0.82737	0.21188	0.058855	0.79022	1.2713	0.3464	0.4665	0.90043	0.88781	-0.1717	0.15105	0.19378	0.79878	-0.3241	0.0056615	0.0091494	2.7857	1.478	0.15819	0.72814	0.5801	-0.7856	0.033985	0.10225	0.46338	-1.1097	0.0032352	0.38365	0.69832	-0.518	0.04412	0.10508	3.6627
Bonemar	11.98	842.531	M+Na	5	819.541423	PS(P-18:0/22:6(4Z,7	C46H78NO9P			LMGP030	78829		0.23708	0.99994	1.1017	0.1397	0.20702	0.99946	1.1271	0.1726	0.15102	0.1908	1.3532	0.4364	0.048342	0.059737	0.77473	-0.3682	0.37727	0.9998	0.90192	-0.1489	0.37	0.42628	1.2205	0.2875	0.80203	0.99932	1.0231	0.0329	0.25614	0.30448	1.4543	0.063736	0.79705	0.79287	-0.3348	0.73305	0.91505	0.96294	-0.0545	0.13376	0.17585	1.453	0.539	0.015092	0.022429	0.65734	-0.6053	0.15115	0.72814	0.72202	-0.4699	0.89749	0.92821	1.0491	0.0691	0.06986	0.87279	1.2145	0.2804	0.26944	0.34054	1.4013
Bonemar	0.33	957.7906	M+H	0	956.7832911	TG(22:4(7Z,10Z,13	C63H104O6		HMDB0054712			131765279	0.91666	0.99994	0.98821	-0.0171	0.68701	0.99946	1.0709	0.0988	2.49E-01	0.29508	1.1572	0.2107	5.48E-02	6.71E-02	1.3035	0.3824	0.60483	0.9998	0.94547	-0.0809	4.92E-01	0.54616	1.0941	0.1298	0.6188	0.99932	1.0836	0.1159	0.4792	0.52003	0.76202	0.69624	0.99919	1.0996	0.137	0.82415	0.94674	0.96093	-0.0575	9.17E-01	9.30E-01	1.0767	0.1066	0.031326	0.044207	1.8049	0.8519	0.25935	0.72814	0.71936	-0.4752	0.36386	0.49499	0.77455	-0.3686	0.56474	0.99929	0.87387	-0.1945	0.46635	0.51386	0.79267
Jejunum	0.42	243.0634	M-H	5	244.0695361	Uridine	C9H12N2	C00299	HMDB0000296		90	6029	4.797E-06	0.017183	0.65919	-0.6012	0.031473	0.51202	0.8226	-0.2817	6.90E-01	0.88108	0.94785	-0.0773	1.07E-01	2.06E-01	0.84661	-0.2402	0.10124	0.6012	1.2663	0.3406	1.65E-01	0.5821	1.2003	0.2634	0.064599	0.99981	1.2479	0.3195	0.15341	0.55452	2.0524	0.10723	0.64069	0.73175	-0.4506	0.10202	0.54913	0.74414	-0.4264	3.26E-01	9.90E-01	0.88439	-0.1773	3.64E-01	1.00E+00	1.1249	0.1698	0.16327	0.4946	0.83329	-0.2631	1.32E-02	4.83E-01	0.73695	-0.4404	0.81981	0.94912	1.0169	0.0242	0.048932	0.81161	3.4955
Jejunum	0.43	283.0682	M+Cl	8	248.1008362	gamma-Glutamylthr	C9H16N2O6		HMDB0029159			53861142	0.0007199	0.11542	0.32159	-1.6367	0.11329	0.51203	0.83045	-0.268	3.86E-02	0.81952	2.7982	1.4845	5.65E-09	2.02E-05	0.15066	-2.7307	0.44688	0.9428	1.4929	0.5781	6.38E-03	0.11035	4.1773	2.0626	0.2487	0.99981	2.5823	1.3687	0.0075748	0.15329	6.2168	0.24883	0.67086	1.271	0.346	0.91586	0.96969	1.1582	0.2119	2.41E-01	0.99041	1.4198	0.5057	0.60037	0.99953	0.91074	-0.1349	0.56369	0.79454	0.82528	-0.277	0.559	0.80221	1.1717	0.2287	0.31421	0.78807	0.91125	-0.1341	0.49829	0.84074	0.72019
Jejunum	7.98	593.3981	M+Cl	0	558.428425	DG(14:1(9Z)/18:4(6	C35H58O5		HMDB0007048		58683	53477983	0.2189	0.4086	0.7703	-0.3765	0.19046	0.53508	0.76189	-0.3923	3.34E-02	0.81952	1.639	0.7128	1.16E-07	1.39E-04	0.17714	-2.497	0.95216	0.99865	1.0577	0.0809	1.30E-02	0.1577	1.7336	0.7938	0.80475	0.99981	0.98908	-0.0158	0.020961	0.24143	4.6724	0.07141	0.64069	0.7584	-0.399	0.20657	0.57502	0.82031	-0.2858	6.54E-01	9.90E-01	1.0541	0.0761	3.24E-01	1.00E+00	0.91161	-0.1335	0.74119	0.88887	1.094	0.1296	3.85E-01	0.72571	1.1532	0.2056	0.91536	0.97925	1.0816	0.1132	0.71794	0.89248	0.33666
Jejunum	10.31	479.2922	M+Cl	3	444.3239599	MG(0:0/24:6(6Z,9Z	C28H44O4		HMDB0011560			131801685	0.72497	0.83178	1.1875	0.2479	0.22709	0.56528	0.78698	-0.3456	7.77E-04	0.46402	3.5011	1.8078	1.25E-06	6.34E-04	0.15571	-2.6831	0.68676	0.99373	1.6047	0.6823	5.91E-07	0.0021178	5.618	2.4901	0.49529	0.99981	0.66271	-0.5935	3.468E-05	0.01259	17.422	0.14708	0.65447	0.75153	-0.4121	0.0002746	0.1967	0.42924	-1.2201	6.70E-01	9.90E-01	0.89789	-0.1554	4.44E-01	1.00E+00	1.1177	0.1605	0.61891	0.82691	0.90475	-0.1444	8.33E-01	9.29E-01	0.81237	-0.2998	0.099435	0.78738	0.57115	-0.8081	0.85488	0.93056	0.15797
Jejunum	11.35	479.3191	M-H	10	480.3215909	LysoPA(i-21:0/0:0)	C24H49O7P		HMDB0114768			17786805	0.85817	0.9198	0.94265	-0.0852	0.73447	0.87658	0.88864	-0.1703	4.85E-02	0.81952	1.7032	0.7682	1.34E-06	6.34E-04	0.11397	-3.1333	0.18957	0.69585	1.6164	0.6928	2.10E-04	0.014171	2.7529	1.461	0.88082	0.99981	0.9427	-0.0851	0.0031693	0.11352	7.6632	0.30195	0.70516	0.8552	-0.2257	0.32979	0.66665	0.85564	-0.2249	1.83E-01	9.90E-01	1.3701	0.4543	1.24E-01	1.00E+00	0.75855	-0.3987	0.92141	0.9735	0.94869	-0.076	2.34E-01	0.69032	1.2999	0.3784	0.8575	0.96129	1.0005	0.0007	0.35553	0.81398	1.0868
Jejunum	7.72	579.3876	M-H	6	580.391646	Phoenicoxanthin/ A	C40H52O3	C15967		LMPR0102	41354	16061231	0.013799	0.14482	0.58868	-0.7644	0.04334	0.51202	0.64771	-0.6266	1.67E-02	0.81952	1.6015	0.6794	1.42E-06	6.34E-04	0.24308	-2.0405	0.057549	0.57292	1.6031	0.6809	6.11E-06	0.0069478	2.5674	1.3603	0.5566	0.99981	1.1003	0.1379	0.0001331	0.025092	14.065	0.013666	0.49677	0.6441	-0.6347	0.015385	0.52483	0.65755	-0.6048	3.38E-01	9.90E-01	1.135	0.1826	1.90E-02	7.33E-01	1.853	0.8899	0.52906	0.77005	0.91877	-0.1222	6.92E-01	8.67E-01	1.0428	0.0604	0.85608	0.96089	1.0209	0.0298	0.57164	0.85993	0.57441
Jejunum	9.25	591.3911	M-H	1	592.397521	Collettiside I	C34H56O8			LMST0108	84243		0.0002137	0.058887	1.8775	0.9088	0.064468	0.51202	1.3597	0.4433	1.85E-02	0.81952	1.5952	0.6737	1.69E-06	6.74E-04	0.33928	-1.5595	0.54836	0.99373	1.2182	0.2848	9.54E-04	0.037684	1.9433	0.9585	0.047999	0.99981	0.7242	-0.4656	0.0051842	0.13391	6.8316	0.0041625	0.49677	1.6402	0.7139	0.26622	0.60933	1.3131	0.393	1.43E-01	9.90E-01	0.74503	-0.4246	6.27E-03	4.50E-01	0.58438	-0.775	0.9827	0.99661	1.0128	0.0183	1.26E-01	6.90E-01	0.75454	-0.4063	0.17445	0.78738	0.8006	-0.3208	0.23475	0.81161	1.554
Jejunum	9.5	620.4236	M-H	10	621.4369545	PC(10:0/14:0)[U]	C32H64NO8P				39128		0.9335	0.96221	1.0784	0.1088	0.61588	0.80309	0.92098	-0.1188	8.78E-02	0.81952	1.613	0.6898	2.42E-06	7.11E-04	0.11565	-3.1122	0.41677	0.91993	1.2093	0.2741	1.94E-02	0.19765	1.9506	0.9639	0.58897	0.99981	0.85404	-0.2276	0.042197	0.33966	3.6943	0.3319	0.72758	0.85303	-0.2293	0.29514	0.63878	0.80701	-0.3094	9.05E-01	0.99041	0.91736	-0.1244	4.94E-03	3.69E-01	0.44281	-1.1752	0.41348	0.69339	1.4743	0.5601	2.83E-01	0.7032	1.3525	0.4356	0.76116	0.93819	0.94605	-0.08	0.54221	0.8482	0.6303
Jejunum	11.45	493.3335	M-H	7	494.337241	LysoPA(22:0/0:0)	C25H51O7P		HMDB0114749			17786745	0.7453	0.84805	0.92241	-0.1165	0.7988	0.91066	0.90674	-0.1412	6.06E-02	0.81952	1.5648	0.646	2.826E-06	0.0007108	0.13751	-2.8624	0.1301	0.63715	1.5647	0.6459	0.0002647	0.015853	2.4484	1.2918	0.91705	0.99981	0.98301	-0.0247	0.0028421	0.11311	7.8527	0.35436	0.74862	0.8875	-0.1722	0.31142	0.65209	0.86647	-0.2068	0.16525	0.99041	1.3813	0.466	0.22738	0.99953	0.83394	-0.262	0.9434	0.98607	0.99966	-0.0005	0.15477	0.69032	1.3808	0.4655	0.99914	0.99942	0.9763	-0.0346	0.26875	0.81161	1.3997
Jejunum	7.79	522.3544	M-H	4	523.3637704	enantio-PAF C-16	C26H54NO7P				43413		0.40243	0.56748	1.1981	0.2607	0.16905	0.52276	1.3557	0.4391	1.42E-02	0.81952	1.6202	0.6962	5.32E-06	9.07E-04	0.39627	-1.3354	0.036316	0.50031	1.4732	0.559	3.97E-05	0.010454	2.3869	1.2551	0.57587	0.99981	1.1316	0.1783	0.0001133	0.02387	14.445	0.32328	0.7196	0.79475	-0.3314	0.038958	0.54913	0.57706	-0.7932	5.81E-01	9.90E-01	0.76769	-0.3814	0.15714	0.99953	0.68202	-0.5521	0.36833	0.65935	1.4256	0.5116	6.35E-01	0.83659	1.0944	0.1302	0.52773	0.85527	0.72609	-0.4618	0.60742	0.87158	0.51055
Jejunum	8.37	492.3449	M-H	2	493.353227	PC(P-17:0/0:0)	C25H52NO6P			LMGP010	40403		0.088888	0.29229	1.8428	0.8819	0.26441	0.58364	1.8389	0.8789	1.02E-01	0.81952	0.64248	-0.6383	8.40E-06	1.16E-03	4.1891	2.0666	0.65642	0.99373	0.95646	-0.0642	2.53E-02	0.22732	0.61451	-0.7025	0.74518	0.99981	0.99791	-0.003	0.062217	0.40594	3.179	0.90816	0.9986	0.8588	-0.2196	0.49916	0.77074	0.60567	-0.7234	3.36E-01	9.90E-01	0.57649	-0.7946	2.16E-01	1.00E+00	1.8629	0.8975	0.79254	0.91874	0.89447	-0.1609	1.96E-01	0.69032	0.51565	-0.9555	0.26768	0.78738	0.70525	-0.5038	0.41728	0.82914	0.91141

[illegible]

													1. Metabolites affected by drug only Day 4 ami_50/Vehicle				1. Metabolites affected by drug only Day 4 ami_200/Vehicle				2. Dose response Day 4 Rad + ami_200/Rad + a[illegible]			[illegible]
Type	RT	[illegible]erimental.[illegible]	Adduct	PPM.Error	[illegible]olecular.Weig[illegible]	Name	Formula	Kegg	HMDB	LipidMaps	Metlin	PubChem	p.value	FDR	[illegible]old.Chang[illegible]	log2.FC	p.value	FDR	[illegible]old.Chang[illegible]	log2.FC	p.value	FDR	[illegible]old.Chan[illegible]	[illegible]
Jejunum	9.99	744.5575	M+H	5	743.546505	PE(18:1(11Z)/18:1([illegible]	C41H78NO8P		HMDB0009026		60444	53479625	0.88147	0.94735	1.0412	0.0582	0.36079	0.84632	1.1219	0.166	1.37E-01	0.99884	1.224[illegible]	[illegible]
Jejunum	9.94	772.5875	M+H	3	771.5778051	PE(16:1(9Z)/22:1(1[illegible]	C43H82NO8P		HMDB0008974		60403	53479600	0.40887	0.67733	0.91424	-0.1294	0.85717	0.98818	0.98294	-0.0248	4.61E-02	0.9055	1.336[illegible]	[illegible]
Jejunum	7.78	538.3866	M+H	0	537.3794397	PC(O-16:0/3:0)[U]	C27H56NO7P				40096		0.1541	0.49717	1.434	0.5201	0.036843	0.734	1.6051	0.6826	6.36E-03	0.54706	1.859[illegible]	[illegible]
Jejunum	9.95	716.5256	M+H	4	715.5152049	PE(20:1(11Z)/14:1([illegible]	C39H74NO8P		HMDB000[illegible]	LMGP0201[illegible]	60647	52924545	0.83882	0.92733	1.1891	0.2499	0.027711	0.734	1.3526	0.4357	1.15E-01	0.99884	1.203[illegible]	[illegible]
Jejunum	8.36	411.3622	M+H	0	410.354866	Corbisterol	C29H46O			LMST0104[illegible]	84139		0.76324	0.87857	0.92383	-0.1143	0.95309	0.99426	0.86317	-0.2123	8.91E-02	0.99884	1.280[illegible]	[illegible]
Jejunum	10.55	889.5061	M+Na	8	866.5097856	PG(22:6(4Z,7Z,10Z,[illegible]	C50H75O10P		HMDB011[illegible]	LMGP0401[illegible]	79802	52927240	0.014407	0.30345	0.66398	-0.5908	0.01177	0.734	0.71003	-0.4941	1.31E-02	0.68048	1.235[illegible]	[illegible]
Jejunum	11.28	644.5241	M+Na	4	621.537551	DG(18:2(9Z,12Z)/0:[illegible]	C39H63D5O5			LMGL0201[illegible]	4695		0.50192	0.73002	0.87367	-0.1948	0.81342	0.98102	0.93415	-0.0983	1.24E-01	0.99884	1.275[illegible]	[illegible]
Jejunum	9.69	597.4822	M+Na	5	574.496111	Mayolene-19	C37H66O4			LMFA07010690			0.9779	0.99207	0.99656	-0.005	0.48113	0.87002	1.0713	0.0993	4.84E-01	0.99884	1.075[illegible]	[illegible]
Jejunum	11.88	738.5062	M+H	1	737.4995548	PE(22:5(7Z,10Z,13Z,[illegible]	C41H72NO8P		HMDB0009646		61035	53479942	0.67439	0.83819	1.2112	0.2765	0.010633	0.734	1.3951	0.4804	1.63E-01	0.99884	1.150[illegible]	[illegible]
Jejunum	7.99	305.2481	M+H	2	304.2402	ent-2alpha,3alpha-[illegible]	C20H32O2	C21831					0.15554	0.49822	1.414	0.4998	0.371	0.85045	1.298	0.3763	1.68E-01	0.99884	0.7891	[illegible]
Jejunum	9.8	689.5587	M+H	1	688.551926	SM(d16:1/17:0)	C38H77N2O6P			LMSP0301[illegible]	83739		0.53729	0.75164	1.2712	0.3462	0.046331	0.734	1.3015	0.3802	1.03E-01	0.99884	1.286[illegible]	[illegible]
Jejunum	9.71	739.5199	M+H	10	738.519959	PA(22:2(13Z,16Z)/1[illegible]	C42H75O8P			LMGP1001[illegible]	81931		0.27103	0.58464	0.87165	-0.1982	0.62295	0.91627	0.94369	-0.0836	1.54E-01	0.99884	1.182[illegible]	[illegible]
Jejunum	10.54	904.621	M+Na	4	881.635718	PI-Cer(d20:0/20:0([illegible]	C46H92NO12P			LMSP0303[illegible]	103179		0.14519	0.49344	0.78489	-0.3494	0.085082	0.734	0.75879	-0.3982	3.55E-01	0.99884	1.169[illegible]	[illegible]
Jejunum	8.52	584.3277	M+Na	8	561.3430542	PC(18:2(9Z,12Z)/2:[illegible]	C28H52NO8P				39681		0.052458	0.39572	1.4993	0.5842	0.0073633	0.734	1.6943	0.7607	5.08E-02	0.94734	0.6583	[illegible]

[illegible]

1. Metabolites affected by drug only — Day 4 — ami_50/Vehicle …

Type	RT	erimental.r	Adduct	PPM.Error	Molecular.Weig	Name	Formula	Kegg	HMDB	LipidMaps	Metlin	PubChem	p.value	(further columns)
Lung PO	10.11	892.6854	M+H	7	891.6717055	PC(20:5(5Z,8Z,11Z,	C52H94NO8P		HMDB0008519		59965	53479135	0.30937	[illegible]
Lung PO	8.78	464.2328	M+H	0	463.22533	Trp Lys Met	C22H33N5O4S				15642		0.048537	[illegible]
Lung PO	6.65	297.1459	M+Na	1	274.1568946	Panaquinquecol 4	C17H22O3		HMDB0038939		93550	10468587	0.69972	[illegible]
Lung PO	9.94	771.5875	M+H	3	770.582559	PA(22:2(13Z,16Z)/1	C44H83O8P			LMGP1001	81938		0.79619	[illegible]
Lung PO	10.62	985.669	M+Na	3	962.682334	PI(21:0/22:1(11Z))	C52H99O13P			LMGP0601	80690		0.24897	[illegible]
Lung PO	8.94	606.4492	M+H	0	605.4420399	LysoPC(24:1(15Z))	C32H64NO7P		HMDB001	LMGP0105	61715	53480477	0.86412	[illegible]
Lung PO	9.9	706.5386	M+H	1	705.5308549	PC(12:0/18:0)[U]	C38H76NO8P				39181		0.39204	[illegible]
Lung PO	7.02	510.3554	M+H	0	509.3481395	PC(O-15:0/2:0)[U]	C25H52NO7P				40054		0.74404	[illegible]
Lung PO	0.37	533.9654	M+Na	1	510.97565	Pyridoxalphosphate	C14H14N3	C13745			69677		0.48828	[illegible]
Lung PO	10.57	983.6584	M+Na	3	960.666684	PI(21:0/22:2(13Z,1	C52H97O13P			LMGP0601	80691		0.95497	[illegible]
Lung PO	0.41	364.0657	M+H	1	363.057999	Cyclic pyranopterin	C10H14N5	C18239	HMDB0059639		72085	16061579	0.3355	[illegible]
Lung PO	0.41	308.0911	M+H	0	307.083806	Glutathione	C10H17N3	C00051	HMDB0000125		44	25246407	0.074399	[illegible]
Lung PO	12.02	784.5071	M+H	7	783.50503	PS(18:2(9Z,12Z)/18	C42H74NO10P				40801		0.15344	[illegible]
Lung PO	12	830.4947	M+H	2	829.489384	PS(22:6(4Z,7Z,10Z,	C46H72NO10P		HMDB001	LMGP0301	78495	52925941	0.47062	[illegible]
Lung PO	11.94	947.5076	M+Na	6	924.512902	CL(1'-[18:2(9Z,12Z)	C45H82O15P2			LMGP1201	82366		0.0065008	[illegible]
Lung PO	9.94	798.6029	M+H	3	797.5934552	PC(18:0/19:3(9Z,12	C45H84NO8P				39519		0.96296	[illegible]
Lung PO	8.24	578.4184	M+H	1	577.4107398	LysoPC(22:1(13Z))	C30H60NO7P		HMDB001	LMGP0105	61708	53480471	0.35595	[illegible]
Lung PO	9.98	797.605	M+Na	2	774.6138568	PA(20:0/21:0)	C44H87O8P		HMDB011	LMGP1001	81688	52929115	0.82697	[illegible]
Lung PO	9.91	823.6141	M+Na	6	800.629509	PA(21:0/22:1(11Z))	C46H89O8P			LMGP1001	81864		0.9196	[illegible]
Lung PO	12	806.492	M+H	6	805.489384	PS(16:1(9Z)/22:6(4	C44H72NO10P		HMDB001	LMGP0301	77708	52925154	0.50863	[illegible]
Lung PO	9.93	836.6137	M+H	5	835.602095	LacCer(d18:0/14:0)	C44H85NO13			LMSP0501	83817		0.92954	[illegible]
Lung PO	6.65	298.1397	M+Na	9	275.14812	Lysyl-Glutamate	C11H21N3O5				85852		0.45607	[illegible]
Lung PO	10.65	958.6512	M+Na	0	935.6615347	PE(DiMe(13,5)/DiM	C53H94NO10P		HMDB0061491			131770163	0.059567	[illegible]
Lung PO	0.4	310.0881	M+H	3	309.0817	Met Cys Gly	C10H19N3O4S2				16638		0.072306	[illegible]
Lung PO	9.65	698.5035	M+Na	9	675.5202902	PE(P-16:0/16:0)	C37H74NO7P		HMDB001	LMGP0203	77591	71296148	0.14059	[illegible]
Lung PO	8	464.3824	M+Na	9	441.39707	Oleic Acid-2,6-diisor	C30H51NO				64645		0.12948	[illegible]
Lung PO	0.37	206.0789	M+Na	1	183.0895	L-Normetanephrine	C9H13NO3	C05589				688100	0.11308	[illegible]
Lung PO	0.34	399.1258	M+H	6	398.1161092	Methylacrylate-divi	C18H18N6O3S		HMDB0032389		88413	1151711	0.91839	[illegible]
Lung PO	10.16	871.7075	M+H	9	870.7077571	PA(24:1(15Z)/24:0)	C51H99O8P		HMDB0115480			131822217	0.014437	[illegible]
Lung PO	10.16	870.7004	M+H	7	869.6873556	PC(20:1(11Z)/22:1(	C50H96NO8P		HMDB000	LMGP0101	59762	53478901	0.045299	[illegible]
Lung PO	5.92	500.2784	M+H	2	499.2698892	PE(20:5(5Z,8Z,11Z,	C25H42NO7P		HMDB001	LMGP0205	77693	52925146	0.056946	[illegible]
Lung PO	12.01	785.5105	M+Na	2	762.519959	PA(22:6(4Z,7Z,10Z,	C44H75O8P			LMGP1001	81998		0.23262	[illegible]
Lung PO	11.99	831.4978	M+H	5	830.494534	PI(16:1(9Z)/18:3(6Z	C43H75O13P			LMGP0601	80202		0.1714	[illegible]
Lung PO	11.56	960.6391	M+Na	1	937.649045	FMC-5(d18:1/18:0)	C52H91NO13			LMSP0501	83797		0.0061711	[illegible]
Lung PO	12.03	808.5034	M+Na	8	785.52068	PS(18:1(9Z)/18:2(9	C42H76NO10P				40803		0.45484	[illegible]
Lung PO	11.76	599.5036	M+Na	5	576.5117607	Cohibin D	C37H68O4		HMDB0035398		90692	131751738	0.091021	[illegible]
Lung PO	9.85	819.6037	M+H	9	818.603689	PG(21:0/18:1(9Z))	C45H87O10P			LMGP0401	79510		0.45773	[illegible]
Lung PO	9.69	856.5808	M+H	5	855.5778051	PC(22:5(4Z,7Z,10Z,	C50H82NO8P		HMDB0008674		60120	53479311	0.29189	[illegible]
Lung PO	6.63	275.1341	M+H	3	274.1277197	Glutaminylglutamine	C10H18N4O5		HMDB0028795		85709	7010588	0.82248	[illegible]
Lung PO	9.94	769.5768	M+H	3	768.5669066	PA(22:1(13Z)/19:2(	C44H81O8P		HMDB0115591			131822251	0.97535	[illegible]
Lung PO	0.41	630.0668	M+Na	7	607.0815695	Uridine diphosphate	C17H27N3O17P2		HMDB0000304		3675	1167	0.98514	[illegible]
Lung PO	9.7	713.5075	M+H	6	712.504309	PA(17:1(9Z)/20:2(1	C40H73O8P			LMGP1001	81432		0.57679	[illegible]
Lung PO	9.85	818.5988	M+H	9	817.5985406	PC(22:5(4Z,7Z,10Z,	C48H84NO7P		HMDB0008687		60133	53479337	0.28348	[illegible]
Lung PO	11.91	935.5562	M+H	9	934.557134	PI(22:4(7Z,10Z,13Z,	C51H83O13P			LMGP0601	80801		0.013444	[illegible]
Lung PO	10.62	945.6468	M+H	4	944.635384	PI(22:0/20:3(8Z,11Z	C51H93O13P			LMGP0601	80709		0.22724	[illegible]
Lung PO	9.89	820.6013	M+H	6	819.598935	PS(20:0/18:0)	C44H86NO10P		HMDB011	LMGP0301	78616	52926059	0.93627	[illegible]
Lung PO	10.5	953.693	M+Na	3	930.700747	DAT(16:0/22:0(2Me	C52H98O13			LMSL0300	82433		0.0083836	[illegible]
Lung PO	9.79	705.5387	M+H	6	704.5356064	PA(i-12:0/i-24:0)	C39H77O8P		HMDB0115792			131822448	0.85771	[illegible]
Lung PO	9.78	858.5955	M+H	6	857.5934552	PC(20:3(8Z,11Z,14Z	C50H84NO8P		HMDB0008417		59863	53479017	0.1286	[illegible]
Lung PO	9.89	773.5744	M+H	7	772.561824	PG(P-20:0/17:2(9Z,	C43H81O9P			LMGP0403	79970		0.23842	[illegible]
Lung PO	9.98	745.5799	M+H	8	744.5669066	PA(24:1(15Z)/15:0)	C42H81O8P		HMDB0115457			131822195	0.15836	[illegible]
Lung PO	10.14	894.7006	M+H	7	893.6873556	PC(20:4(8Z,11Z,14Z	C52H96NO8P		HMDB0008486		59932	53479111	0.49909	[illegible]
Lung PO	12.1	893.5262	M+H	10	892.510184	PI(17:2(9Z,12Z)/22:	C48H77O13P			LMGP0601	80297		0.010717	[illegible]
Lung PO	9.71	697.5097	M+Na	7	674.525044	PA(O-20:0/15:1(9Z)	C38H75O7P			LMGP1002	82193		0.0068528	[illegible]
Lung PO	9.88	792.5757	M+H	1	791.5676348	PS(16:0/20:0)	C42H82NO10P		HMDB011	LMGP0301	40820	9547095	0.94325	[illegible]
Lung PO	11.93	887.5586	M+H	7	886.5571293	PI(16:0/22:4(7Z,10Z	C47H83O13P		HMDB000	LMGP0601	61179		0.001935	[illegible]
Lung PO	10.7	923.6517	M+Na	4	900.658442	PGP-Me	C47H98O11P2			LMGP0504	80032		0.06752	[illegible]
Lung PO	8.98	673.5131	M+H	5	672.509394	PA(P-16:0/19:1(9Z))	C38H73O7P			LMGP1003	82257		0.26675	[illegible]
Lung PO	9.99	747.5883	M+Na	2	724.6005757	DG(22:5(4Z,7Z,10Z,	C47H80O5		HMDB0007725		59188	53478478	0.10138	[illegible]
Lung PO	9.72	882.5991	M+H	2	881.5934552	PC(22:5(7Z,10Z,13Z	C52H84NO8P		HMDB0008713		60159	53479389	0.48783	[illegible]
Lung PO	11.94	881.5139	M+H	4	880.510184	PI(18:4(6Z,9Z,12Z,1	C47H77O13P			LMGP0601	80437		0.096179	[illegible]
Lung PO	9.87	779.5613	M+H	4	778.5512565	PA(22:5(4Z,7Z,10Z,	C45H79O8P		HMDB0115361			131822118	0.92127	[illegible]
Lung PO	9.71	675.5411	M+H	4	674.5362747	SM(d18:1/14:0)	C37H75N2O6P		HMDB001	LMSP0301	53976	11433862	0.017512	[illegible]
Lung PO	10.61	931.6356	M+H	9	930.619734	PI(19:1(9Z)/22:2(13	C50H91O13P			LMGP0601	80498		0.133	[illegible]
Lung PO	9.77	739.5267	M+H	1	738.519959	PA(22:2(13Z,16Z)/1	C42H75O8P			LMGP1001	81931		0.41991	[illegible]
Lung PO	11.98	807.4952	M+H	8	806.494529	PI(16:2(9Z,12Z)/16:	C41H75O13P		HMDB0009802		61187		0.70107	[illegible]
Lung PO	10	838.6301	M+H	2	837.6247553	PC(20:3(5Z,8Z,11Z)	C48H88NO8P		HMDB000	LMGP0101	59820	53478969	0.71614	[illegible]
Lung PO	11.08	591.5294	M+H	9	590.52741	Coixenolide	C38H70O4	C17566			71781	46173943	0.011353	[illegible]
Lung PO	9.74	802.5383	M+H	0	801.5308549	PC(20:4(8Z,11Z,14Z	C46H76NO8P		HMDB0008470		59916	53479079	0.56801	[illegible]
Lung PO	10.62	944.6414	M+Na	7	921.6458847	PE(DiMe(13,5)/Mor	C52H92NO10P		HMDB0061496			131770168	0.072943	[illegible]
Lung PO	10.19	966.7779	M+Na	9	943.796906	PC(21:0/26:0)[U]	C55H110NO8P				39819		0.0047521	[illegible]
Lung PO	0.35	398.018	M+Na	5	375.0308099	omega-COOH-tetrar	C15H16Cl3	C11182	HMDB0062513		68898	73665	0.99935	[illegible]
Lung PO	6.66	515.3093	M+Na	3	492.3215909	PA(22:1(11Z)/0:0)	C25H49O7P		HMDB006	LMGP1005	82348	52929762	0.18541	[illegible]
Lung PO	10.7	937.667	M+H	7	936.666684	PI(22:0/19:0)	C50H97O13P			LMGP0601	80705		0.030641	[illegible]
Lung PO	9.61	880.5822	M+H	3	879.5778051	PC(22:6(4Z,7Z,10Z,	C52H82NO8P		HMDB0008747		60193	53479417	0.82613	[illegible]
Lung PO	12.02	874.4893	M+Na	6	851.4948633	PS(DiMe(11,3)/Mor	C45H74NO12P		HMDB0061557			131770234	0.30704	[illegible]
Lung PO	11.91	911.5548	M+Na	8	888.5727793	PI(18:0/20:3(5Z,8Z,	C47H85O13P		HMDB0009813		61198		0.0048418	[illegible]
Lung PO	6.66	498.3458	M+H	7	497.33525	Tumonoic Acid I	C27H47NO7				65437		0.1081	[illegible]
Lung PO	11.9	909.5473	M+H	2	908.541484	PI(22:4(7Z,10Z,13Z,	C49H81O13P			LMGP0601	80792		0.0037831	[illegible]
Lung PO	6.66	997.6744	M+Na	3	974.682334	PI(22:0/22:2(13Z,16	C53H99O13P			LMGP0601	80714		0.075271	[illegible]
Lung PO	9.93	778.5582	M+H	1	777.5519848	PS(15:0/20:0)	C41H80NO10P		HMDB011	LMGP0301	77814	52925260	0.93367	[illegible]
Lung PO	9.63	677.5242	M+H	2	676.515541	PE-Cer(d14:1(4E)/2	C36H73N2O7P			LMSP0302	103099		0.096241	[illegible]
Lung PO	6.69	524.2988	M+H	1	523.29102	PS(18:1(9Z)/0:0)[U]	C24H46NO9P				40832		0.62897	[illegible]
Lung PO	11.91	955.5231	M+Na	8	932.541484	PI(22:4(7Z,10Z,13Z,	C51H81O13P			LMGP0601	80802		0.30176	[illegible]
Lung PO	0.41	179.0498	M+H	7	178.04121	Cysteinyl-Glycine	C5H10N2O3S				85689		0.025582	[illegible]
Lung PO	8.77	387.2736	M+H	6	386.26415	Arg Leu Val	C17H34N6O4				15691		0.0095427	[illegible]
Lung PO	6.66	496.3398	M+H	0	495.3324895	PC(O-14:0/2:0)[U]	C24H50NO7P				40049		0.13655	[illegible]
Lung PO	11.95	882.5215	M+H	7	881.5206846	PS(22:6(4Z,7Z,10Z,	C50H76NO10P		HMDB0112878			131820000	0.098002	[illegible]
Lung PO	0.4	615.1593	M+H	8	614.1472675	Cytidine monophos	C20H31N4	C00128	HMDB0001176		434	448209	0.73245	[illegible]
Lung PO	10.85	926.674	M+H	5	925.661933	PI-Cer(t20:0/22:0(2	C48H96NO13P			LMSP0303	103174		0.013092	[illegible]
Lung PO	11.93	903.4973	M+H	5	902.494534	PI(18:4(6Z,9Z,12Z,1	C49H75O13P			LMGP0601	80445		0.70642	[illegible]
Lung PO	0.36	543.1152	M+Na	8	520.121696	Isorhamnetin 3-(6"-	C24H24O13			LMPK1211	50750		0.40464	[illegible]
Lung PO	9.76	780.555	M+Na	5	757.5621551	PC(18:2(9Z,12Z)/16	C42H80NO8P		HMDB000	LMGP0101	59579	24778972	0.78248	[illegible]
Lung PO	9.74	711.5066	M+Na	3	688.5155392	SM(d18:0/14:1(9Z)	C37H73N2O7P		HMDB0013462			53481777	0.78065	[illegible]
Lung PO	6.66	518.3219	M+H	4	517.3168394	PC(18:3(6Z,9Z,12Z)	C26H48NO7P				40303		0.10072	[illegible]
Lung PO	8.77	267.2681	M+H	1	266.26097	octadecan-3Z,13Z-d	C18H34O				75270		0.049353	[illegible]
Lung PO	10.65	986.6775	M+Na	5	963.6928354	PE-NMe2(13D5/13D	C55H98NO10P		HMDB0114700			131821807	0.058093	[illegible]
Lung PO	11.95	883.5262	M+H	8	882.525834	PI(18:4(6Z,9Z,12Z,1	C47H79O13P			LMGP0601	80436		0.039725	[illegible]
Lung PO	9.1	384.2178	M+H	8	383.207289	PC(O-6:0/2:0)[U]	C16H34NO7P				40153		0.020564	[illegible]
Lung PO	6.27	482.3233	M+H	2	481.3168394	PC(15:0/0:0)[S]	C23H48NO7P				40283		0.79274	[illegible]
Lung PO	11.91	910.5511	M+Na	6	887.5676348	PS(22:2(13Z,16Z)/2	C50H82NO10P		HMDB011	LMGP0301	78450	52925896	0.0060108	[illegible]
Lung PO	12.09	943.4857	M+H	4	942.4824302	Phytolaccoside I	C47H74O19		HMDB0040810		95273	131752946	0.060485	[illegible]
Lung PO	11.91	933.5391	M+Na	8	910.5571293	PI(18:3(9Z,12Z,15Z)	C49H83O13P		HMDB0009865		61250		0.042799	[illegible]
Lung PO	0.4	613.1603	M+H	2	612.1519619	Oxidized glutathione	C20H32N6O12S2		HMDB0003337		45	5255796	0.84537	[illegible]
Lung PO	6.85	482.3602	M+H	1	481.3532062	Lyso-PAF C-16	C24H52NO6P				24070		0.084647	[illegible]
Lung PO	9.83	764.5383	M+H	7	763.5363347	PS(20:0/14:0)	C40H78NO10P		HMDB011	LMGP0301	78529	52925974	0.12793	[illegible]
Lung PO	9.83	765.5371	M+H	8	764.535609	PA(19:1(9Z)/22:4(7	C44H77O8P			LMGP1001	81673		0.2183	[illegible]
Lung PO	11.91	931.5296	M+H	4	930.525834	PI(22:6(4Z,7Z,10Z,1	C51H79O13P			LMGP0601	80830		0.092587	[illegible]
Lung PO	7.43	527.3731	M+H	4	526.363459	PG(O-20:0/0:0)	C26H55O8P			LMGP0406	80025		0.37611	[illegible]
Lung PO	11.95	1019.47	M+H	4	1018.45849	PIP2(20:4(8Z,11Z,1	C45H81O19P3		HMDB0010136		61514	53480327	0.73326	[illegible]
Lung PO	11.88	695.4597	M+H	7	694.4573561	PA(22:4(7Z,10Z,13Z	C39H67O8P		HMDB011	LMGP1001	81955	52929382	0.74128	[illegible]
Lung PO	12.13	476.2048	M+H	9	475.19302	Tfa-VAL-TYR-VAL-OH	C21H28F3N3O6				44312		0.11145	[illegible]
Lung PO	7.42	504.3061	M+H	5	503.3011893	PE(20:3(8Z,11Z,14Z	C25H46NO7P		HMDB001	LMGP0205	77688	52925141	0.018144	[illegible]
Lung PO	11.96	878.4919	M+Na	3	855.5050341	PS(22:6(4Z,7Z,10Z,	C48H74NO10P		HMDB001	LMGP0301	78503	52925949	0.49065	[illegible]
Lung PO	7.45	488.293	M+Na	10	465.299142	N-docosahexaenoyl	C28H39N3O3			LMFA0802	75516		0.81515	[illegible]
Lung PO	11.81	744.4882	M+H	10	743.4737345	PS(18:3(6Z,9Z,12Z)	C39H70NO10P		HMDB011	LMGP0301	78034	52925480	0.78565	[illegible]
Lung PO	7.39	484.3255	M+H	3	483.3196	Tumonoic Acid H	C26H45NO7				65436		0.023354	[illegible]
Lung PO	11.32	643.5231	M+Na	7	620.5379754	DG(18:1n7/0:0/18:	C39H72O5		HMDB0056177			131766646	0.53441	[illegible]
Lung PO	11.9	491.2314	M+H	8	490.2202827	Estradiol acetate glu	C26H34O9		HMDB0061687			131770405	0.54138	[illegible]
Lung PO	11.78	743.4833	M+H	3	742.478485	PG(18:3(6Z,9Z,12Z)	C40H71O10P		HMDB001	LMGP0401	61932	52926638	0.51752	[illegible]
Lung PO	9.73	1198.7775	M+H	9	1197.759781	Galalpha1-3(Fucalph	C60H111NO22			LMSP0505	55660		0.80773	[illegible]
Lung PO	7.43	646.2798	M+Na	5	623.2876507	Leukotriene C5	C30H45N3O9S		HMDB0012993			53481570	0.47678	[illegible]
Lung PO	9.05	416.2757	M+Na	4	393.287909	PGH2-EA	C23H39NO4			LMFA0301	74984		0.65425	[illegible]
Lung PO	9.05	396.2924	M+H	7	395.28243	N-(3-hydroxypheny	C26H37NO2				45072		0.14525	[illegible]
Lung PO	11.97	1012.4716	M+Na	7	989.4897	Cobinamide:Cob(I)i	C48H72Co	C05774					0.87248	[illegible]
Lung PO	9.9	732.554	M+H	0	731.546505	PC(14:0/18:1(9E))[	C40H78NO8P				39233		0.83947	[illegible]
Lung PO	9.05	413.2665	M+Na	1	390.2770097	Nutriacholic acid	C24H38O4		HMDB0000467		2985	53477693	0.53256	[illegible]
Lung PO	11.32	685.5708	M+H	3	684.565254	Lysine-containing sic	C39H76N2O7			LMFA0000	45745		0.71752	[illegible]
Lung PO	11.32	657.5468	M+H	2	656.5379754	DG(20:3n9/0:0/18:	C42H72O5		HMDB0056237			131801940	0.34791	[illegible]
Lung PO	11.34	711.5858	M+H	9	710.584926	DG(21:0/22:6(4Z,7Z	C46H78O5			LMGL0201	4665		0.59285	[illegible]
Lung PO	9.45	442.3009	M+Na	7	419.314792	N-oleoyl histidine	C24H41N3O3			LMFA0802	75515		0.6186	[illegible]
Lung PO	11.88	365.1095	M+H	1	364.1019	His-Ser-OH	C15H16N4O7				65146		0.59647	[illegible]
Lung PO	10.01	752.555	M+H	5	751.5515904	PE(P-18:1(11Z)/20:	C43H78NO7P		HMDB0011417		62216	53480877	0.53501	[illegible]
Lung PO	9.65	630.4492	M+Na	4	607.45769	PC(24:0/0:0)	C32H66NO7P		HMDB001	LMGP0105	61714	24779481	0.062398	[illegible]
Lung PO	8.95	422.2368	M+H	7	421.23252	Phe Gln Lys	C20H31N5O5				17500		0.2239	[illegible]
Lung PO	5.61	167.0718	M+H	9	166.0629942	Ethyl 2-furanacrylat	C9H10O3		HMDB0041611		95984	1550909	0.78774	[illegible]
Lung PO	9.01	693.4557	M+H	10	692.4417061	PA(18:3(9Z,12Z,15Z	C39H65O8P		HMDB011	LMGP1001	40914	9547161	0.92123	[illegible]
Lung PO	11.89	503.2249	M+Na	3	480.23727	Ile Trp Tyr	C26H32N4O5				16193		0.88255	[illegible]
Lung PO	9.18	427.2671	M+H	5	426.2617536	Prostaglandin D2-1-	C23H38O7		HMDB0013653		85243	53481917	0.91569	[illegible]
Lung PO	7.66	405.2985	M+H	4	404.2926598	MG(22:5(4Z,7Z,10Z	C25H40O4		HMDB0011585		62367	53480993	0.2897	[illegible]
Lung PO	11.32	671.5592	M+H	3	670.5536255	DG(18:1(9Z)/22:4(7	C43H74O5		HMDB000	LMGL0201	4548	9543869	0.65765	[illegible]
Lung PO	9.45	441.2975	M+H	6	440.2926598	Suillin	C28H40O4		HMDB0034349		89868	131751551	0.69864	[illegible]
Lung PO	10.62	684.4576	M+H	3	683.4526046	PE(18:3(6Z,9Z,12Z)/	C37H66NO8P		HMDB000	LMGP0201	60526	52924383	0.27024	[illegible]
Lung PO	9.09	371.2559	M+Na	1	348.266445	Dinorlithocholic acid	C22H36O3			LMST04050010			0.52748	[illegible]
Lung PO	8.28	529.4983	M+H	1	528.490631	Linolenyl oleate	C36H64O2			LMFA0701	97131		0.73267	[illegible]
Lung PO	5.69	302.3053	M+H	0	301.298079	Safingol (L-threo-sp	C18H39NO2			LMSP0108	53955		0.72099	[illegible]
Lung PO	9.1	393.2974	M+H	6	392.2926598	Hyodeoxycholic acid	C24H40O4		HMDB0000733		5701	9963687	0.4171	[illegible]
Lung PO	10.91	549.4875	M+H	0	548.4804605	Cohibin B	C35H64O4		HMDB0031169		87462	131751148	0.10432	[illegible]
Lung PO	10.12	525.4698	M+H	6	524.459331	Linolenyl linolenate	C36H60O2			LMFA0701	97127		0.40441	[illegible]
Lung PO	8.88	504.4057	M+H	2	503.3974592	Tetracosatetraenoyl	C31H53NO4		HMDB0060158				0.2335	[illegible]
Lung PO	9.05	397.2928	M+H	5	396.287576	PGF2alpha isopropy	C23H40O5			LMFA0301	36147		0.22101	[illegible]
Lung PO	11.95	879.4963	M+H	6	878.494534	PI(18:4(6Z,9Z,12Z,1	C47H75O13P			LMGP0601	80438		0.91467	[illegible]
Lung PO	11.91	369.1873	M+H	2	368.18082	Asn His Val	C15H24N6O5				17829		0.73556	[illegible]
Lung PO	8.79	359.2709	M+H	5	358.265325	PGF2alpha-d4	C20H30D4O5			LMFA0301	3807		0.79458	[illegible]
Lung PO	11.86	635.3163	M+Na	1	612.327469	PI(19:1(9Z)/0:0)	C28H53O12P			LMGP0605	81183		0.90422	[illegible]
Lung PO	8.94	256.2641	M+H	2	255.2562147	Palmitamide	C16H33NO		HMDB001	LMFA0801	62905	69421	0.72872	[illegible]
Lung PO	11.98	856.5092	M+Na	1	833.5206842	PS(18:1(9Z)/22:6(4	C46H76NO10P		HMDB001	LMGP0301	77709	52925155	0.9186	[illegible]
Lung PO	8.37	494.3618	M+H	3	493.353227	PC(P-17:0/0:0)	C25H52NO6P			LMGP0107	40403		0.11701	[illegible]
Lung PO	10.14	867.6835	M+Na	3	844.6921071	PA(24:0/22:0)	C49H97O8P		HMDB0115446			131822184	0.70156	[illegible]
Lung PO	9.58	477.2821	M+H	5	476.2774	Cyclic-3,20-bis(1,2-e	C27H40O7	C14990			70487	11954096	0.90866	[illegible]
Lung PO	6.81	302.1416	M+H	10	301.13141	ent-6beta-Hydroxyk	C17H19NO	C12191			69356	443691	0.95841	[illegible]
Lung PO	11.98	855.4958	M+H	7	854.494534	PI(18:3(9Z,12Z,15Z)	C45H75O13P			LMGP0601	80976		0.43268	[illegible]
Lung PO	8.47	406.3295	M+Na	1	383.339944	N-stearoyl valine	C23H45NO3			LMFA0802	75504		0.58614	[illegible]
Lung PO	8.77	462.2524	M+Na	5	439.26555	Arg Lys His	C18H33N9O4				16758		0.04231	[illegible]
Lung PO	11.91	929.5113	M+H	7	928.510184	PI(22:6(4Z,7Z,10Z,1	C51H77O13P			LMGP0601	80831		0.74744	[illegible]
Lung PO	11.89	513.2653	M+H	5	512.255626	Lipoxin D4	C25H40N2O7S			LMFA0304	45971		0.7518	[illegible]
Lung PO	6.62	801.4945	M+Na	7	778.499619	PI(P-16:0/15:1(9Z))	C40H75O12P			LMGP0603	81084		0.97547	[illegible]
Lung PO	0.17	355.0401	M+Na	4	332.05219	Purine mononucleot	C10H13N4	C05325			66306	444450	0.16436	[illegible]
Lung PO	9.91	727.5303	M+H	4	726.5199564	PA(22:2(13Z,16Z)/1	C41H75O8P		HMDB011	LMGP1001	81928	52929355	0.76445	[illegible]
Lung PO	8.74	386.272	M+H	8	385.26169	Docosahexaenoyl Gl	C24H35NO3				62993		0.23324	[illegible]
Lung PO	9.05	459.3135	M+H	3	458.30775	Arg Arg Lys	C18H38N10O4				16563		0.086421	[illegible]
Lung PO	6.63	757.4711	M+Na	9	734.488659	PA(22:4(7Z,10Z,13Z	C42H71O8P			LMGP1001	81962		0.88505	[illegible]
Lung PO	8.08	239.2364	M+H	2	238.22967	hexadeca-7,11-dien	C16H30O				75249		0.13081	[illegible]
Lung PO	11.89	571.2838	M+H	6	570.272987	Pipercyclobutanami	C34H38N2O6		HMDB0036360		91504	131751971	0.25914	[illegible]
Lung PO	9.98	760.5843	M+H	1	759.5778051	PC(16:1(9Z)/18:0)	C42H82NO8P		HMDB000	LMGP0101	59451	52922448	0.79068	[illegible]
Lung PO	4.01	235.1314	M+H	6	234.1255944	Pterosin N	C14H18O3		HMDB0038218		92947	148710	0.86975	[illegible]
Lung PO	6.61	845.5223	M+H	6	844.510184	PI(17:0/18:4(6Z,9Z,	C44H77O13P			LMGP0601	80226		0.68623	[illegible]
Lung PO	8.2	399.2127	M+H	7	398.20262	Ser His Arg	C15H26N8O5				17208		0.93922	[illegible]
Lung PO	9.48	421.3275	M+H	9	420.32396	Dihomoursodeoxych	C26H44O4			LMST04020033			0.79283	[illegible]
Lung PO	10.66	920.638	M+H	1	919.6302351	PS(22:5(7Z,10Z,13Z	C52H90NO10P		HMDB0112863			131819994	0.1798	[illegible]
Lung PO	6.64	476.2798	M+H	6	475.2698892	PE(18:3(6Z,9Z,12Z)/	C23H42NO7P		HMDB001	LMGP0205	77683	52925136	0.06483	[illegible]
Lung PO	8.02	409.3062	M+Na	4	386.318481	Shibic acid	C26H42O2			LMFA0103	74367		0.88922	[illegible]
Lung PO	6.69	537.3239	M+H	10	536.311423	PG(20:2(11Z,14Z)/0	C26H49O9P			LMGP0405	80012		0.53024	[illegible]
Lung PO	11.06	853.6657	M+Na	0	830.676457	PA(i-24:0/a-21:0)	C48H95O8P		HMDB0115936			131822592	0.37935	[illegible]
Lung PO	7.45	403.2832	M+H	3	402.2770097	MG(0:0/22:6(4Z,7Z,	C25H38O4		HMDB001	LMGL0101	62340	46896303	0.88179	[illegible]
Lung PO	4.23	327.1424	M+H	4	326.13387	Gly Asn His	C12H18N6O5				16643		0.82266	[illegible]

Type	RT	Experimental.m	Adduct	PPM.Error	Molecular.Weight	Name	Formula	Kegg	HMDB	LipidMaps	Metlin	PubChem	p.value	FDR
Lung_PO	8.82	509.3262	M+H	5	508.3165055	PA(10:0/i-12:0)	C25H49O8P		HMDB0115611			131822267	0.50495	0.99788
Lung_PO	10.03	825.6414	M+Na	9	802.6451569	PA(i-24:0/i-19:0)	C46H91O8P		HMDB0115945			131822601	0.89812	0.99788
Lung_PO	11.95	1105.4472	M+Na	3	1082.454643	LPIM3(18:2(9Z,12Z	C45H79O27P			LMGP15040021			0.90675	0.99788
Lung_PO	11.93	1151.4526	M+Na	7	1128.471771	PIP3(18:2(9Z,12Z)/	C47H88O22P4		HMDB0010162		61540		0.55632	0.99788
Lung_PO	10.66	921.6411	M+H	2	920.635384	PI(18:1(9Z)/22:0)	C49H93O13P			LMGP0601	80950		0.14192	0.78557
Lung_PO	11.96	311.1305	M+H	4	310.1218465	Pentaporphyrin I	C20H14N4		HMDB0000839				0.53088	0.99788
Lung_PO	11.91	457.2326	M+H	5	456.2276905	LysoPA(20:5(5Z,8Z	C23H37O7P		HMDB0114748			25006224	0.97565	0.99788
Lung_PO	9.73	1185.7799	M+H	10	1184.784426	CL(8:0/10:0/11:0/2	C61H118O17P2		HMDB0117489			131824098	0.85819	0.99788
Lung_PO	9.73	1153.8104	M+Na	4	1130.816839	NeuAcalpha2-3Galb	C61H114N2O16			LMSP0601	56052		0.42606	0.99788
Lung_PO	9.85	782.5696	M+H	0	781.5621	PC(16:0/20:4) 1-palr	C44H81NO8P						0.44877	0.99788
Lung_PO	2.84	271.1164	M+H	5	270.11034	Idebenone Metaboli	C13H18O6				773		0.94838	0.99788
Lung_PO	11.9	483.2286	M+Na	9	460.24342	Trp Gln Lys	C22H32N6O5				16113		0.29991	0.95848
Lung_PO	9.72	664.4714	M+Na	7	641.48667	GlcCer(d14:2(4E,6E)	C36H67NO8			LMSP0501	103292		0.18858	0.84315
Lung_PO	9.94	810.5998	M+H	1	809.5934552	PC(20:3(5Z,8Z,11Z)	C46H84NO8P		HMDB000	LMGP0101	59813	53478955	0.92552	0.99788
Lung_PO	6.77	530.323	M+H	2	529.3168394	PE(22:4(7Z,10Z,13Z	C27H48NO7P		HMDB001	LMGP0205	77680	52925133	0.36473	0.98522
Lung_PO	9.59	894.5569	M+H	9	893.5418135	PS(DiMe(11,5)/DiM	C48H80NO12P		HMDB0061561			131770242	0.26392	0.93212
Lung_PO	11.02	771.5145	M+H	3	770.5097851	PG(18:1(9Z)/18:3(9	C42H75O10P		HMDB001	LMGP0401	61908	52927145	0.121	0.75019
Lung_PO	8.94	539.2867	M+Na	8	516.293451	L-Olivosyl-oleandoli	C26H44O1	C11991		LMPK040C	40996	443565	0.60434	0.99788
Lung_PO	11.85	793.497	M+H	6	792.4941351	PG(16:1(9Z)/22:6(4	C44H73O10P		HMDB001	LMGP0401	61874	52927163	0.19314	0.85239
Lung_PO	8.92	573.4127	M+Na	0	550.4236502	PC(O-18:0/O-3:1(2E	C29H61NO6P				40229		0.97723	0.99788
Lung_PO	11.9	445.2307	M+Na	4	422.243343	PA(17:1(9Z)/0:0)	C20H39O7P			LMGP1005	3887		0.93718	0.99788
Lung_PO	11.02	772.5178	M+H	7	771.5050346	PS(20:3(8Z,11Z,14Z	C41H74NO10P		HMDB011	LMGP0301	78257	52925703	0.17083	0.81387
Lung_PO	11.91	473.2389	M+H	1	472.23219	Tyr Lys Tyr	C24H32N4O6				18151		0.80222	0.99788
Lung_PO	11.88	697.4743	M+H	9	696.4730062	PA(18:1(11Z)/18:3(	C39H69O8P		HMDB0114903			131821911	0.73928	0.99788
Lung_PO	5.74	268.2641	M+Na	7	245.2767	Hexadecanal-d5	C16H27D5O				96520		0.90169	0.99788
Lung_PO	4.24	328.1455	M+Na	8	305.15869	Ser Ile Ser	C12H23N3O6				15808		0.86141	0.99788
Lung_PO	9.72	1166.7695	M+Na	3	1143.777358	MIPC(t20:0/26:0(2(	C58H114NO18P			LMSP0303	103224		0.83638	0.99788
Lung_PO	11.88	754.538	M+H	0	753.5308549	PC(18:3(9Z,12Z,15Z	C42H76NO8P		HMDB000	LMGP0101	59646	52922835	0.073196	0.61721
Lung_PO	11.91	419.1966	M+H	10	418.18524	Val Trp Asp	C20H26N4O6				16277		0.82491	0.99788
Lung_PO	11.82	745.4944	M+H	9	744.4941351	PG(18:3(6Z,9Z,12Z)	C40H73O10P		HMDB001	LMGP0401	61931	52926637	0.7557	0.99788
Lung_PO	8.97	421.2338	M+H	8	420.22335	His His Lys	C18H28N8O4				18791		0.6164	0.99788
Lung_PO	11.9	427.2246	M+Na	1	404.23515	Estradiol-17-phenyl	C27H32O3	C14645			70228	68577	0.24073	0.90432
Lung_PO	11.89	481.2339	M+Na	3	458.2433406	PA(20:4(5Z,8Z,11Z	C23H39O7P		HMDB006	LMGP1005	46749	42607498	0.86525	0.99788
Lung_PO	6.64	713.445	M+H	9	712.431539	PG(14:1(9Z)/18:4(6	C38H65O10P			LMGP0401	78949		0.90908	0.99788
Lung_PO	11.94	1149.4559	M+Na	9	1126.45612	PIP3(18:1(11Z)/20:	C47H86O22P4		HMDB0010157		61535		0.55634	0.99788
Lung_PO	12	835.5296	M+H	4	834.5258291	PI(16:1(9Z)/18:1(11	C43H79O13P		HMDB0009800		61185		0.33121	0.96927
Lung_PO	5.66	383.1684	M+Na	10	360.17573	Arg Asp Ala	C13H24N6O6				18542		0.90169	0.99788
Lung_PO	12.01	1011.4766	M+Na	1	988.4879095	Medicagenic acid 3-(	C48H76O21		HMDB0039487			4483634	0.73556	0.99788
Lung_PO	11.96	1130.4439	M+H	3	1129.433689	(15Z)-3-oxotetracos	C45H78N7O18P3S			LMFA07050097			0.71259	0.99788
Lung_PO	11.89	822.5283	M+H	0	821.520688	PS(22:4(7Z,10Z,13Z	C45H76NO10P			LMGP0301	78461		0.12673	0.7575
Lung_PO	11.88	621.308	M+H	7	620.296168	PI(20:4(5Z,8Z,11Z,1	C29H49O12P			LMGP0605	46748		0.68257	0.99788
Lung_PO	6.62	434.248	M+Na	2	411.2594	His Lys Lys	C18H33N7O4				17250		0.69342	0.99788
Lung_PO	9.97	724.5284	M+H	1	723.5202902	PE(P-16:0/20:4(8Z,1	C41H74NO7P		HMDB0011353		62164	53480847	0.99335	0.99963
Lung_PO	11.84	767.4806	M+H	7	766.478485	PG(18:3(6Z,9Z,12Z)	C42H71O10P		HMDB001	LMGP0401	61937	52926645	0.21525	0.87422
Lung_PO	5.65	249.1825	M+Na	0	226.19328	Physeteric acid	C14H26O2				34738		0.88201	0.99788
Lung_PO	11.99	891.5121	M+Na	3	868.5254357	PG(22:5(7Z,10Z,13Z	C50H77O10P		HMDB0116745			131823373	0.22316	0.87722
Lung_PO	11.86	577.3105	M+Na	1	554.32198	PG(10:0/10:0)[U]	C26H51O10P				40851		0.95314	0.99788
Lung_PO	8.66	310.3107	M+H	1	309.30317	Oleoyl Ethyl Amide	C20H39NO				44905		0.941	0.99788
Lung_PO	9.81	689.5512	M+H	5	688.540694	PA(O-20:0/16:1(9Z)	C39H77O7P			LMGP1002	82194		0.21427	0.87405
Lung_PO	11.93	1107.4531	M+Na	6	1084.470293	LPIM3(18:1(9Z)/0:0)	C45H81O27P			LMGP15040020			0.34352	0.97252
Lung_PO	12	834.5263	M+Na	1	811.5363342	PS(18:0/20:4(5Z,8Z	C44H78NO10P		HMDB001	LMGP0301	40824	91746195	0.28941	0.9552
Lung_PO	11.95	1156.4511	M+Na	3	1133.46499	CoA(24:0(3OH))	C45H82N7O18P3S				97423		0.75752	0.99788
Lung_PO	5.17	272.2557	M+H	10	271.251129	Myristoyl-EA	C16H33NO2			LMFA0804	46563		0.76384	0.99788
Lung_PO	11.95	1055.4607	M+Na	3	1032.474149	PIP2[3',4'](17:0/20:	C46H83O19P3			LMGP0801	40895		0.72014	0.99788
Lung_PO	11.87	617.3192	M+H	10	616.31804	Lys-Gln-Ala-Gly-Asp	C25H44N8O10				85172		0.92929	0.99788
Lung_PO	9.99	734.5683	M+H	2	733.5621551	PE(20:0/15:0)	C40H80NO8P		HMDB000	LMGP0201	60625	52924526	0.97072	0.99788
Lung_PO	8.08	313.2735	M+H	1	312.26645	Ricinoleic Acid meth	C19H36O3				45190		0.25957	0.92657
Lung_PO	10.94	809.6091	M+H	4	808.5982067	PA(22:2(13Z,16Z)/2	C47H85O8P		HMDB011	LMGP1001	81949	52929376	0.72969	0.99788
Lung_PO	11.86	797.5268	M+H	9	796.512551	MGDG(20:5(5Z,8Z,1	C47H72O10			LMGL0501	46652		0.492	0.99788
Lung_PO	3.53	331.2091	M+H	1	330.20155	Arg Gly Val	C13H26N6O4				17767		0.64117	0.99788
Lung_PO	11.94	906.5194	M+Na	7	883.5363347	PS(22:6(4Z,7Z,10Z,1	C50H78NO10P		HMDB011	LMGP0301	78509	52925955	0.21384	0.87405
Lung_PO	11.78	180.1031	M+H	7	179.0946287	N-methylphenylalan	C10H13NO2		HMDB0029224		86103	4657542	0.89694	0.99788
Lung_PO	11.86	537.3919	M+H	1	536.3841912	LysoPA(a-25:0/0:0)	C28H57O7P		HMDB0114762			131821854	0.51557	0.99788
Lung_PO	5.9	241.1781	M+H	7	240.1725446	L-Menthyl acetoacet	C14H24O3		HMDB0032369		88396	43045	0.39727	0.99788
Lung_PO	9.97	811.6223	M+H	1	810.6138568	PA(22:2(13Z,16Z)/2	C47H87O8P		HMDB0115315			131822093	0.60449	0.99788
Lung_PO	9.91	740.5269	M+H	6	739.5152049	PE(18:2(9Z,12Z)/18	C41H74NO8P		HMDB000	LMGP0201	40509	91746202	0.94658	0.99788
Lung_PO	6.1	299.2008	M+Na	10	276.20893	Moroctic acid	C18H28O2				34838		0.67795	0.99788
Lung_PO	11.89	515.2326	M+H	7	514.22162	Trp Tyr Phe	C29H30N4O5				17034		0.75063	0.99788
Lung_PO	6.74	336.1294	M+Na	5	313.13862	Gly His Thr	C12H19N5O5				17161		0.94003	0.99788
Lung_PO	3.22	317.1927	M+H	2	316.1859	Arg Ala Ala	C12H24N6O4				19784		0.9601	0.99788
Lung_PO	11.49	315.2319	M+H	0	314.2245802	progesterone	C21H30O2	C00410	HMDB000	LMST0203	402	5994	0.91098	0.99788
Lung_PO	0.16	236.0722	M+H	10	235.0626766	Cysteinyl-Asparagine	C7H13N3O4S		HMDB0028770		85684	18218192	0.93406	0.99788
Lung_PO	6.77	205.0865	M+H	3	204.07864	Levulinic Acid, 3-Ben	C12H12O3				44382		0.56634	0.99788
Lung_PO	4.46	385.184	M+H	3	384.17573	Thr His Gln	C15H24N6O6				16484		0.81872	0.99788
Lung_PO	11.94	357.1525	M+H	2	356.14443	His Ser Asn	C13H20N6O6				16900		0.74207	0.99788
Lung_PO	6.78	390.297	M+H	8	389.292994	N-arachidonoyl GAB	C24H39NO3			LMFA0802	46556		0.34609	0.97453
Lung_PO	7.63	333.2802	M+H	4	332.2715304	Ethyl Arachidonate	C22H36O2		HMDB0062725		404		0.86557	0.99788
Lung_PO	9.64	903.6082	M+Na	0	880.619339	PG(22:1(11Z)/22:4(	C50H89O10P			LMGP0401	79579		0.26641	0.93309
Lung_PO	10.94	823.6244	M+Na	7	800.6407397	SM(d18:0/22:1(13Z	C45H89N2O7P		HMDB0013466			53481785	0.81815	0.99788
Lung_PO	11.94	1059.4668	M+H	5	1058.454643	LPIM3(16:0/0:0)	C43H79O27P			LMGP15040017			0.72502	0.99788
Lung_PO	7.58	303.2294	M+H	8	302.2245802	ent-8(17),13(16),14	C20H30O2		HMDB0039486		94057	72780956	0.045314	0.53154
Lung_PO	11.97	884.5373	M+Na	5	861.5519848	PS(22:4(7Z,10Z,13Z	C48H80NO10P		HMDB011	LMGP0301	78473	52925919	0.33647	0.96933
Lung_PO	6.63	454.293	M+H	0	453.2855393	PC(13:0/0:0)[U]	C21H44NO7P				40277		0.033417	0.51292
Lung_PO	10.22	849.6989	M+H	5	848.687024	PG(O-20:0/22:0)	C48H97O9P			LMGP0402	79880		0.31329	0.96465
Lung_PO	9.78	828.5524	M+H	2	827.546505	PC(20:5(5Z,8Z,11Z,1	C48H78NO8P		HMDB000	LMGP0101	59955	52923373	0.67178	0.99788
Lung_PO	11.86	794.5036	M+H	9	793.4893845	PS(22:6(4Z,7Z,10Z,1	C43H72NO10P		HMDB011	LMGP0301	78485	52925931	0.078927	0.64219
Lung_PO	6.32	289.153	M+H	6	288.14739	N-(3-Indolylacetyl)-L	C16H20N2O3				34533		0.56323	0.99788
Lung_PO	9.26	642.4162	M+Na	9	619.421307	PE(12:0/15:1(9Z))	C32H62NO8P			LMGP0201	76596		0.72302	0.99788
Lung_PO	8.04	425.2875	M+Na	7	402.29545	Lys Lys Lys	C18H38N6O4				18451		0.93501	0.99788
Lung_PO	4.38	261.1107	M+H	10	260.10084	Glutamyl-Hydroxypr	C10H16N2O6				85734		0.69883	0.99788
Lung_PO	3.86	311.1474	M+H	5	310.14164	tetranor-PGAM	C16H22O6				96502		0.90116	0.99788
Lung_PO	12	863.5596	M+H	6	862.5571293	PI(16:0/20:2(11Z,1	C45H83O13P		HMDB000	LMGP0601	61171		0.079072	0.64219
Lung_PO	12.12	840.5734	M+H	2	839.5676348	PS(20:3(5Z,8Z,11Z),	C46H82NO10P		HMDB0112606			131819835	0.11975	0.7482
Lung_PO	9.99	736.5752	M+Na	8	713.58057	GlcCer(d15:1/20:0)	C41H79NO8			LMSP0501	103287		0.95016	0.99788
Lung_PO	11.9	417.2112	M+H	2	416.2046326	Ethyl 7-epi-12-hydro	C20H32O9		HMDB0036340		91484	131751966	0.83501	0.99788
Lung_PO	11.94	1127.4581	M+Na	3	1104.471771	PIP3(18:0/18:1(11Z	C45H88O22P4		HMDB0010152		61530		0.99534	0.99963
Lung_PO	9.72	1171.772	M+H	3	1170.768776	CL(a-13:0/a-13:0/a-	C60H116O17P2		HMDB0090576			131797123	0.8848	0.99788
Lung_PO	6.75	319.164	M+H	9	318.15393	Asn Ser Val	C12H22N4O6				16173		0.63239	0.99788
Lung_PO	6.53	270.21	M+H	4	269.20393	dinor-12-oxo Phyto	C16H19D5O3				96439		0.56657	0.99788
Lung_PO	9.79	717.5524	M+H	2	716.5468393	SM(d18:0/16:1(9Z))	C39H77N2O7P		HMDB0013463			53481779	0.074125	0.61733
Lung_PO	3.72	323.1476	M+H	8	322.1376157	N6-Galacturonyl-L-l	C12H22N2O8		HMDB0033106		88997	131751374	0.89101	0.99788
Lung_PO	10.79	870.6206	M+H	1	869.614585	PS(24:1(15Z)/18:2(	C48H88NO10P		HMDB0112918			102515141	0.3763	0.99245
Lung_PO	11.93	949.491	M+H	8	948.4917666	PGP(22:5(4Z,7Z,10Z	C50H78O13P2		HMDB0116495			131823150	0.85543	0.99788
Lung_PO	9.72	1125.7689	M+Na	5	1102.785539	NeuAcalpha2-3Galb	C59H110N2O16			LMSP0601	56051		0.94348	0.99788
Lung_PO	5.62	285.2027	M+Na	7	262.21172	Spermine NONOate	C10H26N6O2				44819		0.79707	0.99788
Lung_PO	5.03	245.0807	M+H	1	244.0735589	Oxyresveratrol	C14H12O4	C10273	HMDB012	LMPK1309	53239	5281717	0.83681	0.99788
Lung_PO	8.59	284.2951	M+H	1	283.2875148	Stearamide	C18H37NO	C13846	HMDB003	LMFA0801	34494	31292	0.83673	0.99788
Lung_PO	10.7	711.4895	M+H	9	710.4886562	PA(22:4(7Z,10Z,13Z	C40H71O8P		HMDB011	LMGP1001	81956	52929383	0.055384	0.56946
Lung_PO	6.25	328.0952	M+Na	5	305.10454	Pro Cys Ser	C11H19N3O5S				15876		0.97047	0.99788
Lung_PO	11.04	551.5023	M+H	2	550.496111	DG(P-14:0/18:1(9Z)	C35H66O4	C13864		LMGL0204	4697	5282285	0.023636	0.49303
Lung_PO	12.07	812.5428	M+Na	2	789.55198	GPSer(18:0/18:1(9Z	C42H80NO10P				40791		0.13596	0.77588
Lung_PO	11.92	908.5338	M+Na	8	885.5519848	PS(22:4(7Z,10Z,13Z	C50H80NO10P		HMDB0112811			131819942	0.20557	0.86265
Lung_PO	12.04	899.4879	M+H	5	898.4761155	PGP(18:3(6Z,9Z,12Z	C46H76O13P2		HMDB0013575			53481893	0.83948	0.99788
Lung_PO	6.29	290.1479	M+H	7	289.13862	Arg Asp	C10H19N5O5				23897		0.84252	0.99788
Lung_PO	9.73	1183.7775	M+H	9	1182.760109	Ganglioside GM3 (d1	C59H110N2O21		HMDB0011916		62618	53481192	0.68677	0.99788
Lung_PO	11.85	775.5387	M+H	4	774.528201	MGDG(18:3(9Z,12Z	C45H74O10			LMGL0501	75584		0.091789	0.67155
Lung_PO	11.91	927.4953	M+Na	5	904.510184	PI(22:6(4Z,7Z,10Z,1	C49H77O13P			LMGP0601	80821		0.32786	0.96927
Lung_PO	6.64	436.2819	M+Na	1	413.292994	N-docosahexaenoyl	C26H39NO3			LMFA0802	75487		0.040413	0.51292
Lung_PO	6.36	235.1698	M+H	2	234.1619799	Cyclodehydroisolubi	C15H22O2		HMDB0037038		92017	14258974	0.53208	0.99788
Lung_PO	12.1	842.565	M+H	5	841.5621551	PE(22:4(7Z,10Z,13Z	C49H80NO8P		HMDB0009604		60993	53479902	0.10637	0.71048
Lung_PO	11.98	854.4926	M+H	5	853.4893845	PS(22:6(4Z,7Z,10Z,1	C48H72NO10P		HMDB011	LMGP0301	78504	52925950	0.42788	0.99788
Lung_PO	10.03	839.6419	M+Na	10	816.660807	PA(i-19:0/a-25:0)	C47H93O8P		HMDB0115824			131822480	0.96157	0.99788
Lung_PO	9.29	598.3881	M+Na	7	575.3950897	PC(22:2(13Z,16Z)/0	C30H58NO7P		HMDB001	LMGP0105	76577	52924059	0.34791	0.97569
Lung_PO	11.51	441.3717	M+H	2	440.3654308	Sebiferic acid	C30H48O2		HMDB0036774		91794	5321206	0.85835	0.99788
Lung_PO	3.94	389.2506	M+H	0	388.24342	Leu Arg Thr	C16H32N6O5				16383		0.84124	0.99788
Lung_PO	8.04	621.2961	M+Na	8	598.311818	PI(18:1(9Z)/0:0)	C27H51O12P			LMGP0605	46747		0.28707	0.9552
Lung_PO	9.77	696.493	M+H	5	695.4889901	PE(18:4(6Z,9Z,12Z,1	C39H70NO7P		HMDB0009213		60619	53479705	0.45959	0.99788
Lung_PO	9.8	481.4982	M+H	1	480.49063	Mycocerosic acid	C32H64O2				45762		0.65545	0.99788
Lung_PO	9.98	703.5726	M+H	3	702.5675748	SM(d18:1/16:0)	C39H79N2O6P		HMDB006	LMSP0301	41586	9939941	0.81058	0.99788
Lung_PO	11.99	493.2219	M+Na	5	470.2304534	Lucidenic acid D1	C27H34O7		HMDB0038199		92933	14109376	0.9852	0.99788
Lung_PO	11.92	341.1586	M+H	5	340.14952	Asn His Ala	C13H20N6O5				17044		0.64034	0.99788
Lung_PO	10.73	922.652	M+Na	1	899.6615352	PS(20:1(11Z)/24:1(	C50H94NO10P		HMDB0112566			131819813	0.034543	0.51292
Lung_PO	11.92	973.4882	M+Na	9	950.5074166	PGP(22:5(4Z,7Z,10Z	C50H80O13P2		HMDB0116494			131823149	0.82401	0.99788
Lung_PO	11.91	453.2146	M+H	3	452.20597	Phe Thr Trp	C24H28N4O5				16530		0.52656	0.99788
Lung_PO	9.73	1123.7516	M+Na	7	1100.769889	NeuAcalpha2-3Galb	C59H108N2O16			LMSP0601	56053		0.87995	0.99788
Lung_PO	9.74	1156.7768	M+Na	7	1133.780116	Trihexosylceramide (	C60H111NO18		HMDB0004883		7140	20057318	0.93283	0.99788
Lung_PO	4.55	192.1393	M+H	5	191.13101	Pentedrone	C12H17NO				96597		0.88997	0.99788
Lung_PO	3.86	312.1508	M+Na	8	289.16377	Gly Thr Leu	C12H23N3O5				16182		0.76241	0.99788
Lung_PO	9.11	247.1676	M+Na	3	224.17763	Megatomic acid	C14H24O2			LMFA0103	45793		0.40498	0.99788
Lung_PO	10.04	840.6481	M+Na	3	817.6560554	PC(24:0/14:0)	C46H92NO8P		HMDB0008755		60201	53479431	0.94674	0.99788
Lung_PO	11.96	969.5056	M+Na	2	946.5183752	CL(8:0/8:0/8:0/11:0	C44H84O17P2		HMDB0116799			131823408	0.97019	0.99788
Lung_PO	11.89	524.2761	M+Na	3	501.2855393	PE(20:4(5Z,8Z,11Z,1	C25H44NO7P		HMDB001	LMGP0205	46715	42607465	0.16564	0.81014
Lung_PO	9.04	391.2845	M+Na	7	368.2926598	Octadecyl fumarate	C22H40O4		HMDB003	LMFA0701	92842	6436905	0.85428	0.99788
Lung_PO	8.78	381.2946	M+Na	8	358.3083098	MG(0:0/18:0/0:0)	C21H42O4		HMDB0011535		62319	79075	0.019915	0.4765
Lung_PO	8.91	448.3793	M+Na	7	425.3868945	Tetracosanoylglycine	C26H51NO3		HMDB0013310			53481665	0.3553	0.98356
Lung_PO	0.38	324.0606	M+H	5	323.051851	Cytidine 2'-phospha	C9H14N3O8P		HMDB0011692		62430	53481031	0.8338	0.99788
Lung_PO	12.08	813.5463	M+Na	7	790.551259	PA(21:0/22:6(4Z,7Z	C46H79O8P			LMGP1001	3883		0.1688	0.81081
Lung_PO	7.43	487.2818	M+H	0	486.2746402	PA(20:4(5Z,8Z,11Z	C25H43O7P		HMDB0011156		62007	53480670	0.40678	0.99788
Lung_PO	11.96	885.5434	M+H	6	884.5414792	PI(22:3(10Z,13Z,16	C47H81O13P		HMDB0009908		61293		0.049858	0.55171
Lung_PO	11.02	837.6407	M+H	5	836.6295068	PA(22:4(7Z,10Z,13Z	C49H89O8P		HMDB0115347			131822104	0.22853	0.88666
Lung_PO	11.91	297.1308	M+H	8	296.12599	Idebenone Metaboli	C15H20O6				760		0.53295	0.99788
Lung_PO	9.74	1182.7821	M+H	8	1181.764866	Fucalpha1-2Galalph	C60H111NO21			LMSP0506	55922		0.82741	0.99788
Lung_PO	11.89	517.2682	M+H	7	516.25706	Idebenone Metaboli	C25H40O11				770		0.93059	0.99788
Lung_PO	11.91	1068.5007	M+H	4	1067.497266	Cyclolinopeptide I	C55H73N9O9S2		HMDB0036552		91633	636981	0.81	0.99788
Lung_PO	9.73	723.538	M+Na	4	700.551926	SM(d16:1/18:1)	C39H77N2O6P			LMSP0301	83741		0.053547	0.5677
Lung_PO	10.17	553.5016	M+H	7	552.490631	Linoleyl arachidonat	C38H64O2			LMFA0701	97146		0.33513	0.96933
Lung_PO	9.14	254.248	M+H	1	253.240564	Palmitoleamide	C16H31NO			LMFA0801	97431		0.30914	0.95908
Lung_PO	12.01	832.5112	M+Na	2	809.5206842	PE(MonoMe(9,5)/D	C44H76NO10P		HMDB0061547			131770219	0.85963	0.99788
Lung_PO	9.04	333.1288	M+H	1	332.1219656	Mytilin A	C13H20N2O8		HMDB0039002		93608		0.6269	0.99788
Lung_PO	10.24	909.7197	M+Na	10	886.7390573	PA(a-25:0/i-24:0)	C52H103O8P		HMDB0115774			131822430	0.98486	0.99788
Lung_PO	7.82	382.2918	M+H	9	381.287909	PGF2alpha dimethyl	C22H39NO4			LMFA0301	36145		0.784	0.99788
Lung_PO	7.15	331.2234	M+Na	5	308.2327	Palmityl Trifluorome	C17H31F3O				63017		0.99505	0.99963
Lung_PO	6.59	258.2421	M+H	3	257.23548	D-erythro-Sphingosi	C15H31NO2				64626		0.75575	0.99788
Lung_PO	11.04	1064.2929	M+Na	5	1041.308488	trans-2-Enoyl-OPC8	C39H62N7O18P3S		HMDB001	LMFA07050382		53480664	0.5541	0.99788
Lung_PO	11.06	839.657	M+H	5	838.6451569	PA(24:1(15Z)/22:2(	C49H91O8P		HMDB0115475			131822212	0.64966	0.99788
Lung_PO	10.63	709.4818	M+H	2	708.4730062	PA(22:5(7Z,10Z,13Z	C40H69O8P		HMDB0115377			131822134	0.12224	0.75019
Lung_PO	8.99	695.4789	M+H	10	694.4784855	PG(i-13:0/i-17:0)	C36H71O10P		HMDB0116683			131823316	0.66915	0.99788
Lung_PO	9.22	226.9528	M+H	2	225.945003	Ethyl 3-iodo-2E-acry	C5H7IO2			LMFA0109	96865		0.29217	0.9552
Lung_PO	11.95	1116.4518	M+H	9	1115.45442	CoA(24:1(15Z))	C45H80N7O17P3S				75436		0.82462	0.99788
Lung_PO	11.96	853.482	M+H	5	852.478884	PI(22:6(4Z,7Z,10Z,1	C45H73O13P			LMGP0601	80811		0.80017	0.99788
Lung_PO	10.64	972.6652	M+Na	1	949.6771853	PE-NMe2(13D5/13E	C54H96NO10P		HMDB0114701			131821808	0.037239	0.51292
Lung_PO	11.92	329.1604	M+H	3	328.1522	PGDM	C16H24O7				96515		0.56966	0.99788
Lung_PO	5.47	458.2083	M+Na	9	435.22302	Arg Phe Asn	C19H29N7O5				16817		0.37638	0.99245
Lung_PO	11.89	550.292	M+Na	3	527.3011893	LysoPE(0:0/22:5(4Z	C27H46NO7P		HMDB001	LMGP0205	62282	53480943	0.064855	0.60419
Lung_PO	6.29	330.3302	M+H	1	329.32319	Oleoyl Ethanolamide	C20H35D4NO2				96544		0.52495	0.99788
Lung_PO	9.9	766.5682	M+H	8	765.5672404	PC(P-16:0/20:4(8Z,	C44H80NO7P		HMDB0011221		62051	53480687	0.3769	0.99245
Lung_PO	8.79	298.3105	M+H	0	297.30317	Palmitoyl N-Isoprop	C19H39NO				64930		0.67088	0.99788
Lung_PO	11.91	381.1858	M+H	6	380.18082	Pro His Gln	C16H24N6O5				16468		0.85652	0.99788
Lung_PO	11.86	671.3191	M+Na	4	648.327469	PI(22:4(7Z,10Z,13Z	C31H53O12P			LMGP0605	81178		0.40393	0.99788
Lung_PO	6.69	405.2593	M+Na	5	382.27192	Unoprostone	C22H38O5				45686		0.55988	0.99788
Lung_PO	9.72	1199.8012	M+H	5	1198.800076	CL(8:0/a-13:0/15:0,	C62H120O17P2		HMDB0120189			131826798	0.96333	0.99788
Lung_PO	9.08	376.3183	M+H	7	375.313729	N-(5Z,8Z,11Z,14Z-d	C24H41NO2			LMFA0804	75543		0.14531	0.78557
Lung_PO	5.27	441.2103	M+H	7	440.20597	Phe Gln Phe	C23H28N4O5				16088		0.76869	0.99788
Lung_PO	10.13	868.6851	M+H	7	867.6717055	PC(20:3(5Z,8Z,11Z)	C50H94NO8P		HMDB0008381		59827	53478983	0.81444	0.99788
Lung_PO	11.55	469.4028	M+Na	3	446.412381	Lauryl linolenate	C30H54O2			LMFA0701	97080		0.77123	0.99788
Lung_PO	5.56	387.2893	M+H	0	386.2821	Testosterone isocap	C25H38O3	C14663			2784	231084	0.26706	0.93309

Type	RT	erimental.	Adduct	PPM.Error	Molecular.Weig	Name	Formula	Kegg	HMDB	LipidMaps	Metlin	PubChem
Lung PO	11.86	795.5087	M+H	9	794.494534	PI(16:0/15:1(9Z))	C40H75O13P			LMGP0601	80184	
Lung PO	0.51	223.9638	M+Na	10	200.9765637	Cysteine-S-sulfate	C3H7NO5S	C05824	HMDB0000731		58010	7068808
Lung PO	11.26	588.9558	M+H	4	587.94609	Inosine 5'-tetraphos	C10H16N4	C03614			63400	440063
Lung PO	9.89	784.5806	M+H	6	783.5778051	PC(20:3(5Z,8Z,11Z)	C44H82NO8P		HMDB000	LMGP0101	59810	53478949
Lung PO	10.16	895.7104	M+Na	3	872.7234072	PA(i-24:0/i-24:0)	C51H101O8P		HMDB0115949			131822605
Lung PO	12.01	864.5616	M+H	9	863.5465055	PE-NMe2(22:6(4Z,7	C51H78NO8P		HMDB0114601			131821708
Lung PO	10.25	908.7122	M+H	2	907.7030061	PE-NMe2(24:0/22:4	C53H98NO8P		HMDB0114627			131821734
Lung PO	9.93	774.5659	M+H	2	773.557073	PS(P-16:0/20:1(11Z	C42H80NO9P			LMGP0301	78759	
Lung PO	5.47	457.2051	M+H	7	456.20089	Tyr Gln Phe	C23H28N4O6				17604	
Lung PO	8.77	365.3044	M+Na	5	342.3133952	MG(P-18:0e/0:0/0:0)	C21H42O3		HMDB0011153		62004	11824075
Lung PO	10.2	938.815	M+H	7	937.814166	TG(20:2(11Z,14Z)/1	C61H99D5O6			LMGL0301	4714	
Lung PO	5.26	367.1738	M+H	4	366.16517	Asn Pro His	C15H22N6O5				15947	
Lung PO	11.92	303.1469	M+H	6	302.13789	Phe His	C15H18N4O3				23660	
Lung PO	9.68	449.3649	M+H	5	448.35526	Teasterone	C28H48O4	C15791			64149	23724583
Lung PO	11.01	836.6365	M+Na	1	813.648254	DGTA(18:1/22:4(10	C50H87NO7			LMGL0000	46619	
Lung PO	6.25	327.0918	M+Na	2	304.1019	Asp Asn Gly	C10H16N4O7				15760	
Lung PO	12.02	763.5216	M+Na	4	740.535609	PA(22:2(13Z,16Z)/1	C42H77O8P			LMGP1001	81930	
Lung PO	9.78	715.5215	M+H	8	714.5199564	PA(15:0/22:2(13Z,1	C40H75O8P		HMDB011	LMGP1001	81322	52928749
Lung PO	10.23	881.7023	M+Na	6	858.7077571	PA(a-25:0/22:0)	C50H99O8P		HMDB0115756			131822412
Lung PO	11.9	403.1957	M+H	5	402.1863	Gln Gln Gln	C15H26N6O7				20513	
Lung PO	11.88	747.5084	M+H	6	746.496901	MGDG(18:3(9Z,12Z	C43H70O10			LMGL0501	46655	
Lung PO	11.99	833.5347	M+Na	5	810.541089	PG(20:4(5Z,8Z,11Z	C45H79O10P			LMGP0401	79462	
Lung PO	11.9	485.2533	M+H	5	484.24342	Tyr Arg Phe	C24H32N6O5				18415	
Lung PO	0.39	651.1121	M+Na	8	628.1276296	CMP-N-glycoloyl-be	C20H29N4O17P		HMDB0062569			131770458

Name	1. Metabolites affected by drug only				1. Metabolites affected by drug only				2. Dose response				. Metabolites dysregulated by radiation o			
	Day 4				Day 4				Day 4				Day 4			
	ami_50/Vehicle				ami_200/Vehicle				Rad + ami_200/Rad + ami_50				Rad/Vehicle			
	p.value	FDR	Fold.Chang	log2.FC	p.value	FDR	Fold.Chang	log2.FC	p.value	FDR	Fold.Chang	log2.FC	p.value	FDR	Fold.Chang	log2.FC
PI(16:0/15:1(9Z))	0.32897	0.96927	1.0881	0.12175	0.7018	0.91262	0.97102	-0.042421	0.45649	0.89349	0.90402	-0.14557	0.98194	0.98445	1.0077	0.011071
Cysteine-S-sulfate	0.49941	0.99788	0.94198	-0.08623	0.84437	0.94707	0.99461	-0.007801	0.59607	0.89491	0.90717	-0.14056	0.30082	0.3922	0.90252	-0.14796
Inosine 5'-tetraphos	0.61363	0.99788	1.0519	0.072937	0.42518	0.83975	1.1684	0.22449	0.4317	0.89349	0.8665	-0.20673	0.30843	0.40006	0.85173	-0.23154
PC(20:3(5Z,8Z,11Z)	0.31915	0.96559	1.1052	0.14433	0.43658	0.83975	1.0944	0.13012	0.44791	0.89349	0.91062	-0.13507	0.064256	0.15962	0.82344	-0.28027
PA(i-24:0/i-24:0)	0.68382	0.99788	0.95509	-0.066288	0.39901	0.83975	1.1726	0.22966	0.78395	0.94432	0.96094	-0.057477	0.014011	0.073068	0.74395	-0.42672
PE-NMe2(22:6(4Z,7	0.11483	0.73592	0.87451	-0.19346	0.58883	0.88417	0.95536	-0.065884	0.85952	0.95666	0.96964	-0.044482	0.023238	0.091917	0.77032	-0.37648
PE-NMe2(24:0/22:4	0.55978	0.99788	1.2063	0.27054	0.077091	0.64535	1.51	0.59452	0.28163	0.89349	0.77656	-0.36484	0.96793	0.97563	1.0432	0.060992
PS(P-16:0/20:1(11Z	0.56334	0.99788	0.95878	-0.060727	0.91055	0.96831	1.0305	0.043365	0.43322	0.89349	0.89508	-0.15991	0.023366	0.092034	0.81106	-0.30212
Tyr Gln Phe	0.68334	0.99788	0.95268	-0.069939	0.93478	0.97555	1.0059	0.008543	0.3966	0.89349	0.87605	-0.19092	0.25918	0.35683	0.88199	-0.18116
MG(P-18:0e/0:0/0:0)	0.017139	0.47481	0.75985	-0.39622	0.36518	0.83954	0.90577	-0.14278	0.49762	0.89349	0.8767	-0.18984	0.0065958	0.053434	0.68975	-0.53586
TG(20:2(11Z,14Z)/1	0.33347	0.96933	0.8993	-0.15312	0.78582	0.93285	1.0883	0.12208	0.68055	0.90545	0.91043	-0.13538	0.059729	0.15467	0.78383	-0.35139
Asn Pro His	0.82423	0.99788	0.9755	-0.03578	0.46681	0.84465	1.0956	0.13167	0.36	0.89349	0.85589	-0.2245	0.20963	0.31358	0.8703	-0.20042
Phe His	0.44962	0.99788	1.0965	0.13288	0.29524	0.78793	1.1482	0.19934	0.3255	0.89349	0.85124	-0.23235	0.26433	0.36106	0.83662	-0.25735
Teasterone	0.50165	0.99788	1.1055	0.1447	0.16617	0.70924	1.2817	0.35803	0.44155	0.89349	0.82406	-0.27919	0.97645	0.98032	0.98361	-0.023836
DGTA(18:1/22:4(10	0.21272	0.87383	0.88049	-0.18363	0.026095	0.62003	0.77813	-0.36191	0.47338	0.89349	0.89155	-0.16561	0.054831	0.14889	0.79677	-0.32777
Asp Asn Gly	0.83581	0.99788	1.0202	0.028785	0.79142	0.93445	1.047	0.066221	0.30954	0.89349	0.85411	-0.22751	0.15921	0.26846	0.85157	-0.2318
PA(22:2(13Z,16Z)/1	0.24991	0.91043	0.91763	-0.12401	0.90701	0.96769	1.0103	0.01476	0.48097	0.89349	1.2518	0.324	0.006864	0.053669	0.74597	-0.42282
PA(15:0/22:2(13Z,1	0.50492	0.99788	1.0588	0.082376	0.60945	0.88647	1.0639	0.089409	0.82134	0.95184	1.0274	0.039064	0.0087539	0.059368	0.77802	-0.36211
PA(a-25:0/22:0)	0.68852	0.99788	1.0386	0.054574	0.22934	0.753	1.1908	0.25191	0.98159	0.99519	0.98749	-0.018168	0.20004	0.30505	0.88028	-0.18397
Gln Gln Gln	0.91169	0.99788	1.0022	0.0031576	0.33783	0.82432	1.1814	0.24056	0.28335	0.89349	0.85135	-0.23217	0.30072	0.3922	0.86034	-0.21702
MGDG(18:3(9Z,12Z	0.59986	0.99788	1.0427	0.060313	0.58886	0.88417	1.0686	0.095689	0.4819	0.89349	0.89424	-0.16126	0.29348	0.38633	0.91414	-0.12952
PG(20:4(5Z,8Z,11Z	0.89534	0.99788	1.011	0.015734	0.57759	0.88119	0.97257	-0.04012	0.51577	0.89491	0.92542	-0.11182	0.032047	0.11093	0.86134	-0.21535
Tyr Arg Phe	0.7361	0.99788	1.0378	0.053541	0.67463	0.9008	1.0919	0.12679	0.29522	0.89349	0.85029	-0.23398	0.11564	0.22269	0.81802	-0.2898
CMP-N-glycoloyl-be	0.55112	0.99788	1.1122	0.15347	0.073213	0.6378	0.72285	-0.46823	0.034345	0.89349	1.467	0.55285	0.044155	0.13062	0.76475	-0.38694

Name	4. Metabolites that return to normal level				4. Metabolites that return to normal level				5. Does the dose make a difference?				Does the dose make a differen		
	Day 4				Day 4				Day 4				Day 4		
	Rad + ami_50/Rad				Rad + ami_200/Rad				ami_200/ami_50				Rad + ami_200/Rad + ami_50/Ra		
	p.value	FDR	Fold.Chang	log2.FC	p.value	FDR	Fold.Chang	log2.FC	p.value	FDR	Fold.Chang	log2.FC	p.value	FDR	f.value
PI(16:0/15:1(9Z))	0.78015	0.99967	1.0476	0.067057	0.56062	0.86322	0.94703	-0.078518	0.19828	0.82892	0.89244	-0.16417	0.70533	0.95682	0.35496
Cysteine-S-sulfate	0.64175	0.99967	0.93796	-0.092403	0.27943	0.86322	0.85089	-0.23296	0.74967	0.91192	1.0559	0.078429	0.58336	0.95682	0.55302
Inosine 5'-tetraphos	0.71135	0.99967	1.0792	0.10997	0.60888	0.86649	0.93513	-0.096756	0.65984	0.88086	1.1108	0.15155	0.69243	0.95682	0.37406
PC(20:3(5Z,8Z,11Z)	0.30137	0.99967	1.1383	0.18691	0.85903	0.95188	1.0366	0.051835	0.89231	0.95186	0.9902	-0.014202	0.56543	0.95682	0.58594
PA(i-24:0/i-24:0)	0.92706	0.99967	1.0002	0.0003494	0.68038	0.88607	0.96118	-0.057128	0.26217	0.84965	1.2277	0.29595	0.91972	0.98106	0.084021
PE-NMe2(22:6(4Z,7	0.42208	0.99967	0.90246	-0.14806	0.30268	0.86322	0.87506	-0.19254	0.47155	0.84965	1.0925	0.12758	0.57271	0.95682	0.57243
PE-NMe2(24:0/22:4	0.86226	0.99967	0.99553	-0.006457	0.41541	0.86322	0.77309	-0.37129	0.30527	0.84965	1.2518	0.32398	0.52946	0.95682	0.65555
PS(P-16:0/20:1(11Z	0.69643	0.99967	1.0658	0.091904	0.60437	0.8654	0.95396	-0.068003	0.59657	0.8714	1.0748	0.10409	0.68276	0.95682	0.38863
Tyr Gln Phe	0.99408	0.99967	1.015	0.021534	0.31691	0.86322	0.88922	-0.16938	0.81084	0.92931	1.0559	0.078482	0.5558	0.95682	0.60409
MG(P-18:0e/0:0/0:0)	0.59179	0.99967	0.93578	-0.095752	0.17635	0.86322	0.8204	-0.28559	0.21306	0.83999	1.192	0.25344	0.44295	0.95682	0.84672
TG(20:2(11Z,14Z)/1	0.4348	0.99967	0.89735	-0.15626	0.17431	0.86322	0.81697	-0.29164	0.35738	0.84965	1.2102	0.2752	0.44585	0.95682	0.83965
Asn Pro His	0.92493	0.99967	1.039	0.055159	0.2954	0.86322	0.88925	-0.16935	0.35535	0.84965	1.1231	0.16745	0.52615	0.95682	0.66222
Phe His	0.28262	0.99967	1.204	0.26778	0.86636	0.95461	1.0249	0.035422	0.72324	0.90534	1.0471	0.066461	0.4799	0.95682	0.76045
Teasterone	0.68182	0.99967	0.96239	-0.055312	0.14484	0.86322	0.79306	-0.3345	0.40601	0.84965	1.1594	0.21333	0.42959	0.95682	0.87986
DGTA(18:1/22:4(10	0.59275	0.99967	0.94966	-0.074523	0.1485	0.86322	0.84667	-0.24014	0.078261	0.66058	0.88376	-0.17828	0.41125	0.95682	0.92722
Asp Asn Gly	0.90662	0.99967	1.0333	0.04721	0.28216	0.86322	0.88252	-0.1803	0.92531	0.96796	1.0263	0.037436	0.47466	0.95682	0.77224
PA(22:2(13Z,16Z)/1	0.039377	0.99967	0.66985	-0.57808	0.25385	0.86322	0.83852	-0.25408	0.5107	0.85695	1.101	0.13877	0.17139	0.95682	1.9206
PA(15:0/22:2(13Z,1	0.7712	0.99967	0.97263	-0.04003	0.95996	0.98872	0.99933	-0.000966	0.95892	0.98365	1.0049	0.0070327	0.95396	0.98812	0.047235
PA(a-25:0/22:0)	0.68345	0.99967	0.97823	-0.03176	0.63829	0.87674	0.96598	-0.049929	0.3607	0.84965	1.1466	0.19733	0.89497	0.9745	0.11156
Gln Gln Gln	0.48254	0.99967	1.1127	0.15402	0.58015	0.86322	0.94727	-0.078148	0.352	0.84965	1.1789	0.2374	0.48993	0.95682	0.73829
MGDG(18:3(9Z,12Z	0.70699	0.99967	0.96966	-0.044443	0.1992	0.86322	0.86711	-0.20571	0.89892	0.95674	1.0248	0.035376	0.50036	0.95682	0.71577
PG(20:4(5Z,8Z,11Z	0.95842	0.99967	1.0093	0.013386	0.43349	0.86322	0.93405	-0.098434	0.55537	0.86106	0.96202	-0.055854	0.67774	0.95682	0.39629
Tyr Arg Phe	0.52896	0.99967	1.1003	0.13794	0.62673	0.87337	0.9356	-0.096035	0.86796	0.94473	1.0521	0.073252	0.54332	0.95682	0.62812
CMP-N-glycoloyl-be	0.94224	0.99967	0.9984	-0.002314	0.030321	0.69268	1.4646	0.55054	0.042961	0.5719	0.64991	-0.6217	0.027547	0.85436	4.2827

Name	1. Metabolites affected by drug only				1. Metabolites affected by drug only				2. Dose response				. Metabolites dysregulated by radiation o			
	Day 9				Day 9				Day 9				Day 9			
	ami_50/Vehicle				ami_200/Vehicle				Rad + ami_200/Rad + ami_50				Rad/Vehicle			
	p.value	FDR	Fold.Chang	log2.FC	p.value	FDR	Fold.Chang	log2.FC	p.value	FDR	Fold.Chang	log2.FC	p.value	FDR	Fold.Chang	log2.FC
PI(16:0/15:1(9Z))	0.020812	0.31104	1.2292	0.29769	0.60828	0.99988	0.97074	-0.042843	0.47278	0.88892	1.0547	0.076832	0.17502	0.99965	1.0944	0.13016
Cysteine-S-sulfate	0.18995	0.33678	1.1961	0.25834	0.58809	0.99988	0.9407	-0.088192	0.74991	0.94197	0.98351	-0.023992	0.8216	0.99965	0.96645	-0.049238
Inosine 5'-tetraphos	0.08036	0.31104	1.6991	0.76474	0.72485	0.99988	1.14	0.18904	0.90443	0.9778	0.99395	-0.008755	0.77837	0.99965	1.0795	0.1104
PC(20:3(5Z,8Z,11Z)	0.085956	0.31104	1.2376	0.30759	0.61423	0.99988	0.96259	-0.05501	0.45797	0.88892	0.96168	-0.056373	0.56117	0.99965	1.0633	0.088549
PA(i-24:0/i-24:0)	0.19003	0.33678	1.3432	0.42571	0.7002	0.99988	0.96744	-0.047753	0.67911	0.92029	0.95623	-0.064573	0.3032	0.99965	0.85179	-0.23142
PE-NMe2(22:6(4Z,7	0.20078	0.344	1.2232	0.29071	0.89379	0.99988	0.99356	-0.009315	0.89365	0.97567	0.99983	-0.000245	0.49147	0.99965	0.90871	-0.13811
PE-NMe2(24:0/22:4	0.4244	0.52442	1.2811	0.35741	0.53293	0.99988	1.2441	0.31513	0.25599	0.88892	0.85784	-0.22122	0.82014	0.99965	0.95086	-0.0727
PS(P-16:0/20:1(11Z	0.12348	0.31104	1.2926	0.37032	0.91369	0.99988	1.0009	0.0013344	0.41584	0.88892	0.94029	-0.088823	0.40539	0.99965	0.89387	-0.16187
Tyr Gln Phe	0.10933	0.31104	1.4469	0.53295	0.66044	0.99988	1.106	0.14531	0.44089	0.88892	0.94179	-0.08652	0.54134	0.99965	1.0814	0.11288
MG(P-18:0e/0:0/0:0)	0.10484	0.31104	1.306	0.38519	0.39874	0.99988	1.1401	0.18916	0.17029	0.88892	0.85976	-0.21799	0.90782	0.99965	1.0204	0.029133
TG(20:2(11Z,14Z)/1	0.16708	0.32365	1.5024	0.58726	0.78018	0.99988	0.97863	-0.031159	0.10996	0.88892	0.77577	-0.36631	0.27989	0.99965	0.82507	-0.27741
Asn Pro His	0.11512	0.31104	1.3994	0.48485	0.82091	0.99988	1.0679	0.094811	0.40784	0.88892	0.94568	-0.080583	0.75166	0.99965	1.0495	0.06971
Phe His	0.06157	0.31104	1.4969	0.58199	0.72595	0.99988	0.98929	-0.015528	0.46591	0.88892	0.94509	-0.081481	0.70101	0.99965	1.083	0.11502
Teasterone	0.086679	0.31104	1.6326	0.7072	0.8025	0.99988	0.97626	-0.034657	0.47509	0.88892	0.92668	-0.10985	0.99742	0.99965	1.0272	0.038781
DGTA(18:1/22:4(10	0.33263	0.44649	1.1064	0.14593	0.82416	0.99988	1.0317	0.045055	0.30426	0.88892	0.91873	-0.12229	0.91966	0.99965	1.0117	0.016851
Asp Asn Gly	0.097715	0.31104	1.3625	0.4463	0.97245	0.99988	1.0424	0.059855	0.58381	0.89321	0.96589	-0.050071	0.89872	0.99965	1.0139	0.019976
PA(22:2(13Z,16Z)/1	0.80302	0.84779	1.2162	0.28244	0.96215	0.99988	1.0399	0.056387	0.50503	0.89142	0.9466	-0.079179	0.86403	0.99965	1.0162	0.023192
PA(15:0/22:2(13Z,1	0.12029	0.31104	1.279	0.35501	0.84369	0.99988	1.0124	0.017781	0.77836	0.9495	0.99551	-0.006493	0.078443	0.99965	0.73307	-0.44798
PA(a-25:0/22:0)	0.086424	0.31104	1.4353	0.52131	0.75357	0.99988	0.97928	-0.030206	0.85861	0.97057	1.0192	0.027486	0.55604	0.99965	0.92181	-0.11746
Gln Gln Gln	0.051914	0.31104	1.4935	0.57866	0.90001	0.99988	1.0104	0.014918	0.58504	0.89321	0.95478	-0.06676	0.79113	0.99965	1.0478	0.0674
MGDG(18:3(9Z,12Z	0.10447	0.31104	1.2828	0.35924	0.93546	0.99988	1.0125	0.017973	0.89143	0.97567	1.0032	0.0046034	0.81857	0.99965	1.0309	0.043865
PG(20:4(5Z,8Z,11Z	0.085912	0.31104	1.2106	0.27569	0.80429	0.99988	0.99082	-0.013312	0.17231	0.88892	1.1037	0.14235	0.52272	0.99965	0.95137	-0.071924
Tyr Arg Phe	0.16002	0.31861	1.3856	0.47052	0.5594	0.99988	0.91849	-0.12267	0.87267	0.97567	0.97977	-0.029486	0.75258	0.99965	0.9482	-0.07674
CMP-N-glycoloyl-be	0.94411	0.95901	0.93207	-0.10149	0.58961	0.99988	0.82846	-0.2715	0.051936	0.88892	1.2402	0.31063	0.98597	0.99965	0.94108	-0.087618

Name	4. Metabolites that return to normal level				4. Metabolites that return to normal level				5. Does the dose make a difference?				Does the dose make a differen		
	Day 9				Day 9				Day 9				Day 9		
	Rad + ami_50/Rad				Rad + ami_200/Rad				ami_200/ami_50				Rad + ami_200/Rad + ami_50/Ra		
	p.value	FDR	Fold.Chang	log2.FC	p.value	FDR	Fold.Chang	log2.FC	p.value	FDR	Fold.Chang	log2.FC	p.value	FDR	f.value
PI(16:0/15:1(9Z))	0.0037066	0.065656	0.82352	-0.28012	0.076751	0.1222	0.86857	-0.20329	0.031832	0.40808	0.78975	-0.34054	0.017677	0.044426	4.9713
Cysteine-S-sulfate	0.020571	0.06848	0.79894	-0.32384	0.029894	0.066118	0.78576	-0.34783	0.095291	0.40808	0.78647	-0.34653	0.018334	0.045593	4.9168
Inosine 5'-tetraphos	0.028694	0.074985	0.66652	-0.58529	0.038759	0.076565	0.66248	-0.59404	0.19584	0.40808	0.67096	-0.5757	0.018492	0.045725	4.9039
PC(20:3(5Z,8Z,11Z)	0.030692	0.076427	0.8159	-0.29354	0.029468	0.06583	0.78463	-0.34992	0.072492	0.40808	0.77776	-0.3626	0.018613	0.045816	4.8942
PA(i-24:0/i-24:0)	0.034174	0.079804	0.77219	-0.37297	0.01566	0.057639	0.73839	-0.43754	0.1487	0.40808	0.72023	-0.47346	0.018788	0.046024	4.8803
PE-NMe2(22:6(4Z,7	0.011404	0.065656	0.75613	-0.4033	0.031506	0.068234	0.756	-0.40354	0.19801	0.40808	0.81224	-0.30002	0.018853	0.04611	4.8752
PE-NMe2(24:0/22:4	0.044788	0.091515	0.64513	-0.63233	0.021767	0.059154	0.55342	-0.85355	0.89254	0.9345	0.97112	-0.042277	0.018873	0.04611	4.8736
PS(P-16:0/20:1(11Z	0.031642	0.077392	0.80619	-0.31082	0.020547	0.05857	0.75805	-0.39964	0.13708	0.40808	0.77433	-0.36899	0.01889	0.04611	4.8723
Tyr Gln Phe	0.03422	0.079804	0.75172	-0.41174	0.025565	0.06154	0.70796	-0.49825	0.2368	0.42623	0.76438	-0.38764	0.019049	0.046359	4.8598
MG(P-18:0e/0:0/0:0)	0.065365	0.11624	0.79455	-0.3318	0.017712	0.057639	0.68312	-0.54979	0.43078	0.5726	0.87295	-0.19603	0.019104	0.046406	4.8555
TG(20:2(11Z,14Z)/1	0.14143	0.21068	0.76945	-0.37811	0.011296	0.057179	0.59691	-0.74442	0.14366	0.40808	0.65139	-0.61842	0.019108	0.046406	4.8552
Asn Pro His	0.037831	0.083258	0.74719	-0.42045	0.029722	0.065855	0.7066	-0.50103	0.21015	0.41045	0.76311	-0.39004	0.019222	0.046525	4.8464
Phe His	0.031771	0.077509	0.71504	-0.4839	0.028985	0.065374	0.67578	-0.56538	0.067991	0.40808	0.66089	-0.59752	0.019252	0.046533	4.844
Teasterone	0.033695	0.079438	0.61645	-0.69794	0.027756	0.063655	0.57126	-0.80779	0.074802	0.40808	0.59797	-0.74186	0.019452	0.04673	4.8287
DGTA(18:1/22:4(10	0.045751	0.092627	0.79856	-0.32452	0.020322	0.058504	0.73366	-0.44681	0.49427	0.62329	0.93247	-0.10087	0.019721	0.047216	4.8083
Asp Asn Gly	0.021232	0.068488	0.74897	-0.41701	0.031085	0.067779	0.72342	-0.46709	0.16315	0.40808	0.76501	-0.38644	0.019875	0.04742	4.7969
PA(22:2(13Z,16Z)/1	0.022099	0.069128	0.76906	-0.37883	0.023356	0.060095	0.72799	-0.45801	0.83347	0.89083	0.85497	-0.22605	0.01989	0.04742	4.7957
PA(15:0/22:2(13Z,1	0.0069998	0.065656	0.79904	-0.32365	0.03966	0.077598	0.79546	-0.33015	0.13193	0.40808	0.79156	-0.33723	0.019951	0.047468	4.7912
PA(a-25:0/22:0)	0.021762	0.068743	0.73447	-0.44522	0.048166	0.08778	0.7486	-0.41773	0.082996	0.40808	0.6823	-0.55151	0.020007	0.047516	4.787
Gln Gln Gln	0.039375	0.085027	0.72987	-0.45429	0.027557	0.063478	0.69686	-0.52105	0.069602	0.40808	0.67655	-0.56374	0.020122	0.047652	4.7786
MGDG(18:3(9Z,12Z	0.01539	0.065656	0.75644	-0.4027	0.042538	0.081156	0.75886	-0.39809	0.14522	0.40808	0.78935	-0.34127	0.020132	0.047652	4.7779
PG(20:4(5Z,8Z,11Z	0.0046816	0.065656	0.80748	-0.30849	0.17515	0.2351	0.89122	-0.16614	0.10923	0.40808	0.81847	-0.289	0.020279	0.047842	4.7671
Tyr Arg Phe	0.032728	0.078039	0.71674	-0.48048	0.029184	0.065625	0.70224	-0.50996	0.078343	0.40808	0.66288	-0.59319	0.021327	0.049455	4.6929
CMP-N-glycoloyl-be	0.17861	0.25184	1.1689	0.22517	0.017955	0.057639	1.4497	0.53579	0.59388	0.70309	0.88884	-0.17001	0.021417	0.049534	4.6867

To visualize the data on table 7, please zoom to 300%.

Supplementary Table 7

Amifostine protection effect in day 4 and day 9 for validated metabolites across all tissues

			Day 4		Day 9		Day 4												Day 9											
			50 mg	200 mg	50 mg	200 mg	Radiation				Amifostine 50 mg				Amifostine 200 mg				Radiation				Amifostine 50 mg				Amifostine 200 mg			
**Name**	**Organ/Tissue**	**Ionization Mode**	**Recovery Effect Indicator**				*p-value*	*FDR*	*Fold Change*	*Log2(FC)*	*p-value*	*FDR*	*Fold Change*	*Log2(FC)*	*p-value*	*FDR*	*Fold Change*	*Log2(FC)*	*p-value*	*FDR*	*Fold Change*	*Log2(FC)*	*p-value*	*FDR*	*Fold Change*	*Log2(FC)*	*p-value*	*FDR*	*Fold Change*	*Log2(FC)*
PG(18:0/20:4)	Bone marrow	NEG	-	Recovery	-	Recovery	4.87E-03	7.27E-03	0.6752	-0.5667	6.16E-01	1.00E+00	0.9206	-0.1194	4.54E-03	1.29E-02	1.6256	0.7009	1.49E-02	2.33E-02	0.5364	-0.8985	3.94E-01	7.80E-01	0.6772	-0.5623	7.96E-03	3.80E-02	1.9693	0.9777
PG(18:0/20:4)	Bone marrow	NEG	-	Recovery	-	Recovery	3.03E-03	4.68E-03	0.6968	-0.5212	3.34E-01	1.00E+00	0.8737	-0.1948	4.54E-03	1.29E-02	1.5097	0.5942	1.68E-02	2.59E-02	0.5660	-0.8212	2.84E-01	7.80E-01	0.6805	-0.5554	1.31E-02	4.99E-02	1.7519	0.8089
PA(18:0/18:2)	Bone marrow	NEG	Recovery	Recovery	-	Recovery	8.49E-12	2.52E-09	0.1243	-3.0078	1.02E-01	1.00E+00	1.2232	0.2907	1.11E-06	4.88E-04	9.7116	3.2797	2.92E-09	6.01E-07	0.1036	-3.2708	1.75E-01	7.80E-01	0.7289	-0.4563	5.74E-08	1.26E-05	6.3296	2.6621
PE(P-18:0/18:1)	Bone marrow	NEG	Recovery	Recovery	-	Recovery	8.06E-12	2.52E-09	0.1162	-3.1052	4.17E-01	1.00E+00	1.1837	0.2433	6.06E-07	4.88E-04	8.4898	3.0857	1.53E-09	4.54E-07	0.0984	-3.3458	2.57E-01	7.80E-01	0.7707	-0.3759	8.46E-09	3.23E-06	7.8643	2.9753
PC(O-16:0/20:4)	Bone marrow	NEG	Recovery	Recovery	-	Recovery	6.07E-10	6.43E-08	0.3139	-1.6715	2.67E-01	1.00E+00	1.1193	0.1626	1.59E-06	4.88E-04	3.4678	1.7940	1.18E-06	3.59E-05	0.2940	-1.7663	8.56E-02	7.80E-01	0.7106	-0.4930	2.31E-05	1.44E-03	2.4950	1.3190
PE(P-18:0/18:1)	Bone marrow	NEG	Recovery	Recovery	-	Recovery	4.85E-10	5.90E-08	0.1450	-2.7858	9.80E-01	1.00E+00	1.0220	0.0314	2.69E-06	5.58E-04	6.1377	2.6177	1.38E-09	4.54E-07	0.1009	-3.3087	2.25E-01	7.80E-01	0.7629	-0.3904	1.91E-07	3.16E-05	5.9599	2.5753
PC(18:3/18:3)	Bone marrow	NEG	-	Recovery	-	Recovery	1.11E-06	4.68E-06	0.3865	-1.3715	9.73E-01	1.00E+00	0.9980	-0.0029	7.62E-06	8.49E-04	2.6047	1.3811	2.20E-04	6.08E-04	0.3719	-1.4271	3.82E-01	7.80E-01	0.7561	-0.4033	2.30E-05	1.44E-03	3.5225	1.8166
PE-NMe(16:0/16:0)	Bone marrow	NEG	Recovery	Recovery	-	Recovery	2.05E-07	1.38E-06	0.2562	-1.9647	8.62E-01	1.00E+00	1.0287	0.0408	5.10E-06	7.08E-04	3.8023	1.9269	3.28E-08	5.16E-06	0.1793	-2.4799	3.20E-01	7.80E-01	0.8140	-0.2970	1.27E-06	1.14E-04	4.3402	2.1177
PE(P-18:0/18:1)	Bone marrow	NEG	Recovery	Recovery	-	Recovery	2.39E-10	3.56E-08	0.1453	-2.7828	4.01E-01	1.00E+00	1.1489	0.2003	3.12E-06	5.96E-04	6.7324	2.7511	9.71E-10	4.33E-07	0.1259	-2.9896	1.61E-01	7.80E-01	0.7680	-0.3809	7.86E-09	3.23E-06	6.1360	2.6173
PC(17:0/20:4)	Bone marrow	NEG	Recovery	Recovery	-	Recovery	1.10E-09	9.71E-08	0.2856	-1.8080	7.75E-01	1.00E+00	1.0446	0.0630	1.85E-06	4.88E-04	2.6038	1.3806	1.28E-07	1.23E-05	0.2423	-2.0453	1.74E-01	7.80E-01	0.7659	-0.3847	1.42E-06	1.22E-04	3.1836	1.6707
PE(18:1/18:1)	Bone marrow	NEG	Recovery	Recovery	-	Recovery	2.15E-09	1.40E-07	0.2414	-2.0505	1.45E-01	1.00E+00	1.1489	0.2002	5.75E-06	7.33E-04	3.3505	1.7444	6.06E-08	7.05E-06	0.2499	-2.0008	1.58E-01	7.80E-01	0.7787	-0.3610	4.85E-08	1.18E-05	3.7251	1.8973
DHPE	Bone marrow	NEG	Recovery	Recovery	-	Recovery	8.36E-01	8.53E-01	0.9900	-0.0145	9.42E-01	1.00E+00	1.0106	0.0152	1.14E-05	8.77E-04	2.2310	1.1577	4.81E-02	6.85E-02	0.7326	-0.4490	2.93E-01	7.80E-01	0.8253	-0.2770	2.16E-02	6.62E-02	1.3545	0.4378
PS(18:0/18:2)	Bone marrow	NEG	Recovery	Recovery	-	Recovery	6.68E-09	2.72E-07	0.1967	-2.3460	4.68E-01	1.00E+00	1.1870	0.2473	1.81E-06	4.88E-04	6.0044	2.5860	1.72E-09	4.59E-07	0.1416	-2.8205	1.41E-01	7.80E-01	0.7539	-0.4076	1.53E-07	2.73E-05	4.4090	2.1405
PC(18:0/22:6)	Bone marrow	NEG	Recovery	Recovery	Recovery	Recovery	1.76E-06	6.54E-06	2.0222	1.0159	6.72E-01	1.00E+00	0.9620	-0.0559	2.37E-05	1.09E-03	0.5359	-0.8999	1.91E-03	3.53E-03	2.2005	1.1378	6.61E-01	8.09E-01	0.8649	-0.2093	1.53E-02	5.32E-02	0.5720	-0.8058
PE-NMe(16:0/16:0)	Bone marrow	NEG	Recovery	Recovery	-	Recovery	4.67E-03	7.00E-03	0.6549	-0.6108	8.55E-01	1.00E+00	1.0035	0.0050	8.36E-05	1.53E-03	1.8895	0.9180	2.19E-03	3.98E-03	0.5923	-0.7557	2.02E-01	7.80E-01	0.7753	-0.3673	1.86E-02	6.02E-02	1.4279	0.5139
PI(18:0/0:0)	Bone marrow	NEG	-	Recovery	-	Recovery	4.17E-05	9.31E-05	0.5940	-0.7514	2.56E-02	1.00E+00	0.7404	-0.4336	3.01E-03	9.58E-03	1.5160	0.6003	7.40E-03	1.21E-02	0.4967	-1.0096	6.23E-01	7.90E-01	0.7170	-0.4799	1.38E-02	5.08E-02	1.8376	0.8778
C16-18:1 PE	Bone marrow	NEG	Recovery	Recovery	-	Recovery	1.21E-07	9.51E-07	0.2624	-1.9301	9.29E-01	1.00E+00	1.0248	0.0353	5.29E-06	7.08E-04	4.4401	2.1506	4.17E-08	5.87E-06	0.1817	-2.4605	2.66E-01	7.80E-01	0.7855	-0.3483	1.15E-06	1.10E-04	4.0586	2.0210
PI(18:0/18:0)	Bone marrow	NEG	-	Recovery	-	Recovery	8.80E-07	3.88E-06	0.3048	-1.7140	3.62E-01	1.00E+00	0.8516	-0.2318	2.15E-04	1.98E-03	2.5906	1.3733	3.59E-03	6.28E-03	0.4212	-1.2474	4.86E-01	7.80E-01	0.6739	-0.5695	5.46E-02	1.27E-01	1.5856	0.6650
PA(18:0/20:4)	Bone marrow	NEG	Recovery	Recovery	-	Recovery	6.74E-09	2.72E-07	0.3927	-1.3487	9.95E-01	1.00E+00	1.0358	0.0508	1.97E-06	4.88E-04	2.8592	1.5156	6.29E-06	6.18E-05	0.3202	-1.6428	1.12E-01	7.80E-01	0.7023	-0.5098	9.20E-05	4.40E-03	2.3449	1.2295
PC(16:0/18:2)	Bone marrow	NEG	-	Recovery	-	Recovery	3.46E-05	7.86E-05	0.5118	-0.9664	7.77E-01	1.00E+00	0.9831	-0.0247	9.21E-06	8.77E-04	2.2073	1.1423	1.48E-04	4.65E-04	0.4054	-1.3027	4.32E-01	7.80E-01	0.8032	-0.3163	7.19E-02	1.55E-01	1.5276	0.6113
DHAPC	Bone marrow	NEG	Recovery	Recovery	Recovery	Recovery	7.36E-08	7.69E-07	2.3087	1.2071	5.05E-01	1.00E+00	0.9426	-0.0852	1.18E-04	1.61E-03	0.5884	-0.7652	6.49E-05	2.68E-04	2.8511	1.5115	1.24E-01	7.80E-01	0.6948	-0.5253	7.70E-03	3.73E-02	0.5403	-0.8881
PC(18:0/18:1)	Bone marrow	NEG	-	Recovery	-	Recovery	6.51E-06	1.88E-05	0.4992	-1.0024	5.45E-01	1.00E+00	0.9361	-0.0953	1.59E-04	1.72E-03	1.8793	0.9102	6.64E-03	1.11E-02	0.5411	-0.8862	1.96E-01	7.80E-01	0.6433	-0.6365	4.61E-04	1.24E-02	2.5902	1.3730
PI(18:1/0:0)	Bone marrow	NEG	-	Recovery	-	Recovery	7.58E-06	2.15E-05	0.3714	-1.4288	2.21E-01	1.00E+00	0.8328	-0.2640	1.18E-03	5.01E-03	1.9660	0.9753	3.43E-03	6.05E-03	0.4508	-1.1494	4.55E-01	7.80E-01	0.6961	-0.5226	6.67E-03	3.49E-02	1.9209	0.9418
PC(18:0/18:2)	Bone marrow	NEG	-	Recovery	Recovery	Recovery	3.72E-07	2.08E-06	2.7407	1.4545	6.51E-01	1.00E+00	1.0243	0.0347	2.39E-04	2.07E-03	0.6002	-0.7364	3.23E-05	1.71E-04	3.4654	1.7930	5.15E-01	7.80E-01	0.8139	-0.2970	2.17E-03	3.00E-02	0.4518	-1.1461
PC(18:0/20:4)	Bone marrow	NEG	Recovery	Recovery	Recovery	Recovery	2.57E-07	1.61E-06	2.5666	1.3598	8.14E-01	1.00E+00	0.9715	-0.0417	1.67E-04	1.75E-03	0.5706	-0.8093	1.61E-04	4.88E-04	3.3839	1.7587	3.76E-01	7.80E-01	0.7452	-0.4242	3.81E-03	3.18E-02	0.4344	-1.2031
(Lys8,Lys9)-Neurotensin (8-13)	Bone marrow	NEG	Recovery	Recovery	-	Recovery	1.88E-05	4.63E-05	0.3745	-1.4171	7.47E-01	1.00E+00	1.0642	0.0898	5.69E-05	1.42E-03	3.4835	1.8005	6.42E-05	2.67E-04	0.3247	-1.6229	1.90E-01	7.80E-01	0.6808	-0.5548	1.23E-02	4.86E-02	1.7549	0.8114
PC(18:0/22:6)	Bone marrow	NEG	Recovery	Recovery	Recovery	Recovery	1.36E-05	3.51E-05	1.7287	0.7897	7.56E-01	1.00E+00	0.9753	-0.0361	9.76E-05	1.57E-03	0.6152	-0.7008	1.31E-02	2.06E-02	1.8797	0.9105	5.97E-01	7.83E-01	0.8298	-0.2692	9.49E-02	1.89E-01	0.6562	-0.6078
PC(18:0/22:6)	Bone marrow	NEG	-	Recovery	-	Recovery	1.66E-05	4.15E-05	0.3801	-1.3957	2.01E-01	1.00E+00	0.7813	-0.3561	1.35E-03	5.43E-03	2.1859	1.1282	3.17E-02	4.65E-02	0.5925	-0.7551	6.09E-01	7.85E-01	0.7150	-0.4839	1.91E-01	3.24E-01	1.2499	0.3218
PI(18:0/18:0)	Bone marrow	NEG	Recovery	Recovery	-	Recovery	1.35E-04	2.66E-04	0.4374	-1.1929	7.80E-01	1.00E+00	1.0396	0.0560	6.48E-05	1.42E-03	2.2667	1.1806	1.75E-05	1.14E-04	0.3822	-1.3875	9.22E-02	7.80E-01	0.7085	-0.4972	4.54E-02	1.10E-01	1.4447	0.5307
PE(O-16:0/18:1)	Bone marrow	NEG	-	Recovery	-	Recovery	1.61E-04	3.12E-04	0.4640	-1.1077	5.17E-01	1.00E+00	0.9217	-0.1176	1.32E-05	9.04E-04	3.0401	1.6041	1.59E-06	4.00E-05	0.2753	-1.8612	3.06E-01	7.80E-01	0.7813	-0.3561	1.11E-03	2.16E-02	2.1892	1.1304
PE(17:0/20:4)	Bone marrow	NEG	Recovery	Recovery	-	Recovery	6.42E-09	2.72E-07	0.2998	-1.7379	9.59E-01	1.00E+00	1.0444	0.0627	2.71E-06	5.58E-04	2.6084	1.3831	3.68E-07	2.40E-05	0.2357	-2.0848	9.76E-02	7.80E-01	0.6890	-0.5375	2.67E-06	2.10E-04	3.3179	1.7303
DHAPC	Bone marrow	NEG	Recovery	Recovery	Recovery	Recovery	1.19E-06	4.97E-06	2.3112	1.2086	7.80E-01	1.00E+00	0.9549	-0.0666	1.21E-03	5.07E-03	0.5987	-0.7402	2.07E-04	5.83E-04	2.6720	1.4179	2.15E-01	7.80E-01	0.7318	-0.4505	1.14E-02	4.62E-02	0.5399	-0.8893
16:0-18:1-PS	Bone marrow	NEG	Recovery	Recovery	-	Recovery	3.69E-05	8.31E-05	0.4515	-1.1472	4.53E-01	1.00E+00	1.1293	0.1754	8.07E-05	1.53E-03	2.7742	1.4721	2.23E-04	6.14E-04	0.4239	-1.2382	2.14E-01	7.80E-01	0.7235	-0.4669	4.38E-02	1.07E-01	1.4445	0.5306
PC(18:1/14:0)	Bone marrow	NEG	Recovery	Recovery	-	Recovery	2.51E-08	4.92E-07	0.3046	-1.7152	9.75E-01	1.00E+00	1.0772	0.1072	1.51E-05	9.59E-04	3.3226	1.7323	5.34E-06	5.67E-05	0.2747	-1.8642	1.56E-01	7.80E-01	0.6773	-0.5622	4.21E-03	3.18E-02	2.1147	1.0804
PE(16:0/18:2)	Bone marrow	NEG	Recovery	Recovery	-	Recovery	9.01E-02	1.12E-01	0.8562	-0.2240	7.63E-01	1.00E+00	1.0049	0.0071	2.01E-06	4.88E-04	2.1456	1.1014	5.09E-01	5.66E-01	0.9641	-0.0527	1.66E-01	7.80E-01	0.6980	-0.5186	5.56E-01	6.76E-01	1.0304	0.0433
PC(17:0/20:4)	Bone marrow	NEG	Recovery	-	Recovery	Recovery	9.59E-04	1.61E-03	1.5577	0.6394	5.83E-01	1.00E+00	0.9424	-0.0856	6.59E-03	1.67E-02	1.2996	0.3781	6.12E-03	1.03E-02	2.2423	1.1650	3.06E-01	7.80E-01	0.7168	-0.4804	6.65E-01	7.51E-01	0.8194	-0.2874
PE(18:0/18:2)	Bone marrow	NEG	Recovery	-	Recovery	Recovery	1.71E-02	2.35E-02	1.2739	0.3492	6.85E-01	1.00E+00	0.9728	-0.0397	3.38E-05	1.26E-03	1.5689	0.6498	1.92E-01	2.37E-01	1.3508	0.4338	1.82E-01	7.80E-01	0.7341	-0.4459	1.63E-01	2.86E-01	0.7293	-0.4555
PC(16:0/12:0)	Bone marrow	NEG	Recovery	Recovery	Recovery	Recovery	4.86E-05	1.07E-04	2.8956	1.5338	1.17E-01	1.00E+00	0.7138	-0.4864	9.61E-05	1.56E-03	0.3903	-1.3573	2.67E-04	6.92E-04	3.1521	1.6563	3.06E-01	7.80E-01	0.7653	-0.3860	1.24E-02	4.87E-02	0.5411	-0.8862
PE(22:6/22:6)	Bone marrow	NEG	Recovery	Recovery	Recovery	Recovery	2.64E-06	8.93E-06	2.2286	1.1561	5.51E-01	1.00E+00	0.9482	-0.0767	2.62E-04	2.16E-03	0.6124	-0.7074	5.10E-03	8.67E-03	1.9506	0.9640	2.35E-01	7.80E-01	0.7456	-0.4235	8.90E-03	3.99E-02	0.5490	-0.8650
PE(P-16:0/22:6)	Bone marrow	NEG	-	Recovery	-	-	1.01E-04	2.05E-04	0.6240	-0.6803	1.10E-01	1.00E+00	0.7893	-0.3413	6.54E-03	1.67E-02	1.5693	0.6502	4.24E-02	6.10E-02	0.6302	-0.6662	2.74E-01	7.80E-01	0.5432	-0.8804	5.31E-01	6.60E-01	0.9766	-0.0341
PC(16:0/18:1)	Bone marrow	NEG	-	Recovery	-	Recovery	1.22E-03	2.01E-03	0.4975	-1.0073	4.50E-01	1.00E+00	0.8363	-0.2579	2.79E-03	8.99E-03	1.9708	0.9788	5.56E-05	2.43E-04	0.4591	-1.1232	5.18E-02	7.80E-01	0.7140	-0.4861	4.87E-02	1.16E-01	1.4840	0.5695
PG(18:0/22:6)	Bone marrow	NEG	-	Recovery	-	Recovery	1.05E-01	1.29E-01	0.7979	-0.3258	3.48E-01	1.00E+00	0.8203	-0.2858	8.82E-03	2.09E-02	1.6575	0.7290	2.50E-01	3.00E-01	0.8513	-0.2323	5.59E-01	7.80E-01	0.6982	-0.5184	2.36E-01	3.76E-01	1.1612	0.2156
PG(21:0/22:6)	Bone marrow	NEG	Recovery	Recovery	-	Recovery	4.65E-05	1.03E-04	0.3537	-1.4994	6.08E-01	1.00E+00	1.1428	0.1925	9.41E-05	1.55E-03	2.9030	1.5376	3.49E-06	4.87E-05	0.2830	-1.8209	1.58E-01	7.80E-01	0.7203	-0.4733	1.89E-02	6.10E-02	1.7187	0.7813
PC(16:0/14:0)	Bone marrow	NEG	Recovery	Recovery	Recovery	-	8.21E-01	8.40E-01	0.9895	-0.0152	8.62E-01	1.00E+00	1.0077	0.0111	2.44E-05	1.09E-03	1.6866	0.7541	8.13E-01	8.44E-01	1.1486	0.1999	1.71E-01	7.80E-01	0.6831	-0.5498	3.78E-01	5.30E-01	1.0886	0.1224
PA(18:0/18:1)	Bone marrow	NEG	Recovery	Recovery	-	Recovery	3.43E-06	1.10E-05	0.3579	-1.4825	7.50E-01	1.00E+00	1.0827	0.1146	1.21E-06	4.88E-04	4.0986	2.0351	3.26E-06	4.85E-05	0.2469	-2.0183	2.99E-01	7.80E-01	0.7334	-0.4473	4.16E-04	1.19E-02	2.5552	1.3534
1,4-IP2	Bone marrow	NEG	-	Recovery	Recovery	Recovery	4.41E-06	1.36E-05	1.9737	0.9809	4.04E-01	1.00E+00	1.0942	0.1299	1.82E-03	6.60E-03	0.6888	-0.5378	2.13E-04	5.94E-04	2.7998	1.4853	3.29E-01	7.80E-01	0.7579	-0.3999	8.56E-03	3.95E-02	0.5016	-0.9953
Prostaglandin F3alpha	Bone marrow	NEG	-	Recovery	Recovery	Recovery	5.02E-07	2.59E-06	3.3664	1.7512	5.44E-01	1.00E+00	1.1183	0.1613	1.55E-03	5.92E-03	0.6005	-0.7358	1.39E-05	9.71E-05	4.4714	2.1607	5.53E-01	7.80E-01	0.8415	-0.2490	5.08E-03	3.24E-02	0.4535	-1.1410
GlCC(d18:1/12:0)	Bone marrow	NEG	-	Recovery	Recovery	Recovery	2.45E-07	1.56E-06	2.7538	1.4614	9.99E-01	1.00E+00	1.0153	0.0220	4.41E-04	2.73E-03	0.5940	-0.7515	1.20E-05	8.82E-05	3.4239	1.7756	4.27E-01	7.80E-01	0.8286	-0.2712	2.79E-03	3.01E-02	0.5021	-0.9939
PIP2(16:0/16:0)	Bone marrow	NEG	-	Recovery	Recovery	Recovery	5.09E-07	2.61E-06	2.8041	1.4876	6.29E-01	1.00E+00	1.0696	0.0971	2.08E-03	7.39E-03	0.6214	-0.6864	8.65E-06	7.37E-05	3.7506	1.9071	2.91E-01	7.80E-01	0.7672	-0.3823	6.09E-03	3.35E-02	0.4929	-1.0206
HPOTA	Bone marrow	NEG	-	Recovery	Recovery	Recovery	3.76E-06	1.19E-05	2.1261	1.0882	4.56E-01	1.00E+00	1.0745	0.1037	1.05E-02	2.37E-02	0.7147	-0.4847	5.87E-04	1.26E-03	2.4583	1.2977	3.65E-01	7.80E-01	0.7866	-0.3463	1.28E-02	4.97E-02	0.5641	-0.8260
PC(18:0/22:6)	Bone marrow	NEG	Recovery	Recovery	Recovery	Recovery	1.97E-09	1.32E-07	4.0120	2.0043	2.42E-01	1.00E+00	0.8836	-0.1786	1.65E-04	1.75E-03	0.5574	-0.8433	7.19E-06	6.58E-05	3.9934	1.9976	4.41E-01	7.80E-01	0.8054	-0.3122	1.16E-03	2.21E-02	0.4307	-1.2154
PE(18:1/0:0)	Bone marrow	NEG	-	Recovery	Recovery	Recovery	3.43E-05	7.80E-05	1.9282	0.9473	6.73E-01	1.00E+00	1.0383	0.0543	1.03E-02	2.35E-02	0.7322	-0.4497	1.53E-04	4.73E-04	2.0123	1.0088	5.23E-01	7.80E-01	0.8976	-0.1559	1.96E-02	6.23E-02	0.6909	-0.5335
PG(16:0/22:6)	Bone marrow	NEG	Recovery	Recovery	Recovery	Recovery	1.85E-09	1.30E-07	2.8229	1.4972	7.11E-01	1.00E+00	0.9726	-0.0401	5.51E-04	3.11E-03	0.6353	-0.6546	2.42E-05	1.42E-04	2.8012	1.4861	1.03E-01	7.80E-01	0.7282	-0.4576	1.99E-03	2.90E-02	0.5431	-0.8808
PC(22:0/0:0)	Bone marrow	NEG	Recovery	Recovery	Recovery	Recovery	3.95E-07	2.16E-06	1.9797	0.9853	8.79E-01	1.00E+00	0.9909	-0.0132	1.09E-03	4.73E-03	0.7289	-0.4562	2.46E-04	6.55E-04	2.4203	1.2752	2.62E-01	7.80E-01	0.7717	-0.3739	2.47E-02	7.23E-02	0.6228	-0.6831
PG(18:0/22:6)	Bone marrow	NEG	-	Recovery	-	Recovery	5.98E-01	6.30E-01	0.9607	-0.0579	3.71E-01	1.00E+00	0.8528	-0.2297	1.11E-02	2.47E-02	1.5477	0.6301	4.28E-01	4.85E-01	0.9431	-0.0846	6.05E-01	7.84E-01	0.7034	-0.5076	1.41E-01	2.57E-01	1.2931	0.3709
8(9)-EpETE	Bone marrow	NEG	-	Recovery	Recovery	Recovery	1.31E-07	1.00E-06	1.9461	0.9606	6.35E-01	1.00E+00	1.0521	0.0732	1.06E-03	4.67E-03	0.7353	-0.4436	7.09E-04	1.48E-03	2.2146	1.1471	4.10E-01	7.80E-01	0.8140	-0.2969	3.78E-02	9.61E-02	0.6421	-0.6392
PC(18:0/22:6)	Bone marrow	NEG	Recovery	Recovery	Recovery	Recovery	3.65E-06	1.16E-05	3.0724	1.6193	8.05E-01	1.00E+00	0.9501	-0.0738	1.72E-03	6.35E-03	0.4385	-1.1893	2.03E-02	3.09E-02	2.3898	1.2569	5.91E-01	7.82E-01	0.9717	-0.0414	4.93E-02	1.17E-01	0.4693	-1.0914
PE(18:0/22:6)	Bone marrow	NEG	Recovery	-	-	Recovery	2.50E-01	2.88E-01	1.0721	0.1004	2.84E-01	1.00E+00	0.8511	-0.2327	1.27E-03	5.21E-03	1.6239	0.6995	3.06E-01	3.59E-01	0.5456	-0.8742	2.33E-01	7.80E-01	0.6296	-0.6676	5.83E-02	1.31E-01	1.5079	0.5925
PC(p-18:0/18:1)	Bone marrow	NEG	-	Recovery	-	Recovery	1.22E-01	1.48E-01	0.8027	-0.3171	3.81E-01	1.00E+00	0.8469	-0.2398	2.33E-02	4.39E-02	1.4868	0.5722	4.79E-02	6.83E-02	0.6004	-0.7360	5.50E-01	7.80E-01	0.6502	-0.6211	5.74E-02	1.30E-01	1.5811	0.6609
PC(18:0/22:6)	Bone marrow	NEG	Recovery	Recovery	Recovery	Recovery	4.01E-09	2.27E-07	4.2653	2.0927	2.51E-01	1.00E+00	0.8789	-0.1863	2.14E-04	1.98E-03	0.5482	-0.8673	3.91E-06	4.93E-05	4.2502	2.0875	4.66E-01	7.80E-01	0.8172	-0.2912	7.84E-04	1.68E-02	0.4163	-1.2642
PS(18:0/18:2)	Bone marrow	NEG	Recovery	Recovery	-	Recovery	2.29E-04	4.29E-04	0.6461	-0.6303	4.79E-01	1.00E+00	1.0991	0.1364	3.20E-04	2.36E-03	1.8422	0.8815	6.88E-03	1.14E-02	0.5770	-0.7935	2.33E-01	7.80E-01	0.7350	-0.4442	2.57E-01	4.00E-01	1.1836	0.2432
PC(20:4)	Bone marrow	NEG	Recovery	Recovery	Recovery	Recovery	6.17E-05	1.31E-04	2.1916	1.1320	3.37E-01	1.00E+00	0.9197	-0.1207	2.48E-04	2.11E-03	0.6259	-0.6761	5.68E-04	1.23E-03	2.4326	1.2825	4.02E-01	7.80E-01	0.8060	-0.3112	2.88E-03	3.06E-02	0.4840	-1.0469
GP1	Bone marrow	NEG	-	Recovery	-	Recovery	3.38E-06	1.09E-05	0.4151	-1.2687	4.71E-01	1.00E+00	0.8962	-0.1581	4.27E-02	7.18E-02	1.3143	0.3943	1.26E-04	4.16E-04	0.3800	-1.3959	2.05E-01	7.80E-01	0.7398	-0.4349	1.67E-02	5.57E-02	1.5112	0.5957
LysoPC(18:0)	Bone marrow	NEG	-	Recovery	Recovery	Recovery	3.54E-06	1.13E-05	2.8764	1.5243	9.17E-01	1.00E+00	1.0245	0.0349	1.58E-03	6.00E-03	0.6315	-0.6632	4.76E-05	2.20E-04	3.1400	1.6508	5.69E-01	7.80E-01	0.8502	-0.2341	1.05E-02	4.41E-02	0.5332	-0.9074
PE(16:0/20:4)	Bone marrow	NEG	-	Recovery	-	Recovery	8.53E-01	8.68E-01	0.9563	-0.0644	5.72E-01	1.00E+00	0.9343	-0.0981	2.89E-03	9.27E-03	1.5096	0.5941	5.28E-02	7.47E-02	0.6768	-0.5631	2.98E-01	7.80E-01	0.7858	-0.3479	1.14E-01	2.18E-01	1.2540	0.3266
LysoPE(16:0)	Bone marrow	NEG	Recovery	Recovery	Recovery	Recovery	1.72E-06	6.42E-06	1.9100	0.9336	9.61E-01	1.00E+00	0.9968	-0.0046	2.35E-03	8.03E-03	0.7409	-0.4327	1.47E-03	2.80E-03	2.0633	1.0450	4.09E-01	7.80E-01	0.8203	-0.2858	8.62E-02	1.76E-01	0.6945	-0.5260
14(15)-EpEDE	Bone marrow	NEG	-	Recovery	Recovery	Recovery	6.69E-02	8.50E-02	0.6484	-0.6250	3.49E-01	1.00E+00	0.8200	-0.2863	2.97E-02	5.41E-02	1.6032	0.6810	6.94E-02	9.55E-02	3.0785	1.6222	2.61E-01	7.80E-01	0.4500	-1.1522	1.54E-01	2.74E-01	0.3984	-1.3276
3-Iodo-L-thyronine	Bone marrow	NEG	-	Recovery	Recovery	Recovery	1.25E-06	5.11E-06	2.1237	1.0866	3.70E-01	1.00E+00	1.1134	0.1550	1.24E-02	2.69E-02	0.7622	-0.3918	7.15E-04	1.49E-03	2.4917	1.3171	5.56E-01	7.80E-01	0.8296	-0.2696	3.33E-02	8.73E-02	0.5905	-0.7600
PG(18:0/22:6)	Bone marrow	NEG	Recovery	-	-	Recovery	9.68E-01	9.71E-01	1.0354	0.0502	2.78E-01	1.00E+00	0.8162	-0.2930	3.50E-02	6.13E-02	1.4221	0.5080	4.69E-01	5.27E-01	0.9615	-0.0566	5.73E-01	7.82E-01	0.7025	-0.5095	1.49E-01	2.68E-01	1.2654	0.3396
PA(O-16:0/0:0)	Bone marrow	NEG	-	Recovery	Recovery	Recovery	9.45E-06	2.58E-05	2.0256	1.0183	6.38E-01	1.00E+00	1.0509	0.0716	1.09E-02	2.44E-02	0.7453	-0.4242	9.45E-04	1.89E-03	2.4052	1.2662	7.27E-01	8.38E-01	0.8703	-0.2005	8.16E-02	1.70E-01	0.6446	-0.6336
LysoPE(16:0)	Bone marrow	NEG	-	Recovery	Recovery	Recovery	6.52E-06	1.89E-05	2.1574	1.1093	6.28E-01	1.00E+00	1.0705	0.0983	1.21E-02	2.65E-02	0.7047	-0.5050	4.30E-04	9.84E-04	2.8247	1.4981	4.24E-01	7.80E-01	0.7674	-0.3820	2.71E-01	4.18E-01	0.7299	-0.4541
PS(18:0/18:2)	Bone marrow	NEG	Recovery	Recovery	-	Recovery	7.44E-05	1.56E-04	0.3591	-1.4774	6.30E-01	1.00E+00	1.1446	0.1949	8.62E-04	4.08E-03	2.4512	1.2935	2.95E-05	1.62E-04	0.2977	-1.7483	1.76E-01	7.80E-01	0.6762	-0.5646	1.36E-02	5.08E-02	1.7082	0.7725
GPEtn(18:0p/20:4)	Bone marrow	NEG	Recovery	Recovery	-	Recovery	1.01E-06	4.32E-06	0.4355	-1.1991	7.26E-01	1.00E+00	1.0074	0.0107	2.25E-05	1.09E-03	1.8931	0.9208	4.34E-06	5.08E-05	0.3172	-1.6565	1.30E-01	7.80E-01	0.7082	-0.4978	1.61E-04	6.95E-03	2.3133	1.2099
LysoPC(18:1)	Bone marrow	NEG	-	Recovery	Recovery	Recovery	5.61E-08	6.58E-07	2.8579	1.5150	7.37E-01	1.00E+00	1.0490	0.0690	5.22E-03	1.43E-02	0.6745	-0.5681	2.97E-05	1.62E-04	3.5249	1.8176	5.63E-01	7.80E-01	0.8447	-0.2434	3.87E-03	3.18E-02	0.4873	-1.0371
GPEtn(18:0p/20:4)	Bone marrow	NEG	Recovery	Recovery	-	Recovery	1.23E-06	5.10E-06	0.4367	-1.1953	7.11E-01	1.00E+00	1.0069	0.0100	2.39E-05	1.09E-03	1.9152	0.9375	5.03E-06	5.56E-05	0.3149	-1.6670	1.21E-01	7.80E-01	0.6980	-0.5187	1.81E-04	7.50E-03	2.3194	1.2137
PA(18:0/20:4)	Bone marrow	NEG	-	Recovery	-	Recovery	7.59E-03	1.10E-02	0.7775	-0.3631	7.31E-01	1.00E+00	0.9843	-0.0228	3.62E-04	2.47E-03	1.5259	0.6097	4.62E-03	7.93E-03	0.5902	-0.7608	8.60E-02	7.80E-01	0.6610	-0.5973	7.57E-02	1.61E-01	1.2964	0.3746
PC(16:0/22:6)	Bone marrow	NEG	-	Recovery	Recovery	Recovery	4.68E-03	7.01E-03	1.3460	0.4287	8.12E-01	1.00E+00	1.0129	0.0185	1.61E-02	3.29E-02	0.7989	-0.3240	1.25E-01	1.63E-01	1.4119	0.4977	3.93E-01	7.80E-01	0.8058	-0.3116	9.71E-01	9.81E-01	0.9333	-0.0996
PC(18:0/22:6)	Bone marrow	NEG	-	Recovery	-	Recovery	1.39E-07	1.02E-06	0.3013	-1.7307	5.85E-01	1.00E+00	0.9210	-0.1187	1.22E-02	2.66E-02	1.5939	0.6726	4.44E-06	5.11E-05	0.2257	-2.1475	1.61E-01	7.80E-01	0.6843	-0.5474	5.00E-02	1.18E-01	1.5100	0.5945
17-Keto-docosahexaenoic acid	Bone marrow	NEG	-	Recovery	Recovery	Recovery	1.56E-04	3.04E-04	2.0514	1.0366	4.52E-01	1.00E+00	1.1197	0.1631	1.41E-02	2.99E-02	0.7217	-0.4706	7.28E-05	2.89E-04	2.5886	1.3722	4.52E-01	7.80E-01	0.8419	-0.2483	2.05E-02	6.42E-02	0.6302	-0.6661
LysoPC(15:0)	Bone marrow	NEG	-	Recovery	Recovery	Recovery	1.13E-05	3.01E-05	2.1870	1.1290	8.54E-01	1.00E+00	1.0515	0.0724	1.89E-03	6.83E-03	0.6670	-0.5843	6.90E-05	2.78E-04	2.9737	1.5722	4.83E-01	7.80E-01	0.8449	-0.2432	4.13E-03	3.18E-02	0.5082	-0.9767
PI(17:0/20:4)	Bone marrow	NEG	Recovery	Recovery	-	Recovery	1.54E-05	3.90E-05	0.4400	-1.1845	9.40E-01	1.00E+00	1.0337	0.0478	1.56E-03	5.92E-03	1.5786	0.6586	4.76E-05	2.20E-04	0.3517	-1.5075	1.79E-01	7.80E-01	0.7126	-0.4888	1.88E-01	3.21E-01	1.2126	0.2781
GPCho(18:0p/18:1)	Bone marrow	NEG	-	Recovery	-	-	2.68E-01	3.06E-01	0.8345	-0.2610	8.20E-01	1.00E+00	0.9251	-0.1124	2.04E-02	3.97E-02	1.5032	0.5880	5.86E-02	8.20E-02	0.7376	-0.4391	8.72E-02	7.80E-01	0.6239	-0.6807	8.66E-01	9.10E-01	0.9013	-0.1499
Ac-DY(2-malonyl)-VPML-NH2	Bone marrow	NEG	Recovery	Recovery	-	Recovery	1.88E-02	2.58E-02	0.7252	-0.4635	8.73E-01	1.00E+00	1.0155	0.0222	4.28E-04	2.70E-03	1.5505	0.6328	1.09E-02	1.74E-02	0.6563	-0.6076	7.14E-02	7.80E-01	0.6649	-0.5888	3.88E-01	5.39E-01	1.1032	0.1417
PC(16:0/22:6)	Bone marrow	NEG	Recovery	Recovery	Recovery	Recovery	8.04E-08	8.05E-07	2.3589	1.2381	3.03E-01	1.00E+00	0.9135	-0.1305	2.68E-03	8.79E-03	0.6997	-0.5151	5.68E-03	9.61E-03	2.0651	1.0462	2.94E-01	7.80E-01	0.7361	-0.4421	3.99E-02	1.01E-01	0.5861	-0.7708
PG(16:0/18:1)	Bone marrow	NEG	-	Recovery	-	Recovery	6.67E-02	8.47E-02	0.7601	-0.3957	1.41E-01	1.00E+00	0.7234	-0.4672	3.07E-02	5.55E-02	1.4188	0.5047	2.28E-02	3.45E-02	0.6202	-0.6891	1.48E-01	7.80E-01	0.5563	-0.8461	3.53E-01	5.05E-01	1.1622	0.2169
PC(16:0/22:6)	Bone marrow	NEG	Recovery	Recovery	Recovery	Recovery	8.80E-08	8.40E-07	2.3522	1.2340	2.84E-01	1.00E+00	0.9093	-0.1372	2.88E-03	9.25E-03	0.7000	-0.5145	6.87E-03	1.14E-02	2.0499	1.0356	3.06E-01	7.80E-01	0.7360	-0.4422	4.28E-02	1.06E-01	0.5851	-0.7732
PG(16:0/22:6)	Bone marrow	NEG	Recovery	Recovery	Recovery	Recovery	3.25E-09	1.93E-07	2.6352	1.3979	5.31E-01	1.00E+00	0.9469	-0.0787	1.29E-03	5.29E-03	0.6809	-0.5544	1.02E-04	3.62E-04	2.4441	1.2893	1.03E-01	7.80E-01	0.7230	-0.4680	5.55E-03	3.30E-02	0.5771	-0.7932
N-Stearoyltaurine	Bone marrow	NEG	Recovery	Recovery	-	Recovery	3.90E-04	7.00E-04	0.5852	-0.7731	9.60E-01	1.00E+00	1.0130	0.0187	8.72E-03	2.08E-02	1.5141	0.5984	1.05E-02	1.67E-02	0.6224	-0.6841	1.44E-01	7.80E-01	0.7116	-0.4909	6.07E-03	3.35E-02	1.7297	0.7905
GPEtn(18:0p/20:4)	Bone marrow	NEG	Recovery	-	-	Recovery	2.25E-01	2.60E-01	1.2697	0.3445	6.26E-01	1.00E+00	0.9221	-0.1170	1.56E-02	3.23E-02	1.4889	0.5742	8.65E-01	8.87E-01	0.9907	-0.0135	1.28E-01	7.80E-01	0.7406	-0.4333	2.30E-01	3.69E-01	1.4661	0.5520
PI(16:0/16:0)	Bone marrow	NEG	Recovery	Recovery	-	Recovery	2.62E-05	6.16E-05	0.4278	-1.2250	4.90E-01	1.00E+00	1.1695	0.2259	8.56E-04	4.07E-03	1.8989	0.9252	6.88E-05	2.78E-04	0.3488	-1.5196	1.86E-01	7.80E-01	0.6921	-0.5310	2.69E-03	3.01E-02	2.0376	1.0269
PC(18:0/20:4)	Bone marrow	NEG	-	Recovery	-	Recovery	3.61E-01	4.01E-01	0.8898	-0.1685	2.43E-01	1.00E+00	0.8071	-0.3092	1.38E-01	1.93E-01	1.3093	0.3888	2.78E-01	3.30E-01	0.8452	-0.2427	4.60E-01	7.80E-01	0.6740	-0.5692	1.10E-01	2.13E-01	1.3548	0.4381
PS(18:0/22:6)	Bone marrow	NEG	Recovery	Recovery	-	Recovery	4.90E-06	1.49E-05	0.4558	-1.1337	7.15E-01	1.00E+00	1.1253	0.1703	3.42E-04	2.43E-03	1.7865	0.8372	4.17E-05	2.03E-04	0.3541	-1.4976	1.70E-01	7.80E-01	0.7093	-0.4956	1.30E-02	4.99E-02	1.6558	0.7275
[Lys8]-ADH	Bone marrow	NEG	Recovery	Recovery	Recovery	Recovery	7.99E-06	2.24E-05	2.6209	1.3901	9.61E-02	1.00E+00	0.7474	-0.4200	1.81E-03	6.58E-03	0.4545	-1.1377	1.03E-01	1.37E-01	2.3254	1.2174	4.31E-01	7.80E-01	0.5898	-0.7617	2.23E-02	6.74E-02	0.3239	-1.6263
PG(18:0/20:4)	Bone marrow	NEG	Recovery	Recovery	Recovery	Recovery	3.99E-06	1.26E-05	2.4311	1.2816	3.30E-01	1.00E+00	0.8894	-0.1691	3.17E-04	2.36E-03	0.5799	-0.7861	2.96E-04	7.34E-04	2.5235	1.3354	3.01E-01	7.80E-01	0.7791	-0.3602	4.89E-03	3.19E-02	0.5333	-0.9071
PS(18:0/20:4)	Bone marrow	NEG	Recovery	Recovery	-	Recovery	1.53E-05	3.87E-05	0.4426	-1.1759	5.65E-01	1.00E+00	1.1397	0.1887	1.33E-03	5.39E-03	1.7722	0.8256	3.59E-05	1.83E-04	0.3595	-1.4760	1.80E-01	7.80E-01	0.7139	-0.4862	3.30E-03	3.18E-02	1.9364	0.9534
LysoPC(17:0/0:0)	Bone marrow	NEG	-	Recovery	Recovery	Recovery	7.55E-08	7.80E-07	2.5830	1.3690	6.45E-01	1.00E+00	1.0803	0.1114	2.61E-02	4.84E-02	0.7542	-0.4069	8.49E-05	3.18E-04	2.8583	1.5151	5.81E-01	7.82E-01	0.8666	-0.2065	1.31E-02	4.99E-02	0.5654	-0.8226
LysoPC(18:0)	Bone marrow	NEG	-	Recovery	Recovery	Recovery	1.13E-07	9.26E-07	2.7273	1.4474	9.60E-01	1.00E+00	1.0160	0.0229	7.06E-02	1.09E-01	0.7909	-0.3384	8.73E-06	7.38E-05	3.2857	1.7162	5.12E-01	7.80E-01	0.8506	-0.2335	1.95E-03	2.89E-02	0.4965	-1.0101
PE(18:0/22:6)	Bone marrow	NEG	Recovery	Recovery	Recovery	Recovery	1.75E-07	1.23E-06	2.2268	1.1550	3.88E-01	1.00E+00	0.9096	-0.1367	9.16E-04	4.20E-03	0.6564	-0.6073	1.09E-03	2.14E-03	1.9327	0.9507	7.47E-02	7.80E-01	0.7110	-0.4921	2.51E-03	3.01E-02	0.5634	-0.8277
PE(18:0/22:6)	Bone marrow	NEG	Recovery	Recovery	Recovery	Recovery	1.12E-06	4.68E-06	1.9793	0.9850	4.06E-01	1.00E+00	0.9143	-0.1292	4.49E-03	1.28E-02	0.7307	-0.4526	5.17E-03	8.78E-03	1.7194	0.7819	7.94E-02	7.80E-01	0.7114	-0.4912	9.76E-03	4.23E-02	0.6174	-0.6958
PC(18:1/16:0)	Bone marrow	NEG	Recovery	Recovery	Recovery	Recovery	1.52E-06	5.83E-06	2.2783	1.1880	2.67E-01	1.00E+00	0.8609	-0.2162	6.34E-03	1.63E-02	0.6961	-0.5226	1.47E-03	2.80E-03	2.2933	1.1974	4.27E-01	7.80E-01	0.7952	-0.3306	3.67E-01	5.18E-01	0.7754	-0.3670
PC(18:1/16:0)	Bone marrow	NEG	Recovery	Recovery	Recovery	Recovery	1.70E-06	6.36E-06	2.2351	1.1604	2.53E-01	1.00E+00	0.8556	-0.2249	6.23E-03	1.61E-02	0.6960	-0.5229	1.81E-03	3.36E-03	2.2365	1.1612	4.22E-01	7.80E-01	0.7952	-0.3305	4.26E-01	5.72E-01	0.7929	-0.3348
15(R),19(R)-HydroxyPGF1alpha	Bone marrow	NEG	Recovery	Recovery	-	Recovery	2.61E-06	8.87E-06	2.2044	1.1404	2.44E-01	1.00E+00	0.8698	-0.2012	6.32E-03	1.63E-02	0.7109	-0.4922	7.94E-03	1.29E-02	1.5679	0.6488	5.10E-01	7.80E-01	1.0641	0.0896	1.63E-01	2.86E-01	0.8095	-0.3049
PG(16:0/0:0)	Bone marrow	NEG	Recovery	Recovery	Recovery	Recovery	3.03E-06	9.99E-06	3.0240	1.5965	2.63E-02	1.00E+00	0.6536	-0.6135	1.55E-02	3.22E-02	0.6001	-0.7368	1.25E-02	1.97E-02	3.6019	1.8487	3.37E-01	7.80E-01	0.5457	-0.8738	2.79E-02	7.87E-02	0.3210	-1.6395
PC(14:0/18:0)	Bone marrow	NEG	Recovery	Recovery	Recovery	Recovery	8.51E-06	2.36E-05	2.2376	1.1620	3.17E-01	1.00E+00	0.8604	-0.2169	6.78E-03	1.71E-02	0.6621	-0.5949	7.17E-02	9.81E-02	1.5542	0.6362	2.70E-01	7.80E-01	0.7467	-0.4215	2.15E-01	3.50E-01	0.7250	-0.4640
TGT	Bone marrow	NEG	-	Recovery	Recovery	Recovery	1.25E-05	3.28E-05	2.0219	1.0157	8.48E-01	1.00E+00	1.0397	0.0562	1.63E-02	3.33E-02	0.7452	-0.4242	1.62E-04	4.90E-04	2.5931	1.3747	3.56E-01	7.80E-01	0.7936	-0.3335	1.41E-02	5.14E-02	0.5789	-0.7887
PI(17:1/0:0)	Bone marrow	NEG	Recovery	Recovery	Recovery	Recovery	5.62E-05	1.21E-04	3.0864	1.6259	5.09E-02	1.00E+00	0.6310	-0.6642	6.13E-03	1.59E-02	0.4794	-1.0608	4.29E-02	6.17E-02	3.5607	1.8321	8.38E-01	9.04E-01	0.6732	-0.5708	5.63E-03	3.30E-02	0.1851	-2.4334
PE(22:6)	Bone marrow	NEG	Recovery	Recovery	Recovery	Recovery	1.16E-04	2.32E-04	1.8463	0.8847	8.48E-02	1.00E+00	0.7748	-0.3682	7.64E-02	1.17E-01	0.7518	-0.4115	6.97E-02	9.57E-02	2.4568	1.2968	4.94E-01	7.80E-01	0.6234	-0.6818	2.15E-01	3.50E-01	0.5073	-0.9790
PS(16:0/20:4)	Bone marrow	NEG	Recovery	Recovery	-	Recovery	1.26E-04	2.51E-04	0.5190	-0.9461	7.48E-01	1.00E+00	1.0839	0.1162	6.82E-03	1.71E-02	1.5578	0.6395	6.73E-05	2.75E-04	0.3344	-1.5805	3.07E-01	7.80E-01	0.7437	-0.4272	7.71E-04	1.68E-02	2.4764	1.3082
PI(16:0/0:0)	Bone marrow	NEG	-	Recovery	-	Recovery	3.62E-04	6.52E-04	0.6209	-0.6877	3.94E-01	1.00E+00	0.9079	-0.1394	1.71E-01	2.32E-01	1.1694	0.2257	3.03E-02	4.46E-02	0.6350	-0.6551	5.01E-01	7.80E-01	0.7445	-0.4256	5.64E-03	3.30E-02	1.8783	0.9094
PI(16:0/0:0)	Bone marrow	NEG	Recovery	Recovery	Recovery	Recovery	7.84E-04	1.33E-03	1.7053	0.7701	4.79E-02	1.00E+00	0.7478	-0.4193	8.51E-02	1.28E-01	0.7628	-0.3906	2.57E-01	3.08E-01	2.0469	1.0334	1.78E-01	7.80E-01	0.4499	-1.1525	4.56E-02	1.10E-01	0.3393	-1.5594
Ptd(S)Ins-(3,4)-P2 (1,2-dioctanoyl)	Bone marrow	NEG	Recovery	Recovery	Recovery	Recovery	1.77E-03	2.84E-03	1.3085	0.3879	6.86E-01	1.00E+00	0.9812	-0.0274	9.04E-03	2.13E-02	0.7567	-0.4022	8.04E-01	8.36E-01	1.1032	0.1418	1.00E-01	7.80E-01	0.7091	-0.4959	9.69E-01	9.80E-01	0.9429	-0.0849
PC(16:1/16:1)	Bone marrow	NEG	Recovery	Recovery	Recovery	Recovery	1.92E-03	3.06E-03	1.3239	0.4048	6.63E-01	1.00E+00	0.9751	-0.0364	6.72E-03	1.70E-02	0.7401	-0.4343	7.03E-01	7.44E-01	1.1269	0.1724	8.68E-02	7.80E-01	0.7000	-0.5145	9.45E-01	9.61E-01	0.9378	-0.0927
PI(18:0/20:4)	Bone marrow	NEG	Recovery	Recovery	-	Recovery	2.15E-03	3.40E-03	0.5631	-0.8286	9.97E-01	1.00E+00	1.0824	0.1142	8.08E-04	3.95E-03	1.6803	0.7488	2.24E-05	1.35E-04	0.3837	-1.3820	7.58E-02	7.80E-01	0.6821	-0.5520	1.00E-01	1.97E-01	1.3742	0.4586
C-8 Ceramide-1-phosphate	Bone marrow	NEG	Recovery	Recovery	Recovery	Recovery	3.05E-03	4.70E-03	1.9478	0.9618	9.17E-01	1.00E+00	0.9950	-0.0073	3.39E-02	5.98E-02	0.6459	-0.6305	1.15E-03	2.23E-03	2.1387	1.0967	4.47E-01	7.80E-01	0.8855	-0.1754	3.63E-03	3.18E-02	0.5794	-0.7874
Folic acid	Bone marrow	NEG	Recovery	Recovery	-	Recovery	1.56E-02	2.17E-02	1.8035	0.8508	7.83E-01	1.00E+00	0.9388	-0.0911	3.01E-01	3.77E-01	0.7622	-0.3918	1.82E-02	2.80E-02	1.7980	0.8464	4.53E-01	7.80E-01	1.0958	0.1320	2.01E-01	3.36E-01	0.7089	-0.4964
18:1 BMP (S,R)	Bone marrow	NEG	Recovery	Recovery	-	Recovery	2.09E-02	2.83E-02	0.6423	-0.6387	7.72E-01	1.00E+00	1.0623	0.0872	1.16E-02	2.55E-02	1.6066	0.6840	5.44E-02	7.66E-02	0.6906	-0.5341	7.21E-02	7.80E-01	0.5999	-0.7372	6.26E-01	7.25E-01	1.0567	0.0796
PE(16:0/20:4)	Bone marrow	NEG	Recovery	Recovery	-	Recovery	3.24E-02	4.29E-02	0.8467	-0.2401	7.70E-01	1.00E+00	1.0008	0.0011	1.49E-03	5.79E-03	1.3450	0.4276	3.70E-02	5.37E-02	0.6987	-0.5173	2.01E-01	7.80E-01	0.7141	-0.4859	1.45E-02	5.20E-02	1.5767	0.6569
R-1 Methanandamide Phosphate	Bone marrow	NEG	Recovery	Recovery	-	Recovery	3.33E-02	4.40E-02	1.8792	0.9101	7.43E-01	1.00E+00	0.9120	-0.1329	4.33E-01	5.13E-01	0.7637	-0.3888	2.29E-02	3.47E-02	1.8593	0.8948	3.96E-01	7.80E-01	1.1504	0.2022	2.42E-01	3.82E-01	0.7119	-0.4903
GPE(P-18:0/20:4)	Bone marrow	NEG	-	Recovery	-	Recovery	8.47E-02	1.06E-01	0.8803	-0.1840	6.04E-01	1.00E+00	0.9749	-0.0367	1.20E-03	5.05E-03	1.3545	0.4378	2.13E-02	3.24E-02	0.6823	-0.5515	6.55E-02	7.80E-01	0.6617	-0.5957	8.59E-02	1.76E-01	1.2731	0.3484
PE(16:0/20:4)	Bone marrow	NEG	-	Recovery	-	Recovery	1.20E-01	1.45E-01	0.8882	-0.1710	7.87E-01	1.00E+00	0.9993	-0.0010	1.51E-03	5.81E-03	1.3389	0.4211	6.54E-02	9.05E-02	0.7481	-0.4187	1.74E-01	7.80E-01	0.7102	-0.4938	1.85E-02	6.01E-02	1.5215	0.6055
Dopamine	Bone marrow	POS	-	Recovery	-	Recovery	4.70E-07	4.48E-06	0.2194	-2.1886	9.28E-01	1.00E+00	0.9629	-0.0546	5.44E-05	2.51E-03	4.8136	2.2671	1.17E-07	2.11E-05	0.1330	-2.9100	1.93E-01	7.28E-01	0.6881	-0.5393	3.88E-05	2.02E-03	4.1236	2.0439
L-Glutathione	Bone marrow	POS	-	Recovery	-	Recovery	4.98E-11	1.22E-08	0.0235	-5.4114	9.84E-01	1.00E+00	0.9931	-0.0101	6.24E-05	2.62E-03	12.5460	3.6492	5.59E-10	9.61E-07	0.0276	-5.1795	1.50E-01	7.28E-01	0.6686	-0.5807	1.59E-05	1.11E-03	7.3254	2.8729
AMP	Bone marrow	POS	-	Recovery	-	Recovery	3.79E-08	1.43E-06	0.0604	-4.0488	1.87E-01	1.00E+00	0.8275	-0.2731	3.40E-04	6.29E-03	4.8558	2.2797	2.56E-07	3.26E-05	0.1029	-3.2812	7.72E-02	7.28E-01	0.6591	-0.6015	1.34E-04	5.10E-03	3.7406	1.9033
Acetyl-carnitine	Bone marrow	POS	Recovery	Recovery	-	Recovery	6.03E-13	6.92E-10	0.0229	-5.4490	4.28E-01	1.00E+00	1.1133	0.1549	5.39E-05	2.51E-03	9.4815	3.2451	1.36E-10	4.68E-07	0.0247	-5.3393	3.96E-01	7.28E-01	0.7448	-0.4250	1.99E-07	6.14E-05	18.4610	4.2064
PC(17:0/0:0)	Bone marrow	POS	Recovery	Recovery	Recovery	Recovery	4.81E-06	1.78E-05	2.2864	1.1931	1.23E-01	1.00E+00	0.8291	-0.2704	9.88E-04	9.76E-03	0.6299	-0.6668	1.40E-03	3.16E-03	2.5019	1.3230	4.51E-01	7.28E-01	0.7668	-0.3832	6.20E-02	1.32E-01	0.5951	-0.7489
1-SPP	Bone marrow	POS	Recovery	Recovery	-	Recovery	3.12E-04	5.20E-04	0.6094	-0.7145	9.97E-01	1.00E+00	1.0387	0.0547	5.23E-04	7.14E-03	1.6568	0.7284	4.91E-04	1.81E-03	0.4692	-1.0919	1.14E-01	7.28E-01	0.7163	-0.4814	1.08E-02	9.65E-02	1.5822	0.6619
D-Glucosyl-beta1-1'-D-erythro-sphingosine	Bone marrow	POS	Recovery	Recovery	Recovery	Recovery	6.53E-07	5.12E-06	1.9815	0.9866	7.49E-01	1.00E+00	0.9749	-0.0368	2.30E-03	1.43E-02	0.7197	-0.4746	6.66E-04	2.11E-03	2.4983	1.3209	4.53E-01	7.28E-01	0.8108	-0.3026	2.43E-02	9.67E-02	0.5880	-0.7661
Cytidine 5'-DP	Bone marrow	POS	Recovery	Recovery	Recovery	Recovery	3.41E-08	1.32E-06	2.2300	1.1571	8.60E-02	1.00E+00	0.8884	-0.1707	1.29E-04	3.78E-03	0.6684	-0.5813	5.57E-05	7.00E-04	2.0796	1.0563	6.95E-01	7.93E-01	0.9280	-0.1078	1.22E-02	9.65E-02	0.6941	-0.5267
20-OH-LTB4	Bone marrow	POS	-	Recovery	Recovery	Recovery	1.04E-07	2.51E-06	2.0862	1.0609	3.94E-01	1.00E+00	1.2478	0.3194	2.98E-04	5.99E-03	0.7110	-0.4920	3.24E-04	1.44E-03	2.2592	1.1758	2.63E-01	7.28E-01	0.7959	-0.3294	1.46E-02	9.65E-02	0.6209	-0.6875
PC(18:1/16:0)	Bone marrow	POS	Recovery	Recovery	Recovery	Recovery	1.36E-05	3.65E-05	1.9292	0.9480	4.30E-01	1.00E+00	0.9145	-0.1290	7.31E-03	2.18E-02	0.7216	-0.4708	4.07E-03	6.84E-03	1.9431	0.9584	4.25E-01	7.28E-01	0.8081	-0.3074	1.62E-02	9.65E-02	0.5735	-0.8022
3beta-Androstanediol	Bone marrow	POS	-	Recovery	Recovery	Recovery	2.44E-05	5.66E-05	2.0128	1.0092	9.34E-01	1.00E+00	1.0093	0.0133	1.50E-02	3.18E-02	0.7205	-0.4730	3.08E-03	5.41E-03	2.4102	1.2691	6.05E-01	7.40E-01	0.8162	-0.2931	7.96E-02	1.52E-01	0.6187	-0.6927
PE(18:1/20:3)	Bone marrow	POS	Recovery	Recovery	Recovery	Recovery	2.75E-06	1.21E-05	2.6060	1.3819	5.11E-01	1.00E+00	0.9369	-0.0940	3.56E-03	1.65E-02	0.6724	-0.5727	1.01E-03	2.63E-03	3.0592	1.6132	7.08E-01	8.00E-01	0.8080	-0.3076	2.77E-02	9.75E-02	0.5012	-0.9965
LysoPC(16:0)	Bone marrow	POS	Recovery	Recovery	Recovery	Recovery	1.13E-05	3.21E-05	2.1230	1.0861	6.09E-01	1.00E+00	0.9313	-0.1027	1.17E-02	2.79E-02	0.7093	-0.4955	1.56E-03	3.35E-03	2.4303	1.2812	4.63E-01	7.28E-01	0.7859	-0.3476	3.63E-02	1.04E-01	0.5759	-0.7961
Oleoyl-L-carnitine	Bone marrow	POS	-	Recovery	Recovery	Recovery	3.27E-02	4.13E-02	1.4105	0.4962	9.27E-01	1.00E+00	1.0110	0.0158	1.92E-02	3.68E-02	0.7502	-0.4147	2.10E-02	3.06E-02	1.8245	0.8675	3.76E-01	7.28E-01	0.8023	-0.3177	4.70E-02	1.16E-01	0.6335	-0.6586
PE(18:1/20:3)	Bone marrow	POS	Recovery	Recovery	Recovery	Recovery	1.56E-06	8.59E-06	2.1424	1.0993	5.14E-01	1.00E+00	0.9366	-0.0945	8.46E-03	2.34E-02	0.7216	-0.4707	1.23E-03	2.96E-03	2.4339	1.2833	4.32E-01	7.28E-01	0.7889	-0.3421	4.82E-02	1.17E-01	0.6071	-0.7200
Nutriacholic acid	Bone marrow	POS	Recovery	Recovery	Recovery	Recovery	4.66E-06	1.76E-05	1.7637	0.8186	5.85E-01	1.00E+00	0.9533	-0.0691	1.34E-02	2.99E-02	0.7760	-0.3659	1.13E-03	2.82E-03	2.3541	1.2352	1.67E-01	7.28E-01	0.7259	-0.4621	2.02E-02	9.65E-02	0.5888	-0.7642
LysoPC(P-18:0)	Bone marrow	POS	Recovery	Recovery	Recovery	Recovery	5.22E-06	1.88E-05	2.4933	1.3180	3.95E-02	1.00E+00	0.7570	-0.4017	1.46E-03	1.20E-02	0.5978	-0.7423	9.68E-04	2.57E-03	2.3150	1.2110	4.01E-01	7.28E-01	0.8105	-0.3032	1.19E-01	2.04E-01	0.7003	-0.5140
Oleoyl-L-carnitine	Bone marrow	POS	Recovery	Recovery	Recovery	Recovery	1.17E-05	3.30E-05	2.1181	1.0828	7.27E-01	1.00E+00	0.9702	-0.0437	8.42E-03	2.34E-02	0.7193	-0.4753	1.25E-03	3.00E-03	2.4391	1.2864	4.10E-01	7.28E-01	0.7806	-0.3573	4.92E-02	1.18E-01	0.6070	-0.7202
Unoprostone isopropyl ester	Bone marrow	POS	Recovery	Recovery	Recovery	Recovery	1.22E-05	3.38E-05	2.5828	1.3689	3.17E-01	1.00E+00	0.8858	-0.1750	4.55E-01	5.12E-01	0.9687	-0.0459	2.73E-04	1.31E-03	2.6347	1.3976	5.53E-01	7.29E-01	0.8391	-0.2531	6.17E-02	1.31E-01	0.6160	-0.6991
Palmitoylcarnitine	Bone marrow	POS	-	Recovery	Recovery	Recovery	1.72E-05	4.38E-05	2.1209	1.0847	8.07E-01	1.00E+00	1.1057	0.1449	1.33E-02	2.98E-02	0.7326	-0.4488	4.80E-02	6.60E-02	1.8314	0.8730	5.78E-01	7.35E-01	0.7814	-0.3559	1.02E-01	1.80E-01	0.6066	-0.7211
Ursocholic acid	Bone marrow	POS	Recovery	Recovery	Recovery	Recovery	1.93E-05	4.76E-05	1.9715	0.9793	7.64E-01	1.00E+00	0.9594	-0.0599	3.44E-02	5.56E-02	0.7735	-0.3706	3.13E-03	5.48E-03	2.2330	1.1590	3.52E-01	7.28E-01	0.7605	-0.3949	8.10E-02	1.54E-01	0.6340	-0.6575
Oleoyl-L-carnitine	Bone marrow	POS	Recovery	Recovery	Recovery	Recovery	2.37E-05	5.54E-05	2.0393	1.0281	7.61E-01	1.00E+00	0.9797	-0.0295	6.85E-03	2.12E-02	0.7207	-0.4725	7.68E-04	2.28E-03	2.4555	1.2960	4.31E-01	7.28E-01	0.8082	-0.3072	4.19E-02	1.11E-01	0.6228	-0.6831
LH	Bone marrow	POS	-	Recovery	Recovery	Recovery	3.03E-05	6.65E-05	1.8837	0.9136	9.50E-01	1.00E+00	1.0198	0.0283	4.65E-02	6.97E-02	0.7879	-0.3439	2.13E-03	4.09E-03	2.6149	1.3868	4.21E-01	7.28E-01	0.7502	-0.4147	5.45E-02	1.24E-01	0.5722	-0.8055
Oleoyl-L-carnitine	Bone marrow	POS	Recovery	Recovery	Recovery	Recovery	3.52E-05	7.53E-05	2.2684	1.1817	6.70E-01	1.00E+00	0.9598	-0.0591	1.77E-02	3.52E-02	0.7335	-0.4471	8.79E-04	2.45E-03	2.6765	1.4203	5.03E-01	7.28E-01	0.7832	-0.3526	5.55E-02	1.24E-01	0.5827	-0.7792
Butanoyl PAF	Bone marrow	POS	Recovery	Recovery	Recovery	Recovery	7.87E-05	1.48E-04	2.1381	1.0964	8.50E-01	1.00E+00	0.9777	-0.0326	8.64E-03	2.36E-02	0.6602	-0.5990	6.32E-05	7.39E-04	2.3200	1.2141	2.25E-01	7.28E-01	0.8338	-0.2622	6.91E-04	1.90E-02	0.5893	-0.7630
[Arg8] alpha-Neo-Endorphin (1-8)	Bone marrow	POS	Recovery	Recovery	Recovery	Recovery	8.03E-05	1.51E-04	1.8986	0.9249	2.49E-01	1.00E+00	0.9024	-0.1483	6.12E-02	8.75E-02	0.8363	-0.2579	9.27E-04	2.52E-03	2.3053	1.2049	1.73E-01	7.28E-01	0.7259	-0.4622	8.96E-03	9.65E-02	0.5251	-0.9293
Linoleoylcarnitine	Bone marrow	POS	-	Recovery	Recovery	Recovery	3.19E-04	5.29E-04	1.5961	0.6745	8.87E-01	1.00E+00	1.0176	0.0252	4.07E-02	6.34E-02	0.7900	-0.3401	3.40E-03	5.87E-03	2.1625	1.1127	3.75E-01	7.28E-01	0.7877	-0.3442	6.93E-02	1.40E-01	0.6494	-0.6229
Palmitoylcarnitine	Bone marrow	POS	Recovery	Recovery	Recovery	Recovery	5.59E-04	8.85E-04	5.0210	2.3280	1.38E-01	1.00E+00	0.5535	-0.8533	1.22E-02	2.85E-02	0.3912	-1.3540	1.35E-03	3.09E-03	4.6577	2.2196	7.40E-01	8.26E-01	0.8994	-0.1530	6.63E-02	1.37E-01	0.3958	-1.3370
Inosine	Bone marrow	POS	Recovery	Recovery	Recovery	Recovery	9.78E-03	1.31E-02	0.4922	-1.0226	5.58E-01	1.00E+00	1.8314	0.8730	1.79E-03	1.31E-02	2.6393	1.4001	4.67E-02	6.44E-02	0.6168	-0.6970	3.04E-01	7.28E-01	1.2377	0.3077	1.81E-02	9.65E-02	1.6430	0.7163
LysoPE(16:0)	Bone marrow	POS	-	Recovery	Recovery	Recovery	2.92E-02	3.71E-02	1.7157	0.7788	4.25E-01	1.00E+00	1.1662	0.2218	3.35E-01	3.90E-01	0.7902	-0.3397	2.88E-03	5.13E-03	2.6446	1.4031	2.35E-01	7.28E-01	0.7147	-0.4847	6.97E-02	1.40E-01	0.5906	-0.7598
CTR	Bone marrow	POS	-	Recovery	-	Recovery	3.45E-02	4.33E-02	0.7047	-0.5049	1.71E-01	1.00E+00	0.8506	-0.2334	2.80E-01	3.33E-01	1.0975	0.1342	1.75E-01	2.18E-01	0.8265	-0.2749	3.23E-01	7.28E-01	0.7680	-0.3809	4.89E-01	6.21E-01	1.0791	0.1098
Oleoyl-L-carnitine	Bone marrow	POS	Recovery	-	Recovery	Recovery	9.09E-01	9.19E-01	0.9626	-0.0550	2.56E-01	1.00E+00	1.5791	0.6591	6.75E-03	2.11E-02	0.7341	-0.4459	1.29E-02	1.95E-02	3.2070	1.6812	5.04E-02	7.28E-01	0.4001	-1.3216	5.48E-02	1.24E-01	0.4149	-1.2691
LysoPC(14:0)	Bone marrow	POS	-	-	-	-	1.82E-01	2.09E-01	0.8417	-0.2487	1.22E-01	1.00E+00	0.8347	-0.2607	7.72E-01	8.11E-01	0.9612	-0.0571	5.58E-01	6.14E-01	0.9908	-0.0133	1.27E-01	7.28E-01	0.6002	-0.7364	7.22E-01	8.09E-01	0.9801	-0.0289
LysoPE(18:0)	Jejumun	NEG	Recovery	Recovery	Recovery	Recovery	1.25E-06	6.34E-04	0.1557	-2.6831	6.87E-01	9.94E-01	1.6047	0.6823	5.91E-07	2.12E-03	5.6180	2.4901	4.44E-01	1.00E+00	1.1177	0.1605	6.19E-01	8.27E-01	0.9048	-0.1444	8.33E-01	9.29E-01	0.8124	-0.2998
LysoPE(18:0)	Jejumun	NEG	Recovery	Recovery	-	Recovery	1.34E-06	6.34E-04	0.1140	-3.1333	1.90E-01	6.96E-01	1.6164	0.6928	2.10E-04	1.42E-02	2.7529	1.4610	1.24E-01	1.00E+00	0.7586	-0.3987	9.21E-01	9.74E-01	0.9487	-0.0760	2.34E-01	6.90E-01	1.2999	0.3784
PI(20:4/0:0)	Jejumun	NEG	Recovery	Recovery	Recovery	-	1.42E-06	6.34E-04	0.2431	-2.0405	5.75E-02	5.73E-01	1.6031	0.6809	6.11E-06	6.95E-03	2.5674	1.3603	1.90E-02	7.33E-01	1.8530	0.8899	5.29E-01	7.70E-01	0.9188	-0.1222	6.92E-01	8.67E-01	1.0428	0.0604
PE(O-18:0/0:0)	Jejumun	NEG	Recovery	Recovery	Recovery	Recovery	6.52E-05	3.34E-03	0.3581	-1.4817	1.34E-01	6.39E-01	1.5545	0.6364	8.72E-04	3.64E-02	2.0256	1.0183	5.18E-01	1.00E+00	1.1867	0.2469	3.15E-01	6.25E-01	0.8037	-0.3154	5.77E-01	8.08E-01	0.8714	-0.1986
L-Cysteine-glutathione disulfide	Jejumun	NEG	Recovery	Recovery	Recovery	-	7.56E-04	1.50E-02	0.2456	-2.0254	2.86E-01	8.06E-01	1.7396	0.7987	2.33E-01	6.53E-01	2.0334	1.0239	9.24E-01	1.00E+00	1.2573	0.3303	4.69E-01	7.27E-01	0.7397	-0.4350	4.87E-01	7.69E-01	1.5067	0.5914
TUDCA	Jejumun	NEG	Recovery	Recovery	Recovery	-	3.64E-03	3.90E-02	0.4174	-1.2604	4.95E-01	9.69E-01	1.1268	0.1722	7.33E-01	9.25E-01	1.0293	0.0416	9.42E-01	1.00E+00	1.0140	0.0200	2.22E-01	5.41E-01	0.6951	-0.5247	8.08E-01	9.17E-01	1.1462	0.1969
1a,1b-dihomo PGF2alpha	Jejumun	NEG	Recovery	Recovery	-	Recovery	5.47E-03	4.92E-02	0.4129	-1.2762	9.84E-01	1.00E+00	1.0782	0.1086	1.96E-01	6.14E-01	1.5818	0.6616	1.47E-02	6.68E-01	0.3785	-1.4018	4.82E-01	7.35E-01	0.7614	-0.3932	2.00E-01	6.90E-01	1.3008	0.3794
DDG(18:3)	Jejunum	POS	Recovery	Recovery	Recovery	-	2.52E-07	1.87E-04	0.3017	-1.7288	5.07E-03	8.41E-01	1.5441	0.6268	4.79E-06	3.55E-03	2.8644	1.5182	7.07E-01	1.00E+00	1.1495	0.2010	7.22E-01	8.93E-01	0.9158	-0.1269	8.44E-01	1.00E+00	1.0025	0.0037
PC(P-19:1/0:0)	Jejunum	POS	-	Recovery	Recovery	Recovery	1.55E-04	9.03E-03	2.8683	1.5202	9.16E-01	1.00E+00	1.0630	0.0882	9.77E-02	5.18E-01	0.7224	-0.4690	5.42E-01	1.00E+00	1.3540	0.4372	8.35E-01	9.37E-01	0.8597	-0.2180	3.06E-01	1.00E+00	0.5966	-0.7451
Docosahexaenoylglycine	Jejunum	POS	Recovery	Recovery	Recovery	Recovery	1.56E-04	9.03E-03	0.5836	-0.7770	9.26E-04	5.73E-01	1.4764	0.5621	2.84E-05	5.86E-03	1.6574	0.7289	8.93E-01	1.00E+00	1.0633	0.0885	9.26E-01	9.78E-01	0.9517	-0.0714	8.56E-01	1.00E+00	0.9555	-0.0656
F-dM-R-F-NH2	Jejunum	POS	-	-	-	-	1.75E-04	9.57E-03	0.5306	-0.9144	9.10E-01	1.00E+00	0.9842	-0.0230	9.14E-01	9.98E-01	0.9975	-0.0037	1.44E-01	1.00E+00	0.8283	-0.2718	6.48E-01	8.65E-01	0.8924	-0.1642	8.47E-01	1.00E+00	0.9565	-0.0642
LysoPC(18:1)	Jejunum	POS	-	Recovery	Recovery	Recovery	1.91E-04	9.94E-03	2.1629	1.1130	9.68E-01	1.00E+00	1.0662	0.0924	1.18E-01	5.76E-01	0.7858	-0.3477	5.27E-01	1.00E+00	1.2975	0.3757	8.24E-01	9.33E-01	0.8638	-0.2113	2.97E-01	1.00E+00	0.6438	-0.6353
Didecanoyl Lecithin	Jejunum	POS	-	-	Recovery	Recovery	2.22E-03	4.10E-02	2.0787	1.0557	7.96E-01	1.00E+00	1.1627	0.2175	3.85E-01	8.92E-01	1.2150	0.2810	5.77E-02	1.00E+00	2.3286	1.2195	6.69E-01	8.72E-01	0.6329	-0.6600	5.96E-02	1.00E+00	0.3771	-1.4068
Tauroursodeoxycholic acid	Jejunum	POS	Recovery	Recovery	Recovery	Recovery	2.25E-03	4.10E-02	0.7106	-0.4929	7.38E-02	1.00E+00	1.2323	0.3013	2.51E-03	5.92E-02	1.4835	0.5690	8.31E-01	1.00E+00	0.9604	-0.0584	1.32E-01	7.15E-01	1.2454	0.3166	6.99E-01	1.00E+00	1.0563	0.0790
IAK	Jejunum	POS	Recovery	Recovery	-	-	1.30E-02	1.02E-01	1.7418	0.8006	2.67E-01	1.00E+00	0.8084	-0.3070	4.93E-04	2.70E-02	0.3816	-1.3897	1.13E-02	6.82E-01	0.6396	-0.6447	4.71E-01	7.89E-01	0.8733	-0.1955	2.29E-01	1.00E+00	0.7418	-0.4310
Tauroursodeoxycholic acid	Jejunum	POS	Recovery	Recovery	Recovery	-	1.74E-01	3.83E-01	0.8024	-0.3176	2.91E-01	1.00E+00	1.3592	0.4428	7.99E-04	3.03E-02	2.1350	1.0942	1.71E-01	1.00E+00	0.7113	-0.4914	1.33E-01	7.15E-01	1.3461	0.4288	7.44E-01	1.00E+00	0.9656	-0.0504
L-Citrulline	Lung	NEG	Recovery	Recovery	-	Recovery	2.62E-05	2.13E-03	0.5120	-0.9657	3.64E-01	9.56E-01	1.1300	0.1763	1.87E-01	7.92E-01	1.1790	0.2376	3.01E-01	1.00E+00	0.9062	-0.1422	8.58E-01	9.25E-01	0.9923	-0.0112	1.81E-01	3.11E-01	1.1674	0.2233
GPEtn(18:0p/22:6)	Lung	NEG	-	Recovery	Recovery	Recovery	7.16E-05	4.82E-03	0.6926	-0.5299	3.65E-01	9.56E-01	0.9227	-0.1161	6.73E-02	5.43E-01	1.2619	0.3356	9.62E-03	2.25E-01	0.8407	-0.2504	1.88E-01	4.24E-01	1.0948	0.1307	2.52E-01	3.90E-01	1.0937	0.1292
Cer(D18:1/18:1)	Lung	NEG	Recovery	Recovery	-	-	3.78E-04	1.21E-02	0.6075	-0.7190	1.88E-01	9.56E-01	1.1572	0.2107	4.02E-03	1.91E-01	1.5371	0.6202	6.56E-01	1.00E+00	1.0491	0.0691	1.26E-02	3.47E-01	1.2727	0.3479	1.03E-01	2.16E-01	1.2386	0.3087
Prostaglandin B3	Lung	NEG	-	-	-	-	4.03E-03	3.76E-02	0.7432	-0.4282	6.10E-01	9.56E-01	0.9553	-0.0660	7.46E-01	9.83E-01	0.9773	-0.0331	7.77E-01	1.00E+00	0.9640	-0.0529	6.42E-02	3.47E-01	0.8508	-0.2331	1.86E-02	1.03E-01	0.7849	-0.3495
Omdm-2	Lung	POS	Recovery	Recovery	-	-	7.02E-02	1.67E-01	0.8485	-0.2371	1.30E-05	1.96E-02	6.5501	2.7115	1.60E-09	2.83E-06	46.0420	5.5249	3.68E-01	1.00E+00	1.0847	0.1173	2.07E-08	3.66E-05	3.2211	1.6875	1.79E-13	5.62E-10	21.8780	4.4514
Lyso-SM	Lung	POS	-	Recovery	-	Recovery	9.32E-04	2.48E-02	0.6435	-0.6361	6.05E-01	1.00E+00	0.9616	-0.0566	3.55E-01	8.63E-01	1.1319	0.1787	1.18E-02	5.91E-01	0.7995	-0.3229	3.07E-01	3.89E-01	0.9302	-0.1044	3.95E-01	4.60E-01	1.0832	0.1153
PE(16:0/20:4)	Lung	POS	-	-	Recovery	Recovery	1.42E-03	3.04E-02	0.6435	-0.6360	7.32E-01	1.00E+00	0.9695	-0.0447	5.91E-01	8.63E-01	0.9538	-0.0682	8.60E-01	1.00E+00	1.0059	0.0084	6.32E-02	1.13E-01	0.8198	-0.2867	1.40E-02	5.72E-02	0.7404	-0.4336
